# Multicomponent Oxidative Nitrile Thiazolidination Reaction for Selective Modification of N-terminal Dimethylation Posttranslational Modification

**DOI:** 10.1021/jacs.3c02369

**Published:** 2023-07-24

**Authors:** Benjamin Emenike, Julia Donovan, Monika Raj

**Affiliations:** Department of Chemistry, Emory University, Atlanta, Georgia 30322, United States

## Abstract

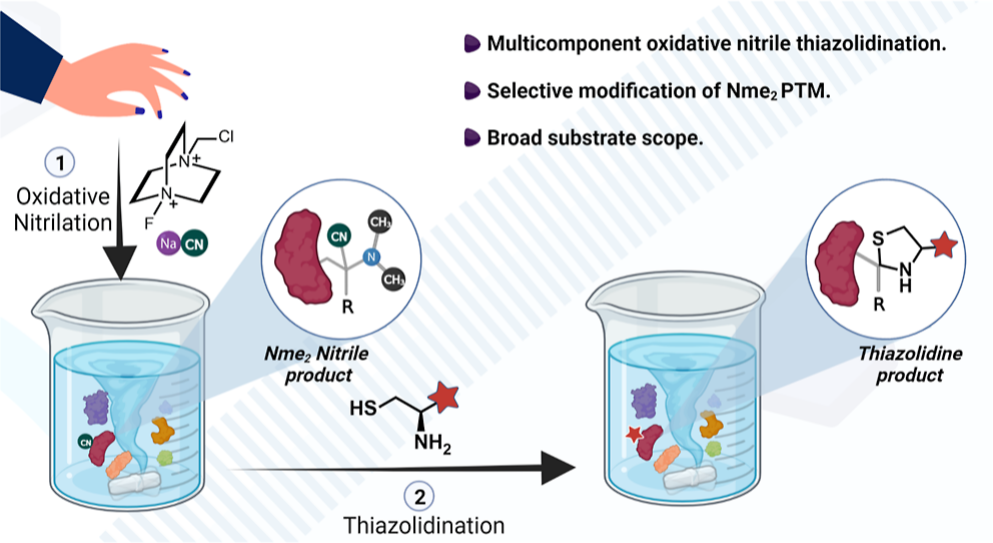

Protein α-N-terminal dimethylation (Nme_2_) is an underexplored posttranslational modification (PTM) despite the increasing implications of α-N-terminal dimethylation in vital physiological and pathological processes across diverse species; thus, it is imperative to identify the sites of α-N-terminal dimethylation in the proteome. So far, only ∼300 α-N-terminal methylation sites have been discovered including mono-, di-, and tri-methylation, due to the lack of a pan-selective method for detecting α-N-terminal dimethylation. Herein, we introduce the three-component coupling reaction, oxidative nitrile thiazolidination (OxNiTha) for chemoselective modification of α-Nme_2_ to thiazolidine ring in the presence of selectfluor, sodium cyanide, and 1,2 aminothiols. One of the major challenges in developing a pan-specific method for the selective modification of α-Nme_2_ PTM is the competing reaction with dimethyl lysine (Kme_2_) PTM of a similar structure. We tackle this challenge by trapping nitrile-modified Nme_2_ with aminothiols, leading to the conversion of Nme_2_ to a five-membered thiazolidine ring. Surprisingly, the 1,2 aminothiol reaction with nitrile-modified Kme_2_ led to de-nitrilation along with the de-methylation to generate monomethyl lysine (Kme_1_). We demonstrated the application of OxNiTha reaction in pan-selective and robust modification of α-Nme_2_ in peptides and proteins to thiazolidine functionalized with varying fluorescent and affinity tags under physiological conditions. Further study with cell lysate enabled the enrichment of Nme_2_ PTM containing proteins.

## Introduction

**Figure 1:**
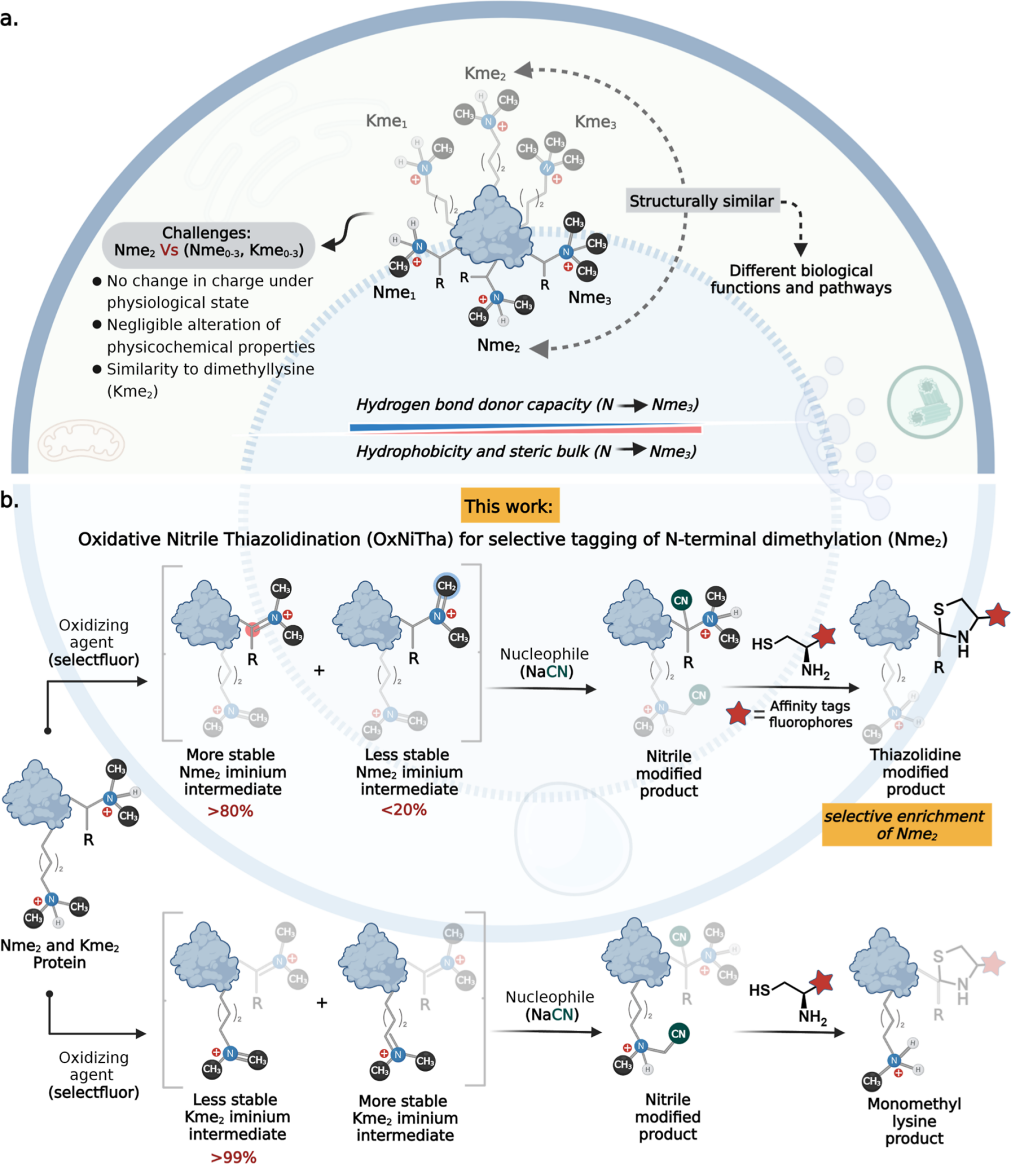
OxNiTha for the selective labeling of Nme_2_-PTM. (a) No change in charge and negligible change in physicochemical properties of Nme_2_ as compared to lysine, N-terminus, and their methylation analogues. (b) OxNiTha step-wise explanation of selective labeling of Nme_2_ by oxidation of Nme_2_ to iminium ion followed by trapping with NaCN and subsequent reaction with amino-thiols to generate N-terminal thiazolidine ring. Trapping of a nitrile group in Kme_2_ leads to de-nitrilation and de-methylation to generate Kme_1_.

Although α-N-terminal dimethylation (Nme_2_) posttranslational modification (PTM) has been discovered four decades ago,^1−3^ it recently gained significant attention due to its emerging role in regulating various biological processes, including DNA repair, epigenetics, translation fidelity, mitosis, genome stability, and its implications in numerous human disorders such as cancer, inflammation, neurodegenerative, and cardiovascular disorders.^4−11^ Moreover, previous studies with eukaryotes showed that the N-terminus methylation takes place at the conserved canonical sequence A/S-PK, but recent studies with prokaryotes and humans showed several non-canonical sequences that are methylated at the N-terminus, thus supporting that Nme_2_ is a widespread PTM.^12−14^ In contrast to dimethyl lysine (Kme_2_) PTM, Nme_2_ is largely underexplored due to the lack of affinity reagents and antibodies for its identification. The current method to identify Nme_2_ PTM involves the use of mass spectrometry (MS) but there are several challenges to its accurate identification by MS;^15,16^ (i) low natural abundance of Nme_2_ PTM in complex mixtures; (ii) lack of affinity agents to selectively enrich Nme_2_ PTMs; (iii) change in mass by two methyl groups (28 Da) on the N-terminus is identical to the N-formylation and Kme_2_ PTMs (28 Da) and identical to the mass difference between Ala Vs Val (28 Da), leading to the false identification. So far, only ∼300 N-methylation sites (including mono-, di-, and tri-*N*-methyl states) have been discovered despite evidence of their vast existence in varying species.^12−14^ To completely understand the role of Nme_2_ PTM, its global identification is required which is possible by a pan-selective chemical method for labeling Nme_2_ sites in a proteome. The major challenge in developing such a chemical method for the selective tagging of Nme_2_ is the small size of the dimethyl group, which leads to negligible alteration in the protein’s physiochemical properties such as bulk, charge, and hydrophobicity, as compared to the free N-terminus, lysine, and their mono-, di-, and tri-methyl analogues (Figure 1a). Herein, we report a chemoselective multicomponent, oxidative nitrile thiazolidination (OxNiTha) reaction for the selective covalent modification of Nme_2_ to a five-membered thiazolidine ring, a first to our knowledge (Figure 1b). Although both Nme_2_ and Kme_2_ are tertiary amines, we demonstrated that this strategy is pan-specific and selectively labels Nme_2_ in the presence of Kme_2_ on the same peptide, independent of the sequence and nearby PTMs (Figure 1b). Moreover, we showed that the selective labeling of Nme_2_ is independent of the nature of amino acid residue at the N-terminus. We demonstrated the robustness and application of the OxNiTha reaction for selective tagging of Nme_2_ peptides and proteins with varying cargos such as affinity tags and fluorophores in a complex cell lysate. There are no other pan-specific chemical methods for the selective labeling of Nme_2_.

## Results and Discussion

### Design and Development of Nme_2_-Selective Reactions

**Figure 2:**
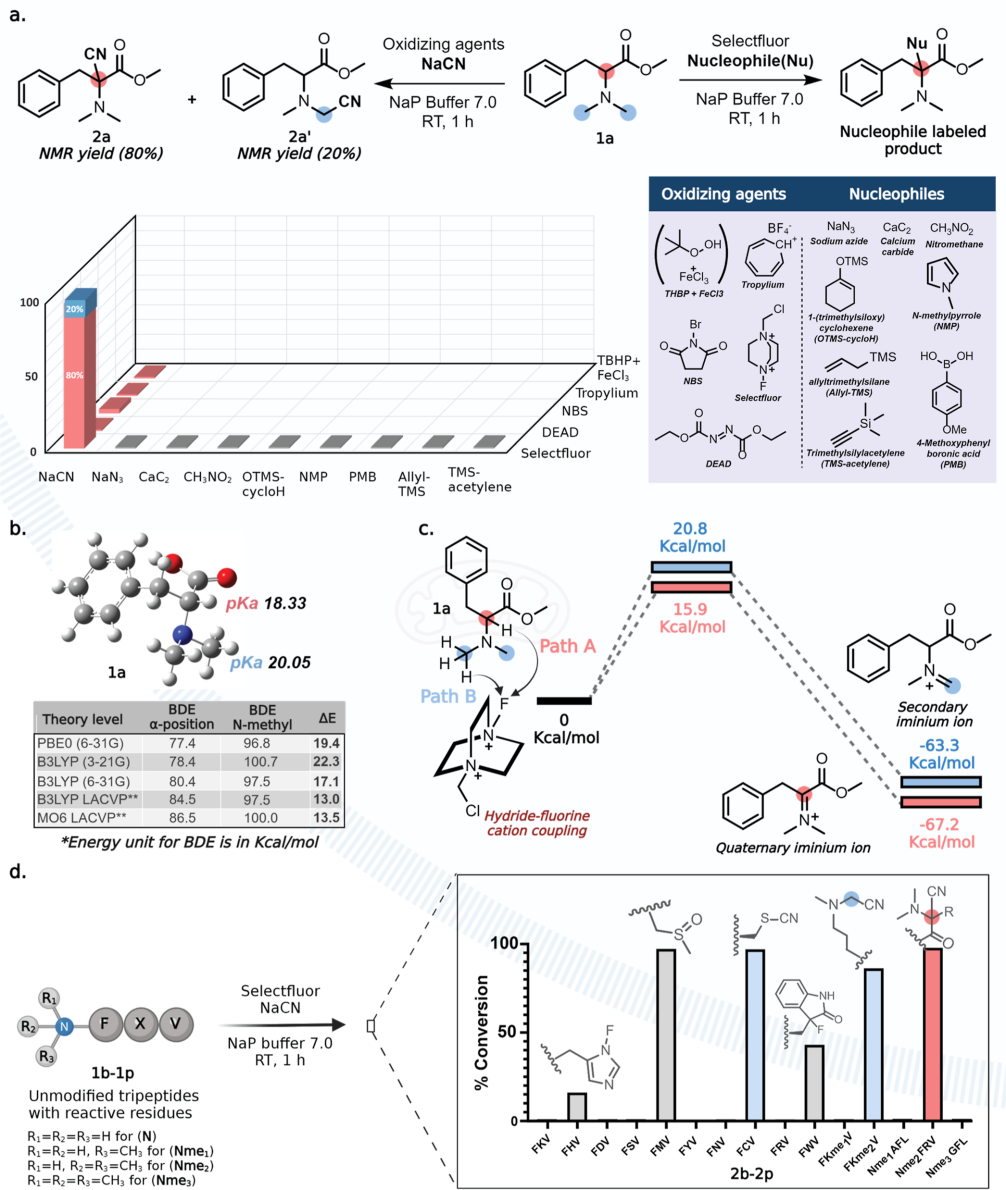
Development of a chemical method for selective modification of Nme_2_. (a) Evaluation of oxidizing reagents and nucleophiles for Nme_2_ modification. (b) DFT calculation of p*K*_a_ and BDEs of more hindered and less hindered proton of Nme_2_-Phe-OMe. (c) Energy of transition states and iminium intermediates for path A and path B. Quaternary iminium ion has lower energy than secondary iminium ion. (d) Chemoselectivity evaluation of OxNiTha on tripeptides bearing various reactive residues including, Nme_1_, Nme_2_, and Nme_3_.

To develop a chemical method for the selective labeling of Nme_2_, we exploited the ability of tertiary amines to form electrophilic iminium ions followed by nucleophilic addition under physiological conditions, resulting in the labeling of tertiary amines with varying nucleophiles (Figure 2a).^17−19^ We screened several oxidizing reagents such as ^*t*^BuOOH (TBHP) with FeCl_3_, tropylium tetrafluoroborate, *N*-bromo succinimide (NBS), diethyl azodicarboxylate (DEAD), selectfluor,^20^ and various nucleophiles such as sodium cyanide (NaCN), sodium azide (NaN_3_), calcium carbide (CaC_2_), nitromethane (CH_3_NO_2_), 1-(trimethyl siloxy)cyclohexene (OTMS-cycloH), *N*-methyl pyrrole (NMP), allyltrimethyl silane (Allyl-TMS), trimethylsilyl acetylene (TMS-acetylene) and 4-methoxy phenyl boronic acid (PMB) on a model small molecule, Nme_2_-Phe-OMe **1a** (Figure 2a, Supporting Information Figure 1). The maximum modification of Nme_2_-Phe-OMe **1a** was observed with selectfluor as an oxidizing reagent and NaCN as a nucleophile to generate the nitrilated product CN-Nme_2_-Phe-OMe (Figure 2a, Supporting Information Figure 1). The NMR analysis of the isolated product showed the nitrilation at two different positions, a major α-nitrile product **2a** with nitrile at the α-position (more substituted) and a minor *N*-methyl-nitrile product **2a′** with nitrile at the methyl group of the N-terminus in the ratio of (4:1) (Figure 2a, Supporting Information Figure 2). The reaction with other nucleophiles led to the de-methylation and the formation of Nme-Phe-OMe (Supporting Information Figure 3).

### Computational Evaluation of Reaction Regioselectivity

To gain mechanistic insights into the observed regioselectivity for α-nitrile product **2a** (∼80%), we turned to the computational study of the key steps in the reaction scheme that are presented in Figure 2b,c. Our extensive density functional theory (DFT) calculations at various levels of theory identified that the more substituted α-position on **1a** has a lower p*K*_a_ (18.33) and lower bond dissociation energy (BDE, ∼77–86) as compared to the less-substituted *N*-methyl group (p*K*_a_ = 20.05 and BDE, ∼96–100) (Figure 2b, Supporting Information Figure 4). Further DFT calculations showed that the free-energy barrier of the transition state for the generation of quaternary iminium intermediate generated from the oxidation via path A is of lower energy (15.9 kcal/mol) as compared to the transition state for the generation of secondary iminium intermediate formed via path B (20.8 kcal/mol, Figure 2c, Supporting Information Figure 4). A similar observation was made when the free energy for iminium ions was calculated for both pathways (Supporting Information Figure 4). These computational observations are consistent with the experimental results presented above; therefore, it is conceivable to conclude that the major controlling factors for the regioselective formation of the α-nitrile product are a combination of favorable enthalpic and thermodynamic properties.

Furthermore, the hydride-fluorine cation coupling mechanism observed for the selecfluor-mediated oxidation of Nme_2_ is unique and differs significantly from those reported in the literature.^21^ Selectfluor is known to undergo either a single electron or two electron transformations. We carried out computational and experimental evaluation of single electron processes using radical scavengers such as 2,2,6,6-tetramethylpiperidinyloxy, 9-azabicyclo[3.3.1]nonane *N*-oxyl, and butylated hydroxytoluene but a radical mechanism for the oxidation of **1a** by selectfluor was not observed (Supporting Information Figure 4). Two electron pathways often involve the direct fluorination of electron-rich nucleophiles such as amines and alkenes.^22^ Our extensive DFT calculations showed no direct fluorination of the nitrogen atom of **1a** by selectfluor (Supporting Information Figure 4). Instead, we observed two transition states which led to the formation of the quaternary iminium ion (path A) and the secondary iminium ion (path B) (see Supporting Information Figure 4). The close analysis of charge distributions, geometries, and intrinsic reaction coordinates clearly identifies the oxidation process to proceed through a hydride-fluorine cation coupling process, as observed from the elongation of the activated C–H bonds of **1a** and N–F bonds of selectfluor and the formation of a nascent H–F bond. The full mechanistic study of this reaction is out of the scope of the current report, and work in this direction is currently ongoing in our laboratory.

### Chemoselectivity Studies

The studies with peptides FXV **1b–1p** containing reactive amino acids (X = K, H, D, S, M, Y, N, C, R, and W), varying lysine methylation states (Kme_1_ and Kme_2_), and peptides with N-terminal monomethyl (Nme_1_-AFL, **1n**), dimethyl (Nme_2_-FRV, **1o**), and trimethyl (Nme_3_-GFL, **1p**) groups showed that the reaction is chemoselective for nitrilation of tertiary amines and generated nitrile-modified FKme_2_V **2m** and nitrile-modified Nme_2_FRV **2o** products in high conversions (82 and 98% respectively, Supporting Information Figure 5). Notably, nitrilation of Kme_2_ occurred at the less hindered position (82%) under the reaction conditions, which was confirmed by NMR analysis of the product obtained from a small molecule mimic of nitrile-modified Kme_2_ (Supporting Information Figure 6). This reversal of regioselectivity in Kme_2_ as compared to Nme_2_ is mainly due to the absence of the amide group at the side chain of lysine. Furthermore, we observed the oxidation of Met (98%), thiocyanate formation with cysteine (98%), and fluorination of Trp (43%) and His (8%) under the reaction conditions, but none of these side products would interfere in the analysis of the nitrile-modified Nme_2_ product. All the observed side-adducts were synthesized on model small molecules and characterized by NMR (Supporting Information Figure 7).

### Development of OxNiTha for Selective Modification of Nme_2_

**Figure 3:**
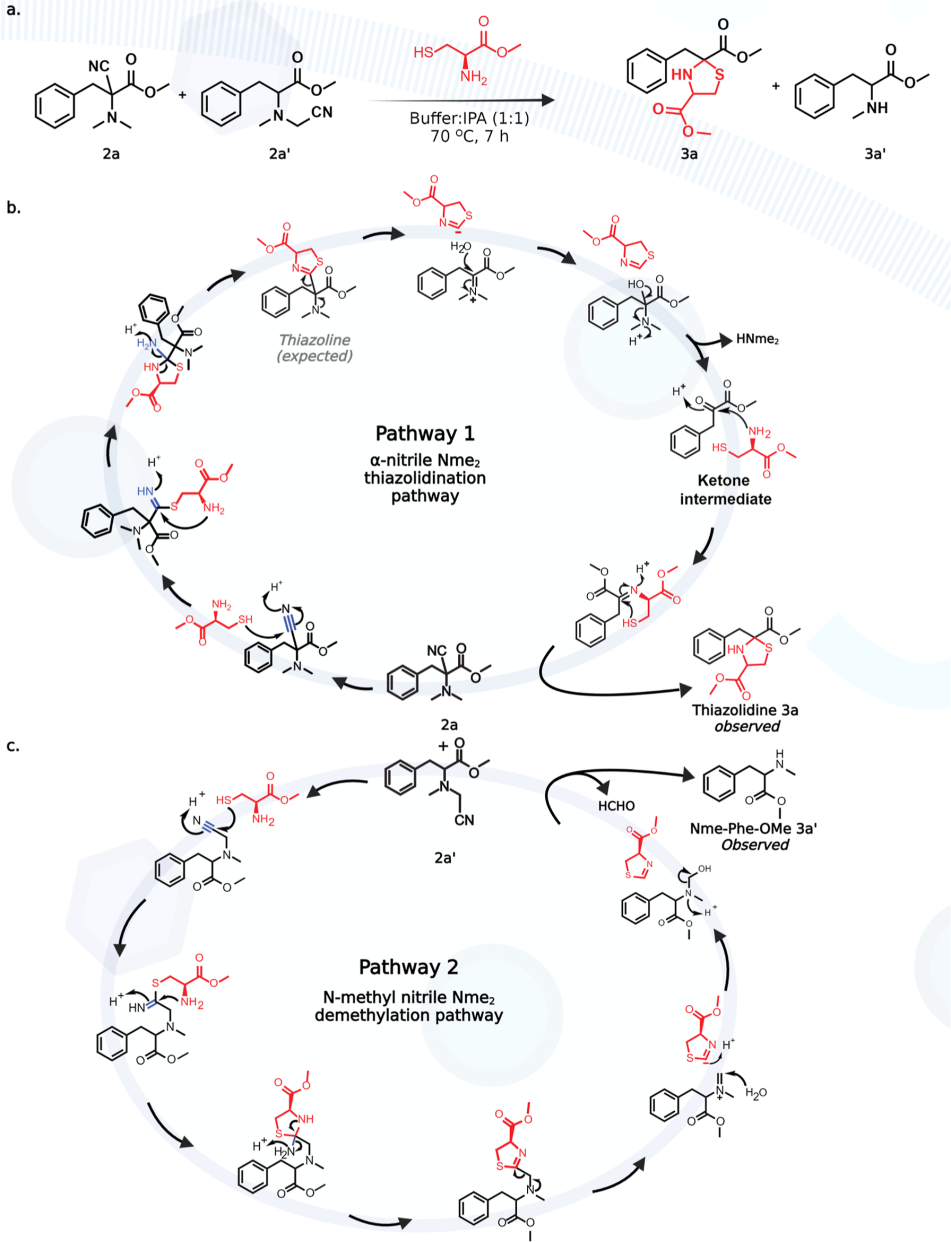
Selective enrichment of *N*,*N*-dimethyl peptide fragments. (a) Formation of thiazolidine product **3a** and de-methylated Nme-Phe-OMe product **3a′** with nitrile-substituted Nme_2_-Phe-OMe **2a** and **2a′.** (b) Plausible mechanism for the observed thiazolidine **3a** product from **2a** upon reaction with cysteine methylester. (c) Plausible mechanism for the observed de-methylated product **3a′** from **2a′** upon reaction with cysteine methylester.

Since chemoselective studies on peptides showed the nitrilation of Nme_2_, Kme_2_, and Cys containing peptides, we sought to develop a strategy for the selective modification and tagging of nitrile-modified Nme_2_ peptides in the presence of nitrile-modified Kme_2_ and cysteine peptides. To achieve this goal, we attempted the functionalization of the nitrile-modified small molecules α-nitrile-Phe-OMe **2a** and *N*-methyl nitrile-Phe-OMe **2a′** with cysteine methylester to generate a thiazoline product via thioimidate intermediate. Surprisingly, we observed the modification of Nme_2_ to thiazolidine-Phe-OMe **3a** at the N-terminus in >90% conversion, as confirmed by NMR (Figure 3a, Supporting Information Figure 8). We hypothesized that the formation of a thiazolidine ring from the nitrile-Nme_2_ product is due to the hydrolysis of thiazoline intermediate due to the nearby tertiary amine, which resulted in the formation of ketone intermediate followed by trapping of ketone with cysteine (Figure 3b, pathway 1). We also observed ∼10% of de-nitrilated and de-methylated product, Nme-Phe-OMe **3a′**, under the reaction conditions as confirmed by NMR (Supporting Information Figure 8). We hypothesized that the de-nitrilation and de-methylation from the *N*-methyl nitrile product **2a′** is due to the cysteine-mediated formation of iminium ion followed by the hydrolysis and the release of formaldehyde (Figure 3c, pathway 2). The incubation of a mixture of **2a** and **2a′** (50 mg) in 2 mL of 1:1 [NaP buffer (10 mM, pH 7)/IPA] at room temperature and 80 °C for 24 h without cysteine did not lead to any hydrolysis and de-nitrilation of **2a** and **2a′**. This experiment further confirms the key role of cysteine in the formation of the de-nitrilated product with **2a′** (Supporting Information Figure 8).

**Figure 4:**
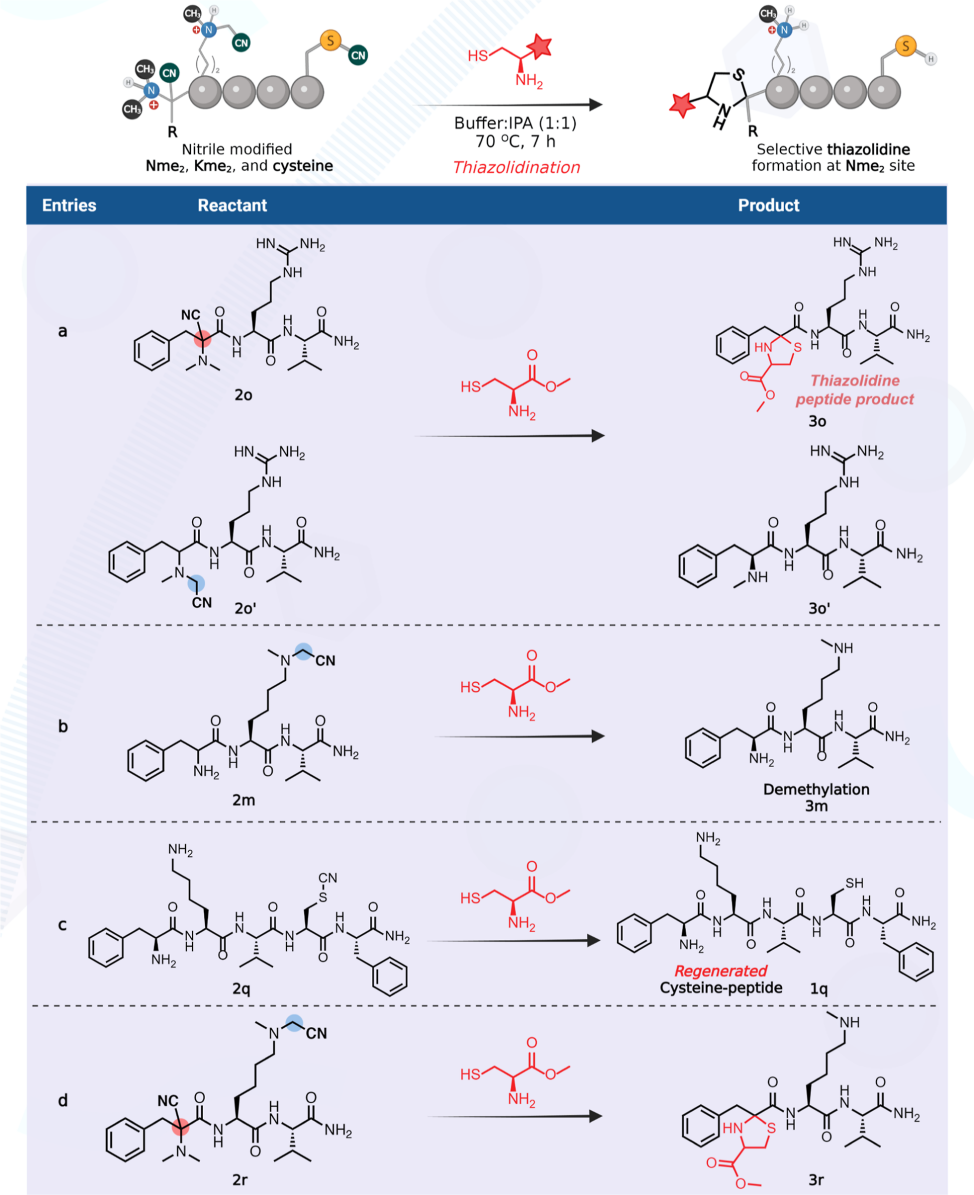
Modification of nitrile containing *N*,*N*-dimethyl (Nme_2_), Kme_2_, and cysteine peptides. (entry a) Modification of nitrile containing *N*,*N*-dimethyl (Nme_2_) peptide to the thiazolidine peptide product. (entry b) De-methylation of nitrile-modified Kme_2_ upon the reaction with cysteine methylester. (entry c) Reaction of thiocyanate-modified peptide with cysteine methylester regenerated unmodified cysteine. (entry d) Selective thiazolidine formation on the Nme_2_ site and de-methylation on the Kme_2_ site by cysteine methylester.

Next, we attempted similar reactions on nitrile-modified peptides, Nme_2_-Phe-Arg-Val **2o** and **2o′** with cysteine methyl ester, and observed the formation of thiazolidine **3o** (>99% conversion) from **2o** and de-methylation with **2o′** to generate a monomethyl N-terminal amine Nme-Phe-Arg-Val **3o′** (∼10%) (Figure 4, entry a, Supporting Information Figures 9 and 10). With nitrile-modified Kme_2_ peptide, Phe-Kme_2_-Val **2m**, we observed the de-methylation on the reaction with cysteine methylester, leading to the formation of monomethyl lysine Kme peptide, Phe-Kme-Val **3m** (Figure 4, entry b, Supporting Information Figures 11 and 12). This is again via pathway 2, involving the formation of iminium ion followed by hydrolysis and release of formaldehyde (for the detailed proposed mechanistic pathway, see Supporting Information Figure 13). The reaction of the cysteine methylester with nitrile-modified cysteine peptide **2q** led to the de-nitrilation and generated unmodified cysteine peptide **1q** (Figure 4, entry c, Supporting Information Figures 14 and 15 for the proposed mechanistic pathway). Similarly, the reaction on a dual-nitrilated peptide with nitrilation on both Nme_2_ and Kme_2_, Nme_2_-Phe-Kme_2_-Val **2r**, resulted in the formation of a single thiazolidine product by the modification of Nme_2_ and de-methylation of nitrilated-Kme_2_ to Kme to generate **3r** in >90% conversion (Figure 4, entry d, Supporting Information Figure 16). Overall, these studies showed that Nme_2_ could be selectively modified to thiazolidine and thus identifiable from a mixture containing reactive amino acids and Kme_2_ PTM.

### Stability of the Nme_2_-Thiazolidine Product

**Figure 5:**
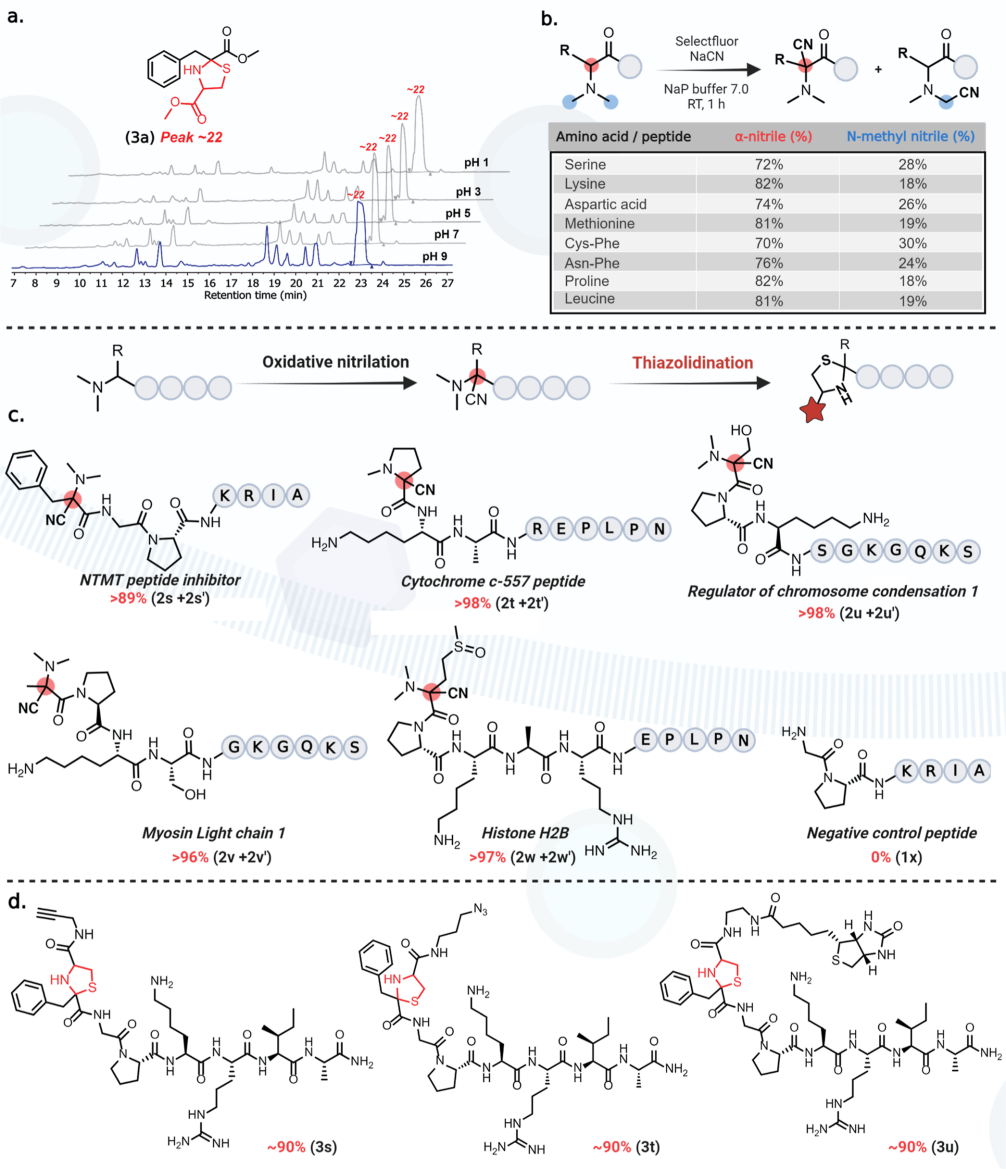
Stability studies and substrate scope of OxNiTha (a) stability studies of the thiazolidine product **3a** across a wide range of pH conditions. (b) OxNiTha of varying *N*,*N*-dimethyl amino acids and peptides containing reactive and bulky amino acids at the N-terminus. (c,d) Pan-specificity studies on N-terminal methyltransferase (NTMT) peptide substrates showed that OxNiTha is independent of the nature of amino acids and cysteine-based affinity tags.

Next, we explored the stability of the α-thiazolidine-Phe-OMe **3a** by incubating it in solutions with varying pH conditions pH 1–9 for 24 h (Figure 5a). No degradation of α-thiazolidine-Phe-OMe **3a** was observed under the reaction conditions. This result provides experimental support for the suitability of the OxNiTha reaction for further downstream modification and tagging of Nme_2_-containing peptides and proteins.

### Substrate Scope with Varying N-terminal Amino Acids

To determine the substrate scope, we carried out reactions with varying Nme_2_ amino acids and peptides containing reactive side chains (e.g., Ser, Lys, Asp, Met, Cys–Phe, and Asn–Phe) and bulky amino acids (e.g., Pro and Leu) at the N-terminus using selectfluor and NaCN under the optimized conditions (Figure 5b, Supporting Information Figures 17 and 18). We observed the high yields of more-substituted α-nitrile products with small amounts of *N*-methyl nitrile products in most cases in the ratio of (4:1) independent of the nature of the side group at the N-terminus, as determined by the NMR (Figure 5b, Supporting Information Figure 18).

### Pan-Specificity: Further Diversification

With the optimized conditions for the formation of α-thiazolidine from Nme_2_, we next demonstrated the pan-specificity by carrying out the OxNiTha reaction with various peptides of different sizes and amino acid compositions including NTMT peptide substrates, which are known to be frequently dimethylated at the N-terminus. Dysregulation in the α-N-terminal methylation of these NTMT substrates has been implicated across various cancers and aging processes.^10,11^ Using solid-phase peptide synthesis,^23^ we synthesized N-terminal sequences of NTMT peptide substrates^24^ Nme_2_-Phe-Gly-Pro-Lys-Arg-Ile-Ala **1s**, Nme_1_-Pro-Lys-Ala-Arg-Glu-Pro-Leu-Pro-Asn (cytochrome *c*-557, **1t**), Nme_2_-Ser-Pro-Lys-Ser-Gly-Lys-Gly-Gln-Lys-Ser (regulator of chromosome condensation RCC1, **1u**), Nme_2_-Ala-Pro-Lys-Ser-Gly-Lys-Gly-Gln-Lys-Ser (myosin light chain 1, **1v**), Nme_2_-Met-Pro-Lys-Ala-Arg-Glu-Pro-Leu-Pro-Asn (histone H2B, **1w**) along with a negative control sequence without Nme_2_, Gly-Pro-Lys-Arg-Ile-Ala **1x** (see Supporting Information Figure 19). Under the optimized reaction conditions, we observed the quantitative conversion of all the peptides to the nitrile-peptide products (**2s–2w** and **2s′–2w′**) (Figure 5c, Supporting Information Figure 20). The reaction with a control peptide without the Nme_2_ (Gly-Pro-Lys-Arg-Ile-Ala, **1x**) did not generate any modified product under the reaction conditions. Next, we synthesized affinity tag-modified cysteine analogues with alkyne, azide, and biotin groups (for synthesis see, Supporting Information Figure 21) and carried out selective thiazolidination of the nitrile-modified Nme_2_ peptides **2s** and **2s′**. We observed ∼90% conversion to the α-thiazolidine peptides (**3s–3u**) along with a very small amount (∼5–10%) of de-methylation product **3s′** obtained from the less substituted *N*-methyl nitrile **2s′** product (Figure 5d, Supporting Information Figure 22).

### Selective Labeling of Nme_2_ Peptides in a Complex Cell Lysate Mixture

**Figure 6:**
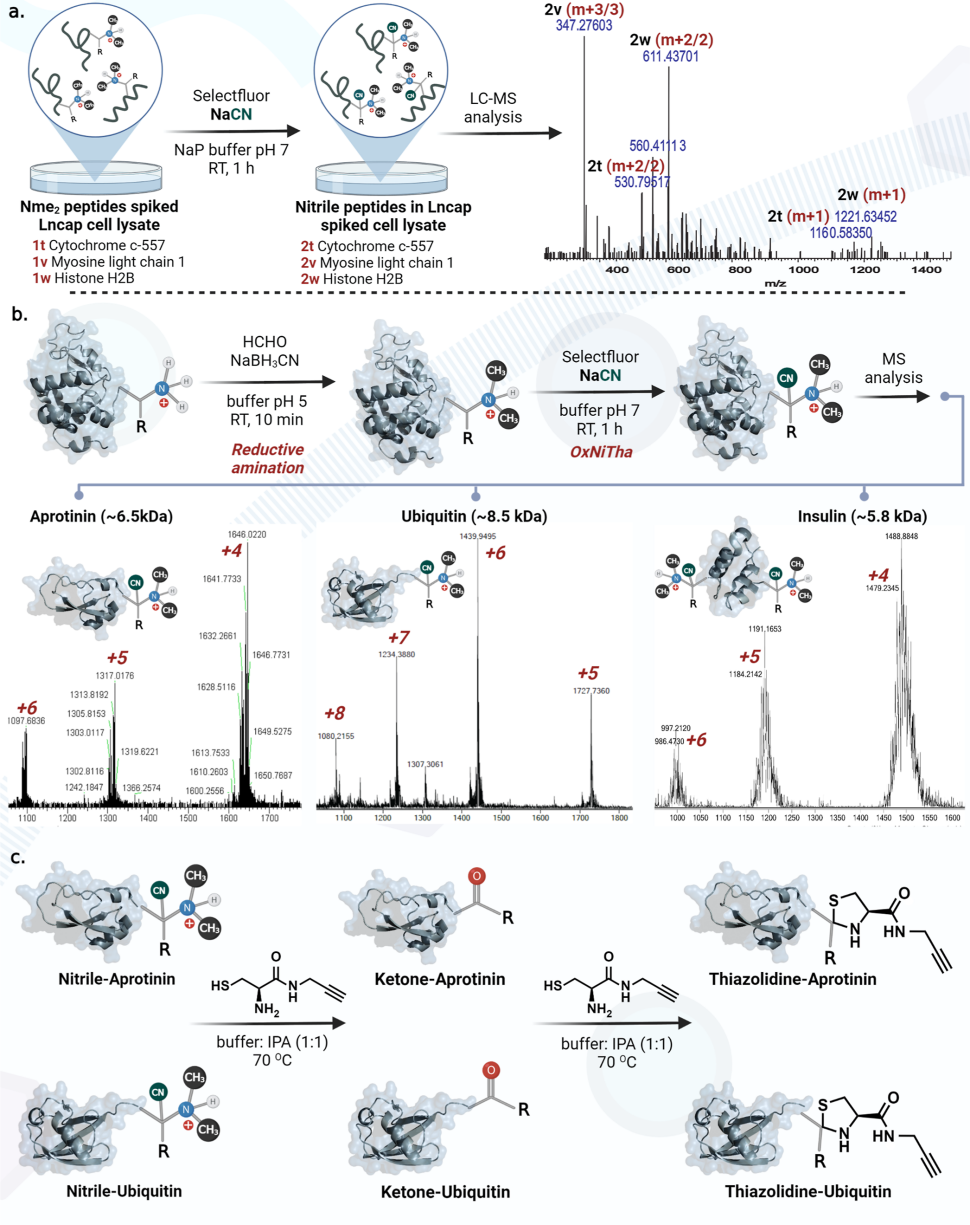
OxNiTha reaction for the selective modification of Nme_2_ peptides and proteins in a complex mixture. (a) Selective modification of Nme_2_ peptides in a complex cell lysate. (b) Selective modification of Nme_2_ proteins. (c) Selective diversification of Nme_2_ proteins with cysteine-alkyne analogue.

To determine the ability of the OxNiTha reaction to label low abundant Nme_2_ peptides in a complex mixture, prostate cancer cell lysate (LnCap) was spiked with three NTMT-derived Nme_2_ peptides of different amino acid compositions and sizes, Nme_1_-Pro-Lys-Ala-Arg-Glu-Pro-Leu-Pro-Asn (cytochrome *c*-557, **1t**), Nme_2_-Ala-Pro-Lys-Ser-Gly-Lys-Gly-Gln-Lys-Ser (myosin light chain 1, **1v**), and Nme_2_-Met-Pro-Lys-Ala-Arg-Glu-Pro-Leu-Pro-Asn (histone H2B, **1w**) (Figure 6a, Supporting Information Figure 23). We incubated the reaction mixture with selectfluor and NaCN for 1 h. The reaction mixture was then analyzed by LCMS, and we observed the formation of nitrile products (**2t**, **2v**, and **2w**) with all three peptides (Figure 6a, Supporting Information Figure 23). No unreacted peptides were observed under the reaction conditions, suggesting the robust and chemoselective nature of the OxNiTha reaction for labeling Nme_2_ in a complex mixture.

### OxNiTha of Nme_2_ Proteins

To demonstrate the compatibility of OxNiTha chemistry on selective labeling of Nme_2_ proteins, we chemically introduced the dimethyl group at the N-terminus of proteins of varying molecular weights; aprotinin (6.5 kDa), ubiquitin (M, 8.5 kDa), and insulin (5.8 kDa) using reductive amination (Figure 6b, Supporting Information Figure 24). Nme_2_ proteins were subsequently subjected to OxNiTha chemistry to generate N-terminally modified nitrile proteins in good conversion, as analyzed by MS (Figure 6b, Supporting Information Figure 25). Along with the formation of the nitrile product with Nme_2_, we observed the oxidation of methionine to sulfoxide in aprotinin and ubiquitin and small amounts of fluorination of histidine in insulin, as characterized by NMR on peptides (Supporting Information Figures 7 and 25). None of these side products interfere during the enrichment and analysis of the Nme_2_ nitrile product. We chose insulin for our studies because it has two N-termini. Notably, both the N-terminus generated *N*,*N*-dimethylation and resulted in di-nitrilation at both the N-termini. Further nitrile-modified proteins, aprotinin and ubiquitin, were treated with cysteine-based alkyne analogue. Surprisingly, we also observed the ketone adducts of the modified aprotinin and ubiquitin by MS, as proposed previously (Supporting Information Figure 26). The continued treatment with cysteine-based alkyne analogue led to the conversion of ketone-modified proteins to α-thiazolidine–aprotinin and α-thiazolidine–ubiquitin, as analyzed by MS (Figure 6c, Supporting Information Figure 26). These results demonstrated the robustness and high efficiency of the OxNiTha chemistry to selectively modify Nme_2_ proteins with affinity tags.

### OxNiTha of the Nme_2_ Cell Lysate

**Figure 7:**
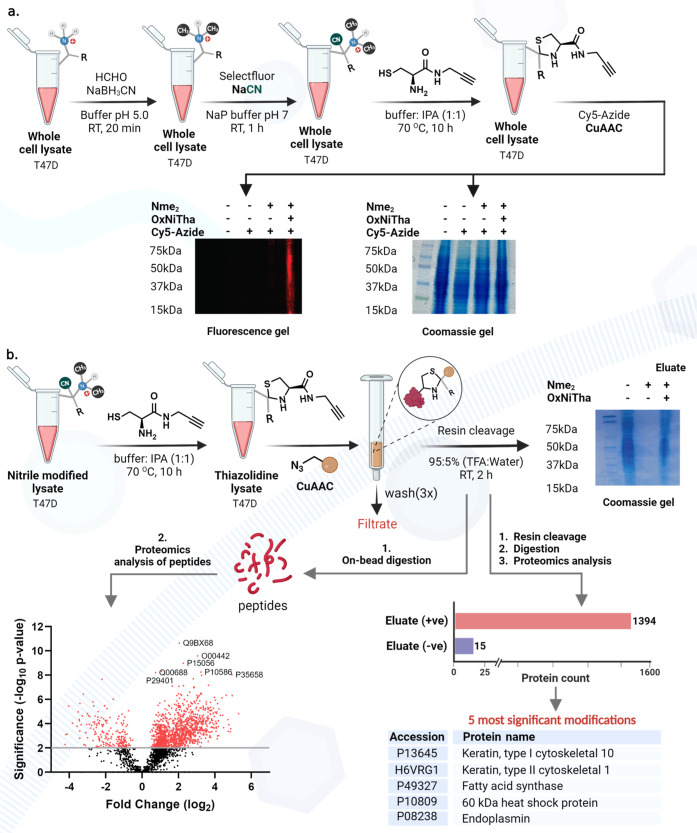
OxNiTha reaction for the selective modification and enrichment of Nme_2_ proteins in a cell lysate. (a) Selective modification of Nme_2_ proteins in a complex cell lysate by fluorophore labeling, as analyzed by gel analysis. High fluorophore labeling was observed under OxNiTha conditions (lane 4). No fluorescence was observed under negative control conditions (lanes 1 and 3). (b) Selective enrichment of Nme_2_ proteins from the cell lysate followed by proteomic analysis on enriched and digested proteins or on bead digestion of proteins. The proteomic analysis of enriched and digested proteins showed capturing of 1394 Nme_2_ proteins as compared to the negative control (15). Top 5 most abundant Nme_2_-modified proteins obtained after enrichment were listed. High enrichment of Nme_2_ proteins was observed under OxNiTha conditions as compared to the negative controls by both pathways. A volcano plot generated by proteomic analysis of enriched and on-bead digested proteins showed the significant enrichment of Nme_2_ proteins.

To further highlight the effectiveness of OxNiTha chemistry in modifying proteins within cell lysates, we conducted experiments on breast cancer cell lysate (T47D). To generate Nme_2_ proteins within the lysate, we treated the lysate with reductive amination reagents (10% formaldehyde and 600 mM sodium cyanoborohydride). Subsequently, we subjected the lysate to OxNiTha chemistry and labeled it with Cy5 azide dye (Figure 7a and Supporting Information Figure 27). Analysis using gel fluorescence clearly revealed specific fluorophore labeling of the thiazolidine-modified cell lysate (Figure 7a, lane 4 and Supporting Information Figure 27). Consequently, no fluorescence was observed in untreated T47D cell lysate and Nme2-modified cell lysate without thiazolidination (Figure 7a, lanes 1–3, Supporting Information Figure 27).

To enrich the Nme_2_-modified proteins in the cell lysate, we first modified Nme_2_ proteins with OxNiTha chemistry to attach nitrile handles on Nme_2_ sites. Next, we incubated nitrile-modified cell lysate with cysteine alkyne to generate thiazolidine alkyne followed by enrichment with azide-functionalized resin using click chemistry. The resin was thoroughly washed to remove non-covalently bound proteins (filtrate), followed by the subsequent release of proteins from the resin under acidic conditions (95% TFA in water). Gel analysis of the eluates clearly showed the release of proteins from the OxNiTha-modified lysates (lane 3), with fewer proteins observed in the negative control Nme_2_ lysate without the thiazolidination step (Figure 7b, lane 2, Supporting Information Figure 28). The enriched proteins were digested followed by proteomics analysis of the digested fragments identifying 1394 proteins for the OxNiTha-modified lysate sample with the most significant being accession numbers P13645, H6VRG1, P49327, P10809, and P08238. In contrast, only 15 proteins were enriched in a negative control cell lysate (Figure 7b, Supporting Information Figure 28).

We also performed on-bead digestion on enriched proteins and proteomic analysis after on-bead digestion clearly showed significant enrichment of Nme_2_ proteins as compared to negative control, as shown by the volcano plot (Figure 7b, Supporting Information Figure 29). These results highlight the robustness of OxNiTha chemistry for selective labeling, enrichment, and profiling of Nme_2_ proteins in a complex cell lysate mixture.

## Conclusions

We introduced the multicomponent chemical method, OxNiTha, for the selective modification and labeling of Nme_2_ PTM in a complex mixture. The reaction works under mild conditions and selectively modifies Nme_2_ to thiazolidine independent of the amino acid sequence and in the presence of other tertiary amine PTMs, such as Kme_2_. We demonstrated the application of OxNiTha chemistry in the selective labeling of Nme_2_ peptides and Nme_2_ proteins with varying affinity tags and fluorophores with high conversions in a complex mixture. Given the high chemoselectivity of this reaction, we demonstrated the application of the OxNiTha method for the fluorescent labeling, enrichment, and proteomic analysis of Nme_2_ proteins from a complex cell lysate mixture. These innovative methods for detecting Nme_2_ PTMs would expand the chemical tool kit available for epigenetics research.

## Supporting Information Available

Optimization of the reaction with varying oxidizing reagents, nucleophiles, and cysteine trapping reagents; reaction procedures; procedure of optimized reactions with peptides, proteins, and cell lysates; and product characterization by NMR, HPLC, and HRMS (PDF)

## Abbreviations

Nme_2_: α-N-terminal dimethylation
PTM: posttranslational modification
OxNiTha: oxidative nitrile thiazolidination
Kme_2_: dimethyl lysine
Kme_1_: monomethyl lysine
MS: mass spectrometry
DFT: density functional theory
BDE: bond dissociation energy
