# Supplementary material for: Multicomponent Oxidative Nitrile Thiazolidination Reaction for Selective Modification of N-terminal Dimethylation Posttranslational Modification
Source: J Am Chem Soc. 2023 Jul 24;145(30):16417–28. doi: 10.1021/jacs.3c02369 (PMC10401698; doi:10.1021/jacs.3c02369)
Supplement: Supplementary file 1 — ja3c02369_si_001.pdf [file ja3c02369_si_001.pdf]

Supporting Information

Multicomponent Oxidative Nitrile Thiazolidination Reaction for Selective Modification of N-terminal dimethylation Posttranslational modification

Benjamin Emenike‡, Julia Donovan‡, Monika Raj‡

Table of content **Pages**

I.	General	2
II.	Materials	2
III.	Purification	2
IV.	Analytical Methods	2-3
V.	Fmoc Solid-Phase Peptide Synthesis	3
VI.	General procedure 1 for oxidative nitrilation of Nme_2 peptides	3
VII.	General procedure 2 for thiazolidination of Nme_2 peptides	3-4
VIII.	Figure 1. Evaluation of oxidizing agents	4-8
IX.	Figure 2. Characterization of Nme_2 nitrile products	8-11
X.	Figure 3. Evaluation of nucleophiles	11-19
XI.	Figure 4. Evaluation of reaction regioselectivity	19-24
XII.	Figure 5. Chemoselectivity studies	24-41
XIII.	Figure 6. Characterization of Kme_2 nitrile products	41-43
XIV.	Figure 7. NMR characterization of chemoselectivity adducts	44-50
XV.	Figure 8. Development of thiazolidination reaction	50-54
XVI.	Figure 9. Nitrilation of Nme_2 peptide 1o to 2o	54-55
XVII.	Figure 10. Thiazolidination of Nme_2 peptide 2o	55-57
XVIII.	Figure 11. Nitrilation of Kme_2 peptide 1m	57-58
XIX.	Figure 12. Thiazolidination of Nme_2 peptide 2m	59-60
XX.	Figure 13. Plausible mechanism for Kme_2 demethylation	60
XXI.	Figure 14. Oxidative nitrile thiazolidination of thiocyanate peptide	61-63
XXII.	Figure 15. Plausible mechanism for thiocyanate reversal	63
XXIII.	Figure 16. Selective enrichment of Nme_2 from Kme_2	64-65
XXIV.	Figure 17. Synthesis of N,N-dimethyl amino acids	65-85
XXV.	Figure 18. Nitrilation of diverse N,N-dimethyl amino acids	86-101
XXVI.	Figure 19. General procedure for N,N-dimethylation of peptides	101
XXVII.	Figure 20. Pan-specific nitrilation of Nme_2 peptides	102-109
XXVIII.	Figure 21. Synthesis of cysteine-based affinity tags	110-117
XXIX.	Figure 22. Thiazolidination mediated cysteine-affinity tag labeling	118-122
XXX.	Figure 23. Nitrilation of cell lysate spiked N,N-dimethyl peptides	122-123
XXXI.	Figure 24. General procedure for N,N-dimethyl protein synthesis	123-127
XXXII.	Figure 25. Nitrilation of N,N-dimethyl proteins	128-131
XXXIII.	Figure 26. Thiazolidination of nitrile modified N,N-dimethyl proteins	131-133
XXXIV.	Figure 27. Thiazolidination of proteins in cell lysate and fluorophore labeling	133-134
XXXV.	Figure 28. Thiazolidination of proteins in cell lysate	134-135
XXXVI.	Figure 29. On-bead digestion of enriched Nme_2 proteins	135-136
XXXVII.	References	136

I. General. All commercial materials (Sigma-Aldrich, Fluka and Novabiochem) were used without further purification. All solvents were reagent or HPLC (Fisher) grade. All reactions were performed under air in glass vials. Yields refer to chromatographically pure compounds; % conversions were obtained by comparing HPLC peak areas of products and starting materials. HPLC and MS were used to monitor reaction progress, and products were characterized using MS and NMR.

II. Materials. Fmoc-amino acids, Rink amide resin, 3-[bis(dimethylamino)methyliumyl]-3H-benzotriazol-1-oxide hexafluorophosphate (HBTU), 1-hydroxy-7-azabenzotriazole (HOAt), N,N′-iisopropylcarbodiimide(DIC), and N,N-diisopropylethylamine (DIEA) were obtained from CreoSalus (Louisville, Kentucky). Piperidine and trifluoroacetic acid (TFA), were obtained from Alfa Aesar (Ward Hill, Massachusetts). N,N-dimethylformamide (DMF), dichloromethane (CH_2Cl_2), methanol (MeOH) and acetonitrile (ACN) were obtained from VWR (100 Matsonford Road Radnor, Pennsylvania). Selectfluor (1-Chloromethyl-4-fluoro-1,4-diazoniabicyclo[2.2.2]octane bis(tetrafluoroborate) and sodium cyanide were obtained from Sigma.

III. Purification. HPLC: Purification of peptides was performed using high performance liquid chromatography (HPLC) on an Agilent 1100 series HPLC equipped with a C-18 reverse phase column with a particle size of 5 μm. All separations involved a mobile phase of 0.1% formic acid in water (solvent A) and 0.1 % formic acid in acetonitrile (solvent B). The HPLC method used a linear gradient of 0-80% solvent B over 30 minutes at ambient temperature with a flow rate of 1 mL min^{-1}. The eluent was monitored by absorbance at 220 nm.

IV. Instrumentation and sample analysis. NMR. ^{1}H and ^{13}C spectra were acquired at 25 °C in DMSO-d_6, $CDCl_3$ using an Agilent DD2 (600 MHz) spectrometer with a 3-mm He triple resonance (HCN) cryoprobe. All ^{1}H NMR chemical shifts (δ) were referenced relative to the residual DMSO-d_6 peak at 2.50 ppm, $CDCl_3$ peak at 7.26 ppm or internal tetramethylsilane (TMS) at 0.00 ppm. ^{13}C NMR chemical shifts were referenced to DMSO-d_6 at 39.52 ppm and $CDCl_3$ at 77.2 ppm. ^{13}C NMR spectra were proton decoupled. NMR spectral data are reported as chemical shift (multiplicity, coupling constants (J), integration). Multiplicity is reported as follows: singlet (s), broad singlet (br s), doublet (d), doublet of doublets (dd), doublet of triplets (td), triplet (t) and multiplet (m). Coupling constant (J) in hertz (Hz).

Analytical HPLC. Analytical HPLC chromatography (HPLC) was performed on an Agilent 1200 series HPLC equipped with a 5 mm C-18 reversed-phase column. The reaction was monitored by analytical reverse phase HPLC using a gradient of water versus acetonitrile. All separations involved mobile phase with 0.1 % formic acid in water (solvent A) and 0.1 % formic acid in acetonitrile (solvent B). Analytical HPLC method used for purification of peptides a linear gradient of 0-80% solvent B over 30 min at room temperature with a flow rate of 1.0 mL min^{-1}. The oxidative tertiary amine reactions were analyzed by HPLC, and MS. HPLC was carried out with 0.1% formic acid: water (solvent A): acetonitrile (solvent B) at detection wavelength 220 nm.
HPLC METHOD A: Gradient: 0 to 80 % **B** (0.1% formic acid in ACN) in 30 min; 80-100 % B in 31-35 min at a flow rate of 1 mL/min.
HPLC METHOD B: Gradient: 0 to 50 % **B** (0.1% formic acid in ACN) in 30 min; 50-100 % B in 31-35 min at a flow rate of 0.5 mL/min.

LC/MS. High resolution LC-MS conditions for all purified peptides: Analyses were performed on an ultraperformance LC system (ACQUITY, Waters Corp., USA) coupled with a quadrupole

time-of-flight mass spectrometer (Q-ToF Premier, Waters) with electrospray ionization (ESI) in positive mode using Mass lynx software (V4.1) or high-performance LC system (Agilent, 1100 series) coupled with triple quadrupole.

LC-MS (Agilent technologies 6460) with electrospray ionization (ESI) in positive mode using Agilent mass hunter (10.0). Unless otherwise mentioned a sample was injected either onto a C4 column (Phenomenex Aeris™ 3.6 µm WIDEPORE C4 200 Å, LC Column 50 x 2.1 mm) with a 400 µL/min flow rate of mobile phase of solution A (90 % H_2O, 10 % acetonitrile and 0.1 % formic acid (FA)) and solution B (95 % acetonitrile, 5 % H_2O, and 0.1 % formic acid) beginning gradient- Time- 0 min 10 % B; 5 min 28 % B; 20 min 38 % B; 22 min 90 % B; C18 column (ACQUITY UPLC BEH 1.7 µm 1x 50 mm) with a 200 µL/min flow rate of mobile phase of solution A (90 % H_2O, 10 % acetonitrile and 0.1 % formic acid) and solution B (90 % acetonitrile, 10 % H_2O, and 0.1 % formic acid) beginning gradient- Time- 1 min 0% B; 1-10 min 100% B for chromatography analysis (or) directly injected with mobile phase 90 % H_2O: 10 % ACN, 0.1% formic acid at 400 µL/min flow rate in ESI positive mode.

HRMS. High resolution MS data were acquired on Thermo Exactive Plus using a heated electrospray source. The solution was infused at a rate of 10-25 µL/min/electrospray using 3.3 KV. The typical settings were Capillary temp 320 ^{o}C. S-lens RF level was between 30-80 with an AGC setting of 1 E6. The maximum injection time was set to 50 ms. Spectra were taken at 140,000 resolutions at m/z 200 using Tune software and analyze with Thermo's Freestyle software.

V. Fmoc Solid-Phase Peptide Synthesis (Fmoc-SPPS).[1] Peptides were synthesized manually on a 0.25 mm scale using Rink amide resin. Resin was swollen with CH_2Cl_2 for 1 h at room temperature. Fmoc was deprotected using 20% piperidine in DMF for 5 min to obtain a deprotected peptide-resin. First, Fmoc protected amino acid (1.25 mm/5 equiv.) was coupled using HOAt (1.25 mm/5 equiv.) and DIC (1.25 mm/5 equiv.) in DMF for 15 min at room temperature. Fmoc-protected amino acids (0.75 mm/3 equiv.) were sequentially coupled on the resin using HBTU (0.75 mm/3 equiv.) and DIEA (1.5 mm/6 equiv.) in DMF for 5 min at room temperature. Peptides were cleaved from the resin using a cocktail of 95:2.5:2.5, trifluoroacetic acid: water:triethylsilane (TES) for 2 h. The resin was removed by filtration and the resulting solution was concentrated. The residue was diluted with ACN/water mixture. The resulting solution was purified by HPLC.

VI. General procedure 1: Nitrilation of N,N-dimethyl peptides to generate nitrile-peptide products.

To 1 mg (6-10mM) of unprotected N,N-dimethyl peptide dissolved in 300 µL of 10 mM sodium phosphate buffer (NaP, pH 7.0), was added sodium cyanide (3 equiv.) and selectfluor (2 equiv.). The reaction mixture was stirred for 1 h. Samples were taken from the reaction mixture, injected into LC-MS to monitor the % conversion of N,N-dimethyl peptides to the nitrile peptide products. The reaction mixture was analyzed by HPLC using **HPLC METHOD A**

VII. General procedure 2: Thiazolidination of nitrile modified N,N-dimethyl peptides to generate thiazolidine-peptide products.

To 1.0 mg (6-10mM) of N,N-dimethyl nitrile peptide dissolved in 600 µL of 10 mM sodium phosphate buffer (NaP, pH 7.0) and isopropyl alcohol (1:1), was added 3 equiv. of cysteine analog. The reaction mixture was stirred at 70 ^{O}C for 7 h. Sample was taken from the reaction

mixture, injected into LC-MS to monitor the generation of thiazolidine peptide products. The reaction mixture was analyzed by HPLC using **HPLC METHOD A** to determine the % conversion.

VIII. Supplementary Figure 1. Evaluation of oxidizing reagents**.**

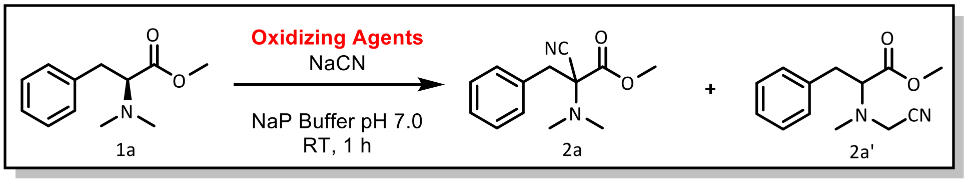

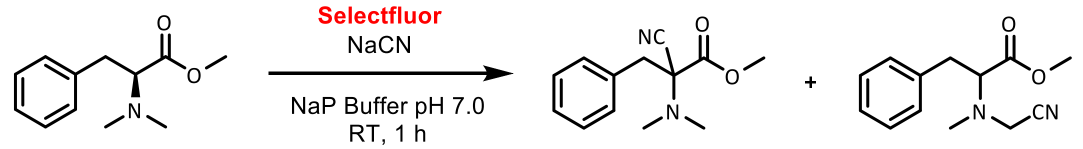

Exact Mass: 207.1259 | **Exact Mass: 232.1212** | **Exact Mass: 232.1212**

1a | 2a | 2a'

To 1.0 mg of N,N-dimethylphenylalanine methylester **1a** dissolved in 300 µL of 10 mM sodium phosphate buffer (NaP, pH 7.0), was added sodium cyanide (3 equiv.) and **selectfluor** (2 equiv.). The reaction mixture was stirred for 1 h. Sample was taken from the reaction mixture, injected into LC-MS to monitor the generation of N,N-dimethylphenylalanine methylester nitrile products **2a** and **2a'**. The reaction mixture was analyzed by HPLC using method A to determine the % conversion.

N,N-dimethylphenylalanine methylester 1a. LCMS: *m/z* 208.12903 (calcd [M+H]+ = 208.1259), Purity: >95 % (HPLC analysis at 220 nm). Retention time in HPLC: 5.280

N,N-dimethylphenylalanine methylester nitrile products 2a and 2a'. LCMS: *m/z* 233.12394 (calcd [M+H]+ = 232.1212), Purity: >95 % (HPLC analysis at 220 nm). Retention time in HPLC: 17.010

HPLC Trace of selectfluor as oxidizing reagent

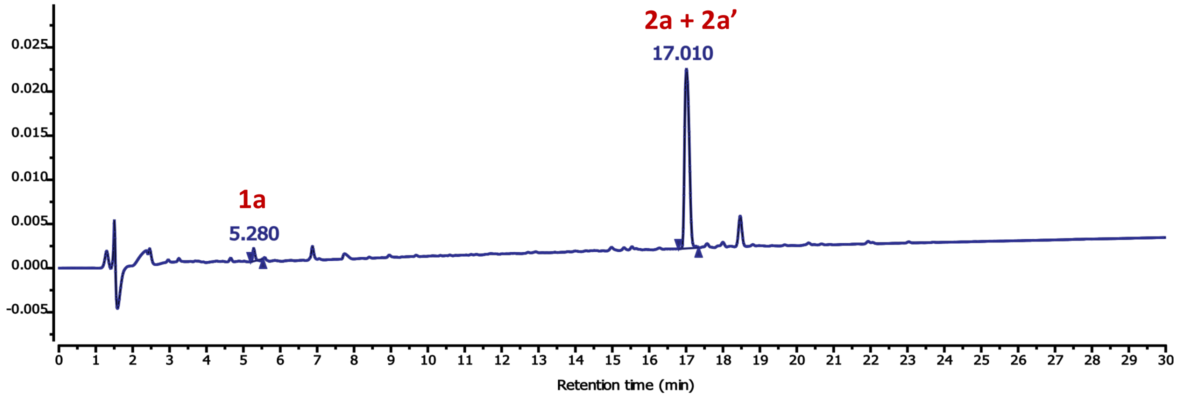

MS-Trace of 1a (peak 5.280)

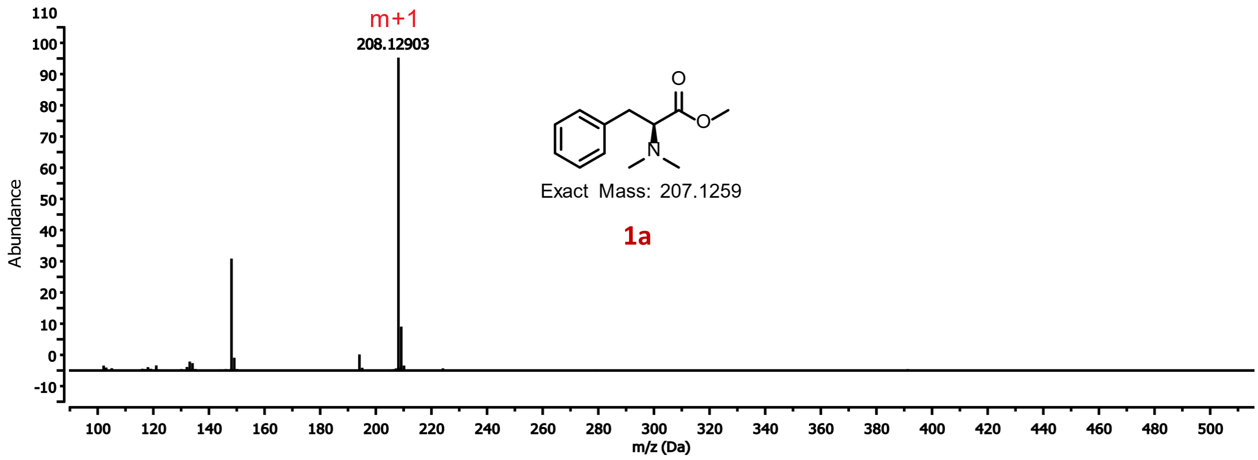

MS-Trace of 2a and 2a' (peak 17.010)

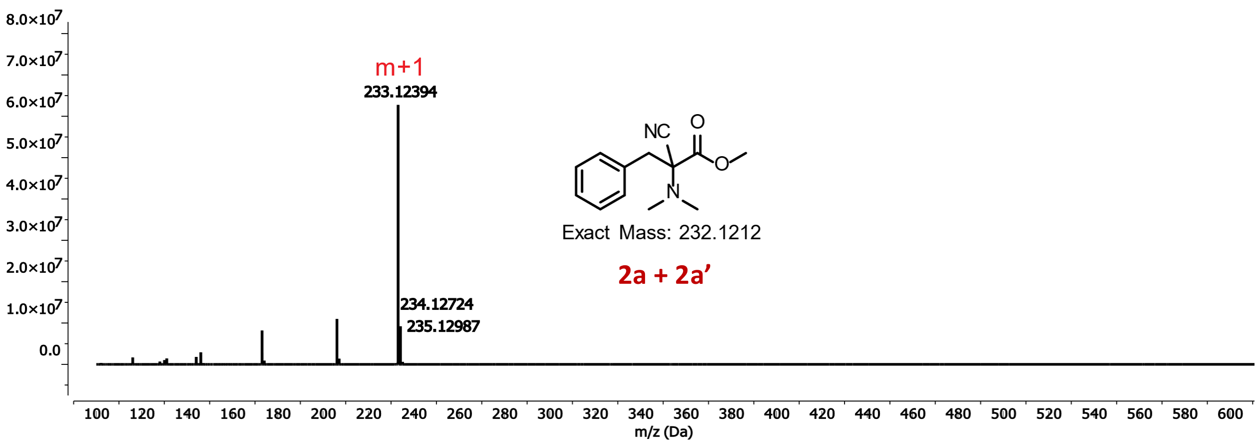

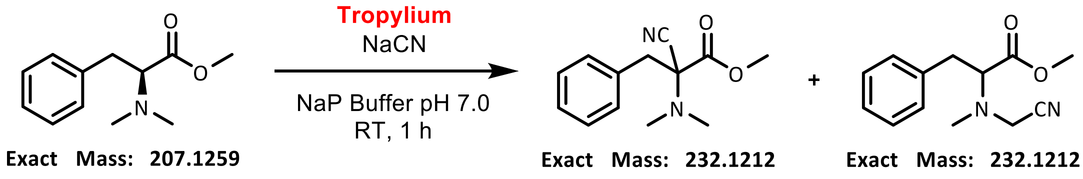

1a 2a 2a'

To 1.0 mg of N,N-dimethylphenylalanine methylester **1a** dissolved in 300 µL of 10 mM sodium phosphate buffer (NaP, pH 7.0), was added sodium cyanide (3 equiv.) and **tropylium tetrafluoroborate** (2 equiv.). The reaction mixture was stirred for 1 h. Sample was taken from the reaction mixture, injected into LC-MS to monitor the generation of N,N-dimethylphenylalanine methylester nitrile products **2a** and **2a'**. The reaction mixture was analyzed by HPLC using method A to determine the % conversion.

N,N-dimethylphenylalanine methylester 1a. LCMS: *m/z* 208.12903 (calcd [M+H]+ = 208.1259), Purity: >95 % (HPLC analysis at 220 nm). Retention time in HPLC: 4.844

HPLC Trace of tropylium as oxidizing reagent

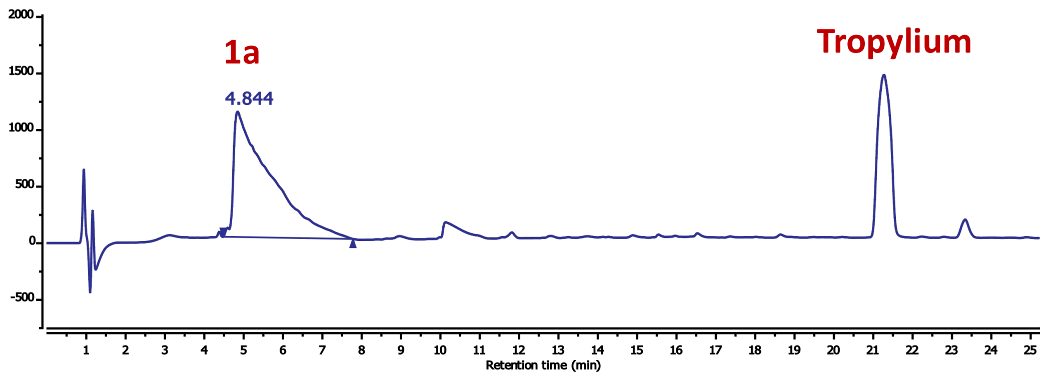

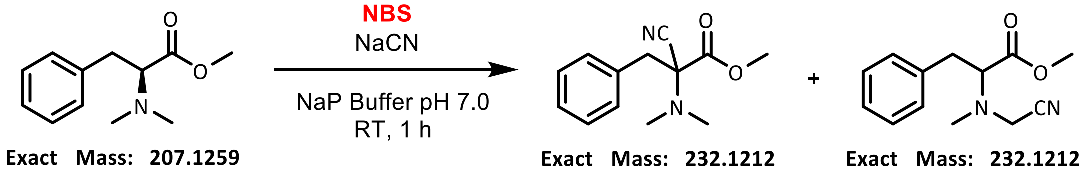

1a 2a 2a'

To 1.0 mg of N,N-dimethylphenylalanine methylester **1a** dissolved in 300 µL of 10 mM sodium phosphate buffer (NaP, pH 7.0), was added sodium cyanide (3 equiv.) and **N-bromosuccinimide** (2 equiv.). The reaction mixture was stirred for 1 h. Sample was taken from the reaction mixture, injected into LC-MS to monitor the generation of N,N-dimethylphenylalanine methylester nitrile products **2a** and **2a'**. The reaction mixture was analyzed by HPLC using method A to determine the % conversion.

N,N-dimethylphenylalanine methylester 1a. LCMS: *m/z* 208.12903 (calcd [M+H]+ = 208.1259), Purity: >95 % (HPLC analysis at 220 nm). Retention time in HPLC: 4.973

HPLC Trace of NBS as oxidizing reagent

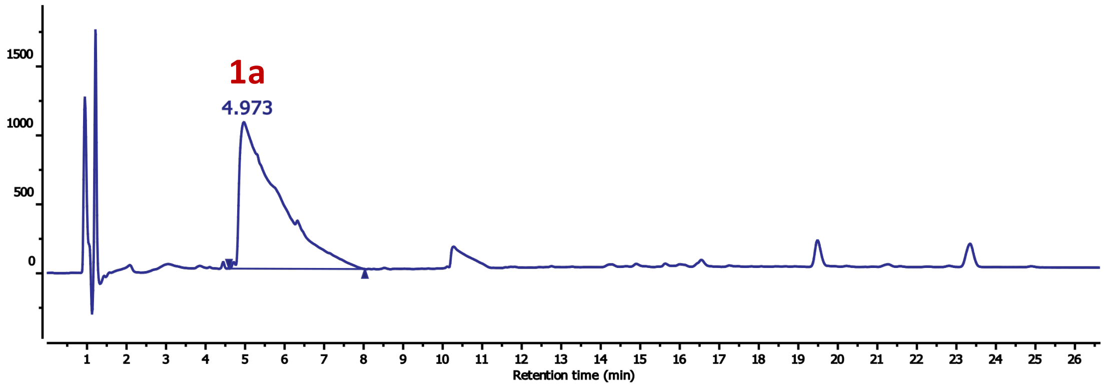

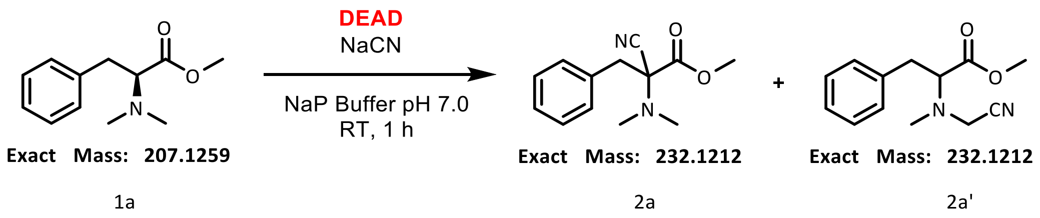

To 1.0 mg of N,N-dimethylphenylalanine methylester **1a** dissolved in 300 µL of 10 mM sodium phosphate buffer (NaP, pH 7.0), was added sodium cyanide (3 equiv.) and **diethyl azodicarboxylate** (2 equiv.). The reaction mixture was stirred for 1 h. Sample was taken from the reaction mixture, injected into LC-MS to monitor the generation of N,N-dimethylphenylalanine methylester nitrile products **2a** and **2a'**. The reaction mixture was analyzed by HPLC using method A to determine the % conversion.

N,N-dimethylphenylalanine methylester 1a. LCMS: *m/z* 208.12903 (calcd [M+H]+ = 208.1259), Purity: >95 % (HPLC analysis at 220 nm). Retention time in HPLC: 5.887

HPLC Trace of DEAD as oxidizing reagent

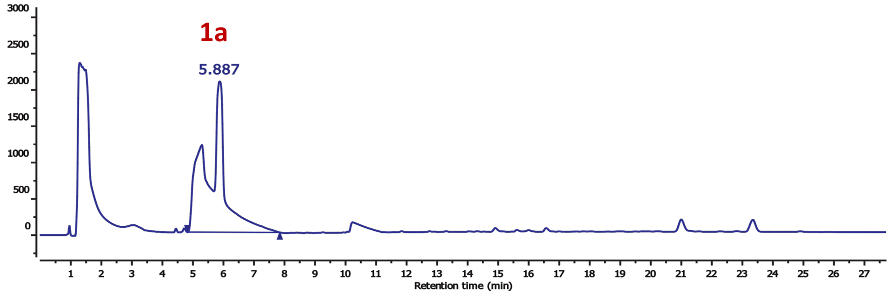

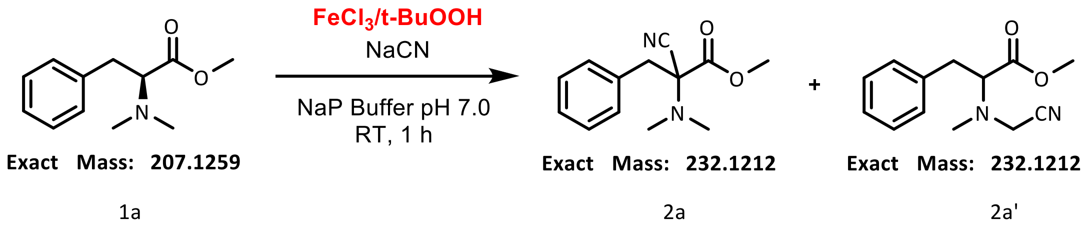

To 1.0 mg of N,N-dimethylphenylalanine methylester **1a** dissolved in 300 µL of 10 mM sodium phosphate buffer (NaP, pH 7.0), was added sodium cyanide (3 equiv.) and **$FeCl_3$/t-BuOOH** (2 equiv.). The reaction mixture was stirred for 1 h. Sample was taken from the reaction mixture, injected into LC-MS to monitor the generation of N,N-dimethylphenylalanine methylester nitrile products **2a** and **2a'**. The reaction mixture was analyzed by HPLC using method A to determine the % conversion.

N,N-dimethylphenylalanine methylester 1a. LCMS: *m/z* 208.12903 (calcd [M+H]+ = 208.1259), Purity: >95 % (HPLC analysis at 220 nm). Retention time in HPLC: 4.944

HPLC Trace of $FeCl_3$/t-BuOOH as oxidizing reagent

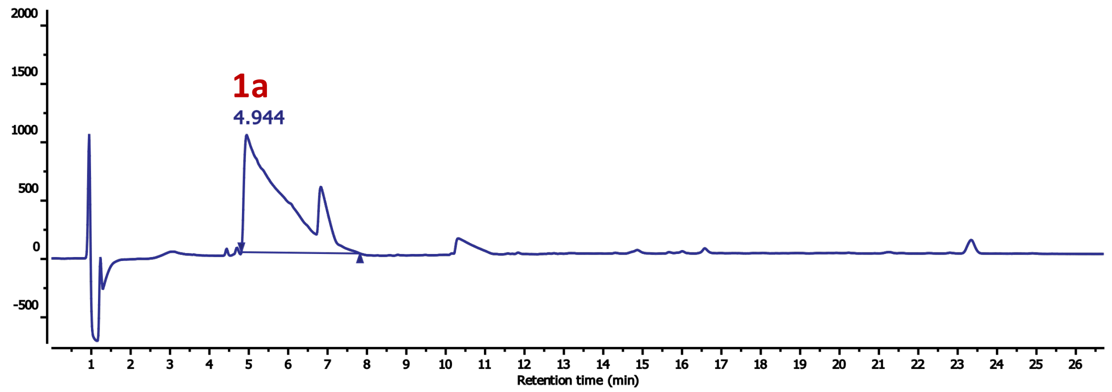

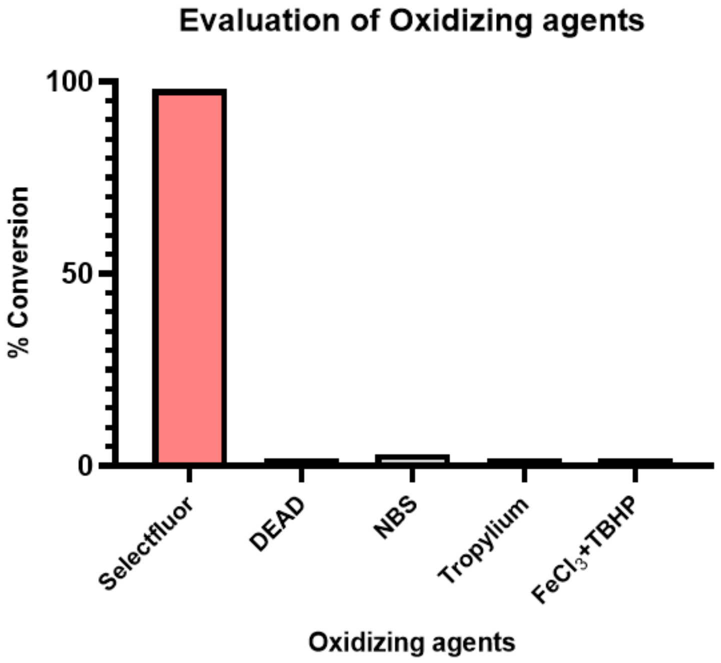

IX. Supplementary Figure 2. Characterization of N,N-dimethylphenylalanine methylester nitrile product **2a** and **2a'**.

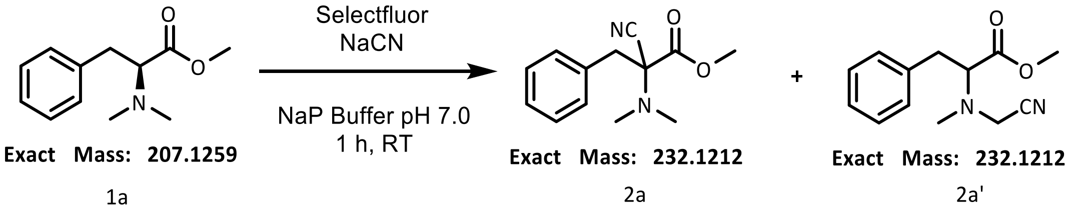

100 mg of N,N-dimethylphenylalanine methylester was modified using **general procedure 1**. Concentration of the mixture gave a residue, which was purified by silica gel column chromatography (eluent: 100% CH_2Cl_2) to give inseparable mixture of two nitrile products **2a** and **2a'** of N,N-dimethylphenylalanine methylester. NMR Yield (80% **2a**, 20% **2a'**)

^{1}H NMR of N,N-dimethyl phenylalanine methyl ester nitrile product 2a (600 MHz, $CDCl_3$) δ 7.35 – 7.30 (m, 3H), 7.30 – 7.27 (m, 2H), 3.56 (s, 3H), 3.37 (d, *J* = 12.8 Hz, 1H), 3.17 (d, *J* = 12.8 Hz, 1H), 2.44 (s, 6H).

^{1}H NMR of N,N-dimethyl phenylalanine methyl ester nitrile product 2a' (600 MHz, $CDCl_3$) δ 7.28 – 7.20 (m, 3H), 7.22 – 7.17 (m, 2H), 3.73 (t, *J* = 4.4 Hz, 1H), 3.68 (d, *J* = 5.9 Hz, 2H), 3.65 (s, 3H), 3.06 (dd, *J* = 13.7, 8.6 Hz, 1H), 2.97 (dd, *J* = 13.6, 6.7 Hz, 1H), 2.53 (s, 3H).

^{13}C NMR of N,N-dimethyl phenylalanine methyl ester nitrile product 2a (151 MHz, $CDCl_3$) δ 167.26, 132.81, 129.96, 128.67, 128.12, 114.51, 74.17, 53.19, 43.01, 40.94.

^{13}C NMR of N,N-dimethyl phenylalanine methyl ester nitrile product 2a' (151 MHz, $CDCl_3$) δ 171.23, 136.95, 129.02, 128.55, 126.83, 115.67, 67.56, 51.55, 42.80, 38.70, 36.02.

^{1}H NMR of N,N-dimethylphenylalanine methylester nitrile product 2a

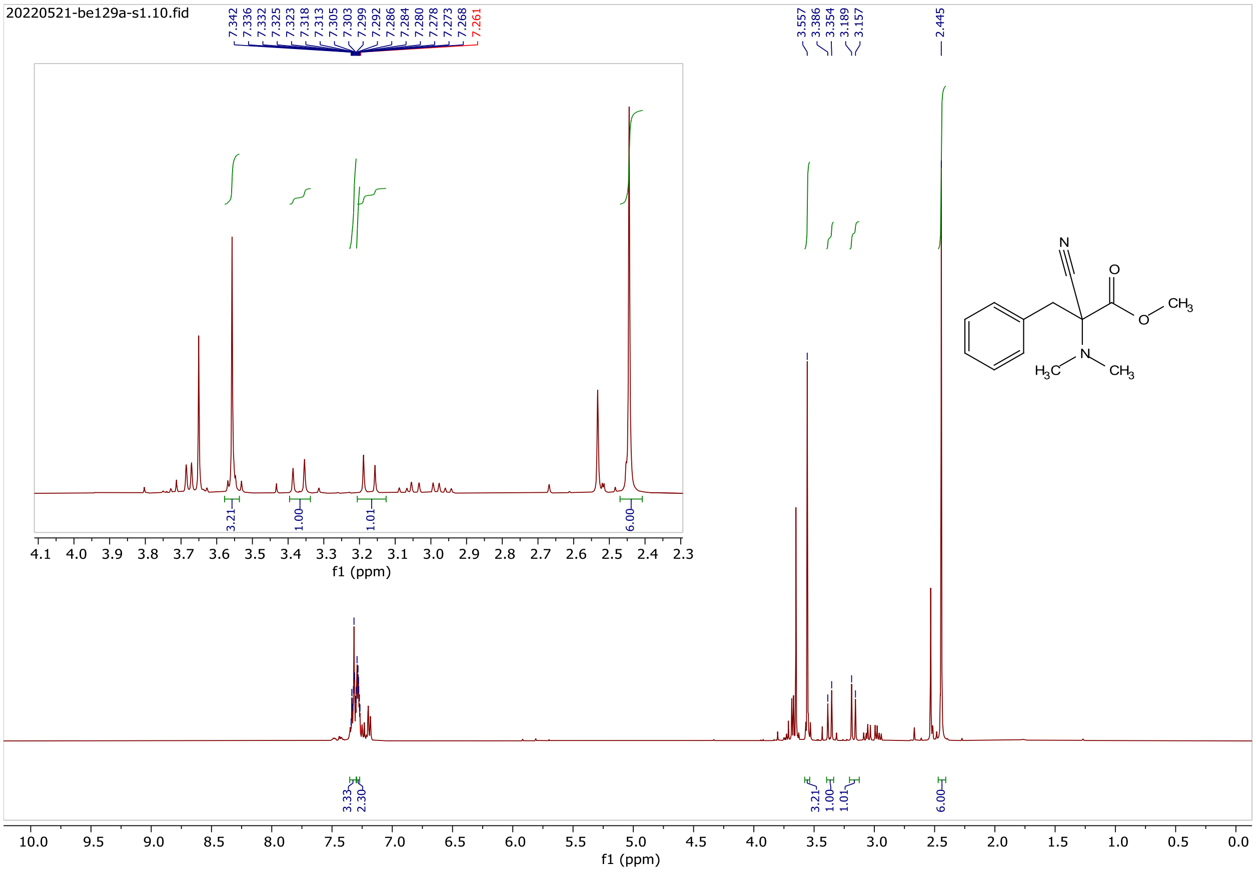

^{1}H NMR of N,N-dimethylphenylalanine methylester nitrile product 2a'

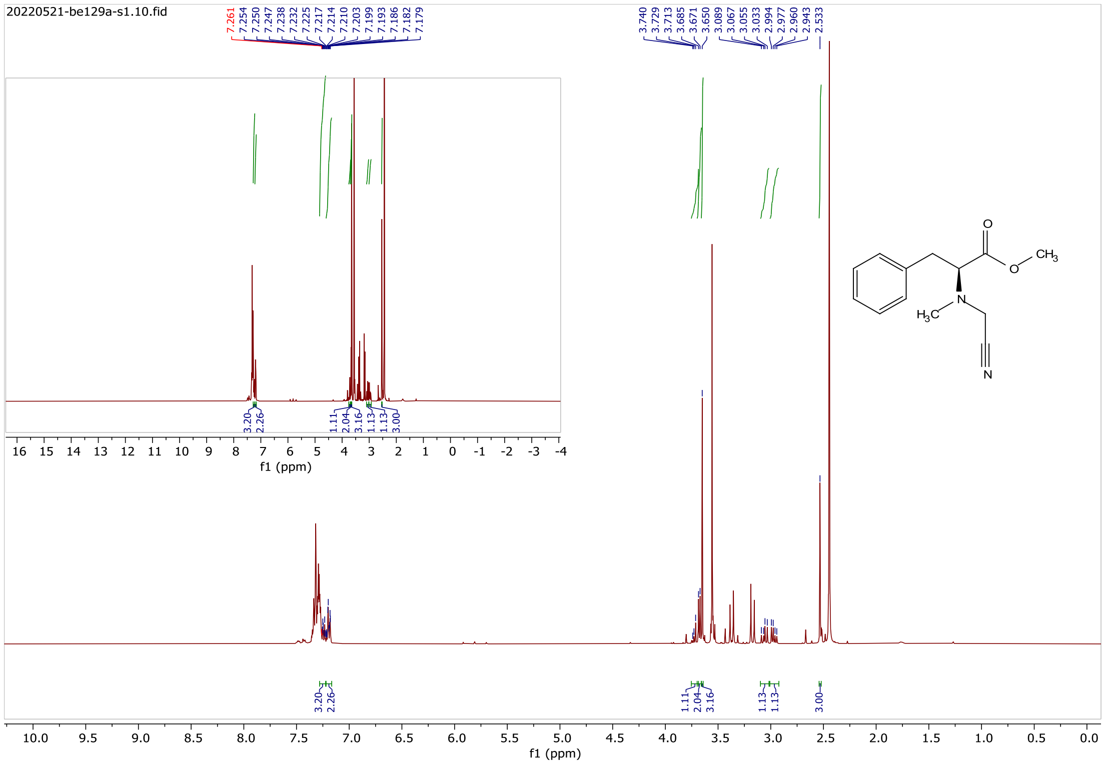

^{13}C NMR of N,N-dimethyl phenylalanine methyl ester nitrile products 2a and 2a'

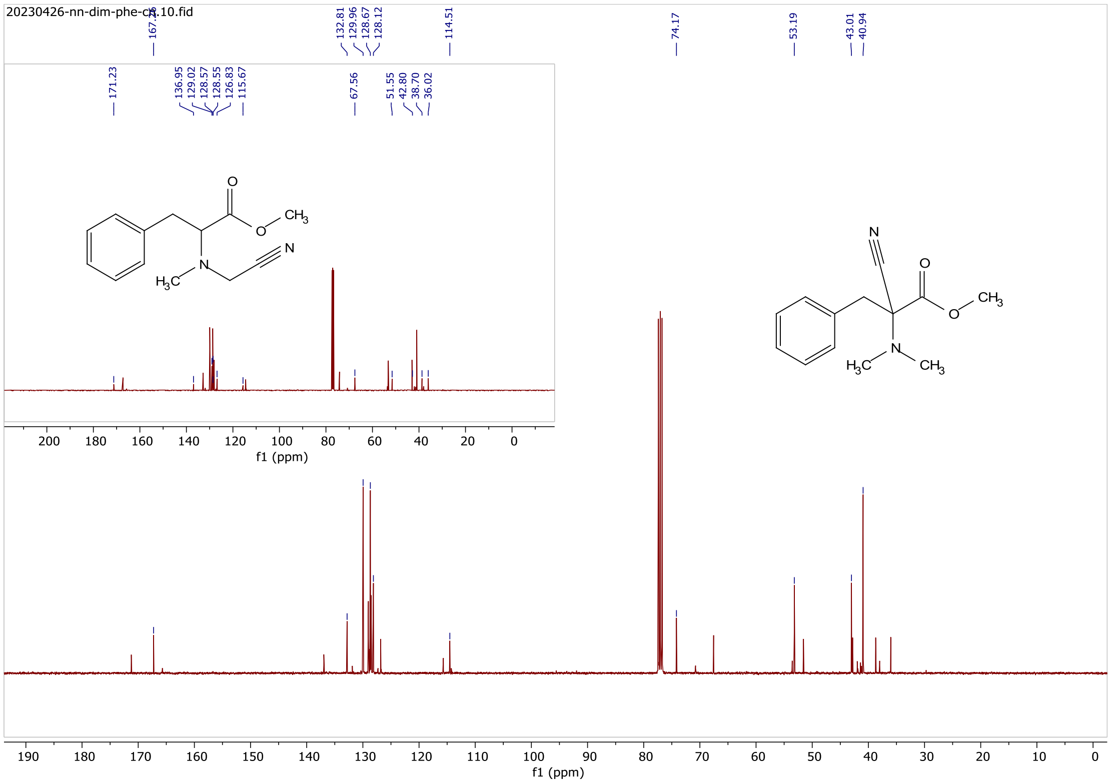

X. Supplementary Figure 3. Evaluation of nucleophiles.

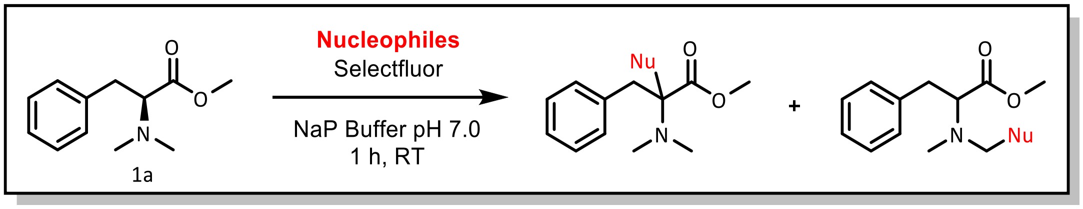

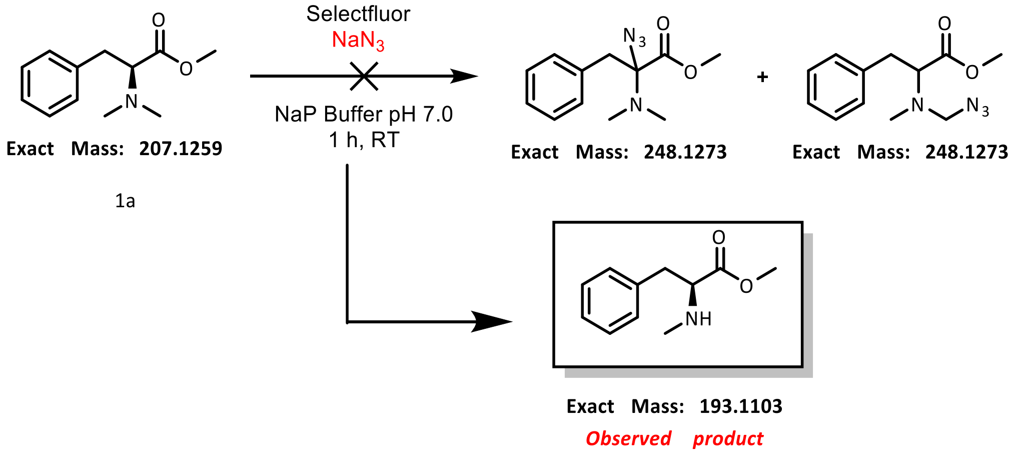

To 1.0 mg of N,N-dimethylphenylalanine methylester **1a** dissolved in 300 µL of 10 mM sodium phosphate buffer (NaP, pH 7.0), was added **sodium azide** (3 equiv.) and selectfluor (2 equiv.). The reaction mixture was stirred for 1 h. Sample was taken from the reaction mixture, injected into LC-MS to monitor the generation of N,N-dimethylphenylalanine methylester azide product. The reaction mixture was analyzed by HPLC using method A and no azide labeled product was observed under the reaction conditions. We observed the demethylation and formation of N-methyl-phenylalanine methyl ester product (>98 %).

N-methyl-phenylalanine methylester**.** LCMS: m/z 194.11377 (calcd [M+H]+ = 194.1174), Purity: >95 % (HPLC analysis at 220 nm). Retention time in HPLC: 6.053

HPLC Trace of sodium azide as a nucleophile

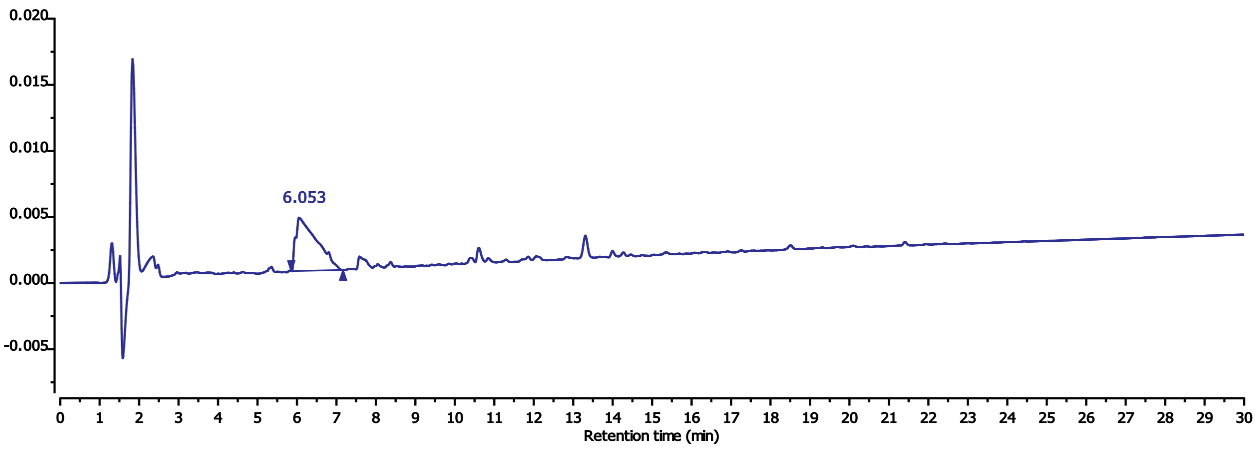

Mass spectra of peak 6.053

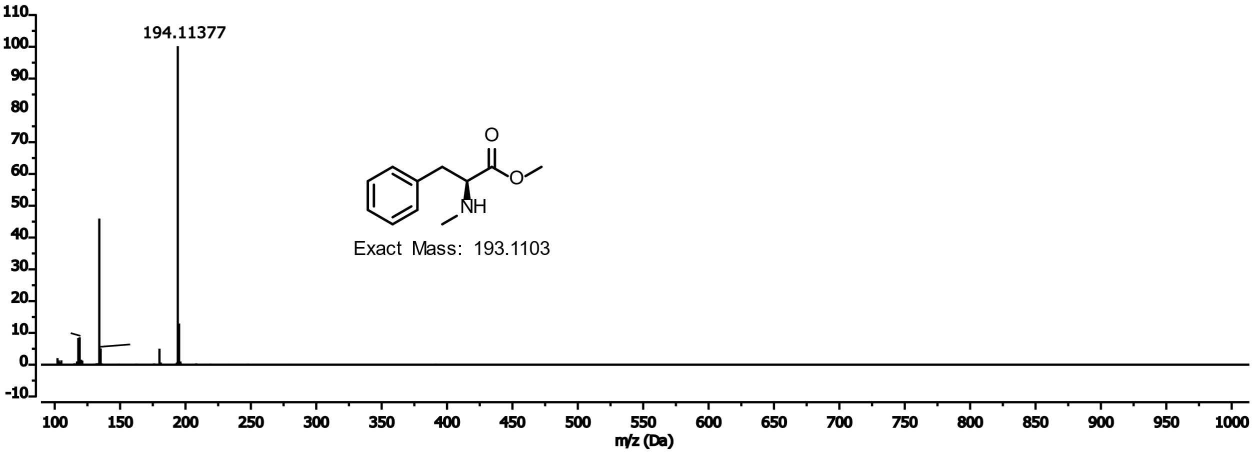

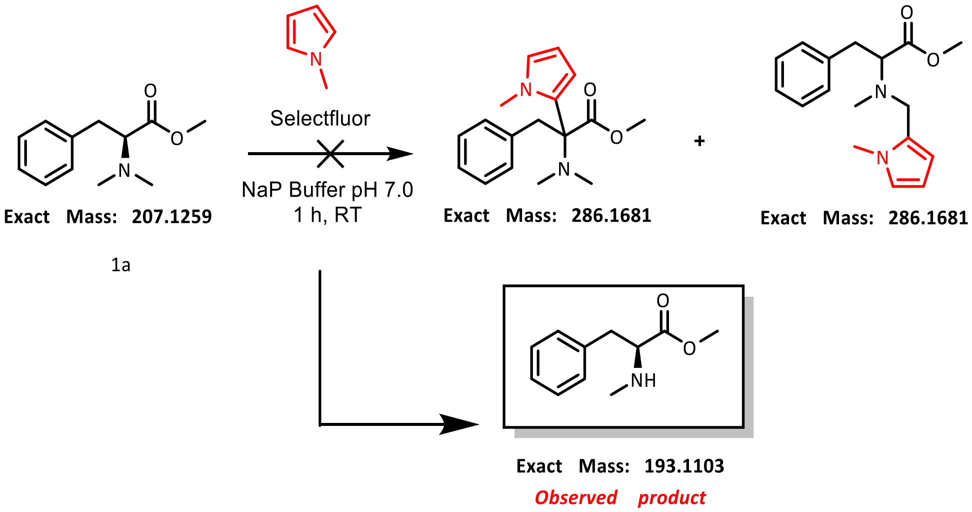

To 1.0 mg of N,N-dimethylphenylalanine methylester **1a** dissolved in 300 μL of 10 mM sodium phosphate buffer (NaP, pH 7.0), was added **N-methylpyrrole** (3 equiv.) and selectfluor (2 equiv.). The reaction mixture was stirred for 1 h. Sample was taken from the reaction mixture, injected into LC-MS to monitor the generation of N,N-dimethylphenylalanine methylester N-methylpyrrole labeled product. The reaction mixture was analyzed by HPLC using method A and no N-methylpyrrole labeled product was observed under the reaction conditions. We observed the demethylation and formation of N-methyl-phenylalanine methyl ester product (27 %).

N,N-dimethylphenylalanine methylester **1a.** LCMS: *m/z* 208.12903 (calcd [M+H]+ = 208.1259), Purity: >95 % (HPLC analysis at 220 nm). Retention time in HPLC: 6.753

N-methyl-phenylalanine methylester**.** LCMS: m/z 194.11377 (calcd [M+H]+ = 194.1174), Purity: >95 % (HPLC analysis at 220 nm). Retention time in HPLC: 6.137

HPLC Trace of N-Methylpyrrole as a nucleophile

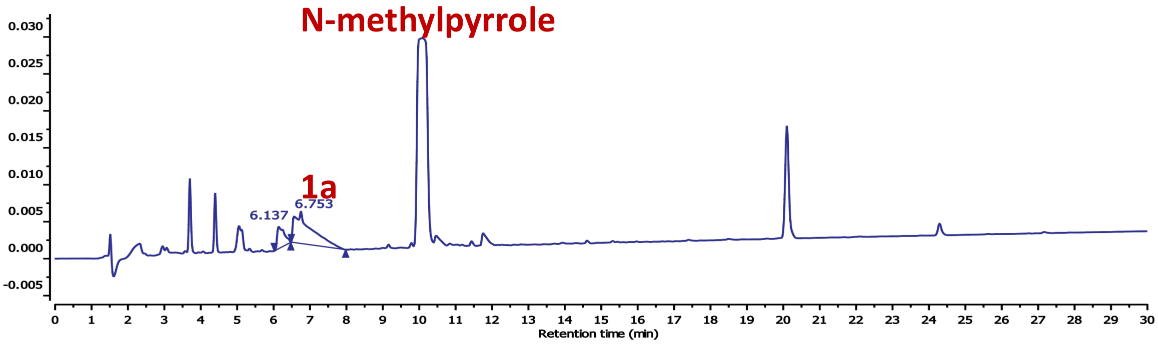

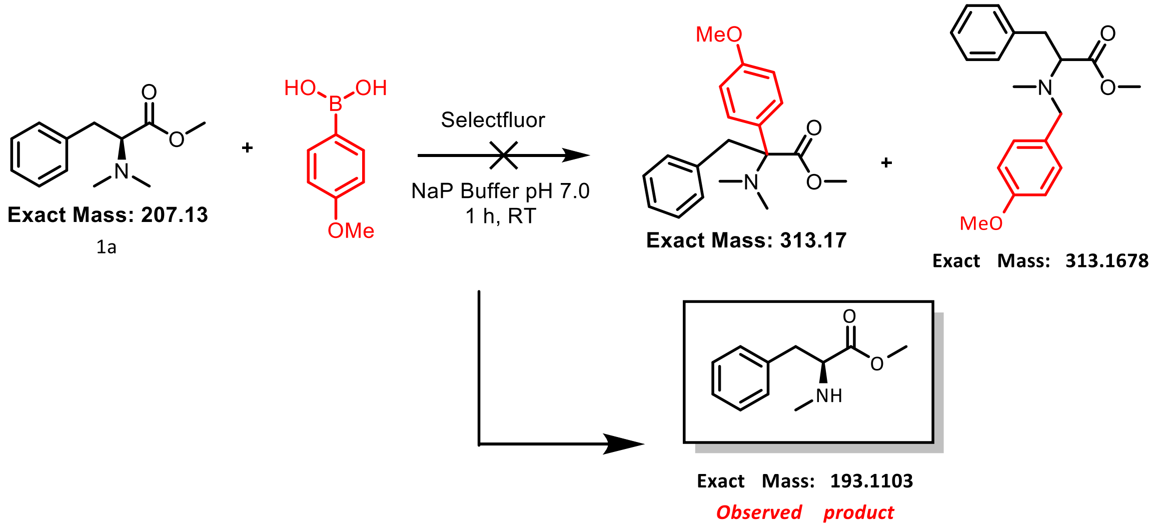

To 1.0 mg of N,N-dimethylphenylalanine methylester **1a** dissolved in 300 μL of 10 mM sodium phosphate buffer (NaP, pH 7.0), was added ***p*-methoxyphenylboronic acid** (3 equiv.) and selectfluor (2 equiv.). The reaction mixture was stirred for 1 h. Sample was taken from the reaction mixture, injected into LC-MS to monitor the generation of N,N-dimethylphenylalanine methylester *p*-methoxyphenyl labeled product. The reaction mixture was analyzed by HPLC using method A and no p-methoxyphenyl labeled product was observed under the reaction conditions. We observed the demethylation and formation of N-methyl-phenylalanine methyl ester products (44 %).

N,N-dimethylphenylalanine methylester **1a**. LCMS: *m/z* 208.12903 (calcd [M+H]+ = 208.1259), Purity: >95 % (HPLC analysis at 220 nm). Retention time in HPLC: 6.863

N-methyl-phenylalanine methylester**.** LCMS: m/z 194.11377 (calcd [M+H]+ = 194.1174), Purity: >95 % (HPLC analysis at 220 nm). Retention time in HPLC: 6.133

HPLC Trace of *p*-methoxyboronic acid as a nucleophile

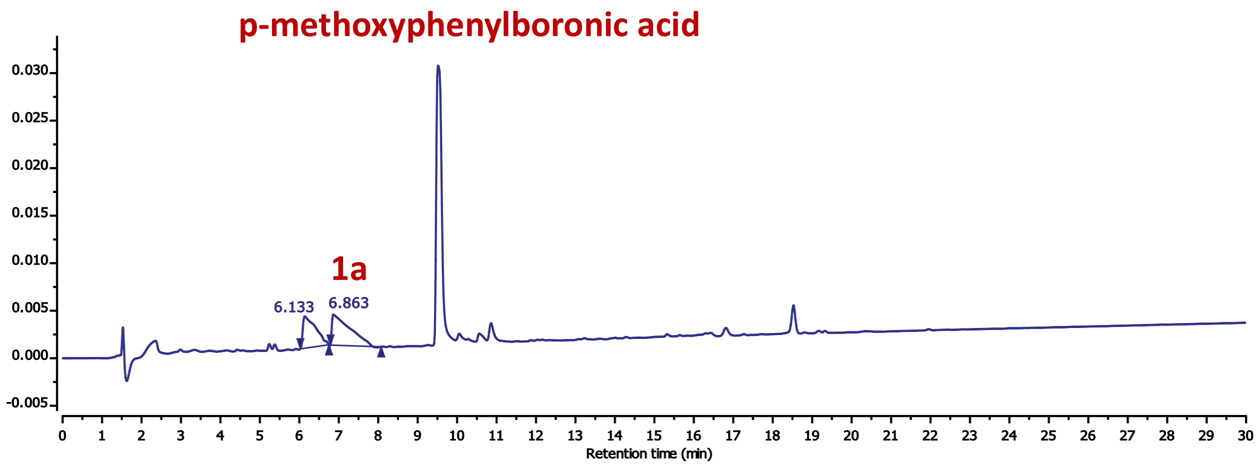

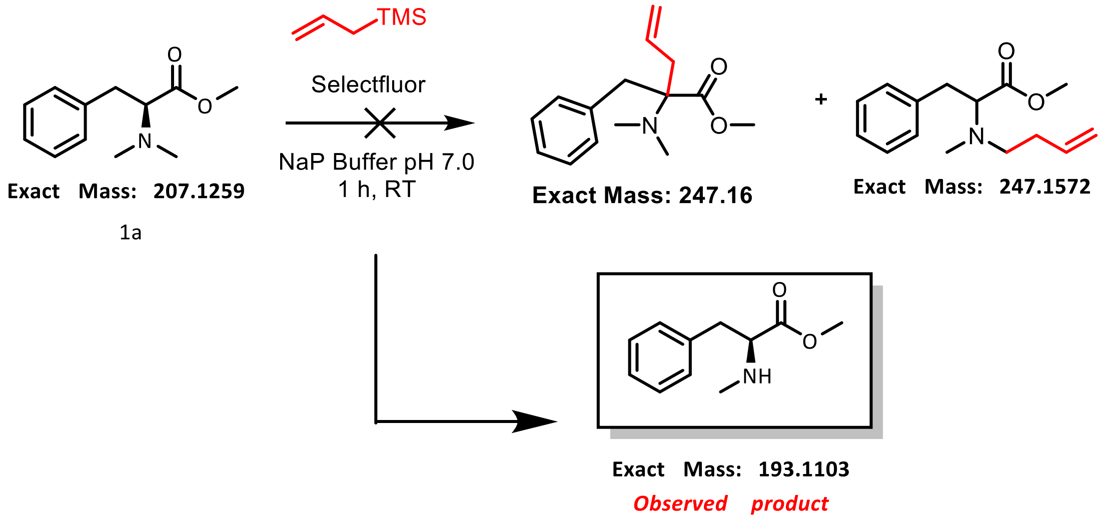

To 1.0 mg of N,N-dimethylphenylalanine methylester **1a** dissolved in 300 μL of 10 mM sodium phosphate buffer (NaP, pH 7.0), was added **allyltrimethylsilane** (3 equiv.) and selectfluor (2 equiv.). The reaction mixture was stirred for 1 h. Sample was taken from the reaction mixture, injected into LC-MS to monitor the generation of N,N-dimethylphenylalanine methylester allyl labeled product. The reaction mixture was analyzed by HPLC using method A and no allyl labeled product was observed under the reaction conditions. We observed the demethylation and formation of N-methyl-phenylalanine methyl ester product (47 %).

N,N-dimethylphenylalanine methylester **1a**. LCMS: *m/z* 208.12903 (calcd [M+H]+ = 208.1259), Purity: >95 % (HPLC analysis at 220 nm). Retention time in HPLC: 7.287

N-methyl-phenylalanine methylester**.** LCMS: m/z 194.11377 (calcd [M+H]+ = 194.1174), Purity: >95 % (HPLC analysis at 220 nm). Retention time in HPLC: 6.593

HPLC Trace of Potassium allyltrifluoroborate as a nucleophile

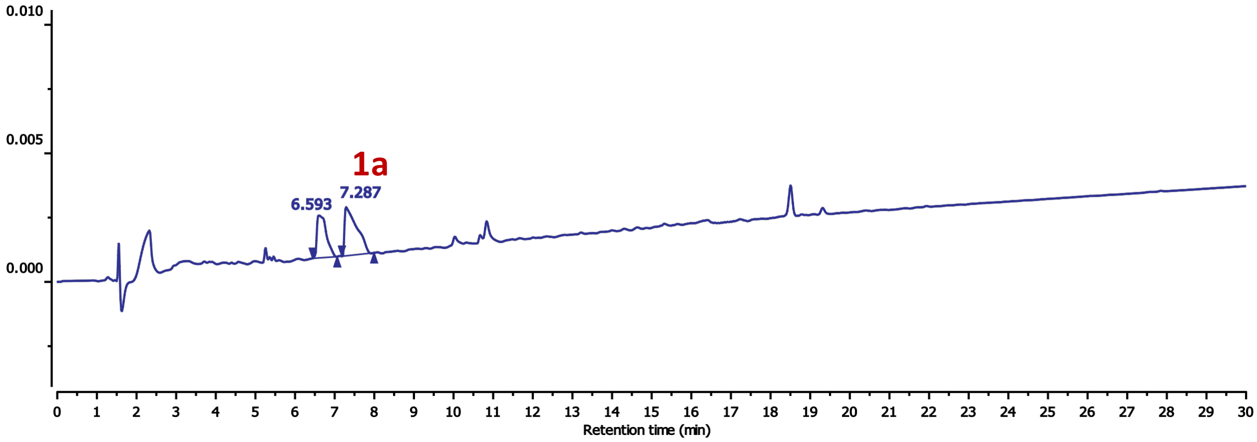

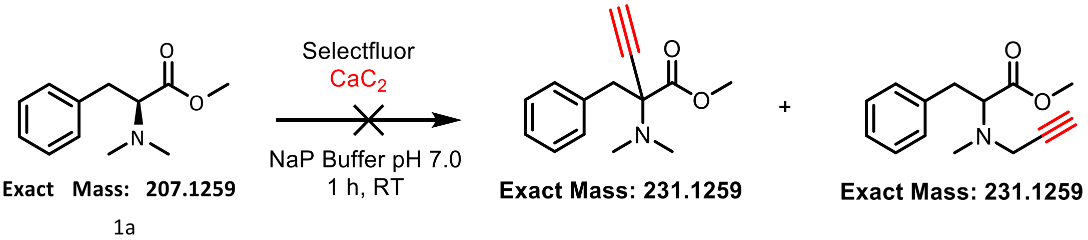

To 1.0 mg of N,N-dimethylphenylalanine methylester **1a** dissolved in 300 µL of 10 mM sodium phosphate buffer (NaP, pH 7.0), was added **calcium carbide** (3 equiv.) and selectfluor (2 equiv.). The reaction mixture was stirred for 1 h. Sample was taken from the reaction mixture, injected into LC-MS to monitor the generation of N,N-dimethylphenylalanine methylester acetylene labeled product. The reaction mixture was analyzed by HPLC using method A and no acetylene labeled product was observed under the reaction conditions.

N,N-dimethylphenylalanine methylester **1a**. LCMS: *m/z* 208.12903 (calcd [M+H]+ = 208.1259), Purity: >95 % (HPLC analysis at 220 nm). Retention time in HPLC: 6.557

HPLC Trace of calcium carbide as a nucleophile

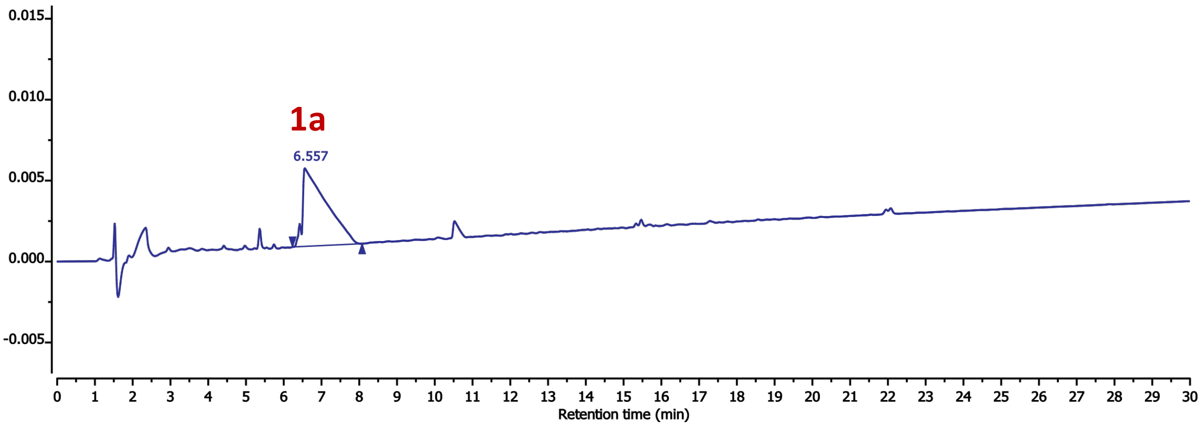

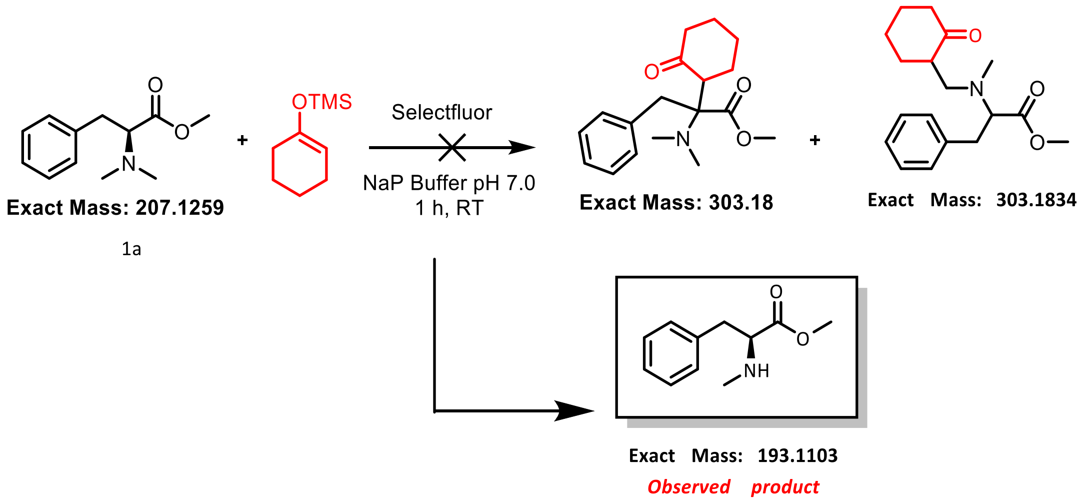

To 1.0 mg of N,N-dimethylphenylalanine methylester **1a** dissolved in 300 µL of 10 mM sodium phosphate buffer (NaP, pH 7.0), was added **1-(Trimethylsiloxy)cyclohexene** (3 equiv.) and selectfluor (2 equiv.). The reaction mixture was stirred for 1 h. Sample was taken from the reaction mixture, injected into LC-MS to monitor the generation of N,N-dimethylphenylalanine methylester cyclohexanone labeled product. The reaction mixture was analyzed by HPLC using method A and no cyclohexanone labeled product was observed under the reaction conditions. We observed the demethylation and formation of N-methyl-phenylalanine methylester product (51 %).

N,N-dimethylphenylalanine methylester **1a.** LCMS: *m/z* 208.12903 (calcd [M+H]+ = 208.1259), Purity: >95 % (HPLC analysis at 220 nm). Retention time in HPLC: 7.170

N-methyl-phenylalanine methylester**.** LCMS: m/z 194.11377 (calcd [M+H]+ = 194.1174), Purity: >95 % (HPLC analysis at 220 nm). Retention time in HPLC: 6.707

HPLC Trace of 1-(Trimethylsiloxy)cyclohexene as a nucleophile

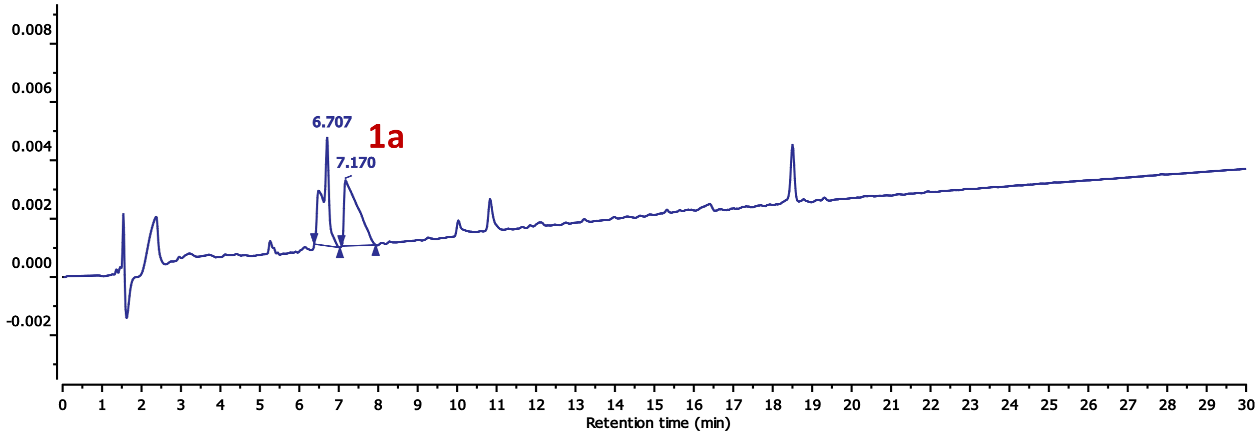

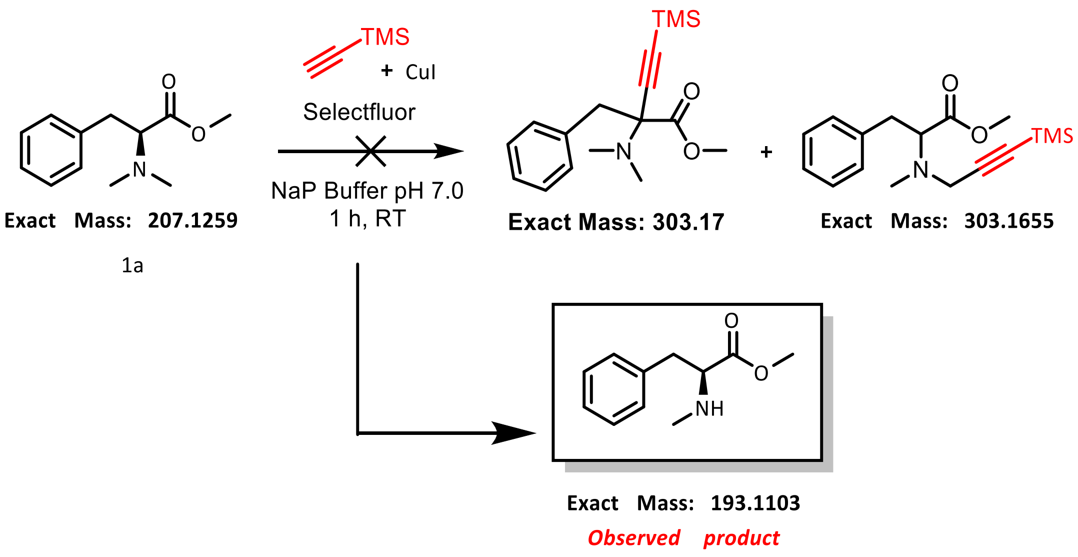

To 1.0 mg of N,N-dimethylphenylalanine methylester **1a** dissolved in 300 µL of 10 mM sodium phosphate buffer (NaP, pH 7.0), was added **trimethylsilylacetylene** (3 equiv.), 10 mol% copper iodide, and selectfluor (2 equiv.). The reaction mixture was stirred for 1 h. Sample was taken from the reaction mixture, injected into LC-MS to monitor the generation of N,N-dimethylphenylalanine methylester trimethylsilylacetylene labeled product. The reaction mixture was analyzed by HPLC using method A and no trimethylsilylacetylene labeled product was observed under the reaction conditions. We observed the demethylation and formation of N-methyl-phenylalanine methylester (53 %).

N,N-dimethylphenylalanine methylester **1a.** LCMS: *m/z* 208.12903 (calcd [M+H]+ = 208.1259), Purity: >95 % (HPLC analysis at 220 nm). Retention time in HPLC: 7.017

N-methyl-phenylalanine methylester**.** LCMS: m/z 194.11377 (calcd [M+H]+ = 194.1174), Purity: >95 % (HPLC analysis at 220 nm). Retention time in HPLC: 6.153

HPLC Trace of Trimethylsilylacetylene as a nucleophile

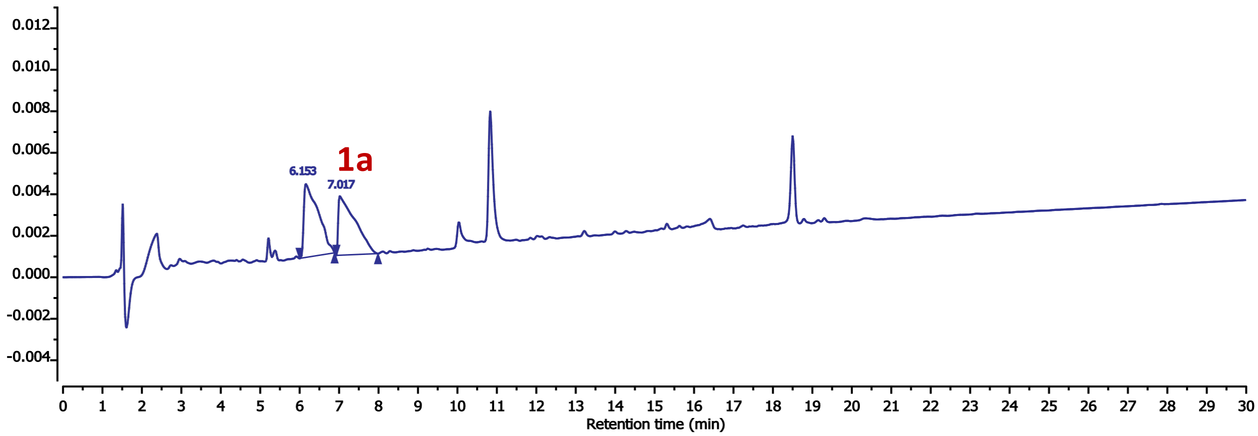

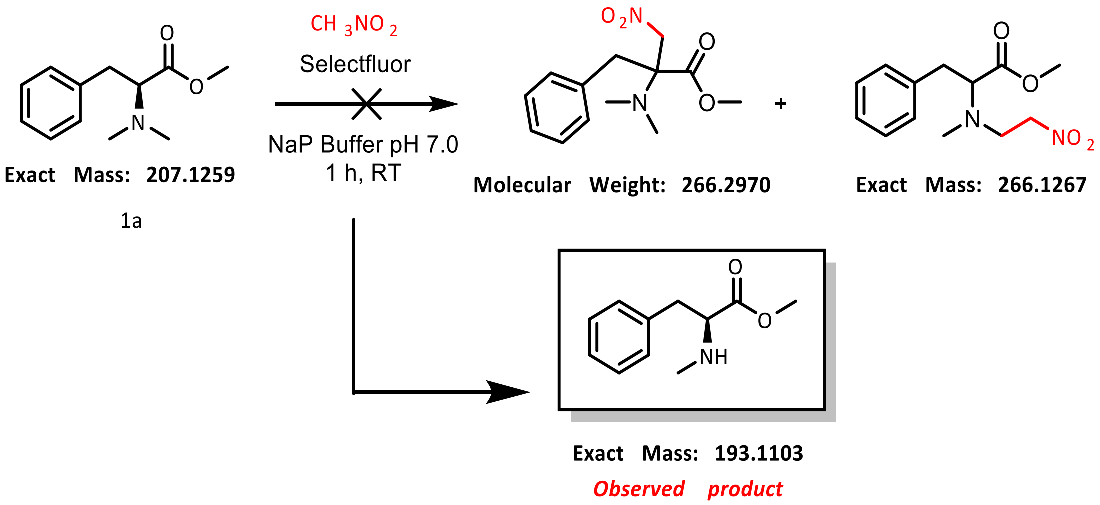

To 1.0 mg of N,N-dimethylphenylalanine methylester **1a** dissolved in 300 µL of 10 mM sodium phosphate buffer (NaP, pH 7.0), was added **nitromethane** (3 equiv.), and selectfluor (2 equiv.). The reaction mixture was stirred for 1 h. Sample was taken from the reaction mixture, injected into LC-MS to monitor the generation of N,N-dimethylphenylalanine methylester nitromethane labeled product. The reaction mixture was analyzed by HPLC using method A and no nitromethane labeled product was observed under the reaction conditions. We observed the demethylation and formation of N-methyl-phenylalanine methylester (74 %).

N,N-dimethylphenylalanine methylester **1a**. LCMS: *m/z* 208.12903 (calcd [M+H]+ = 208.1259), Purity: >95 % (HPLC analysis at 220 nm). Retention time in HPLC: 7.776

N-methyl-phenylalanine methylester**.** LCMS: m/z 194.11377 (calcd [M+H]+ = 194.1174), Purity: >95 % (HPLC analysis at 220 nm). Retention time in HPLC: 6.925

HPLC Trace of nitromethane as a nucleophile

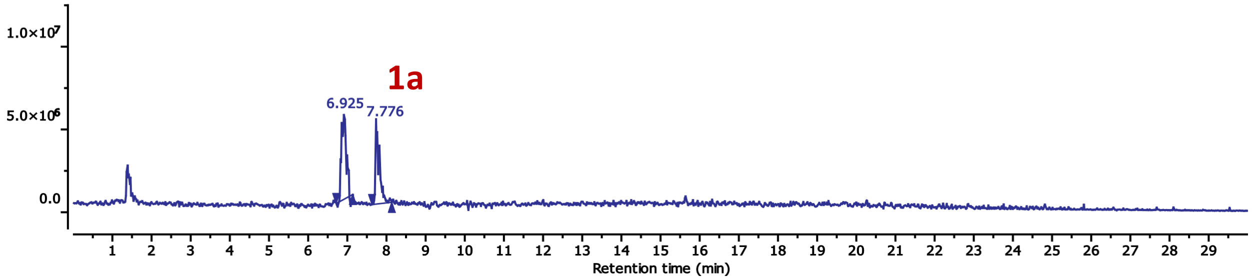

XI. Supplementary Figure 4. Computational evaluation of reaction regioselectivity.

pKa evaluation of α-C-H and N-methyl C-H protons:

DFT-based pKa calculation was performed using the macro-pka workflow in Schrodinger computational suite. Geometry optimization and macro-pKa calculations were done with the B3LYP/6-31G* level of theory. We observed a pKa value of **18.33** for the α-C-H bond and pKa of **20.05** for the N-methyl C-H bonds.

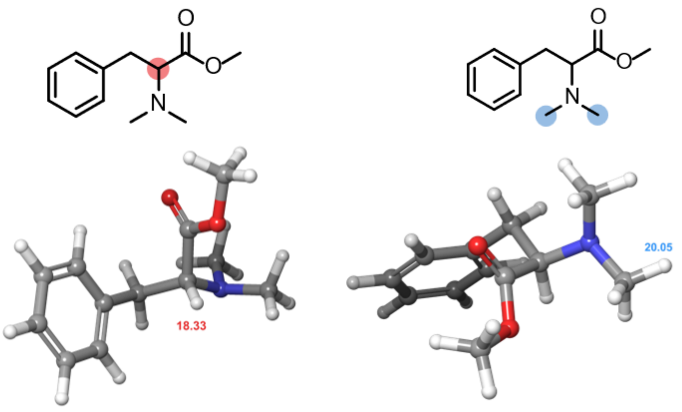

Bond dissociation energy evaluation of α-C-H and N-methyl C-H bonds.

Extensive DFT calculations of the bond dissociation energies was evaluated at various levels of theory (PBE 6-31G, B3LYP 3-21G, B3LYP 6-31G, B3LYP*++DP, B3LYP LACVP3P**, MO6 LACV3P**). Optimizations and BDE calculations at the different levels of theory consistently led to a lower BDE for the **α-C-H** bond.

	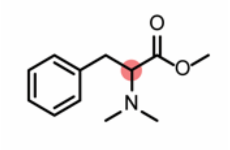	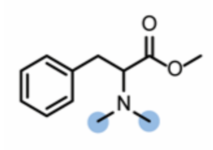	Difference
PBE0 6-31G	77.4 Kcal/mol	96.8 Kcal/mol	19.4 Kcal/mol
DFT(B3LYP) 3-21G	78.4 Kcal/mol	100.7 Kcal/mol	22.3 Kcal/mol
DFT(B3LYP) 6-31G	80.4 Kcal/mol	97.5 Kcal/mol	17.1 Kcal/mol
DFT(B3LYP) 6-31G*++DP	80.1 Kcal/mol	98.1 Kcal/mol	17.9 Kcal/mol
DFT(B3LYP) LACV3P**	84.5 Kcal/mol	97.5 Kcal/mol	13.0 Kcal/mol
DFT(MO6) LACV3P**	86.5 Kcal/mol	100.0 Kcal/mol	13.5 Kcal/mol

Thermodynamic and enthalpic calculations of α-C-H and N-methyl C-H iminium intermediates.

Extensive DFT calculations of the thermodynamic properties of the iminium intermediates of **α-C-H** and **N-methyl C-H** were evaluated at various levels of theory (B3LYP 6-31G, B3LYP 6-31G**, B3LYP-D3 6-31G**, B3LYP-D3 LACVP3P**, MO6-2X LACV3P**). Optimizations and energy calculations at the different levels of theory consistently led to a lower energetics for the α-C-H bond iminium ion, thus indicating the stability of the α-C-H over the N-methyl iminium ions.

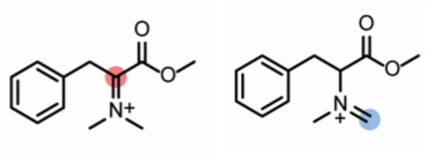

	$-\Delta G_{\alpha}$ Hartree	$-\Delta G_{N\text{-}methyl}$ Hartree	$-\Delta G_{N\text{-}methyl} - \Delta G_{\alpha}$ Kcal/mol
DFT(B3LYP) 6-31G	632.258211	632.251047	4.49
DFT(B3LYP) 6-31G**	671.972956	671.968092	3.05
DFT(B3LYP-D3) 6-31G**	672.001805	671.998142	4.55
DFT(B3LYP-D3BJ) 6-31G	671.802919	671.802938	3.98
DFT(B3LYP-D3) LACVP**	671.903609	671.903609	3.85
DFT(MO6-2X) LACV3P**	671.585789	671.580412	1.96

Evaluation of reaction energetics and identification of transition state

Prediction and optimization of pre-reaction complex (C1 and C2), transition state, and intermediates (C3 and C4) was calculated at the (B3LYP-D3BJ) 6-31G+DP level of theory. Solvent effects were also calculated using the polarizable continuum model (PCM). Intrinsic reaction coordinate (IRC) of the identified transition state was calculated at the (B3LYP-D3BJ) 6-31G+DP level of theory.

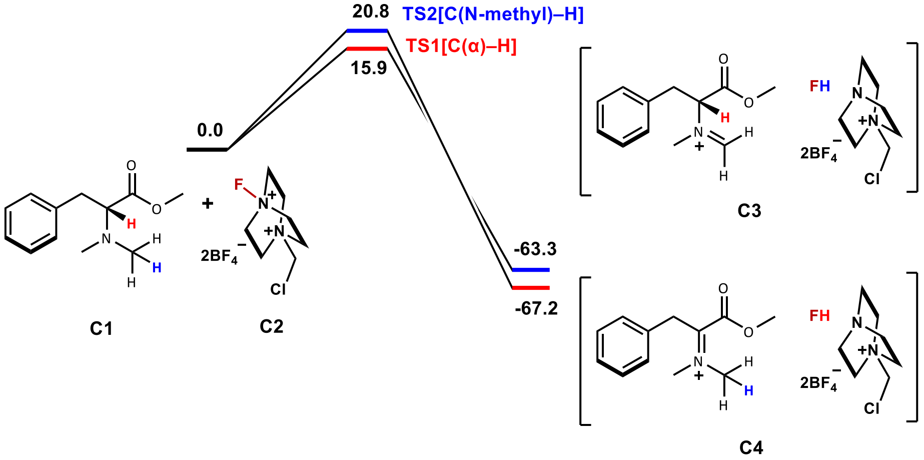

Selected structural and energetic parameters for the pre-reaction complex, transition state, and iminium ions of α-CH oxidation.

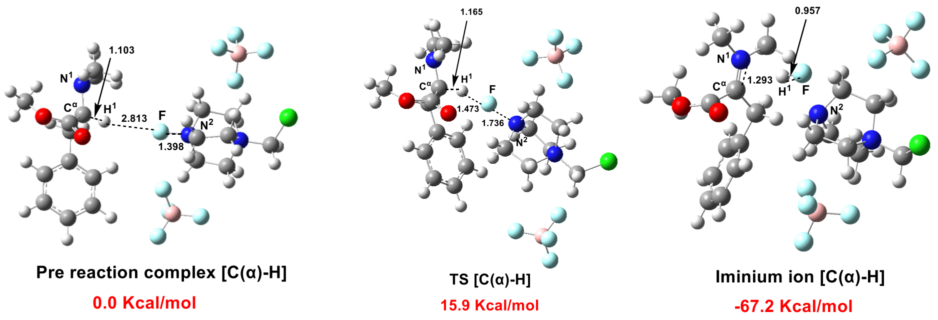

Selected structural and energetic parameters for the pre-reaction complex, transition state, and iminium ions of N-methyl-CH oxidation.

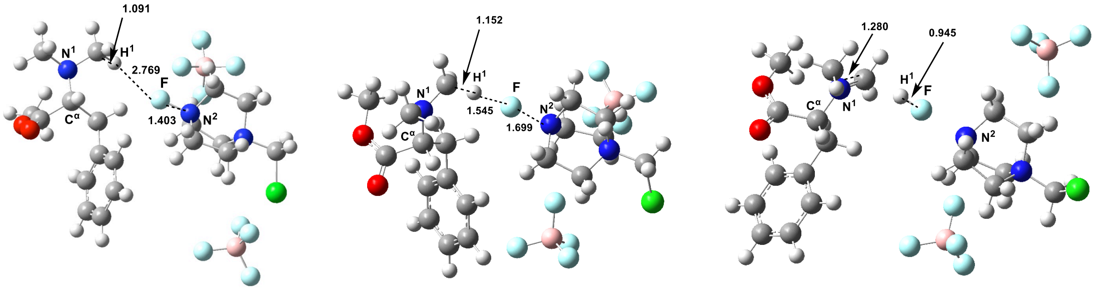

Pre reaction complex [C(N-methyl)-H] 0.0 Kcal/mol

TS [C(N-methyl)-H] 20.8 Kcal/mol

Iminium ion [C(N-methyl)-H] 63.3 Kcal/mol

Mechanistic probing of plausible radical mechanism for selectfluor oxidation of N-terminal dimethylation: To evaluate the possibility of a radical-mediated oxidation, we added stoichiometric amount of radical scavengers such as TEMPO, ABNO, and BHT to the optimized nitriliation reaction conditions. No radical coupling product was observed.

Radical-scavenging experiments with TEMPO: To 1.0 mg of N,N-dimethylphenylalanine methylester **1a** dissolved in 300 µL of 10 mM sodium phosphate buffer (NaP, pH 7.0), was added sodium cyanide (3 equiv.) and **selectfluor** (2 equiv.) and TEMPO (2 equiv.). The reaction mixture was stirred for 1 h. Sample was taken from the reaction mixture, injected into LC-MS to monitor the generation of N,N-dimethylphenylalanine methylester nitrile products **2a** and **2a'**, and the formation of radical coupling products. The reaction mixture was analyzed by HPLC using method A.

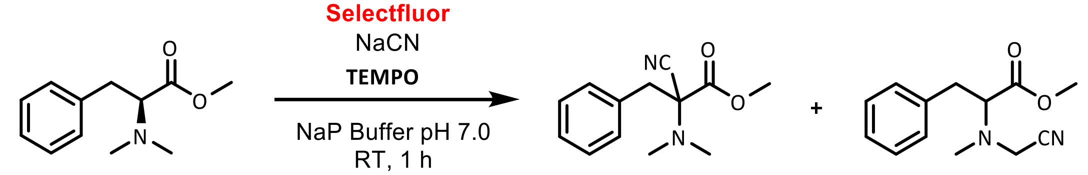

Exact Mass: 207.1259 — Exact Mass: 232.1212 — Exact Mass: 232.1212

1a — 2a — 2a'

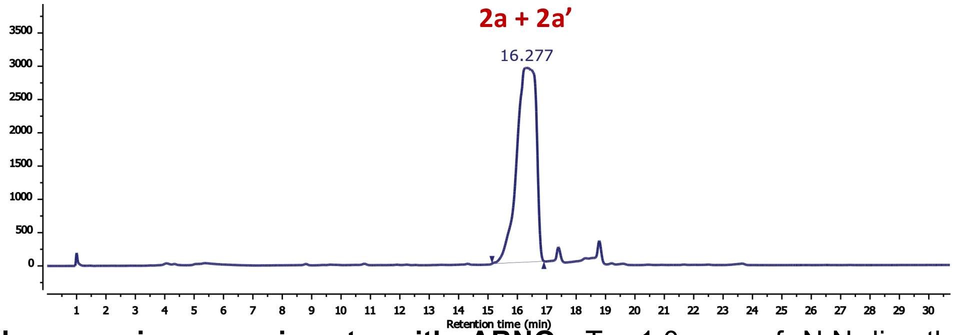

Radical-scavenging experiments with ABNO: To 1.0 mg of N,N-dimethylphenylalanine methylester **1a** dissolved in 300 µL of 10 mM sodium phosphate buffer (NaP, pH 7.0), was added sodium cyanide (3 equiv.), **selectfluor** (2 equiv.) and ABNO (2 equiv.). The reaction mixture was stirred for 1 h. Sample was taken from the reaction mixture, injected into LC-MS to

monitor the generation of N,N-dimethylphenylalanine methylester nitrile products **2a** and **2a’**, and the formation of radical coupling products. The reaction mixture was analyzed by HPLC using method A.

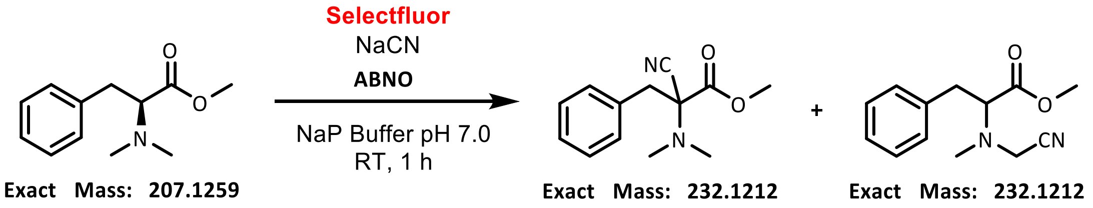

1a 2a 2a'

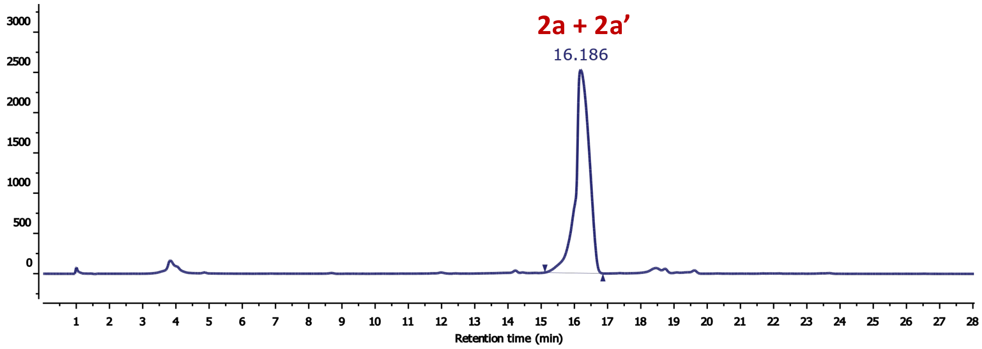

Radical-scavenging experiments with BHT: To 1.0 mg of N,N-dimethylphenylalanine methylester **1a** dissolved in 300 µL of 10 mM sodium phosphate buffer (NaP, pH 7.0), was added sodium cyanide (3 equiv.), **selectfluor** (2 equiv.) and BHT (2 equiv.). The reaction mixture was stirred for 1 h. Sample was taken from the reaction mixture, injected into LC-MS to monitor the generation of N,N-dimethylphenylalanine methylester nitrile products **2a** and **2a’**, and the formation of radical coupling products. The reaction mixture was analyzed by HPLC using method A.

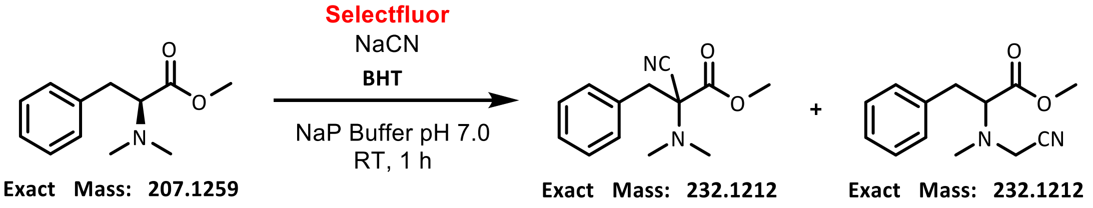

1a 2a 2a'

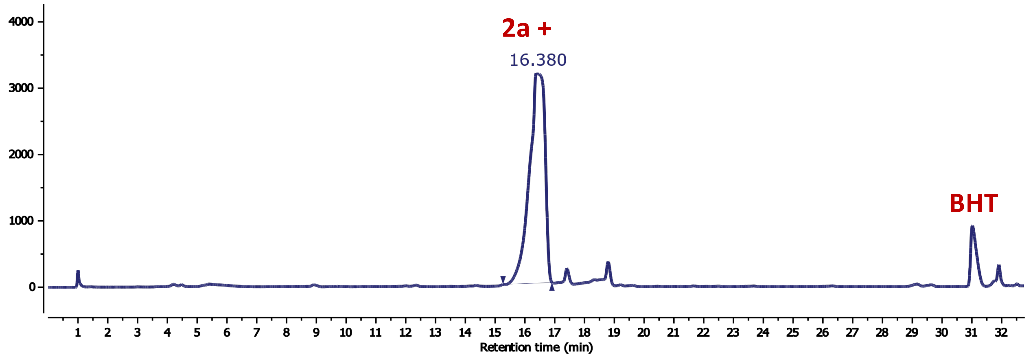

Computational evaluation of N-fluorination mechanism: Extensive DFT calculations did not identify a reaction pathway connecting N-fluorinated **1a** to the iminium ion intermediates.

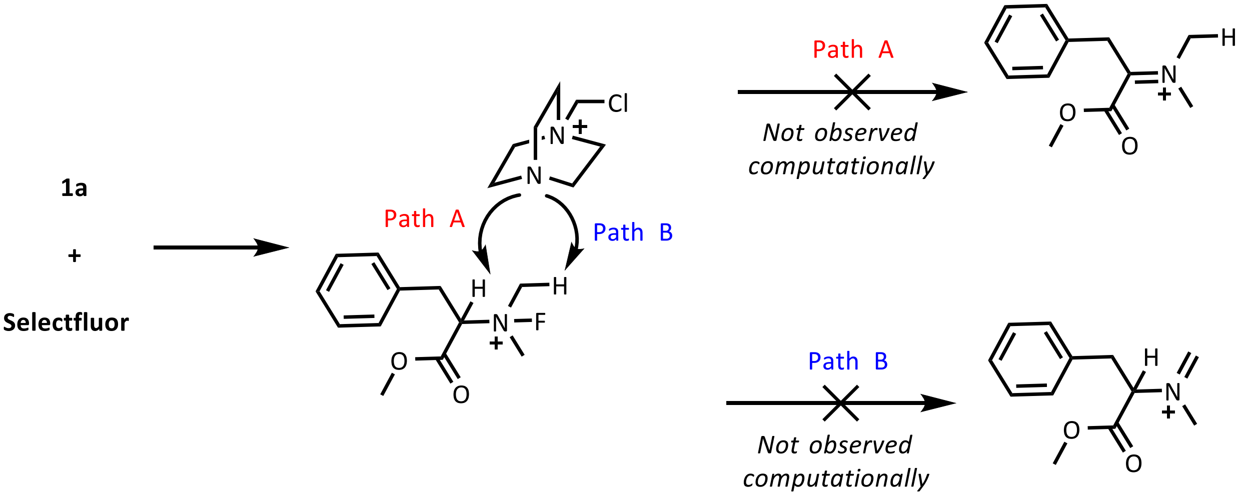

XII. Supplementary Figure 5. Chemoselectivity studies for Nitrile-peptide formation**.**

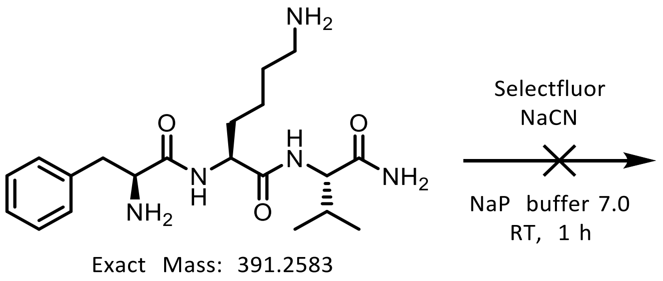

1.0 mg of lysine containing tripeptide FKV **1b** was modified using **general procedure 1.** The reaction mixture was analyzed by HPLC using method A to determine the % conversion.

FKV linear peptide **1b**. LCMS: m/z 392.15067 (calcd [M+H]+ = 392.2656), m/z 196.60472 (calcd [M+2/2]+ = 196.6291), m/z 783.37022 (calcd [2M+H]+ = 783.5240), (HPLC analysis at 220 nm). Retention time in HPLC: 6.496

HPLC Trace of chemoselectivity evaluation of lysine

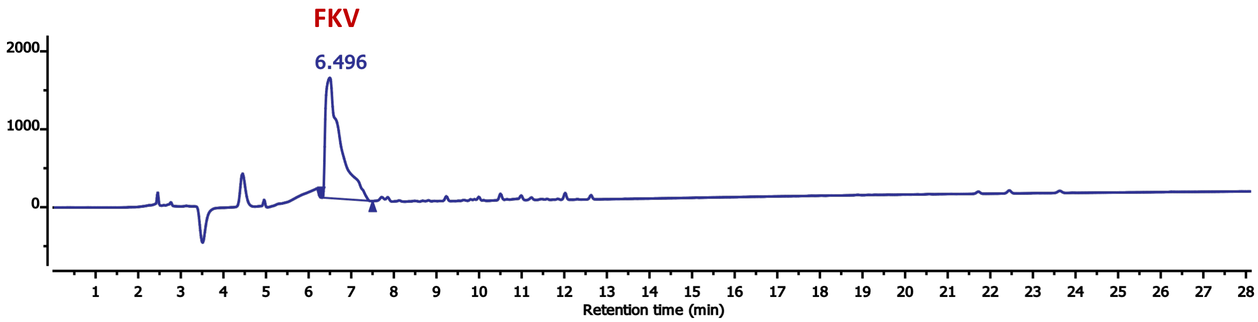

MS-Trace of peak 6.496 (1b)

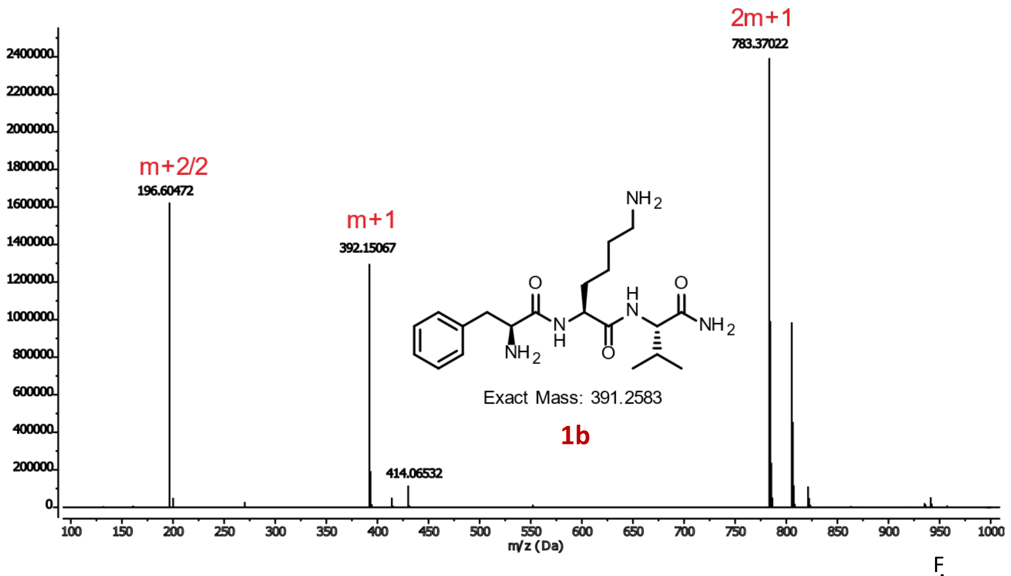

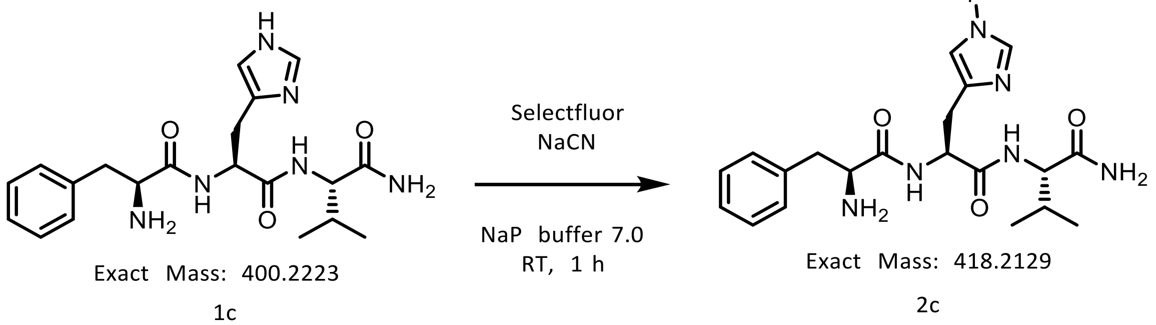

1.0 mg of histidine containing tripeptide FHV **1c** was modified using **general procedure 1.** The reaction mixture was analyzed by HPLC using method A to determine the % conversion. The fluorinated-FHV products was observed here (conversion 8%)

FHV linear peptide **1c**. LCMS: *m/z* 401.06929 (calcd [M+H]+ = 401.2296), *m/z* 201.03720 (calcd [M+2/2]+ = 201.1148), *m/z* 801.27315 (calcd [2M+H]+ = 801.4519), *m/z* 823.24819 (calcd [2M+Na]+ = 823.4338), (HPLC analysis at 220 nm). Retention time in HPLC: 6.536

FHV fluorination peptide products **2c**. LCMS: *m/z* 419.04150 (calcd [M+H]+ = 419.2201), *m/z* 210.02836 (calcd [M+2/2]+ = 210.1100), (HPLC analysis at 220 nm). Retention time in HPLC: 9.521

HPLC Trace of chemoselectivity evaluation of histidine

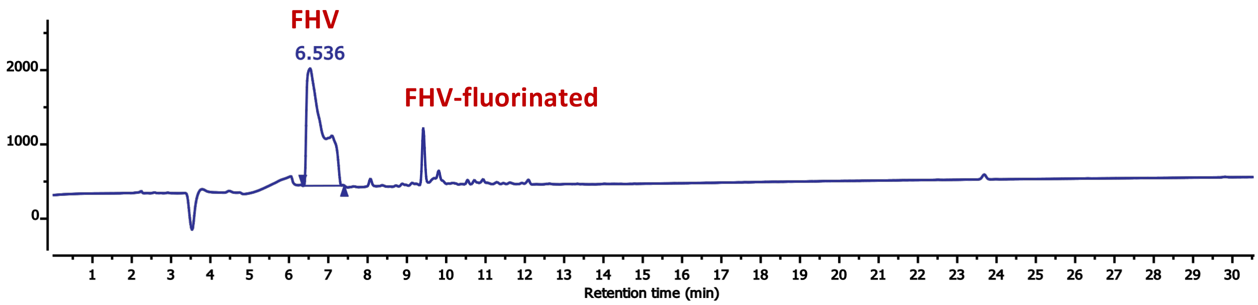

MS-Trace of peak 6.536 (1c)

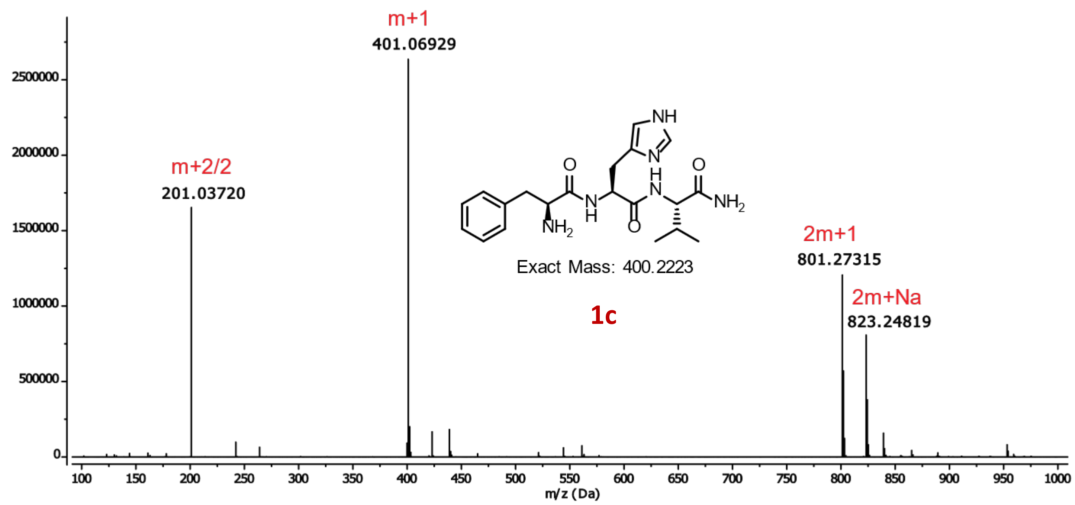

MS-Trace of peak 9.521 (2c)

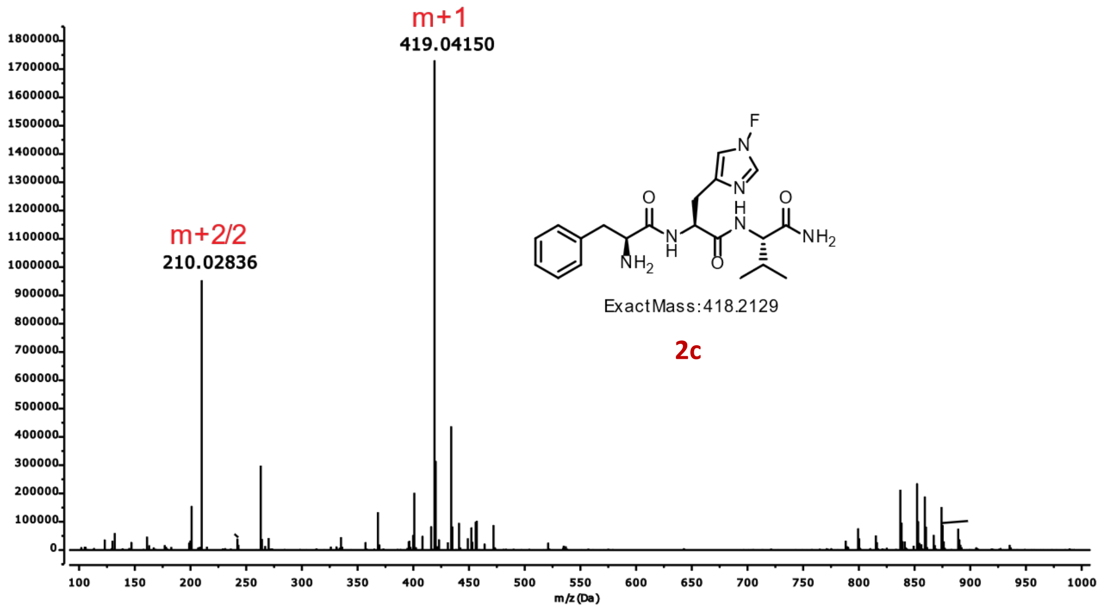

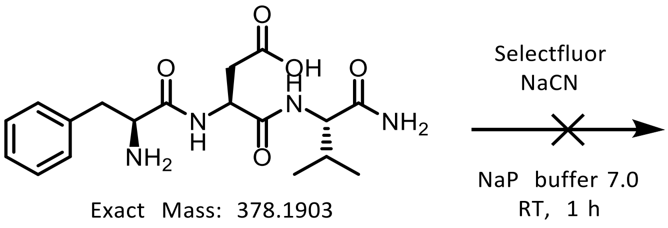

1.0 mg of aspartic acid containing tripeptide FDV **1d** was modified using **general procedure 1.** The reaction mixture was analyzed by HPLC using method A to determine the % conversion.

FDV linear peptide **1d**. LCMS: m/z 379.09165 (calcd [M+H]+ = 379.1976), m/z 401.02190 (calcd [M+Na]+ = 401.1795), m/z 417.03049 (calcd [M+K]+ = 417.1535), (HPLC analysis at 220 nm). Retention time in HPLC: 9.252

HPLC Trace of chemoselectivity evaluation of aspartic acid

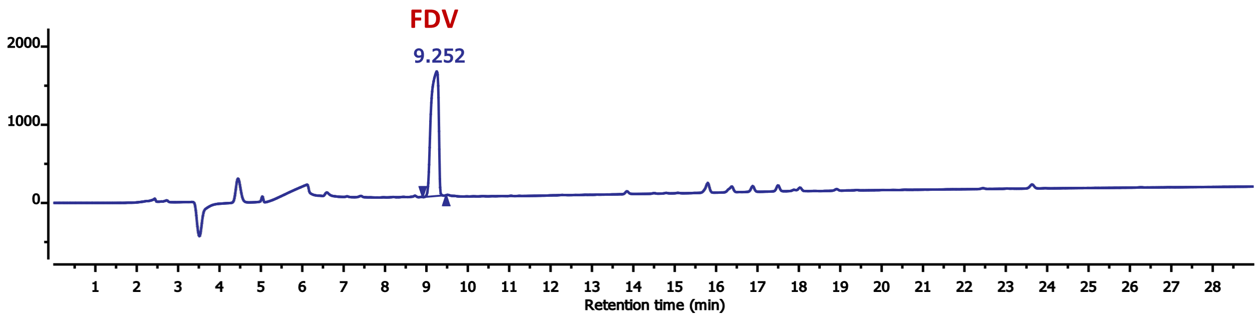

MS-Trace of peak 9.252 (1d)

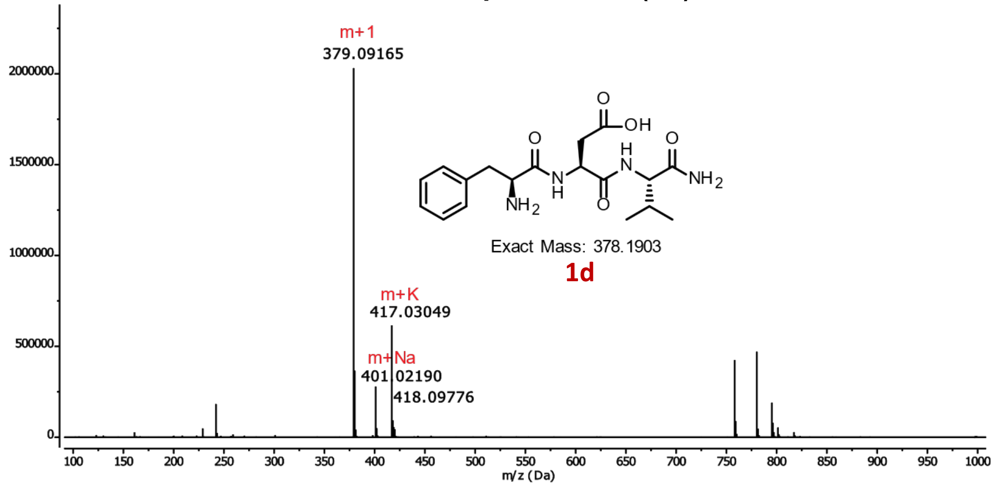

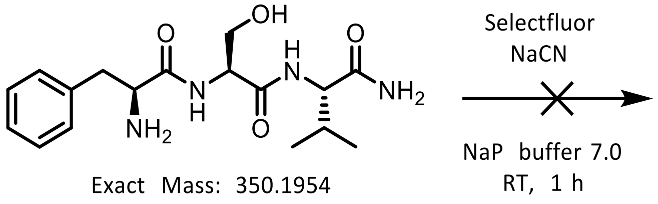

1.0 mg of serine containing tripeptide FSV **1e** was modified using **general procedure 1.** The reaction mixture was analyzed by HPLC using method A to determine the % conversion.

FSV linear peptide **1e**. LCMS: *m/z* 351.15584 (calcd [M+H]+ = 351.2027), *m/z* 373.16921 (calcd [M+Na] = 373.16), *m/z* 389.13582 (calcd [M+K] = 389.1591), *m/z* 701.44533 (calcd [2M+H]+ = 701.3981), *m/z* 723.43486 (calcd [2M+Na] = 723.2800), (HPLC analysis at 220 nm). Retention time in HPLC: 7.878

HPLC Trace of chemoselectivity evaluation of serine

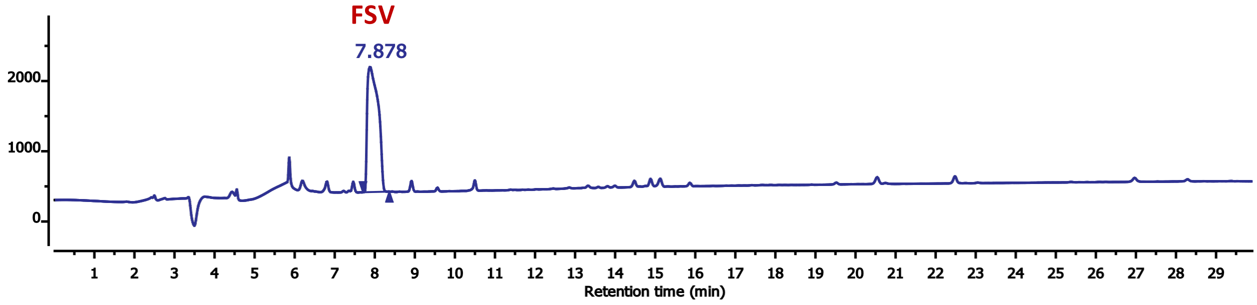

MS-Trace of peak 7.878 (1e)

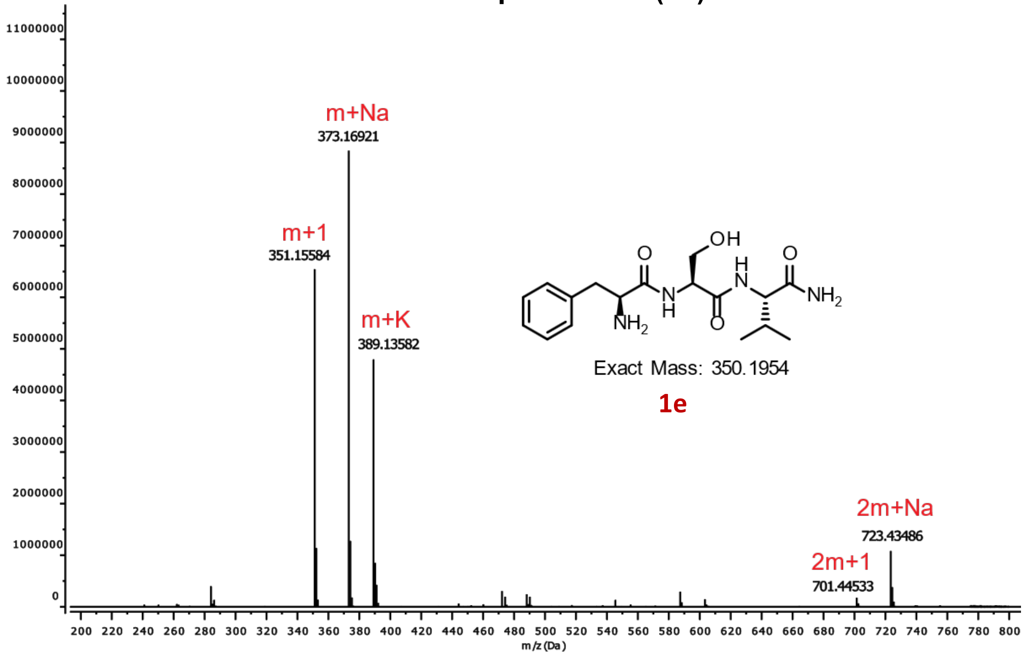

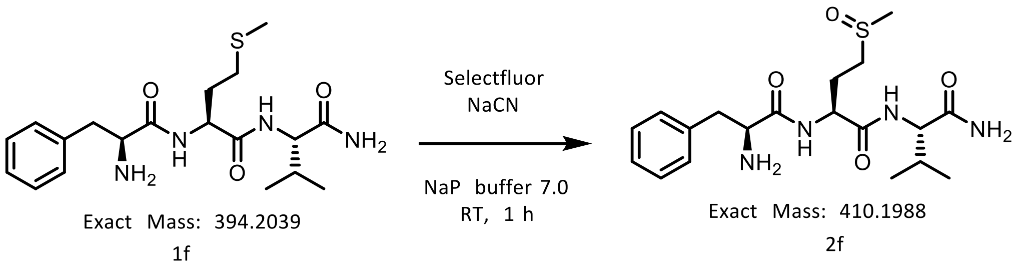

1.0 mg of methionine containing tripeptide FMV **1f** was modified using **general procedure 1.** The reaction mixture was analyzed by HPLC using method A to determine the % conversion. The sulfoxide-FMV product was observed here (conversion 98%).

FMV linear peptide **1f**. LCMS: *m/z* 395.06721 (calcd [M+H]+ = 395.2111), (HPLC analysis at 220 nm). Retention time in HPLC: 6.339

FMV sulfoxide peptide products **2f**. LCMS: *m/z* 411.06721 (calcd [M+H]+ = 411.2061), *m/z* 821.22852 (calcd [2M+H]+ = 821.4048), *m/z* 843.19960 (calcd [2M+Na]+ = 843.3868), (HPLC analysis at 220 nm). Retention time in HPLC: 8.529

HPLC Trace of chemoselectivity evaluation of methionine

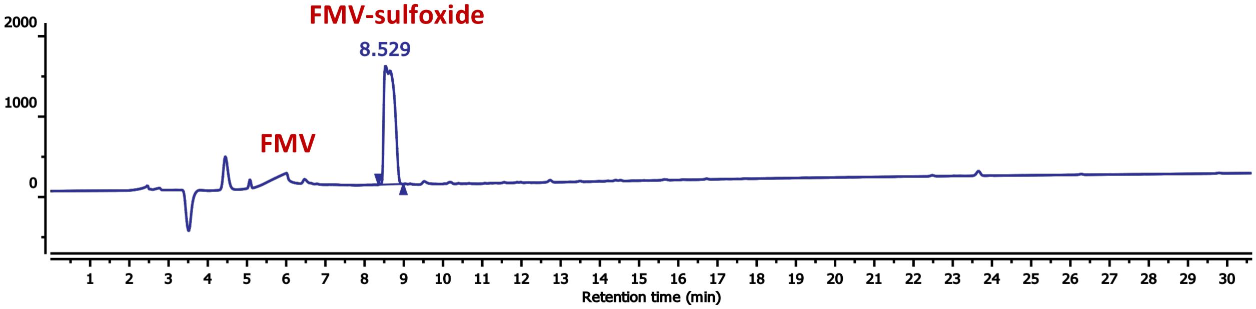

MS-Trace of peak 8.529 (1f)

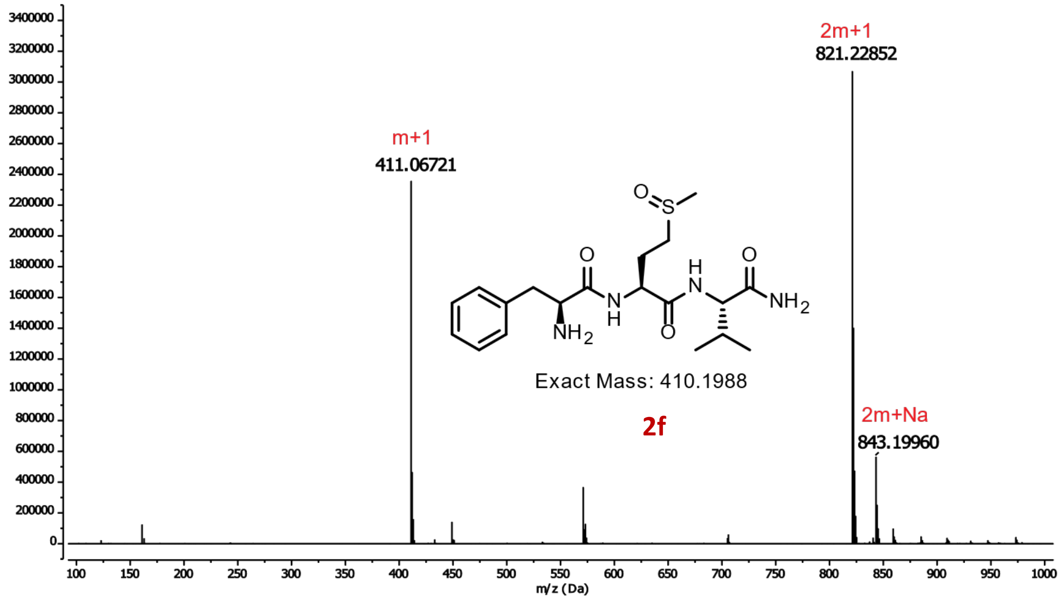

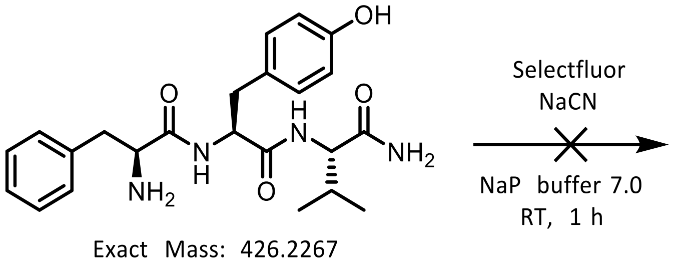

1.0 mg of tyrosine containing tripeptide FYV **1g** was modified using **general procedure 1.** The reaction mixture was analyzed by HPLC using method A to determine the % conversion.

FYV linear peptide **1g**. LCMS: *m/z* 427.22984 (calcd [M+H]+ =427.2340), *m/z* 449.18843 (calcd [M+Na]+ = 449.2159), *m/z* 465.23626 (calcd [M+K]+ = 465.1899), (HPLC analysis at 220 nm). Retention time in HPLC: 10.150

HPLC Trace of chemoselectivity evaluation of tyrosine

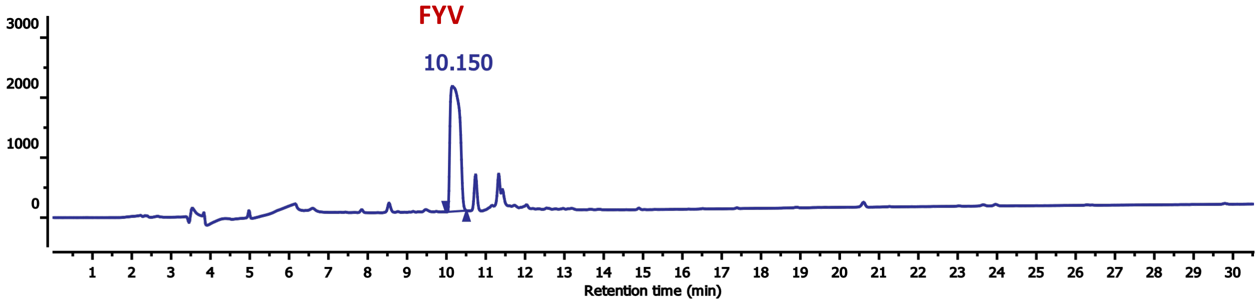

MS-Trace of peak 10.150 (1g)

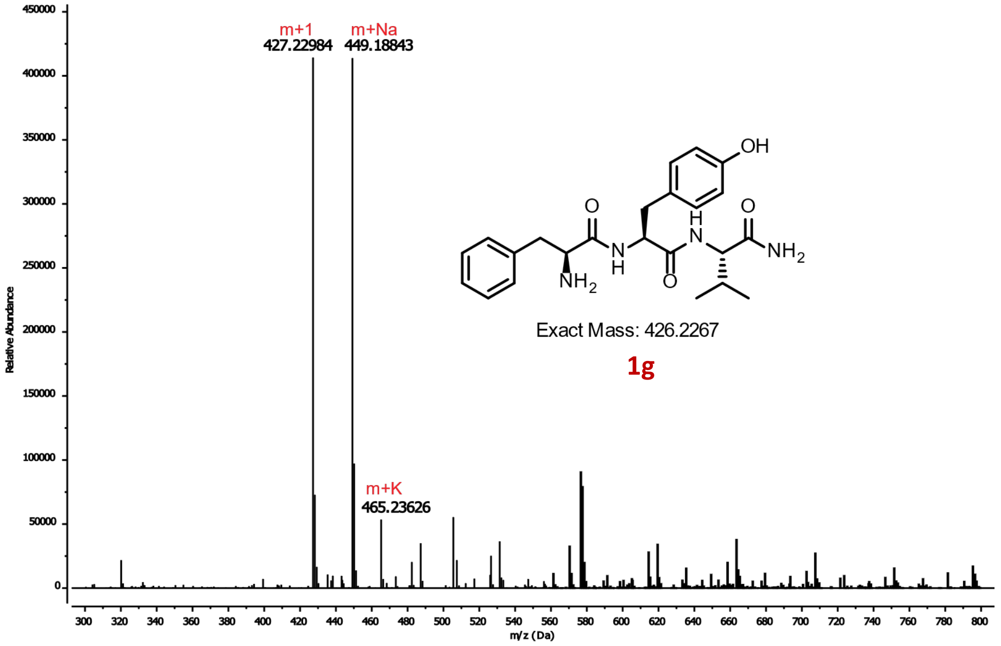

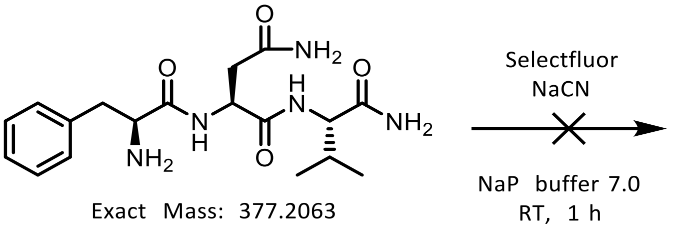

1.0 mg of asparagine containing tripeptide FNV **1h** was modified using **general procedure 1.** The reaction mixture was analyzed by HPLC using method A to determine the % conversion.

FNV linear peptide **1h.** LCMS: *m/z* 378.19993 (calcd [M+H]+ = 378.2136), *m/z* 400.20011 (calcd [M+Na]+ = 400.1955), *m/z* 416.14965 (calcd [M+K]+ = 416.1695), *m/z* 755.46067 (calcd [2M+H]+ = 755.4199), *m/z* 777.44211 (calcd [2M+Na]+ = 777.4018), (HPLC analysis at 220 nm). Retention time in HPLC: 7.852

HPLC Trace of chemoselectivity evaluation of asparagine

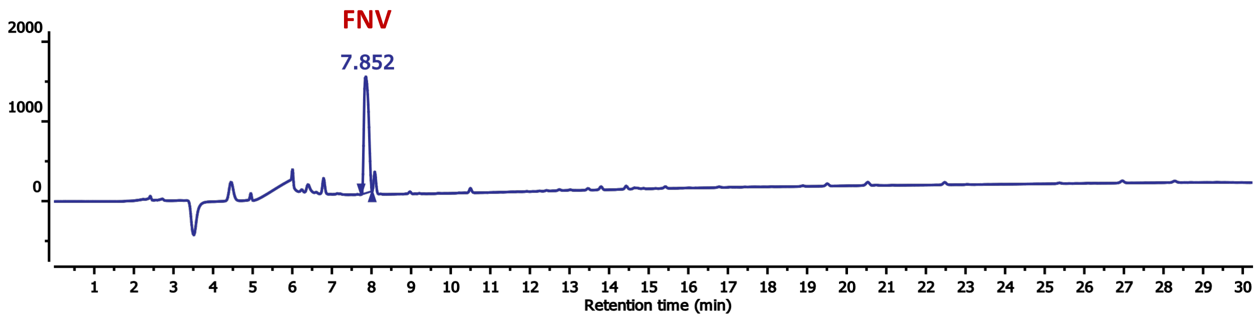

MS-Trace of peak 7.852 (1h)

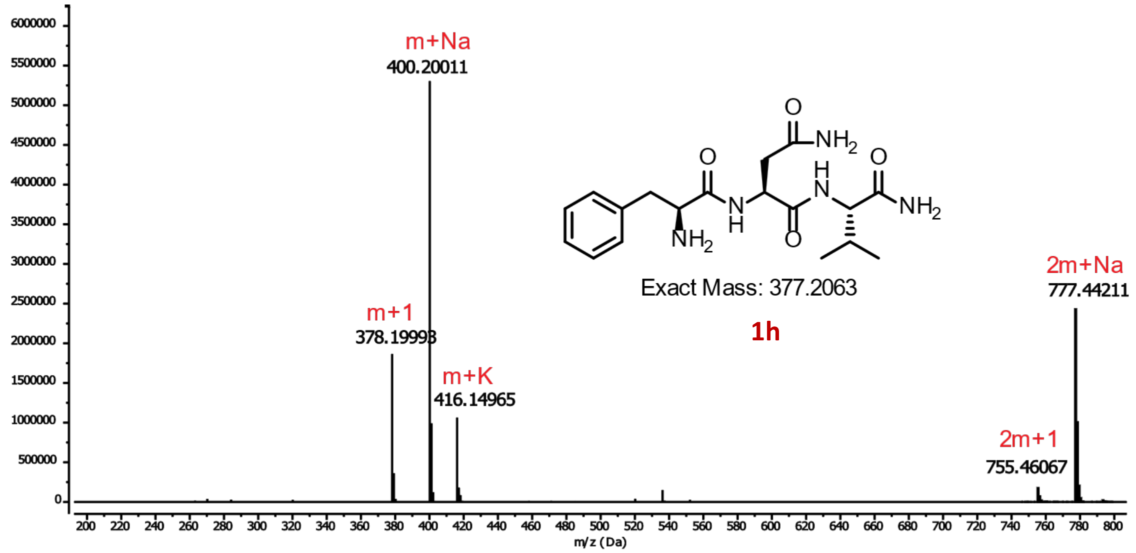

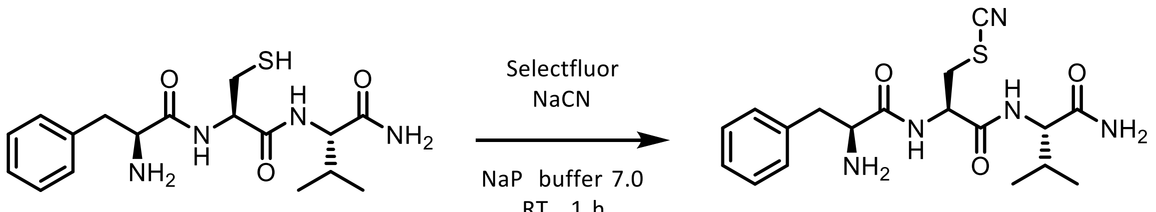

Exact Mass: 366.1726

1i

Exact Mass: 391.1678

2i

1.0 mg of cysteine containing tripeptide FCV **1i** was modified using **general procedure 1.** The reaction mixture was analyzed by HPLC using method A to determine the % conversion. The thiocyanate products was observed here (conversion >98%)

FCV linear peptide **1i**. Purity: >95 % (HPLC analysis at 220 nm). Retention time in HPLC: 8.208

FCV thiocyanate peptide products **2i**. LCMS: m/z 392.08536 (calcd [M+H]+ = 392.08), m/z 414.05780 (calcd [M+Na]+ = 414.3900) Purity: >95 % (HPLC analysis at 220 nm). Retention time in HPLC: 8.802

HPLC Trace of chemoselectivity evaluation of cysteine

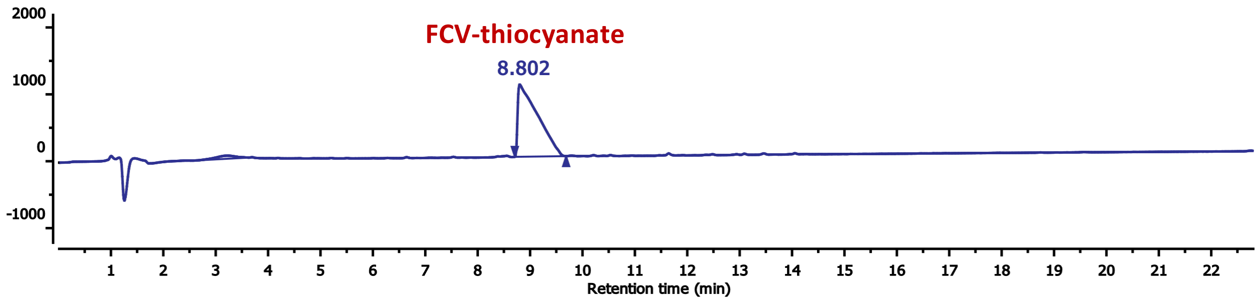

MS-Trace of FCV-thiocyanate peptide products 2i (peak 8.802)

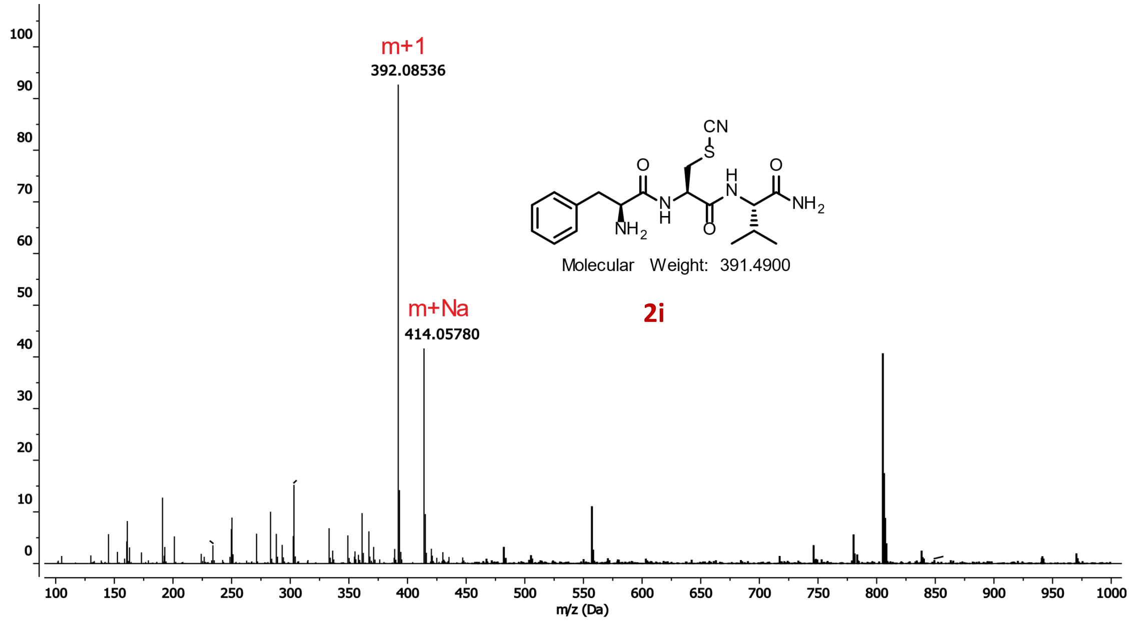

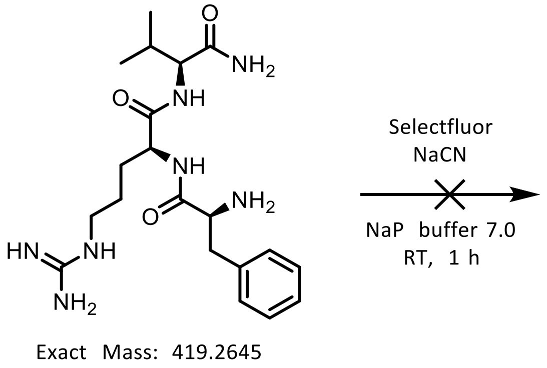

1j

1.0 mg of arginine containing tripeptide FRV **1j** was modified using **general procedure 1.** The reaction mixture was analyzed by HPLC using method A to determine the % conversion.

FRV linear peptide **1j**. LCMS: *m/z* 420.18400 (calcd $[M+H]^+$ = 420.2718), *m/z* 210.59435 (calcd $[M+2/2]^+$ = 210.5659), *m/z* 839.49061 (calcd $[2M+H]^+$ = 839.5363), (HPLC analysis at 220 nm). Retention time in HPLC: 4.740

HPLC Trace of chemoselectivity evaluation of arginine

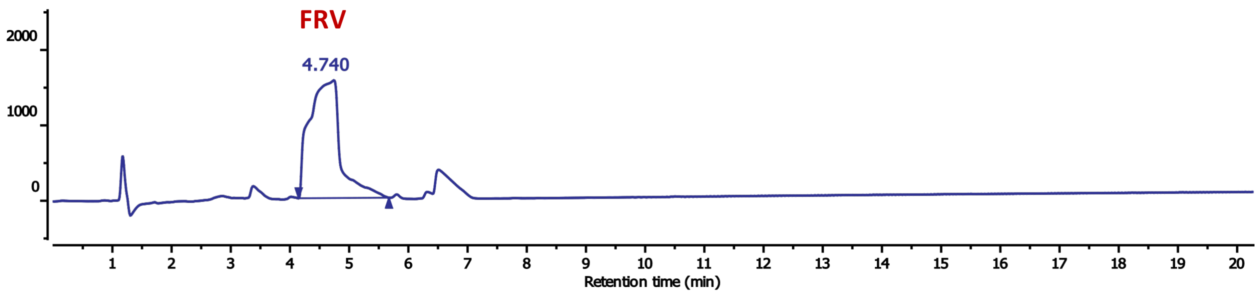

MS-Trace of peak 4.740 (1j)

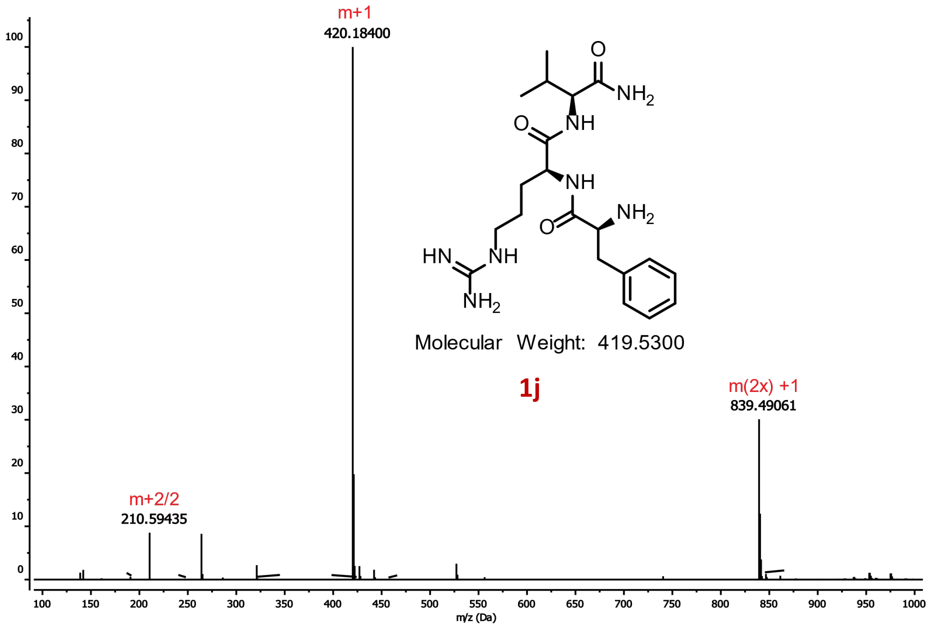

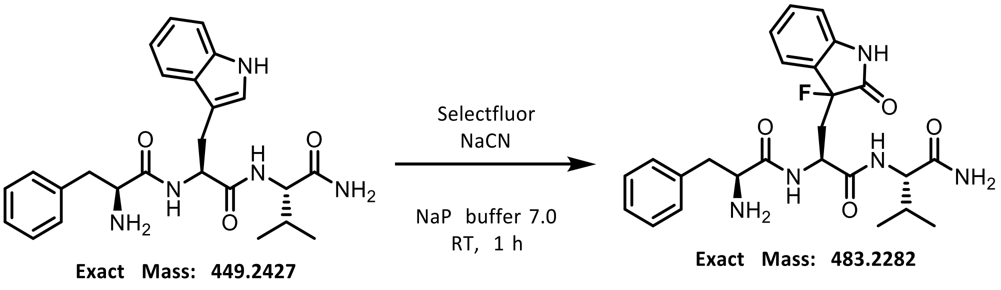

1k

2k

1.0 mg of tryptophan containing tripeptide FWV **1k** was modified using **general procedure 1.** The reaction mixture was analyzed by HPLC using method A to determine the % conversion. The fluorinated products was observed here (conversion 43%)

FWV linear peptide **1k**. LCMS: m/z 450.26093 (calcd [M+H]+ = 450.2500), LCMS: m/z 472.21833 (calcd [M+Na]+ = 472.2319), (HPLC analysis at 220 nm). Retention time in HPLC: 9.763

FWV fluorinated peptide products **2k**. LCMS: m/z 484.10143 (calcd [M+H]+ = 484.2355), (HPLC analysis at 220 nm). Retention time in HPLC: 10.463

HPLC Trace of chemoselectivity evaluation of tryptophan

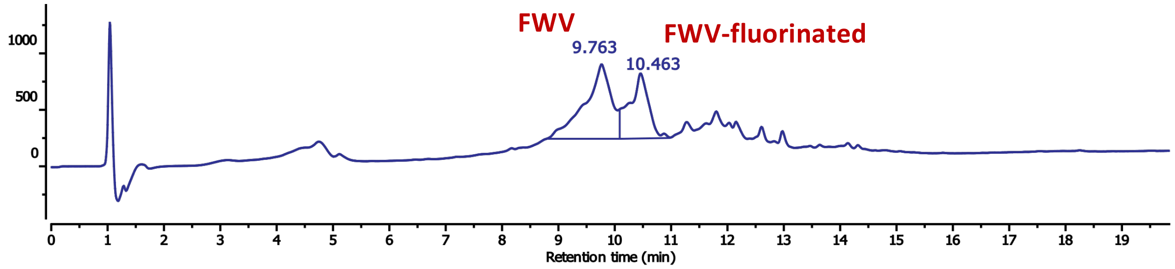

MS-Trace of peak 9.763 (1k)

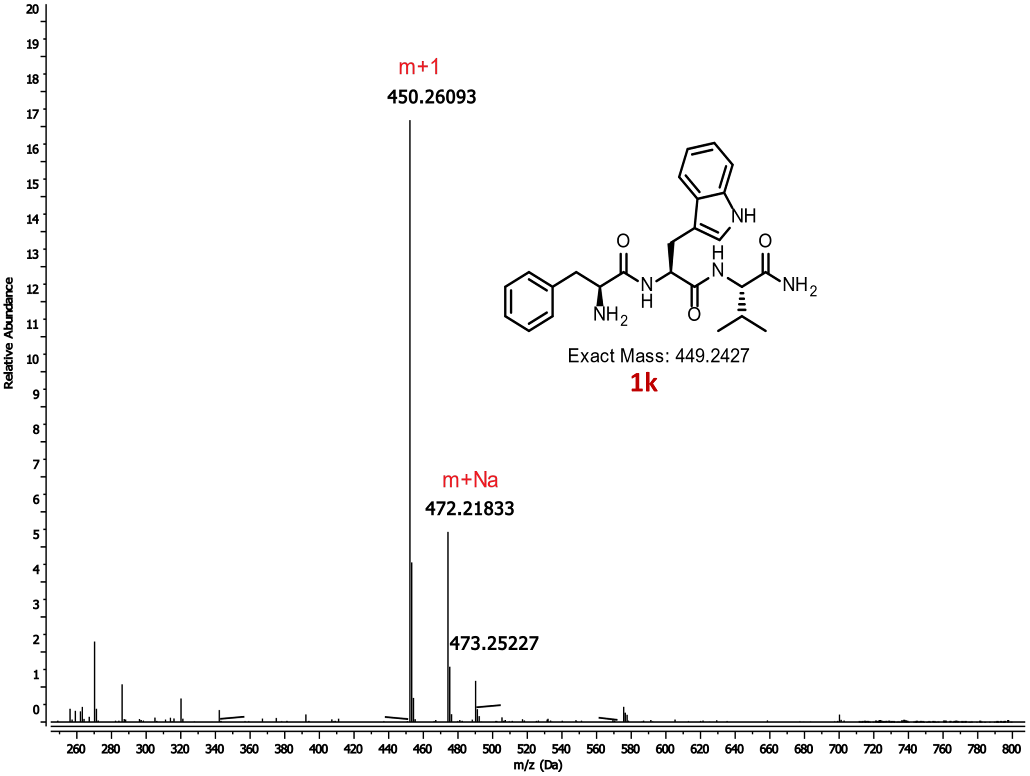

MS-Trace of fluorinated peptide products 3k (peak 10.463)

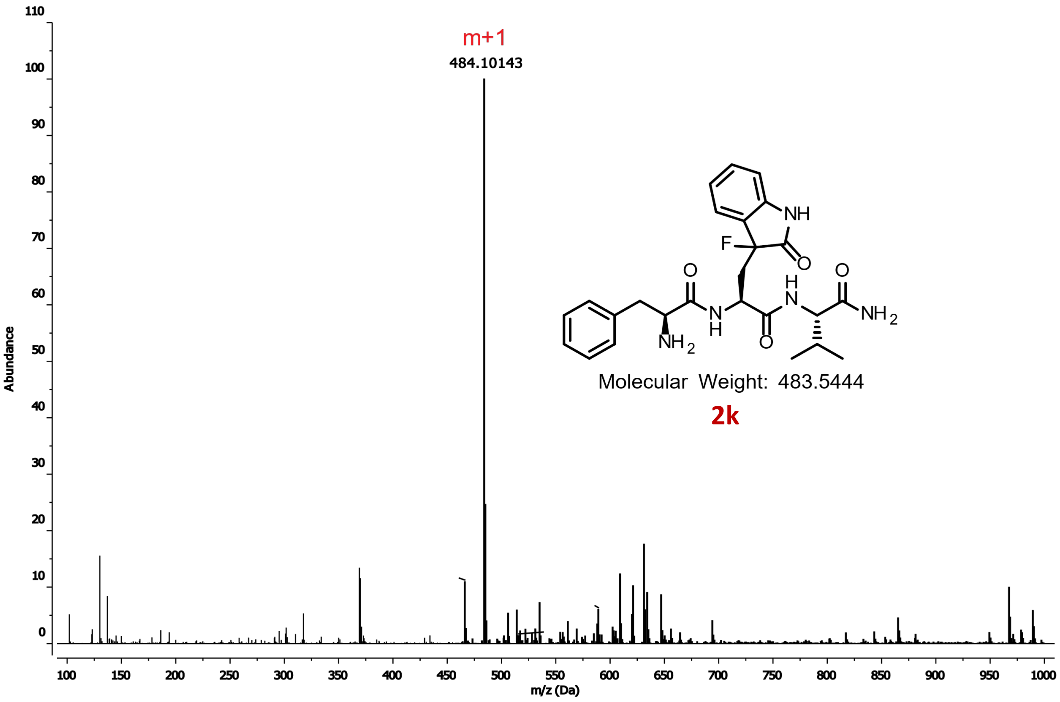

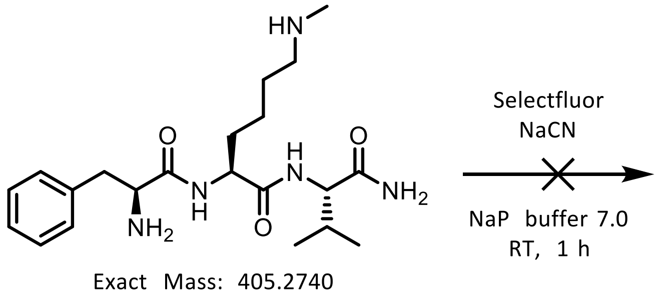

1.0 mg of monomethyllysine peptide FKme$_1$V **1l** was modified using **general procedure 1.** The reaction mixture was analyzed by HPLC using method A to determine the % conversion.

FKme$_1$V linear peptide **1l**. LCMS: m/z 406.29870 (calcd [M+H]+ = 406.54), m/z 811.59185 (calcd [2M+H]+ = 811.59), (HPLC analysis at 220 nm). Retention time in HPLC: 6.436

HPLC Trace of chemoselectivity evaluation of monomethyllysine

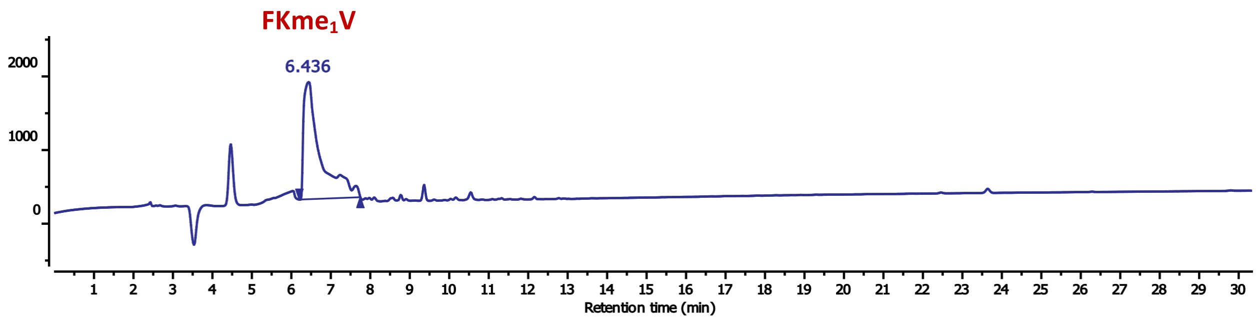

MS-Trace of peak 6.436 (1l)

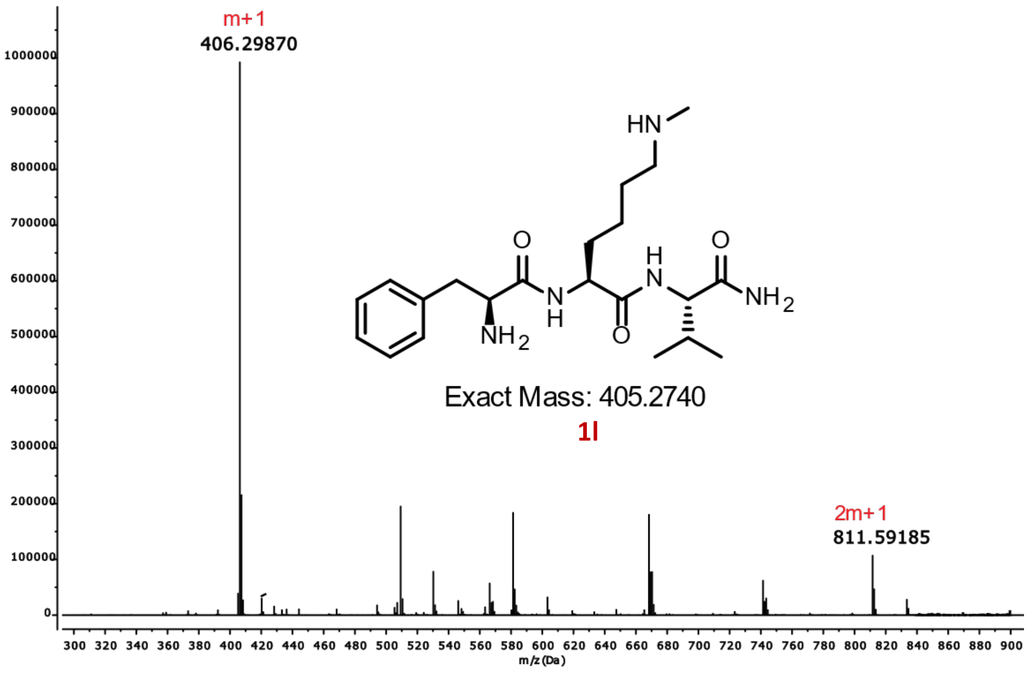

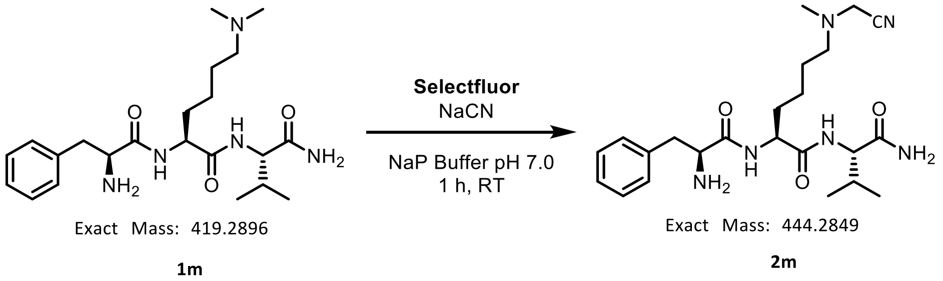

1.0 mg of dimethyllysine peptide FKme_2V **1m** was modified using **general procedure 1.** The reaction mixture was analyzed by HPLC using method A to determine the % conversion.

FKme_2V peptide 1m. LCMS: *m/z* 420.29315 (calcd $[M+H]^+$ = 420.2969), (HPLC analysis at 220 nm). Retention time in HPLC: 5.228

FKme_2(CN)V nitrile-peptide products 2m. LCMS: *m/z* 445.18156 (calcd $[M+H]^+$ = 445.2922), *m/z* 223.15009 (calcd $[M+2/2]^+$ = 223.1424), *m/z* 467.12659 (calcd $[M+Na]^+$ = 467.2747), *m/z* 889.41710 (calcd $[2M+H]^+$ = 889.5771), *m/z* 911.42847 $[2m+Na]^+$ =911.5595. (HPLC analysis at 220 nm). Retention time in HPLC: 6.275

HPLC Trace of the reaction with dimethyllysine peptide 1m

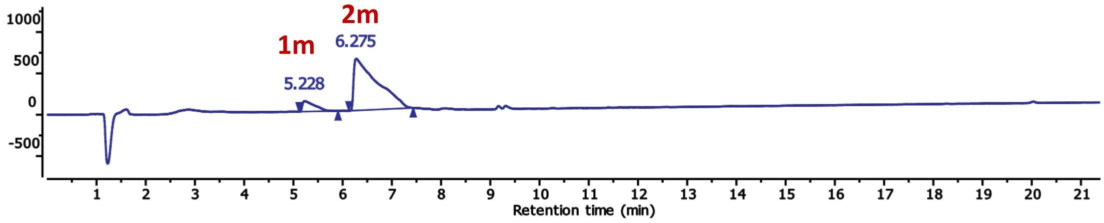

MS-Trace of 1m (peak 5.228)

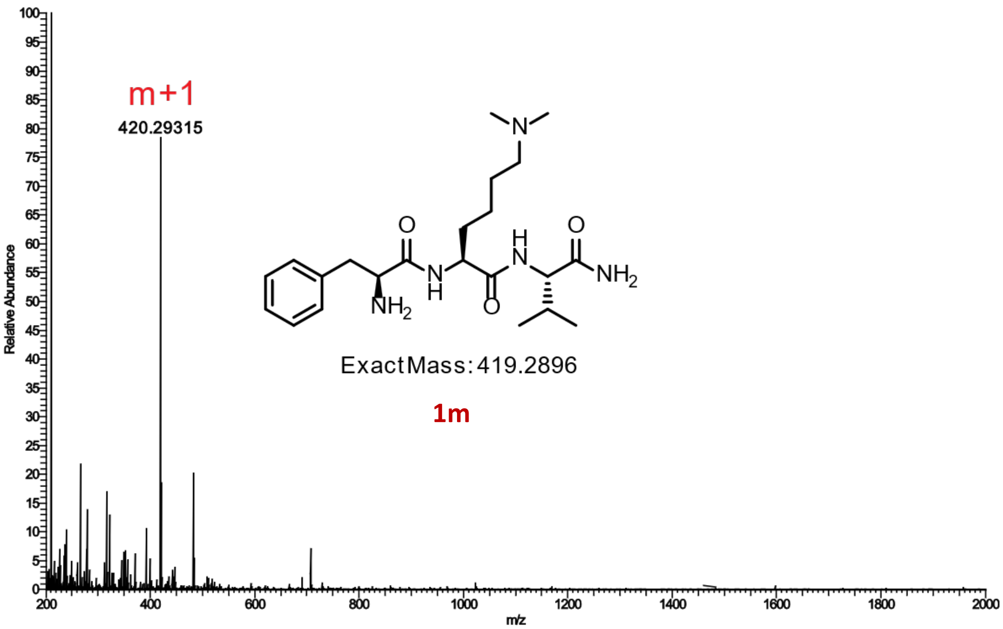

MS-Trace of 2m (peak 6.275)

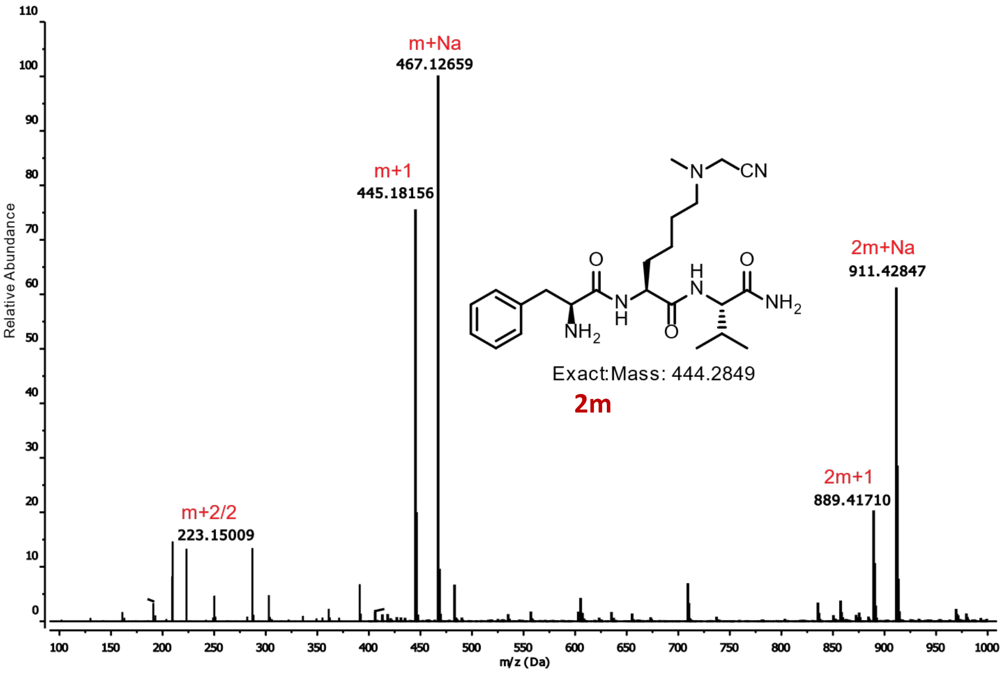

Synthesis of N-terminal monomethyl(Nme$_1$) peptide Nme$_1$AFL 1n: Peptides were synthesized manually on a 0.25 mm scale using a Leucine preloaded wang resin. Resin was swollen with CH_2Cl_2 for 1 h at room temperature. Fmoc was deprotected using 20% piperidine in DMF for 5 min to obtain a deprotected peptide-resin. Fmoc-protected phenylalanine (0.75 mm/3 equiv.) was coupled on the resin using HBTU (0.75 mm/3 equiv.) and DIEA (1.5 mm/6 equiv.) in

DMF for 5 min at room temperature, followed by the deprotection of the Fmoc group on the resin-bound dipeptide. Boc-N-Me-Ala-OH (0.75 mm/3 equiv.) was coupled on the resin using HBTU (0.75 mm/3 equiv.) and DIEA (1.5 mm/6 equiv.) in DMF for 5 min. Peptides were cleaved from the resin using a cocktail of 95:2.5:2.5, trifluoroacetic acid: water: TES for 2 h. The resin was removed by filtration and the resulting solution was concentrated. The residue was diluted with ACN/water mixture. The resulting solution was purified by HPLC and analyzed by MS to obtain pure Nme_1AFL **1n** peptide.

Chemoselectivity evaluation of N-terminal monomethyl(Nme_1) peptide Nme_1AFL 1n:

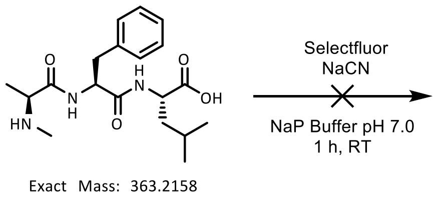

1n

1.0 mg of N-terminal monomethyl containing tripeptide (Nme_1)AFL **1n** was modified using **general procedure 1.** The reaction mixture was subjected to HPLC run using method A to determine the % conversion and analyzed by MS. No modification of the peptide was observed.

(Nme_1)AFL linear peptide 1n. LCMS: m/z 364.13485 (calcd [M]+H = 364.2231) (HPLC analysis at 220 nm). Retention time in HPLC: 6.584

HPLC trace of the reaction with N-methyl peptide (Nme_1)AFL (1n)

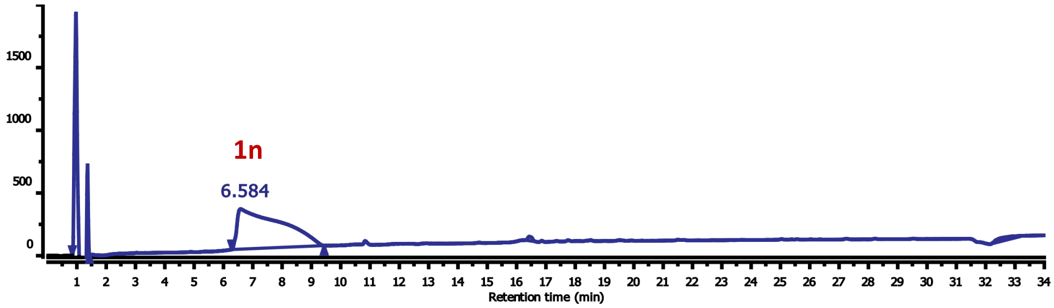

MS-Trace of peak 6.584

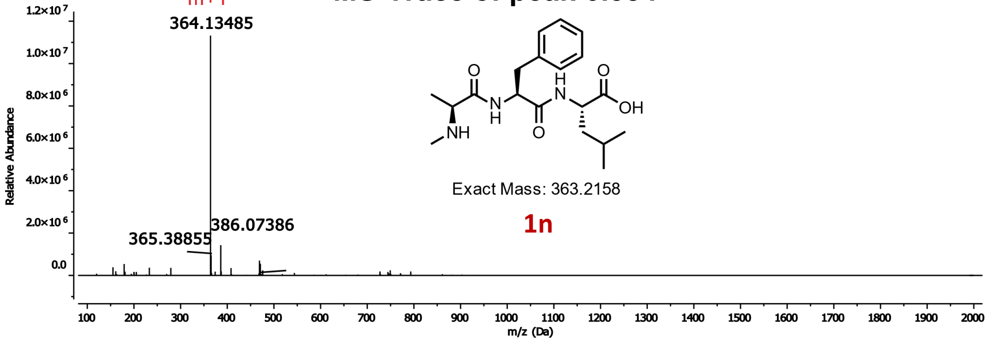

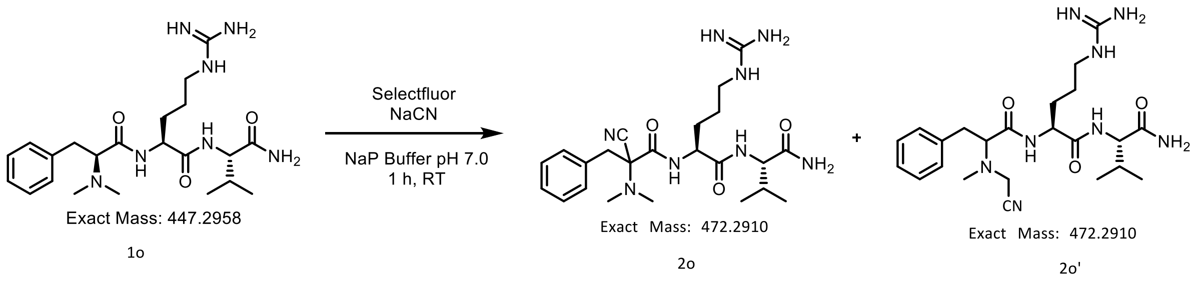

1.0 mg of dimethyllysine peptide Nme_2RV **1o** was modified using **general procedure 1.** The reaction mixture was analyzed by HPLC using method A to determine the % conversion.

N,N-dimethyl-FRV peptide 1o. Purity: >95 % (HPLC analysis at 220 nm). Retention time in HPLC: 5.280

N,N-dimethyl-FRV nitrile peptide products 2o+2o'. LCMS: *m/z* 473.33687 (calcd [M+H]+ = 473.2983), *m/z* 495.31514 (calcd [M+Na]+ = 495.2803), Purity: >95 % (HPLC analysis at 220 nm). Retention time in HPLC: 8.907

HPLC trace of reaction of 1o to 2o and 2o'

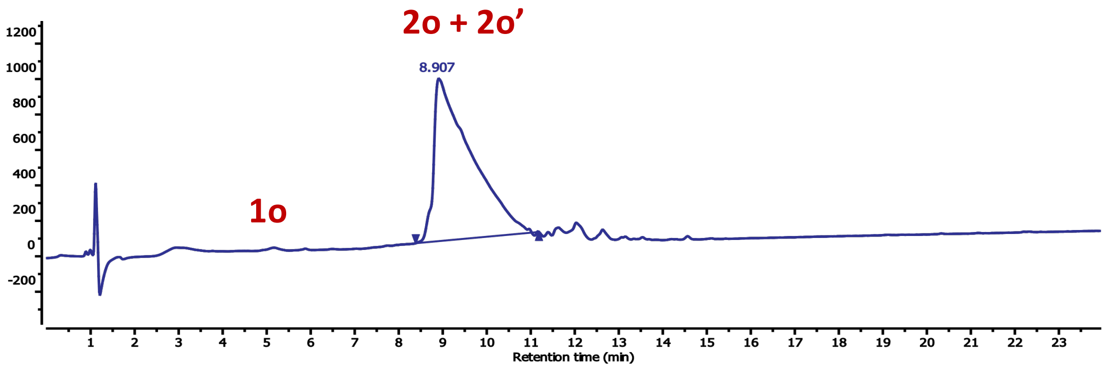

MS-trace of nitrile peptide products 2o + 2o' (peak 8.907)

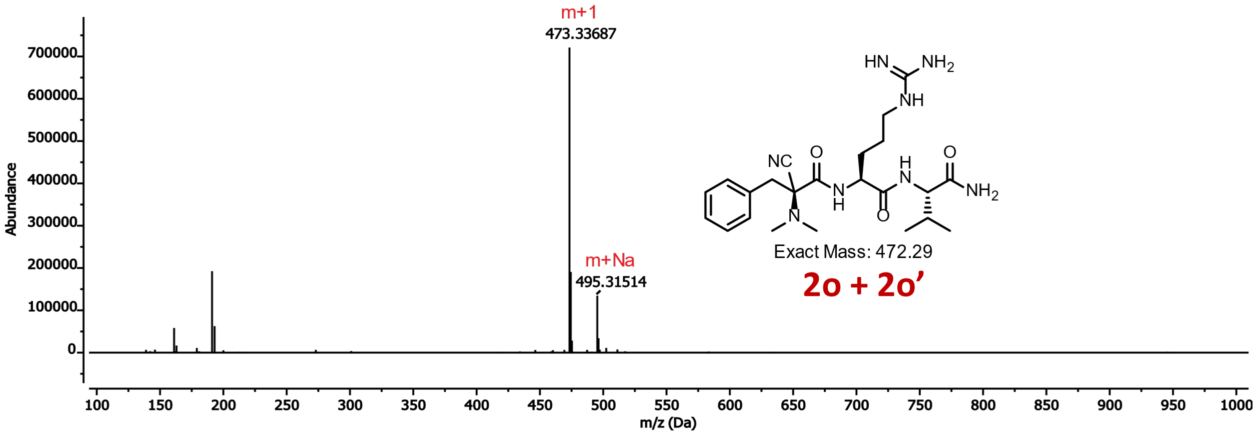

Synthesis of N-terminal trimethyl(Nme_3) peptide Nme_3GFL 1p: Peptides were synthesized manually on a 0.25 mm scale using a leucine preloaded wang resin. Resin was swollen with CH_2Cl_2 for 1 h at room temperature. Fmoc was deprotected using 20% piperidine in DMF for 5

min to obtain a deprotected peptide-resin. Fmoc-protected phenylalanine (0.75 mm/3 equiv.) was coupled on the resin using HBTU (0.75 mm/3 equiv.) and DIEA (1.5 mm/6 equiv.) in DMF for 5 min at room temperature, followed by the deprotection of the Fmoc group on the resin-bound dipeptide. Bromoacetic acid (0.75 mm/3 equiv.) was coupled on the resin using DIC (0.75 mm/3 equiv.) in DMF for 2 h. After washing the resin, resin-bound peptide was incubated with an excess of trimethylamine in THF for 24 h. Peptides were cleaved from the resin using a cocktail of 95:2.5:2.5, trifluoroacetic acid: water: TES for 2 h. The resin was removed by filtration and the resulting solution was concentrated. The residue was diluted with ACN/water mixture. The resulting solution was purified by HPLC and analyzed by MS to obtain pure Nme_3GFL peptide **1p**.

Chemoselectivity evaluation of N-terminal trimethyl(Nme_3) Nme_3GFL 1p peptide:

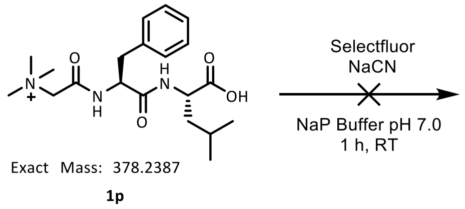

1.0 mg of N-terminal trimethyl containing tripeptide (Nme_3)GFL **1p** was modified using **general procedure 1.** The reaction mixture was subjected to HPLC run using method A to determine the % conversion as analyzed by MS. No modification of the peptide was observed.

(Nme_3)GFL linear peptide 1p. LCMS: m/z 378.19532 (calcd [M]+ = 378.2387) (HPLC analysis at 220 nm). Retention time in HPLC: 8.228

HPLC Trace of the reaction with N,N,N-trimethyl peptide (Nme_3)GFL 1p

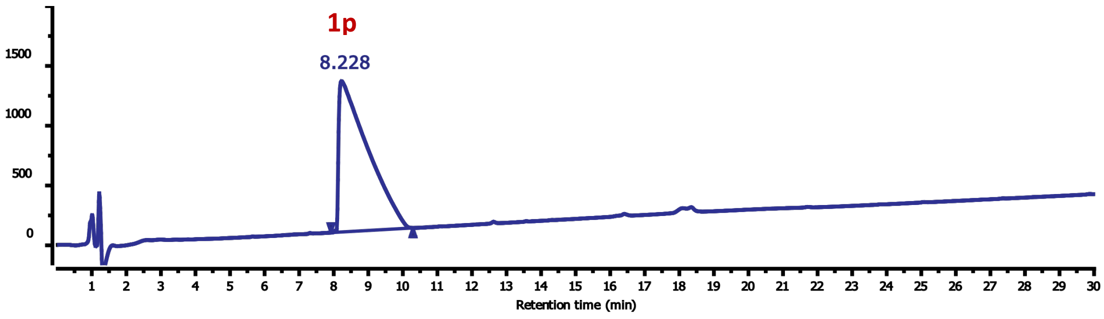

MS-Trace of peak 8.228

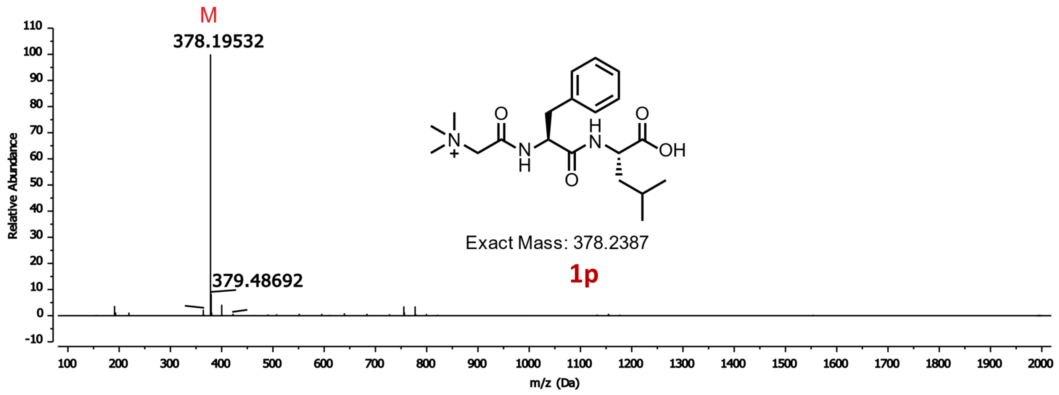

Summary of chemoselectivity studies

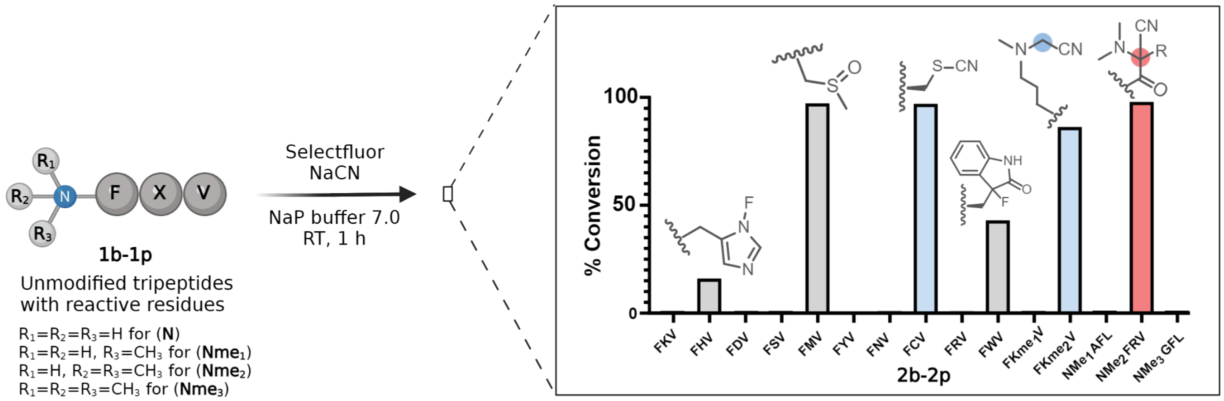

XIII. Supplementary Figure 6. Characterization of nitrile-peptide products using a small molecule dimethyllysine-mimic model compound *N,N*-dimethyl,4-phenylbutylamine.

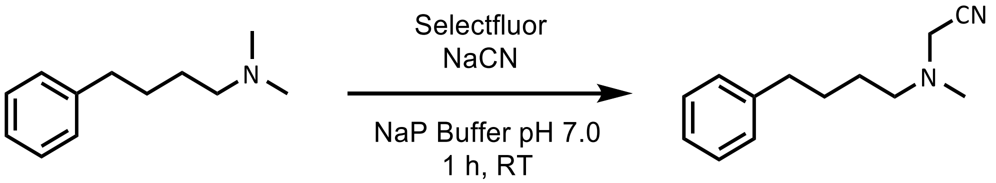

100 mg of *N,N*-dimethyl,4-phenylbutylamine was modified using **general procedure 1**. The mixture was concentrated and purified by silica gel column chromatography (eluent: 100 % CH_2Cl_2) to generate the 88 mg of the nitrile products methyl(4-phenylbutyl)amino]acetonitrile in 77% yield.

***N,N*-dimethyl,4-phenylbutylamine.** ^{1}**H NMR** (600 MHz, $CDCl_3$) δ 7.29 – 7.25 (m, 2H), 7.24 – 7.15 (m, 3H), 2.63 (t, *J* = 7.8 Hz, 2H), 2.26 (t, *J* = 7.8 Hz, 2H), 2.20 (s, 8H), 1.63 (p, *J* = 7.7 Hz, 2H), 1.50 (p, *J* = 7.7 Hz, 2H).

Methyl(4-phenylbutyl) amino]acetonitrile. ^{1}**H NMR** (600 MHz, $CDCl_3$) δ 7.33 – 7.25 (m, 2H), 7.23 – 7.17 (m, 3H), 2.65 (t, *J* = 7.2 Hz, 2H), 2.47 (t, *J* = 7.2 Hz, 2H), 1.67 (p, *J* = 7.5 Hz, 2H), 1.50 (p, *J* = 7.5 Hz, 2H). ^{13}**C NMR** (151 MHz, $CDCl_3$) δ 142.16, 128.48, 128.40, 128.38, 128.36, 125.83, 114.70, 55.57, 45.10, 42.01, 35.62, 28.80, 26.90.

^{1}H NMR of *N,N*-dimethyl-4-phenylbutan-1-amine

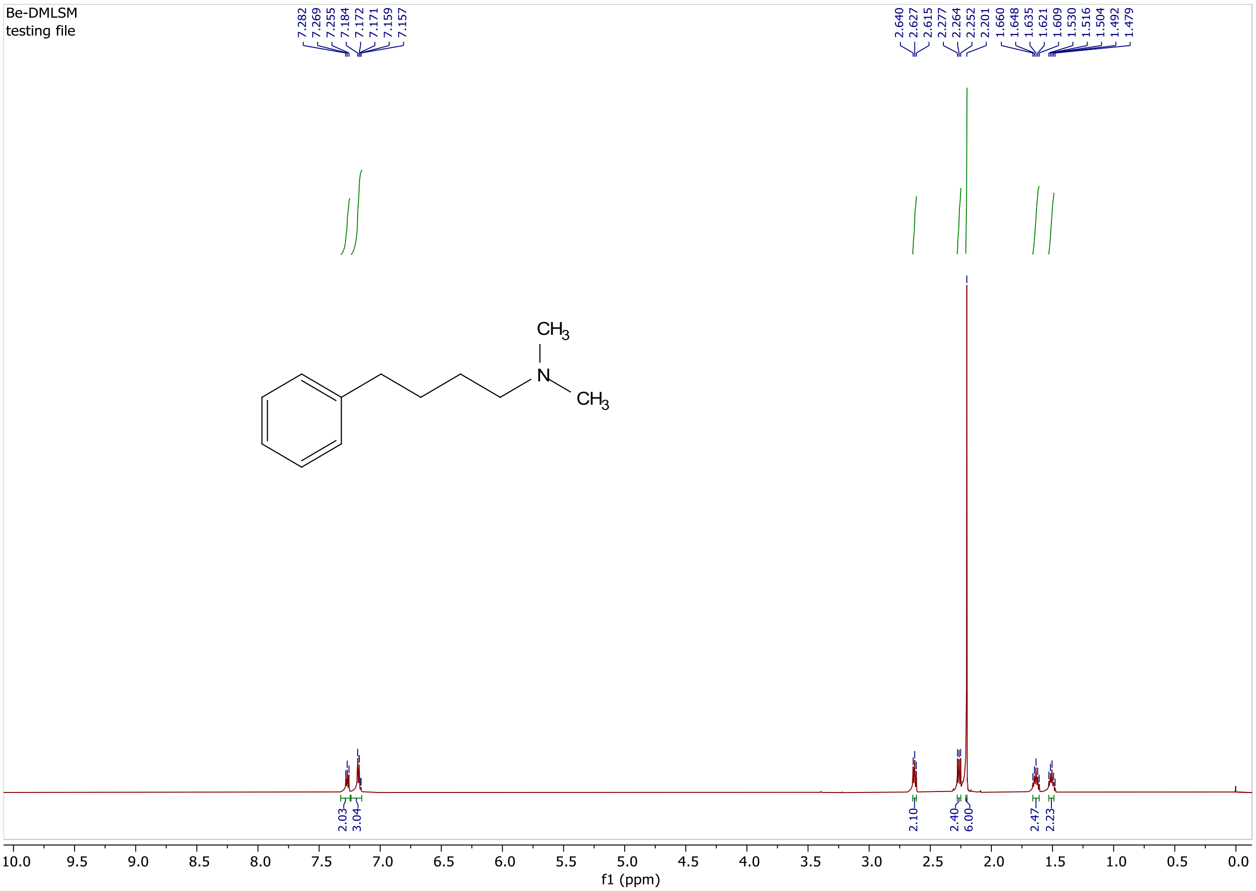

^{1}H NMR of nitrile products 2-(methyl(4-phenylbutyl)amino)acetonitrile

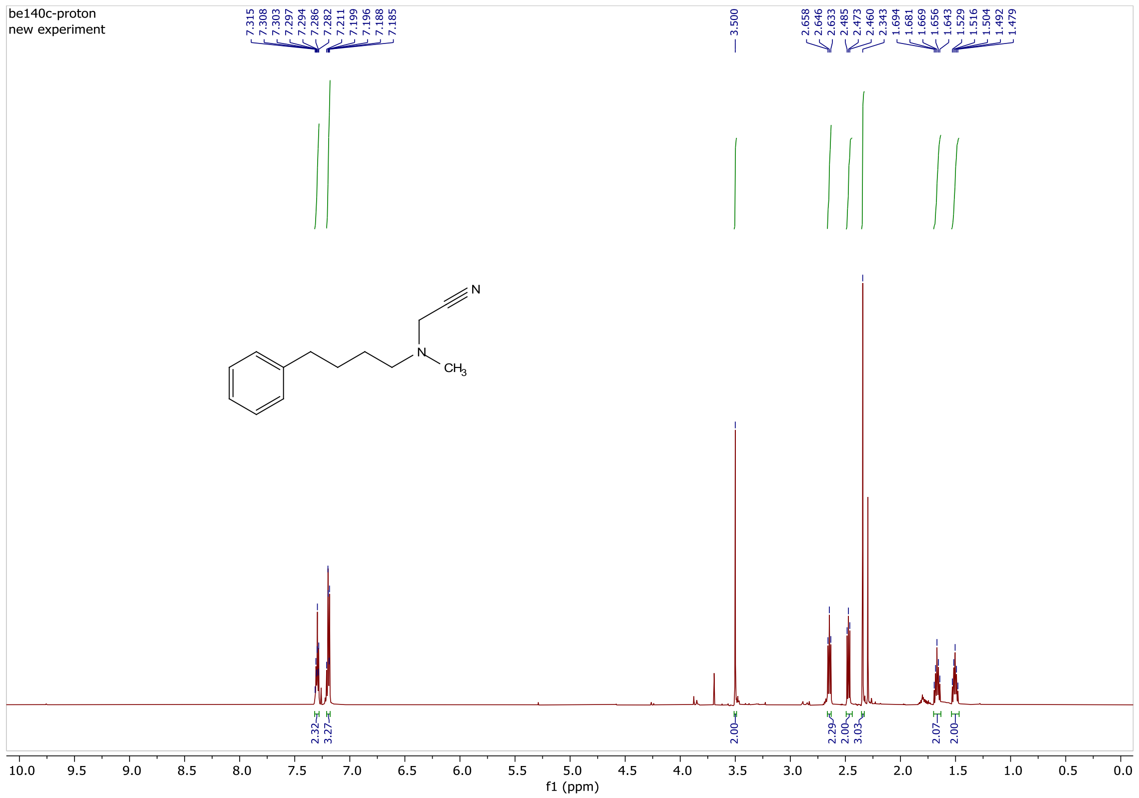

^{13}C NMR of nitrile products 2-(methyl(4-phenylbutyl)amino)acetonitrile

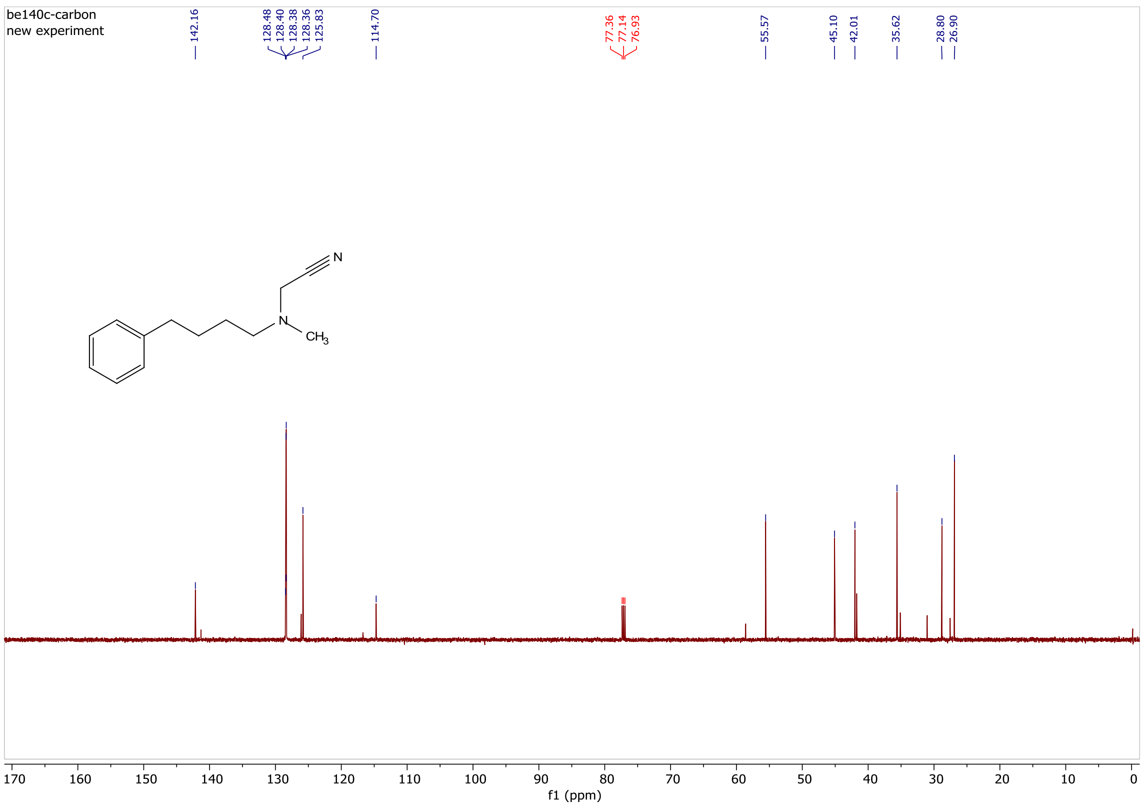

XIV. Supplementary Figure 7. NMR characterization of chemoselectivity adducts.

Formation of thiocyanate.

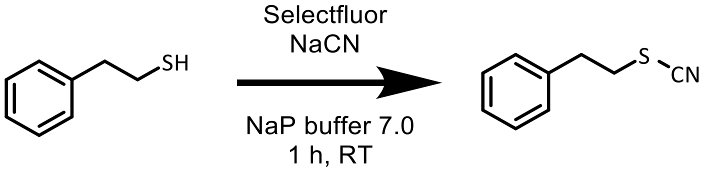

100 mg of 2-Phenyl-1-ethanethiol was modified using **general procedure 1.** Concentration of the mixture gave a residue, which was purified by silica gel column chromatography (eluent: hexane/EtOAc = 4:1) to give thiocyanate products 2-phenylethyl thiocyanate in 81% yield.

.

2-Phenylethanethiol. ^{1}**H NMR** (600 MHz, $CDCl_3$) δ 7.34 – 7.31 (m, 2H), 7.28 – 7.19 (m, 3H), 2.94 (t, J = 7.4 Hz, 2H), 2.81 (q, J = 7.6 Hz, 2H), 1.40 (t, J = 7.8 Hz, 1H). ^{13}**C NMR** (151 MHz, $CDCl_3$) δ 139.85, 128.70, 128.54, 126.55, 40.29, 26.09.

2-Phenylethyl thiocyanate. ^{1}**H NMR** (600 MHz, $CDCl_3$) δ 7.39 – 7.33 (m, 2H), 7.26 – 7.22 (m, 3H), 3.18 (t, J = 7.8 Hz, 2H), 3.12 (t, J = 7.8 Hz, 2H). ^{13}**C NMR** (151 MHz, $CDCl_3$) δ 137.70, 128.90, 128.71, 127.32, 112.13, 36.09, 35.19.

^{1}H NMR of 2-Phenyl-1-ethanethiol

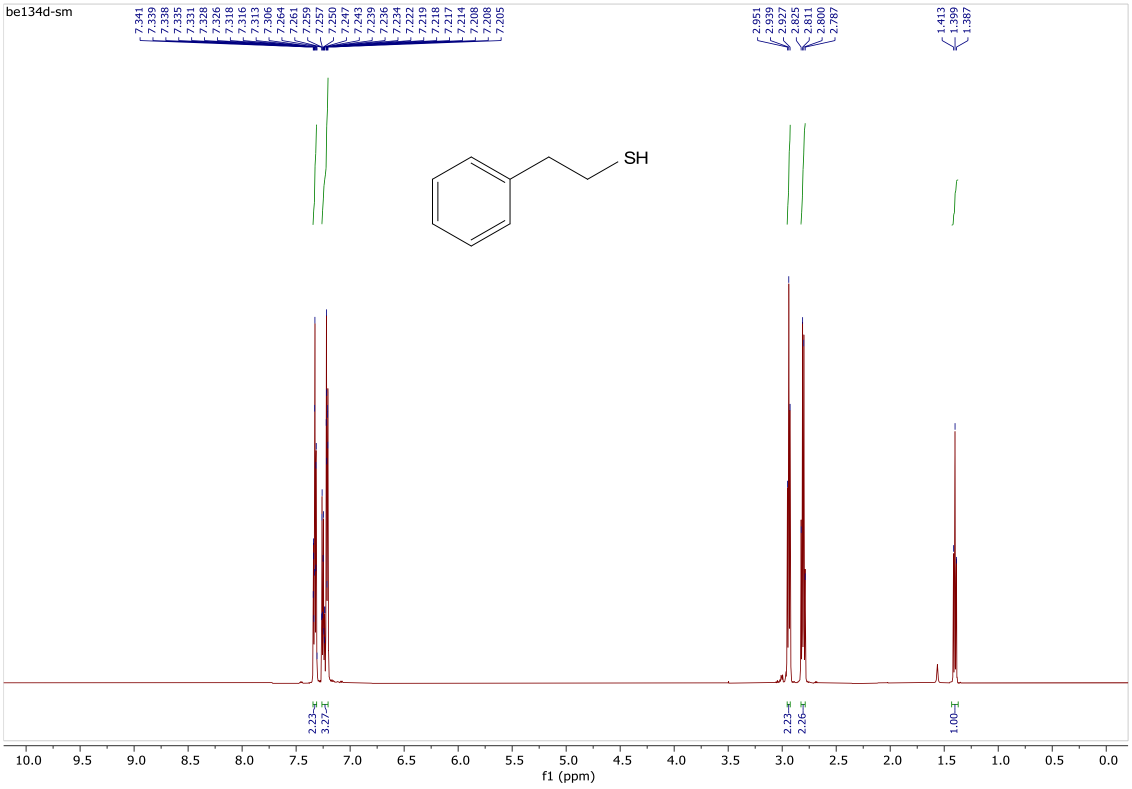

^{13}C NMR of 2-Phenyl-1-ethanethiol

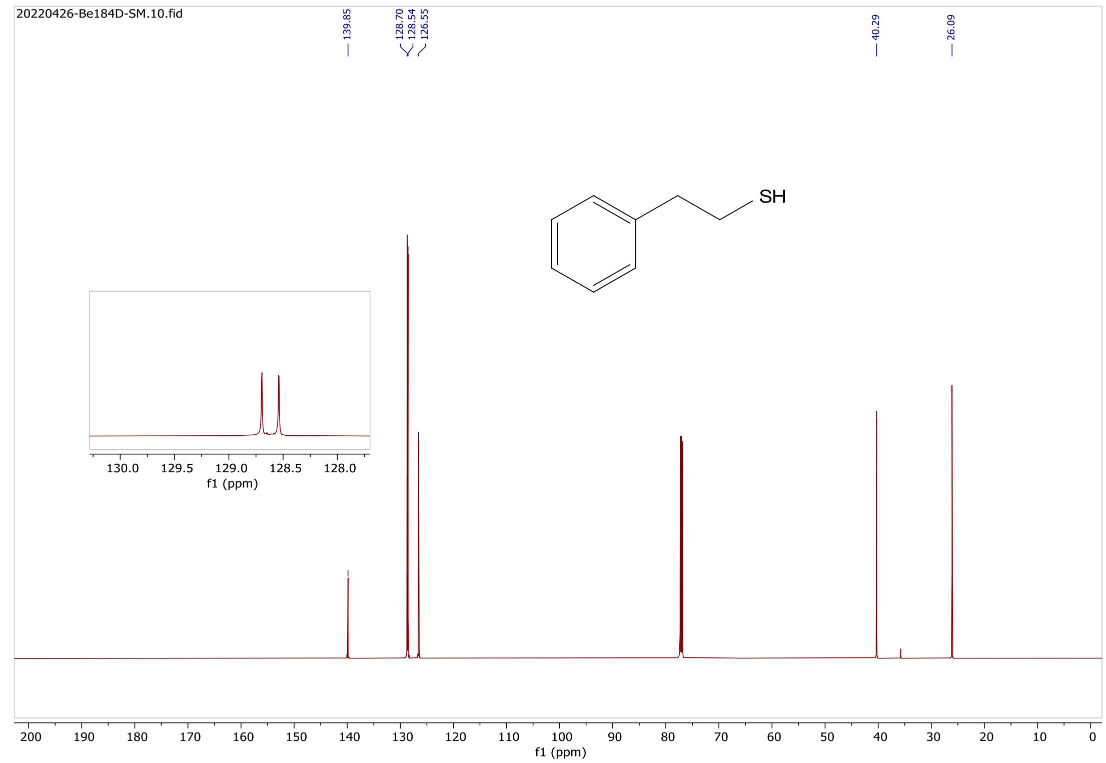

^{1}H NMR of 2-Phenylethyl thiocyanate

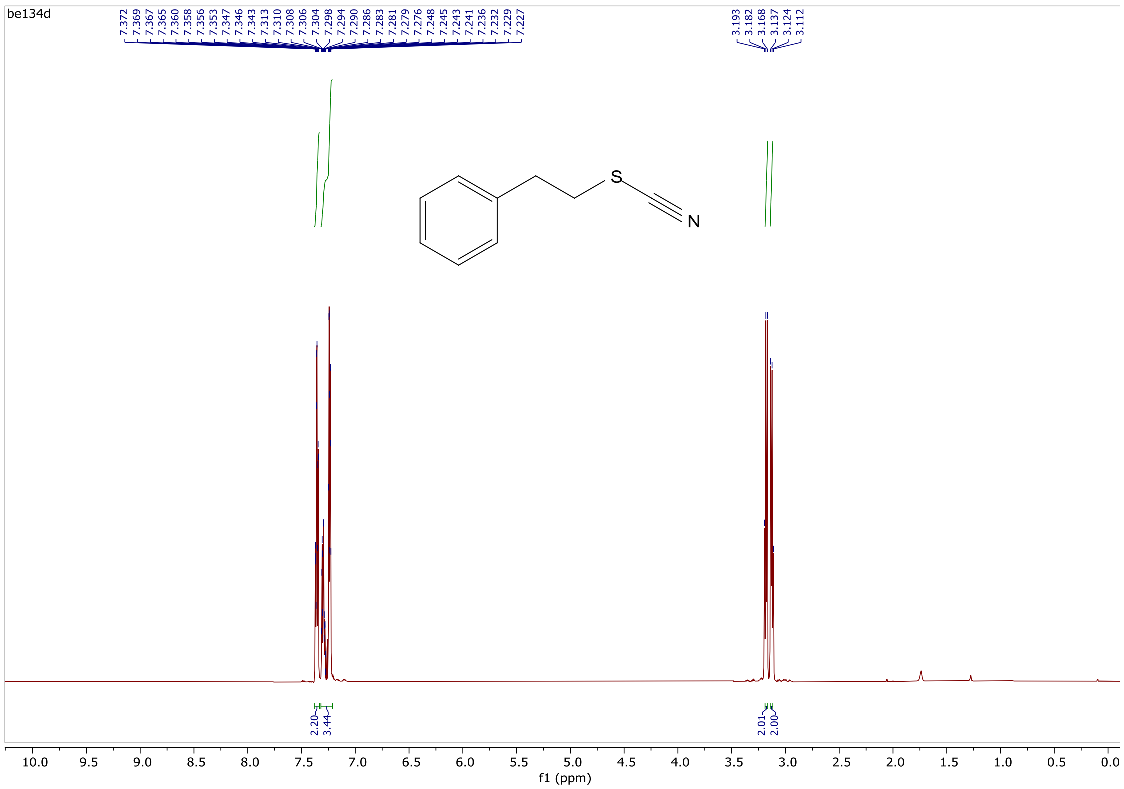

^{13}C NMR of 2-Phenylethyl thiocyanate

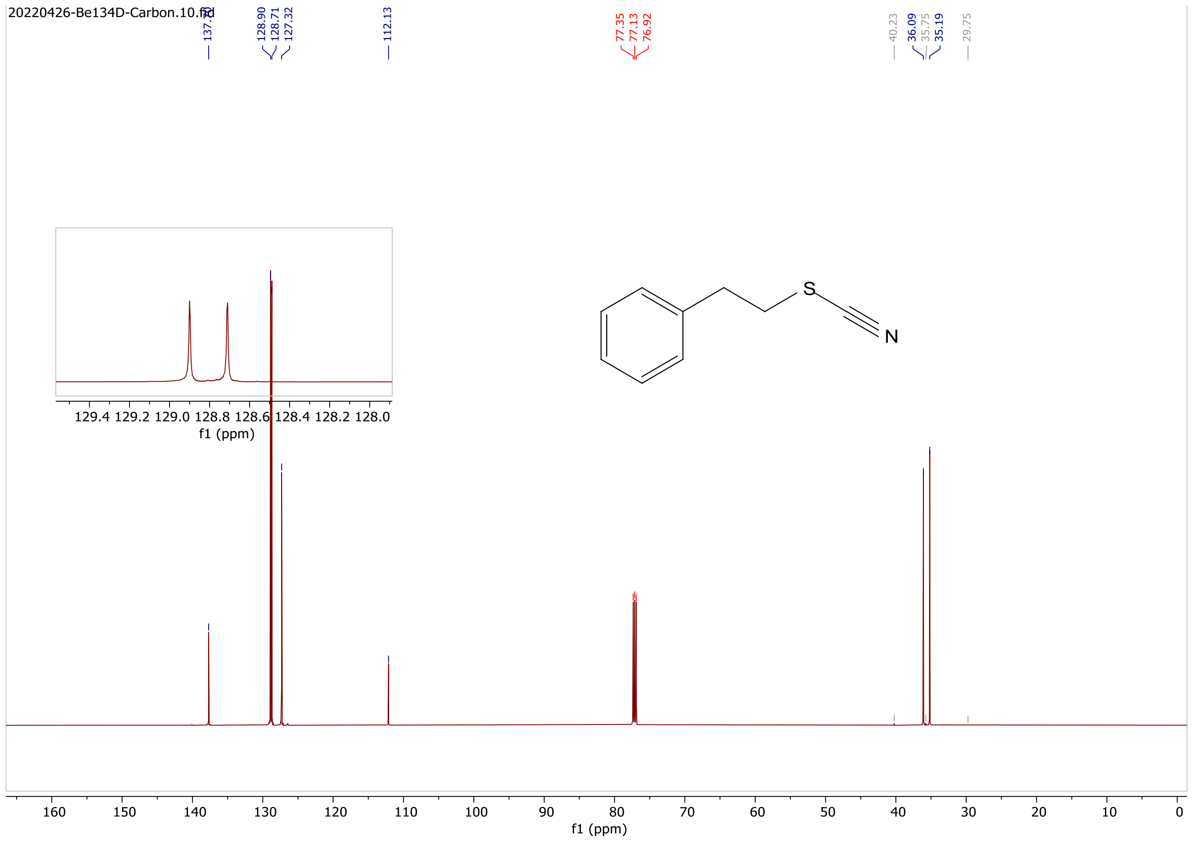

Formation of sulfoxide.

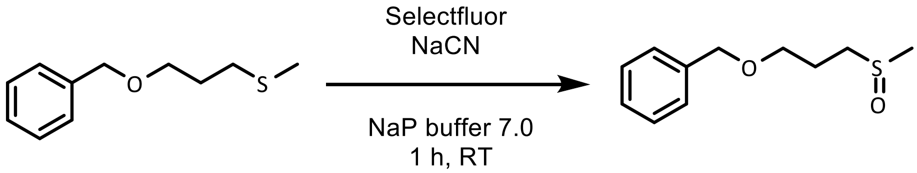

100 mg of benzene, [(3-(methylthio)propoxy]methyl] was modified using **general procedure 1.** Concentration of the mixture gave a residue, which was purified by silica gel column chromatography (eluent: hexane/EtOAc = 1:1) to sulfoxide product, benzene, [[3-[(S)-methylsulfinyl]propoxy]methyl] in 85% yield.

[[3-(methylthio)propoxy]methyl]: ^{1}**H NMR** (600 MHz, $CDCl_3$) δ 7.37 – 7.33 (m, 3H), 7.35 – 7.27 (m, 2H), 4.52 (s, 2H), 3.58 (t, J = 6.2 Hz, 2H), 2.61 (t, J = 7.2 Hz, 2H), 2.10 (s, 3H), 1.93 (p, J = 6.8 Hz, 2H).

[[3-[(S)-methylsulfinyl]propoxy]methyl]: ^{1}**H NMR** (600 MHz, $CDCl_3$) δ 7.37 – 7.30 (m, 3H), 7.30 – 7.23 (m, 2H), 4.51 (s, 2H), 3.67 – 3.56 (m, 2H), 2.88 – 2.76 (m, 2H), 2.56 (s, 3H), 2.08 (p, J = 6.9 Hz, 2H).

^{1}H NMR of [[3-(methylthio)propoxy]methyl]

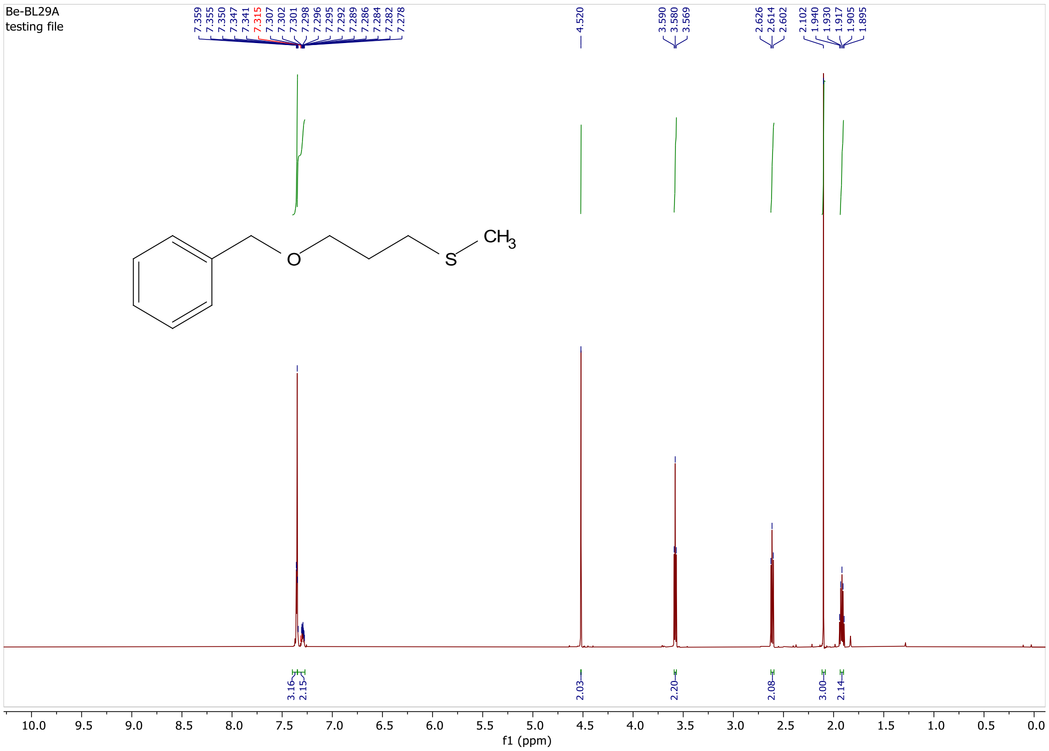

^{1}H NMR of [[3-[(S)-methylsulfinyl]propoxy]methyl]-

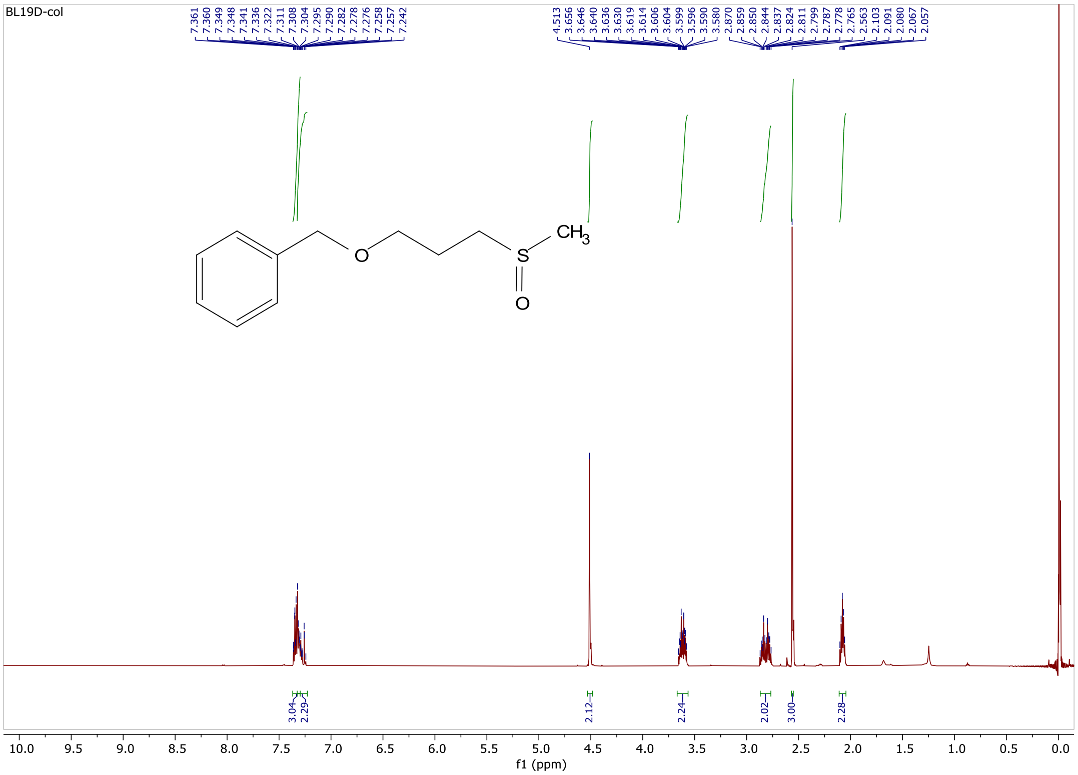

Fluorination of tryptophan.

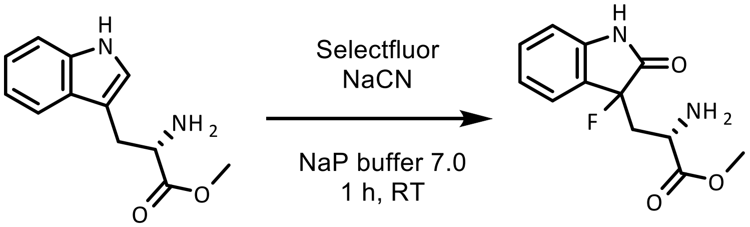

100 mg of tryptophan methylester was modified using **general procedure 2.** Concentration of the mixture gave a residue, which was purified by silica gel column chromatography (eluent: hexane/EtOAc = 5:1) to give 3-Fluoro-2-oxo-L-tryptophan methyl ester in 73% yield.

3-Fluoro-2-oxo-L-tryptophan methyl ester: ^{1}**H NMR** (600 MHz, CD_3OD) δ 7.40 – 7.33 (m, 1H), 7.33 – 7.26 (m, 1H), 7.12 (dtd, *J* = 15.1, 7.5, 1.0 Hz, 1H), 6.94 (ddt, *J* = 7.7, 5.8, 0.8 Hz, 1H), 4.69 (dd, *J* = 10.0, 2.9 Hz, 1H), 3.76 (d, *J* = 11.6 Hz, 3H), 3.03 (s, 3H), 2.59 – 2.48 (m, 1H), 2.30 – 2.21 (m, 1H). ^{13}**C NMR** (151 MHz, CD_3OD) δ 169.29, 169.12, 141.51, 130.59, 124.28, 123.93, 122.98, 110.59, 94.37, 52.43, 52.23, 36.73.

^{1}H NMR of 3-Fluoro-2-oxo-L-tryptophan methyl ester

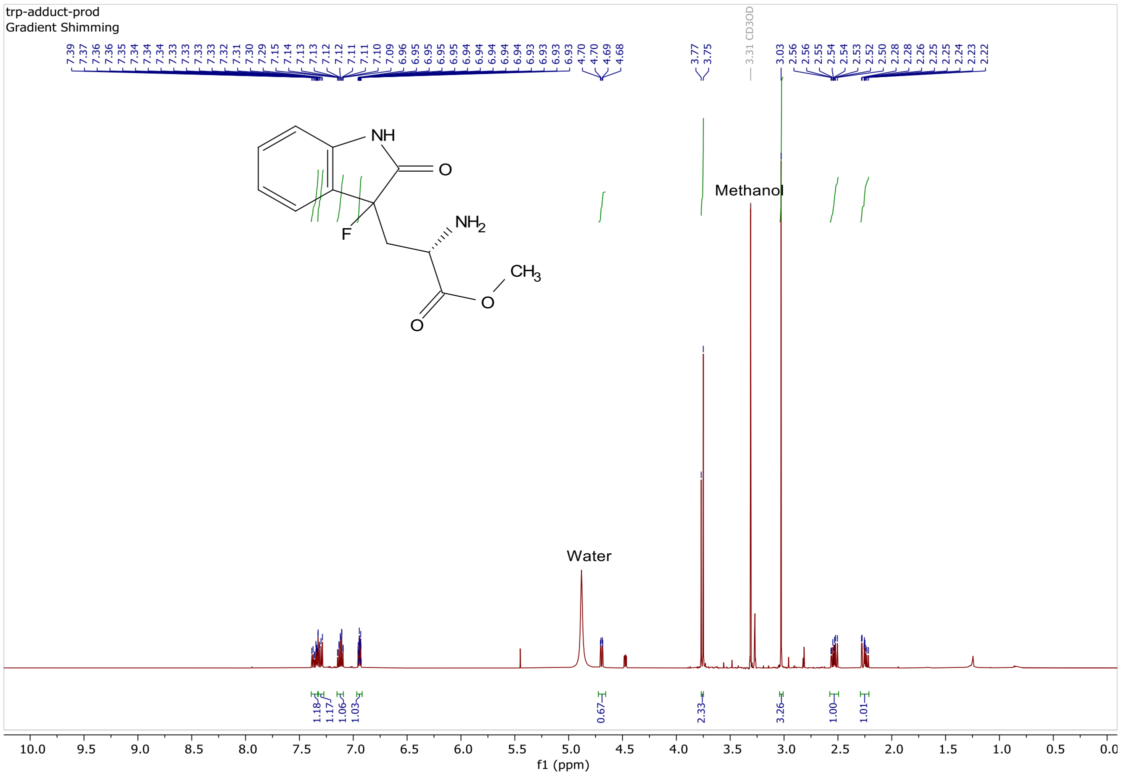

^{13}C NMR of 3-Fluoro-2-oxo-L-tryptophan methyl ester

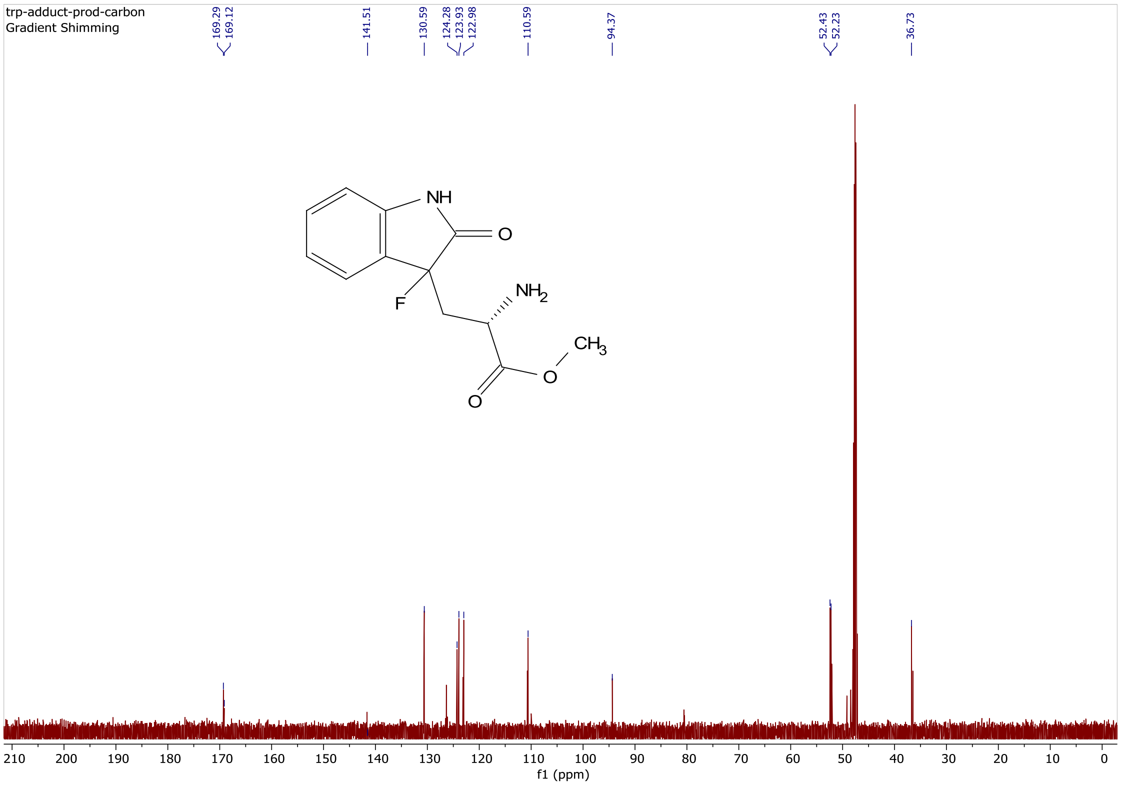

XV. Supplementary Figure 8. Development of selective enrichment strategy for nitrile modified N-terminial dimethylation.

Enrichment modification of N,N-dimethylphenylalanine methylester nitrile products **2a:**

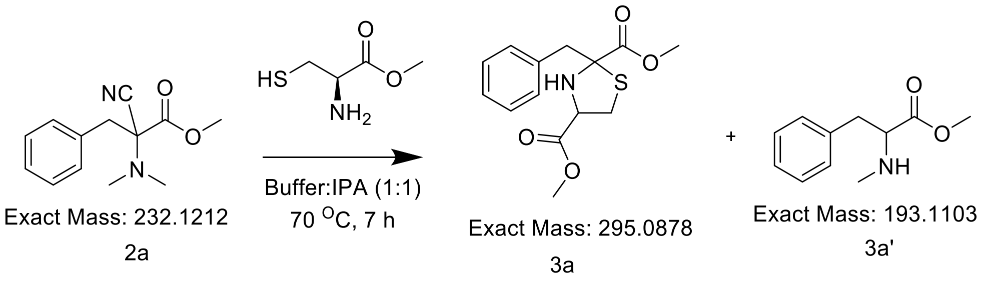

To 1.0 mg of N,N-dimethylphenylalanine methylester nitrile product **2a** dissolved in 600 µL of 10 mM sodium phosphate buffer (NaP, pH 7.0) and isopropyl alcohol (1:1), was added 3 equiv. of cysteine methylester. The reaction mixture was stirred at 70 ^{O}C for 7 h. Sample was taken from the reaction mixture, injected into LC-MS to monitor the generation of thiazolidine product **3a**. The reaction mixture was analyzed by HPLC using method A to determine the % conversion.

Thiazolidine products 3a. LCMS: *m/z* 296.0901 (calcd [M+H]+ = 296.0903) Purity: >95 % (HPLC analysis at 220 nm). Retention time in HPLC: 21.037

Thiazolidine products 3a: **^{1}H NMR** (600 MHz, $CDCl_3$) δ 7.30 (dt, *J* = 8.1, 1.7 Hz, 2H), 7.25 – 7.20 (m, 3H), 3.87 – 3.82 (m, 1H), 3.80 (s, 3H), 3.76 (s, 3H), 3.58 (s, 1H), 3.32 – 3.25 (m, 2H), 3.18 (dd, *J* = 14.1, 1.5 Hz, 1H), 2.83 (td, *J* = 10.3, 1.5 Hz, 1H). **^{13}C NMR** (151 MHz, CD_3OD) δ 173.06, 172.03, 137.96, 131.48, 129.10, 127.98, 80.29, 65.53, 54.14, 53.76, 46.62, 41.43.

Demethylated N-methyl-phenylalanine methylester 3a'. LCMS: *m/z* 193.93040 (calcd [M+H]+ = 193.1103) Purity: >95 % (HPLC analysis at 220 nm). Retention time in HPLC: 3.625

HPLC Trace of N-terminal dimethylation enrichment using cysteine methylester

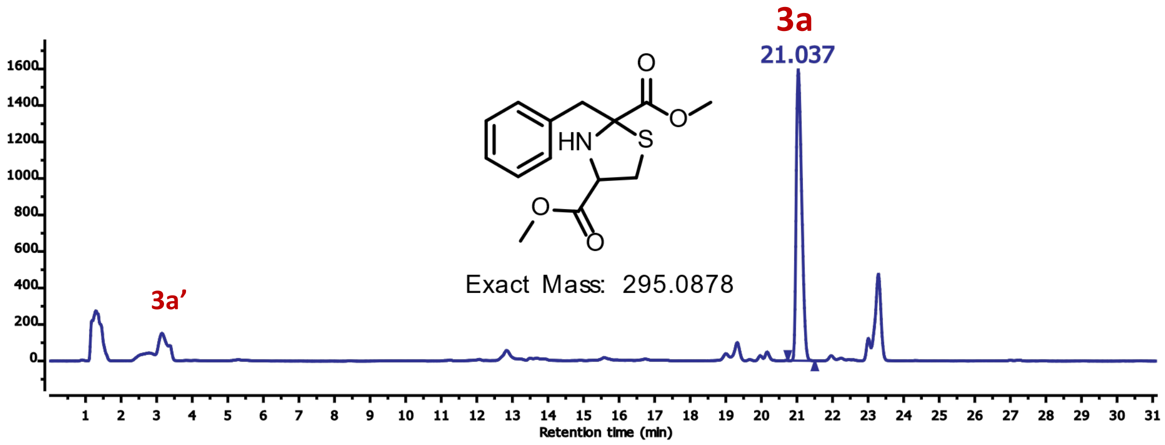

Mass spectra of peak 21.037

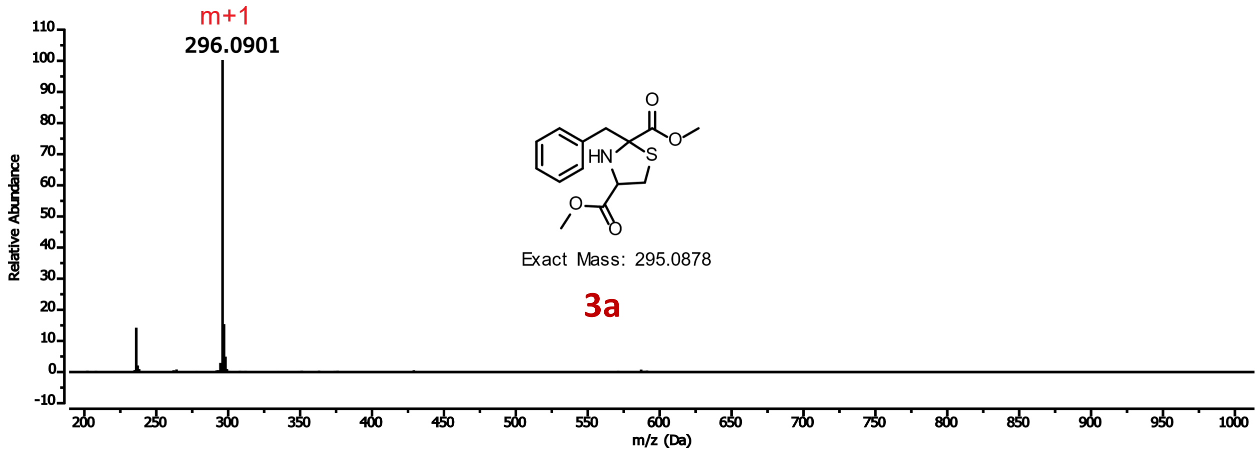

^{1}H NMR of thiazolidine product 3a

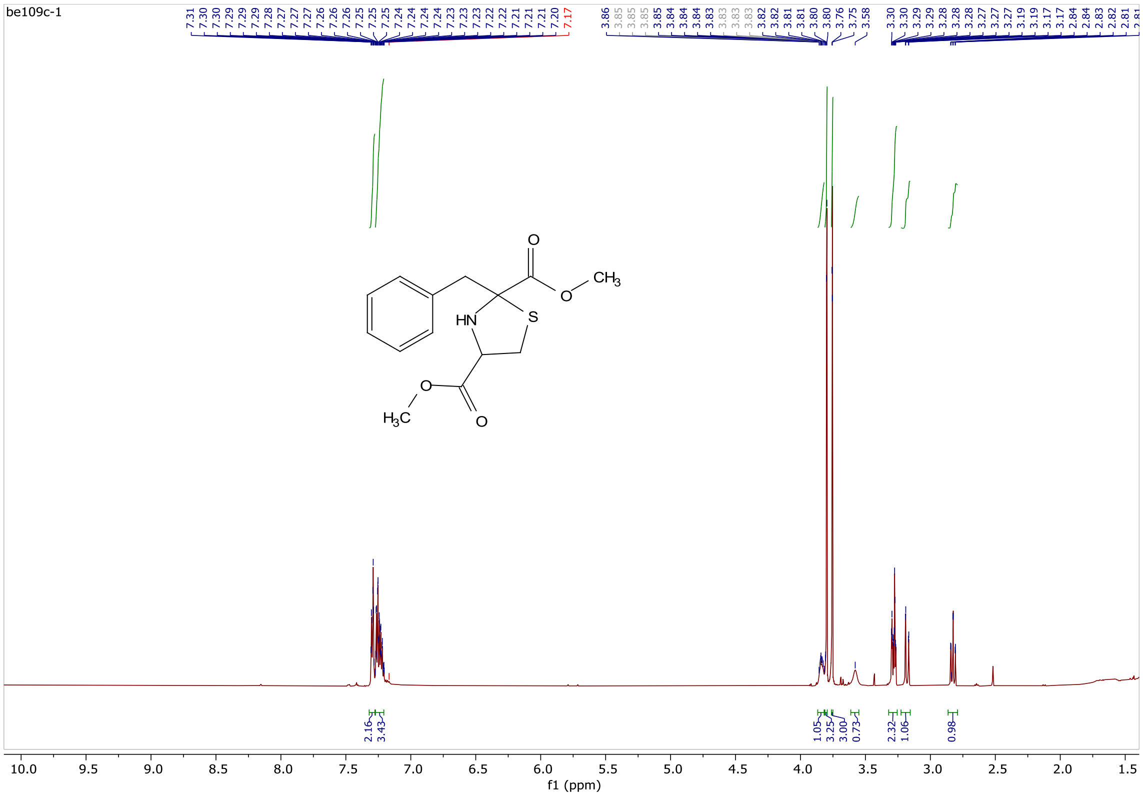

^{13}C NMR of thiazolidine product 3a

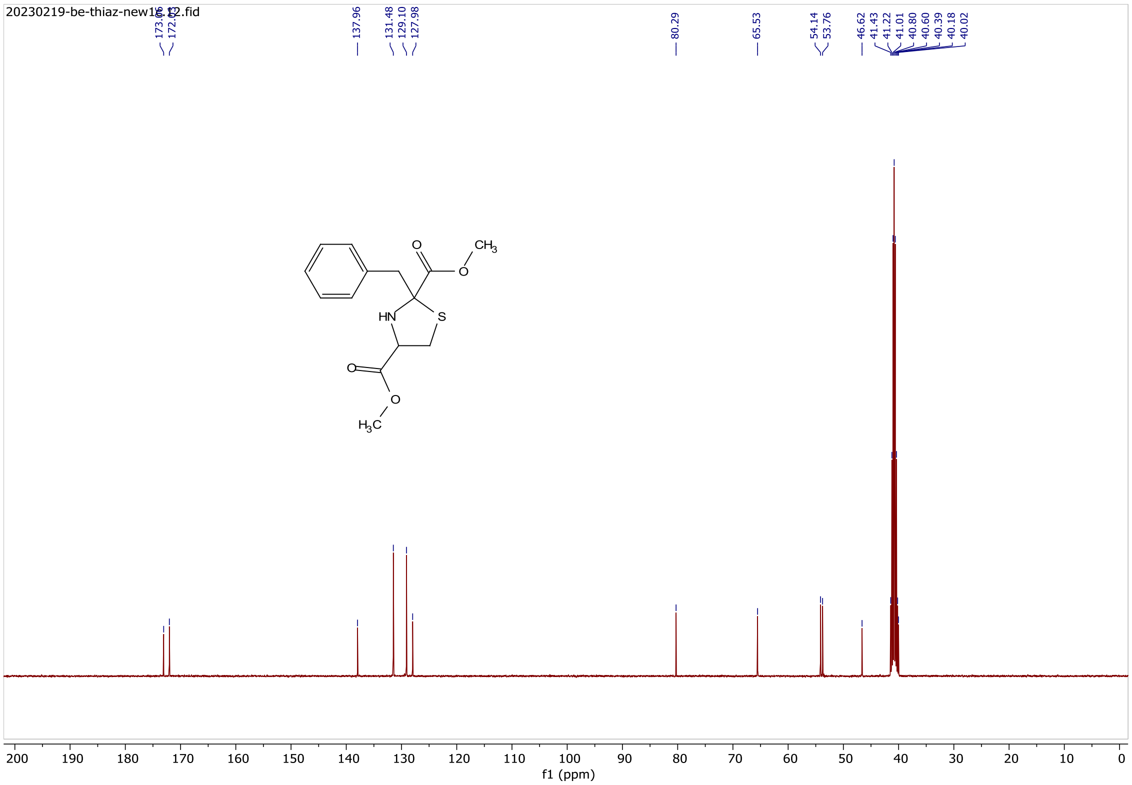

Mass spectra of peak 3a’

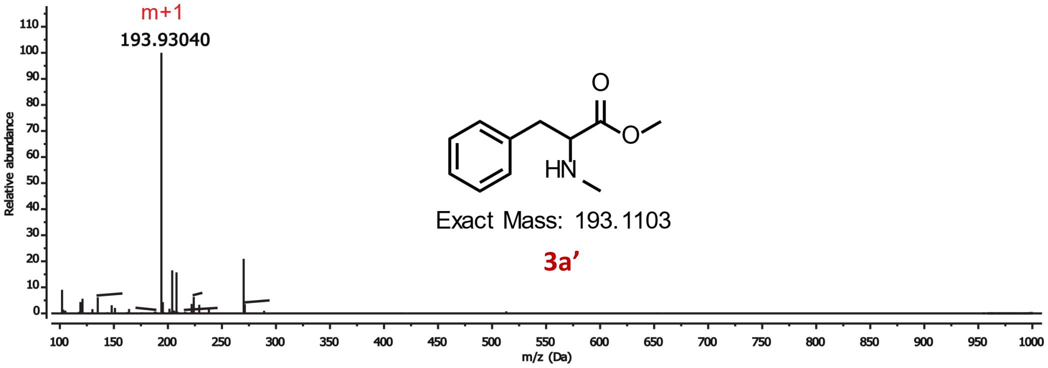

Evaluation of the role of cysteine in denitrilation of 2a’: To evaluate the role of cysteine in denitrilation of 2a’, we incubated 2a’ at room temperature and 80 ^{O}C or 24 h. No breakdown of 2a’ was observed in the absence of cysteine.

HPLC Trace of 2a and 2a’ incubation without cysteine at 80 ^{O}C for 24 h

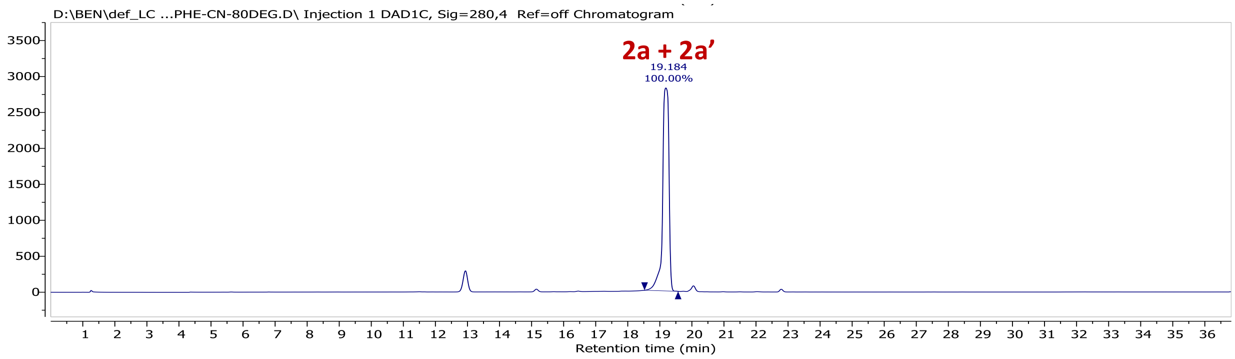

HPLC Trace of 2a and 2a’ incubation without cysteine at RT for 24 h

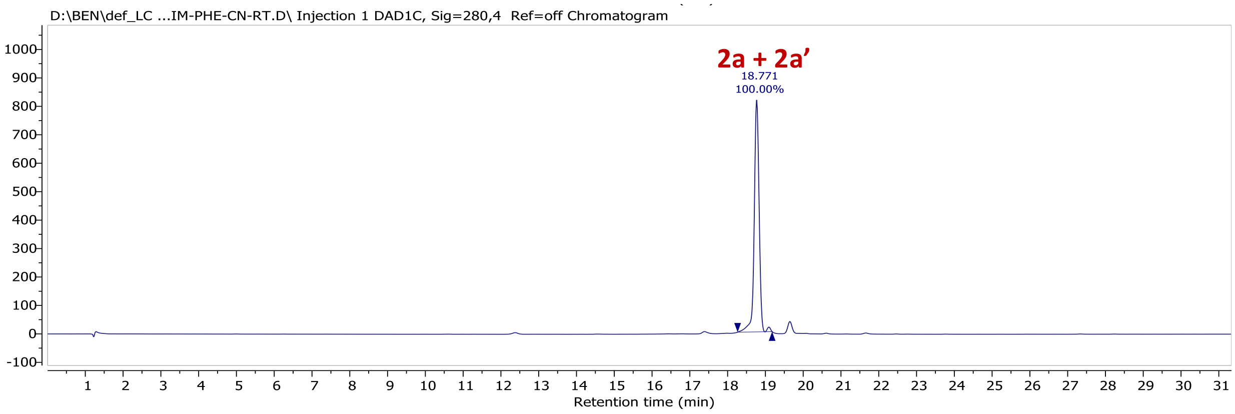

Stacked NMR of 2a' incubation without cysteine at RT or 80 °C for 24 h

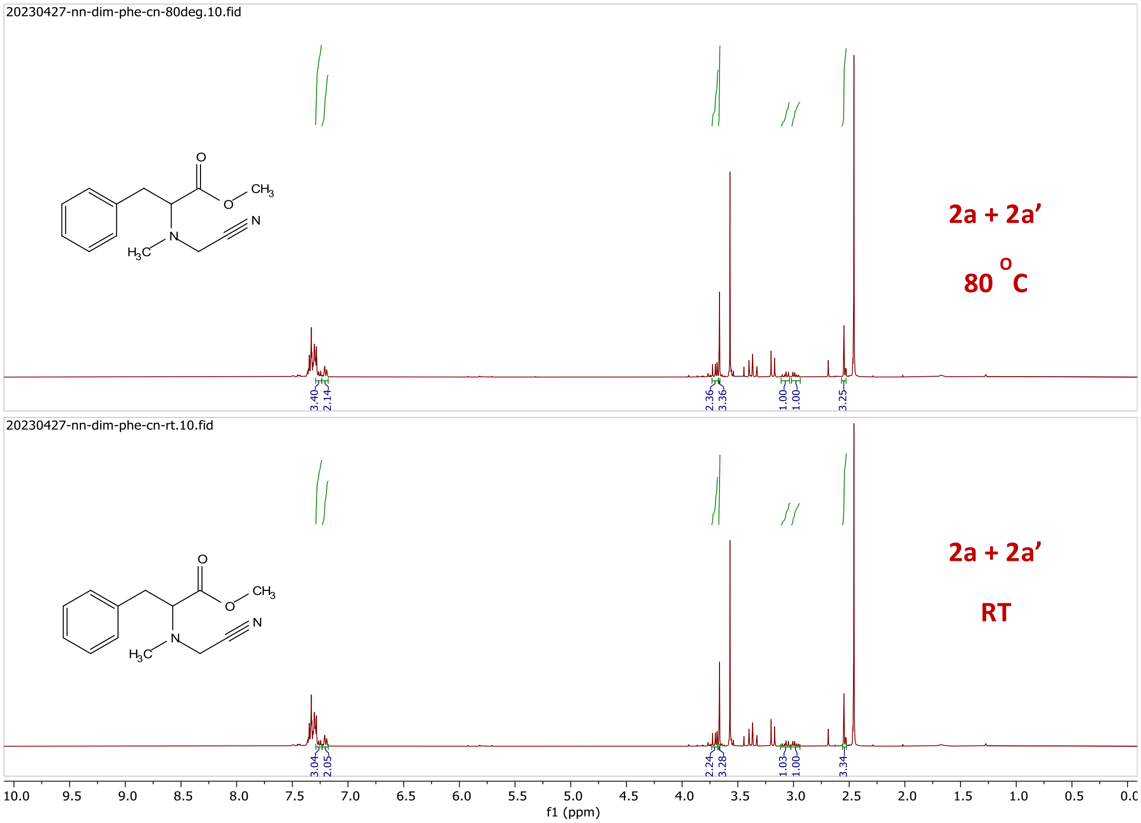

XVI. Supplementary Figure 9. Nitrilation of unprotected N,N-dimethyl peptide **1o** to **2o**.

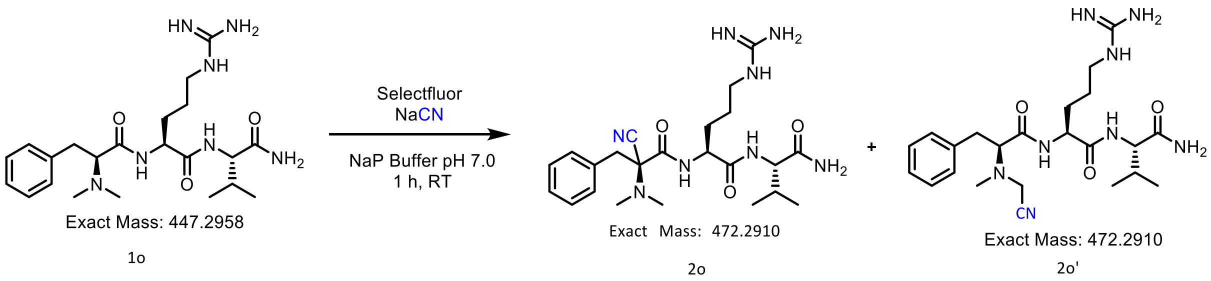

Modification of N,N-dimethyl-FRV peptide 1o: To 1.0 mg of N,N-dimethyl-FRV peptide **1o** dissolved in 300 μL of 10 mM sodium phosphate buffer (NaP, pH 7.0), was added sodium cyanide (3 equiv.) and selectfluor (2 equiv.). The reaction mixture was stirred for 1 h. Sample was taken from the reaction mixture, injected into LC-MS to monitor the generation of nitrile peptide products (2o and 2o'). The reaction mixture was analyzed by HPLC using method A to determine the % conversion. The nitrile products were observed with a conversion >98%.

N,N-dimethyl-FRV nitrile peptide products 2o and 2o'. LCMS: *m/z* 473.33687 (calcd [M+H]+ = 473.2983), *m/z* 495.31514 (calcd [M+Na]+ = 495.2803), Purity: >95 % (HPLC analysis at 220 nm). Retention time in HPLC: 8.907

HPLC Trace of reaction of 1o to 2o and 2o'

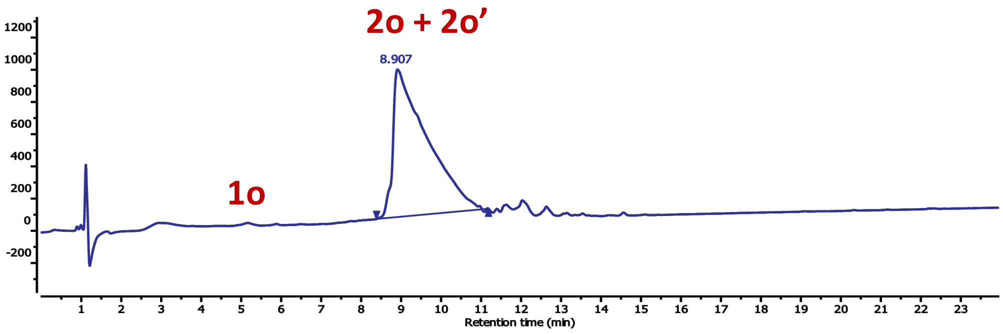

MS-Trace of nitrile peptide products 2o and 2o' (peak 8.907)

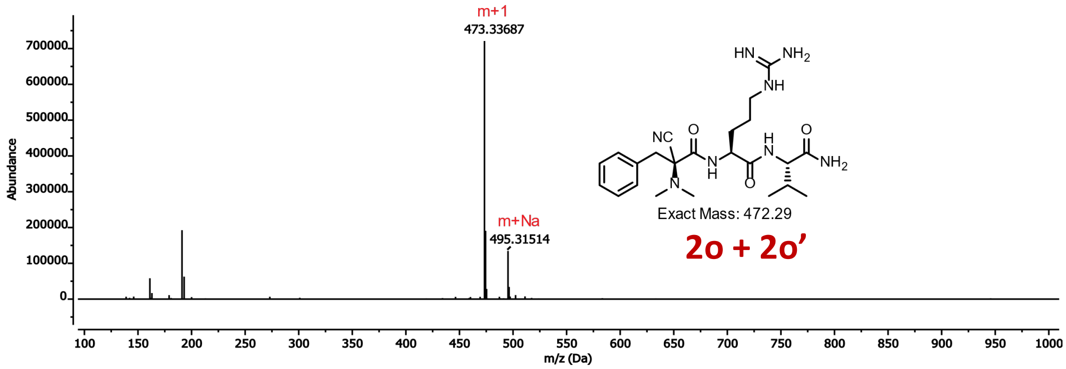

XVII. Supplementary Figure 10. Evaluation of optimized enrichment strategy on N-terminal dimethyl FRV nitrile peptide products 2o and 2o'.

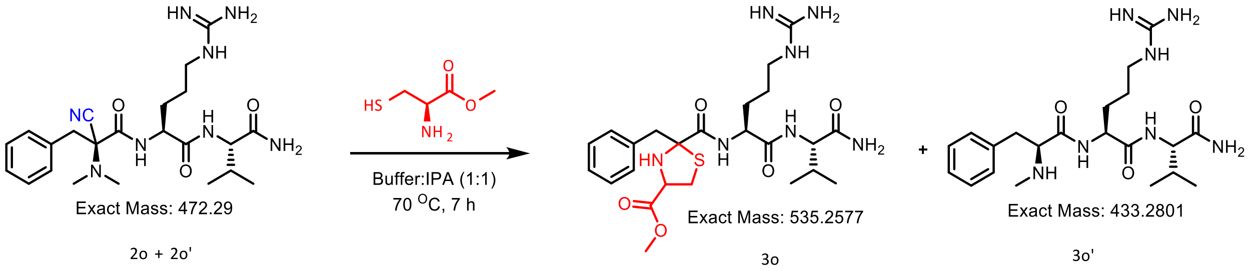

To 1.0 mg of N,N-dimethyl-FRV nitrile peptide products **2o + 2o'** dissolved in 600 µL of 10 mM sodium phosphate buffer (NaP, pH 7.0) and isopropyl alcohol (1:1), was added 3 equiv. of cysteine methylester. The reaction mixture was stirred at 70 °C for 7 h. Sample was taken from the reaction mixture, injected into LC-MS to monitor the generation of thiazolidine peptide product **3o**. The reaction mixture was analyzed by HPLC using method A to determine the % conversion.

N,N-dimethyl-FRV nitrile peptide products 2o and 2o'. LCMS: *m/z* 473.33687 (calcd [M+H]+ = 473.2983), *m/z* 495.31514 (calcd [M+Na]+ = 495.2803), Purity: >95 % (HPLC analysis at 220 nm). Retention time in HPLC: 9.026

N,N-dimethyl-FRV thiazolidine peptide products 3o. LCMS: *m/z* 536.12869 (calcd [M+H]+ = 536.2650), *m/z* 558.11058 (calcd [M+Na]+ = 558.2377), Purity: >95 % (HPLC analysis at 220 nm). Retention time in HPLC: 11.479

Demethylated N-methyl-FRV peptide products 3o'. LCMS: *m/z* 433.17622 (calcd [M+H]+ = 433.2801), *m/z* 217.62173 (calcd [M+2/2]+ = 217.6215), Purity: >95 % (HPLC analysis at 220 nm). Retention time in HPLC: 4.625

HPLC Trace of enrichment of N,N-dimethyl-FRV nitrile peptide using cysteine methyl ester

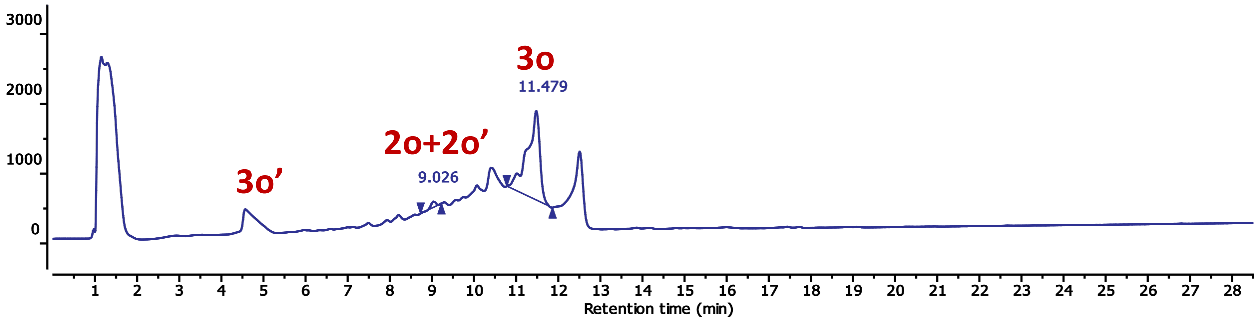

Mass spectra of peak 9.026

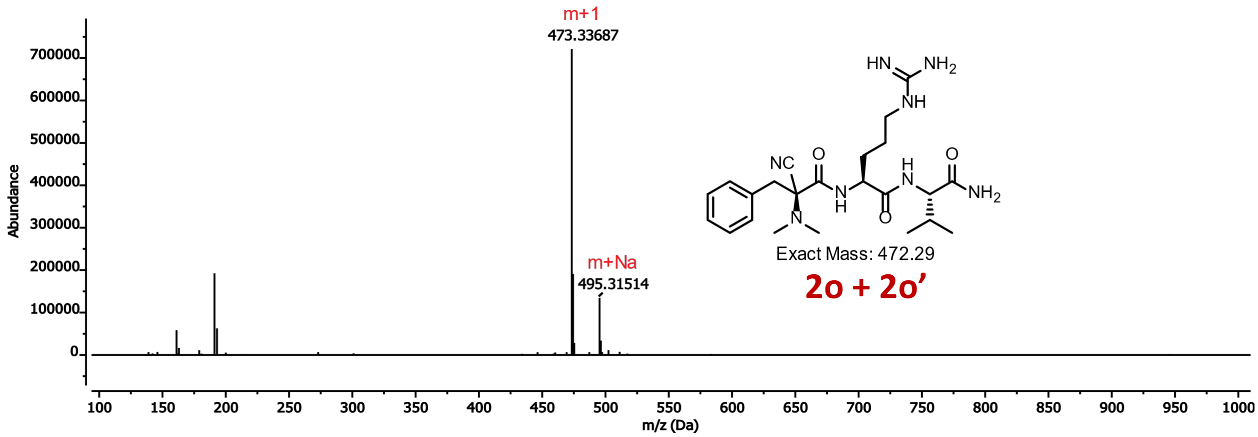

Mass spectra of peak 11.479

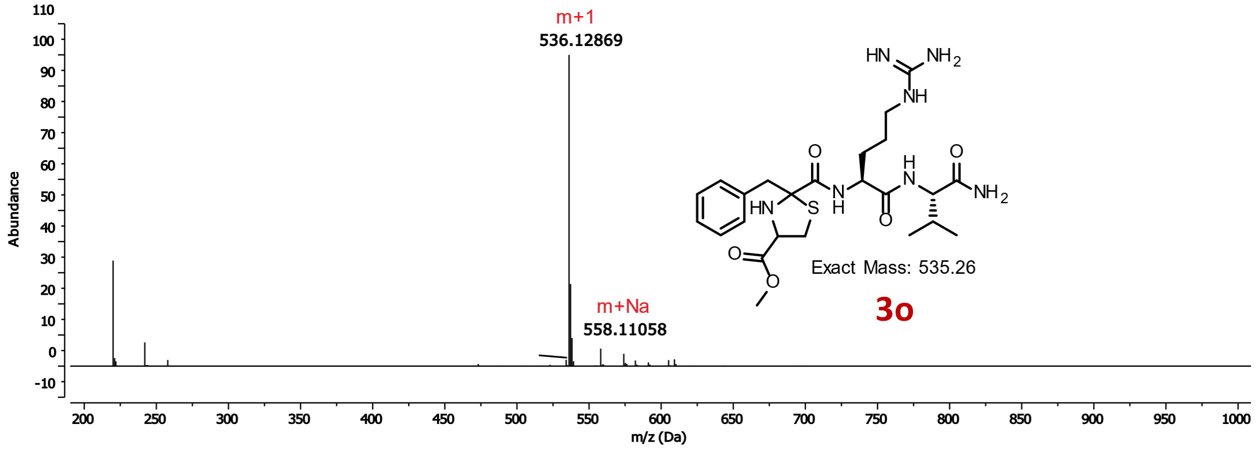

Mass spectra of peak 4.625 (3o')

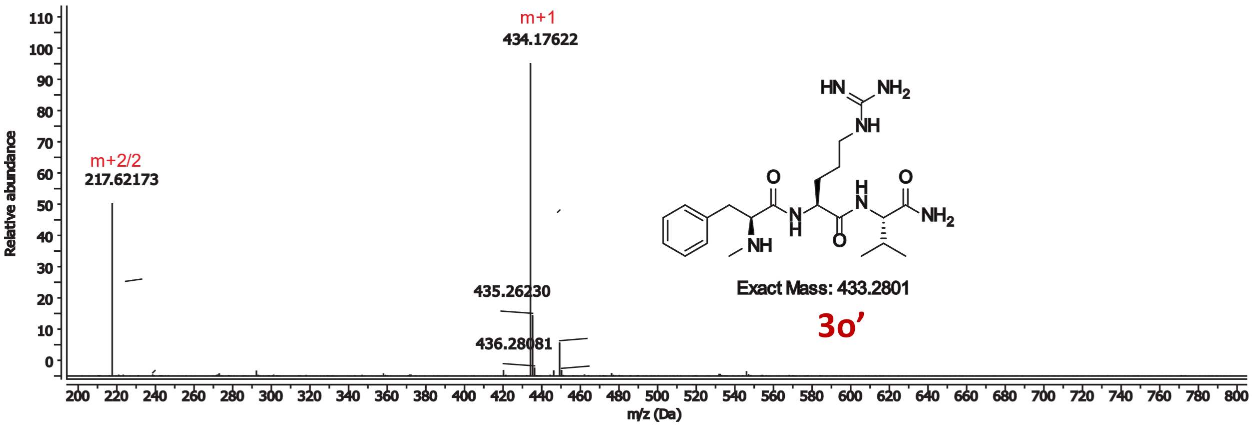

XVIII. Supplementary Figure 11. Nitrilation of unprotected dimethyllysine peptide 1m to generate nitrile peptide product 2m.

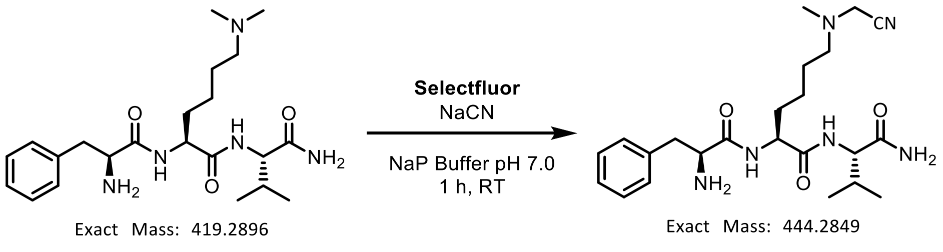

Modification of FKme$_2$V peptide 1m: To 1.0 mg of FKme$_2$V peptide **1m** dissolved in 300 µL of 10 mM sodium phosphate buffer (NaP, pH 7.0), was added sodium cyanide (3 equiv.) and selectfluor (2 equiv.). The reaction mixture was stirred for 1 h. Sample was taken from the reaction mixture, injected into LC-MS to monitor the generation of nitrile peptide product **2m**. The reaction mixture was analyzed by HPLC using method A to determine the % conversion.

FKme$_2$V peptide 1m. LCMS: *m/z* 420.29315 (calcd $[M+H]^+$ = 420.2969), (HPLC analysis at 220 nm). Retention time in HPLC: 5.228

FKme$_2$(CN)V nitrile-peptide 2m. LCMS: *m/z* 445.18156 (calcd $[M+H]^+$ = 445.2922), *m/z* 223.15009 (calcd $[M+2/2]^+$ = 223.1424), *m/z* 467.12659 (calcd $[M+Na]^+$ = 467.2747), *m/z* 889.41710 (calcd $[2M+H]^+$ = 889.5771), *m/z* 911.42847 $[2m+Na]^+$ =911.5595. (HPLC analysis at 220 nm). Retention time in HPLC: 6.275

HPLC Trace of the reaction with dimethyllysine peptide 1m

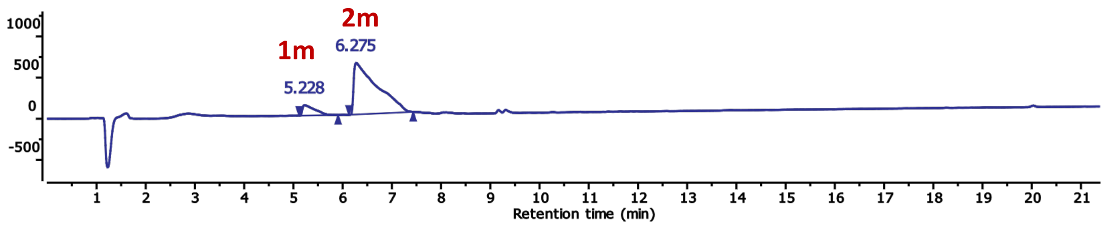

MS-Trace of 1m (peak 5.228)

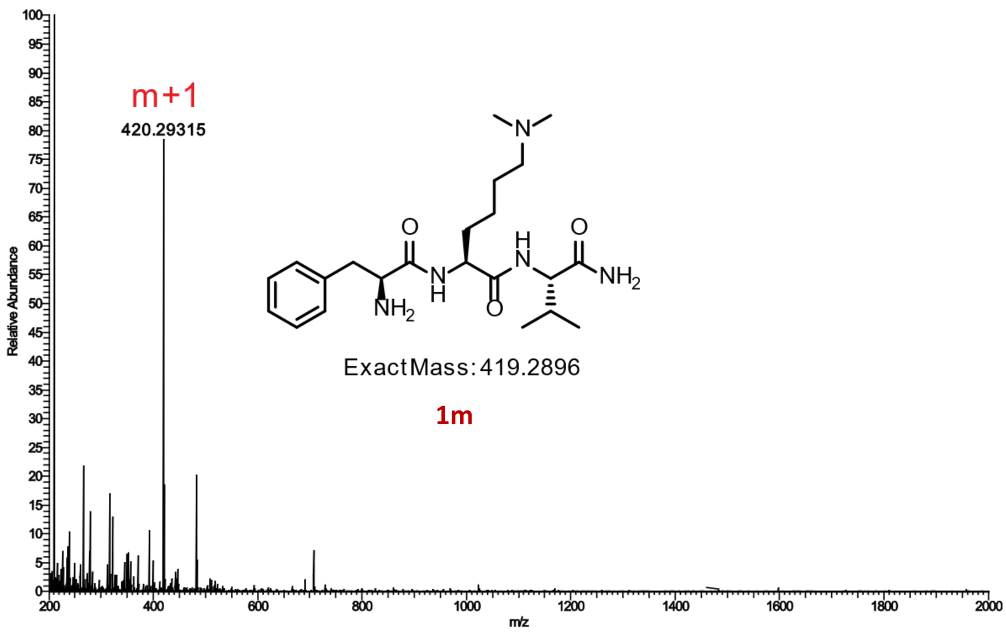

MS-Trace of 2m (peak 6.275)

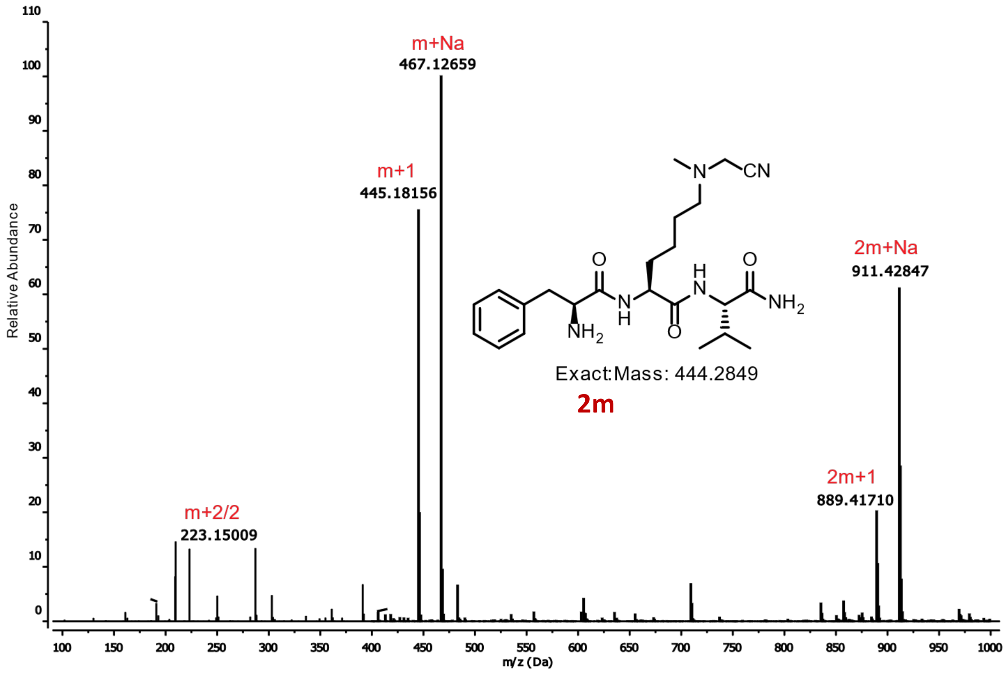

XIX. Supplementary Figure 12. Enrichment of nitrile modified dimethyllysine peptide 2m.

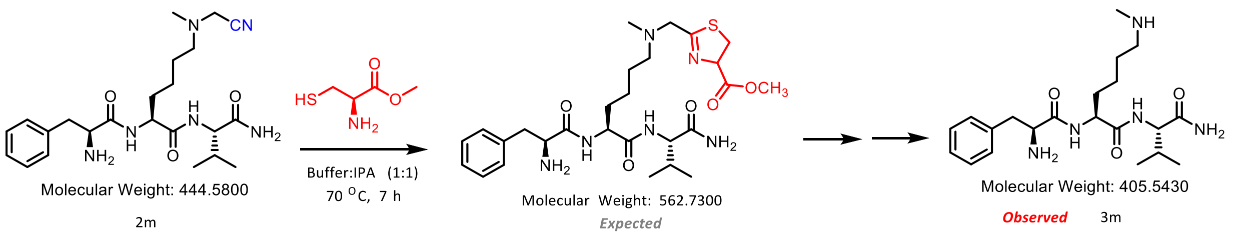

To 1.0 mg of FKme$_2$(CN)V nitrile peptide **2m** dissolved in 600 μL of 10 mM sodium phosphate buffer (NaP, pH 7.0) and isopropyl alcohol (1:1), was added 3 equiv. of cysteine methylester. The reaction mixture was stirred at 70 ^{O}C for 7 h. Sample was taken from the reaction mixture, injected into LC-MS to monitor the generation of demethylation peptide product **3m**. The reaction mixture was analyzed by HPLC using method A to determine the % conversion.

FKme$_2$(CN)V nitrile peptide 2m. LCMS: *m/z* 445.18156 (calcd $[M+H]^+$ = 445.2922), *m/z* 223.15009 (calcd $[M+2/2]^+$ = 223.1424), *m/z* 467.12659 (calcd $[M+Na]^+$ = 467.2747), *m/z* 889.41710 (calcd $[2M+H]^+$ = 889.5771), *m/z* 911.42847 $[2M+Na]^+$ =911.5595. (HPLC analysis at 220 nm). Retention time in HPLC: 9.027

FKme$_1$V monomethyllysine peptide 3m. LCMS: *m/z* 406.29870 (calcd $[M+H]^+$ = 406.2813, *m/z* 811.59185 (calcd $[2M+H]^+$ = 811.5553), (HPLC analysis at 220 nm). Retention time in HPLC: 7.982

HPLC trace of enrichment of dimethyllysine using cysteine methyl ester

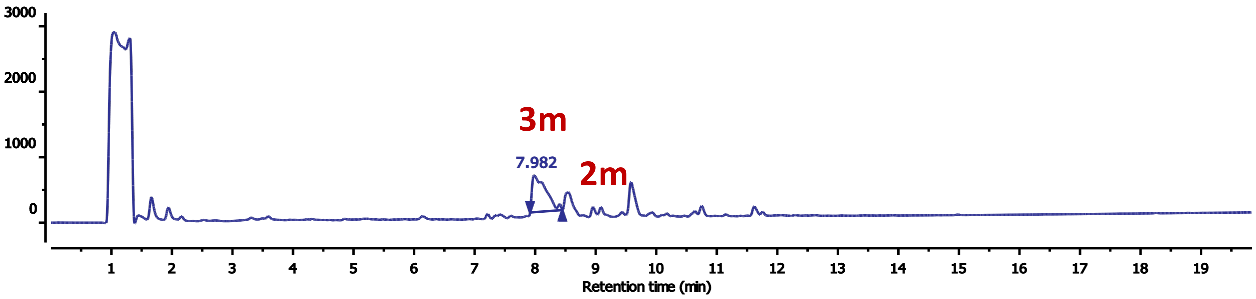

Mass spectra of peak 7.982

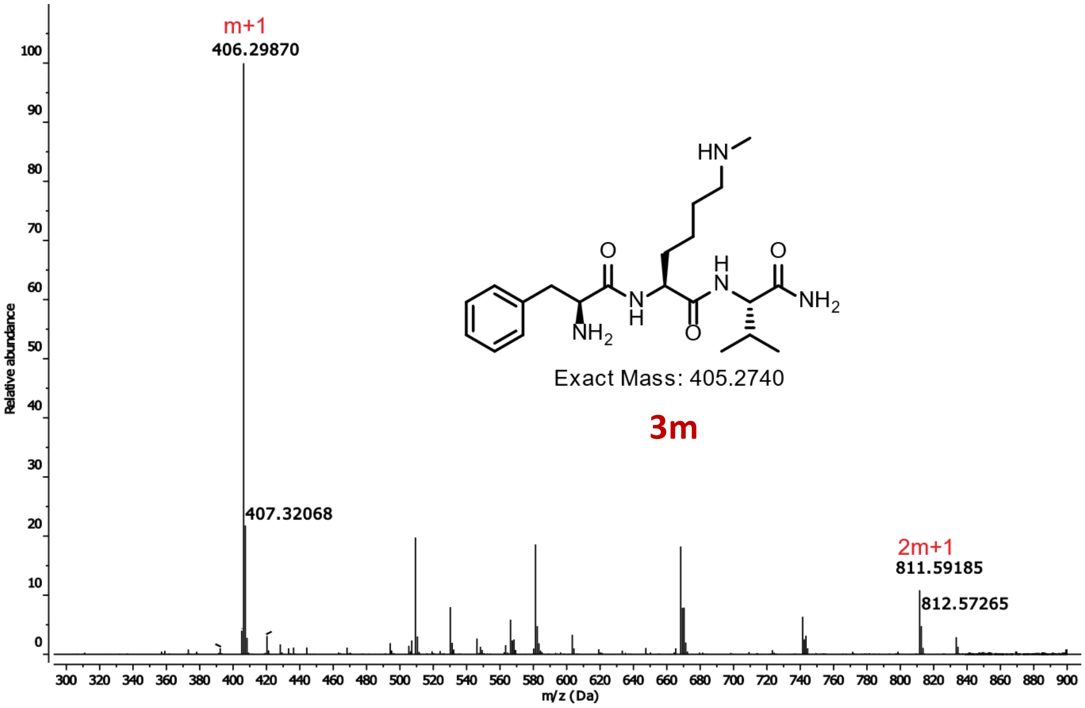

XX. Supplementary Figure 13. Plausible mechanism for the reaction of nitrile modified dimethyllysine peptide with cysteine methylester.

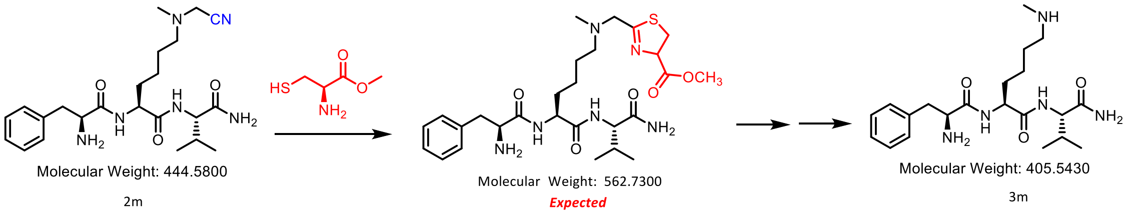

PLAUSIBLE MECHANISM FOR MONOMETHYL GENERATION

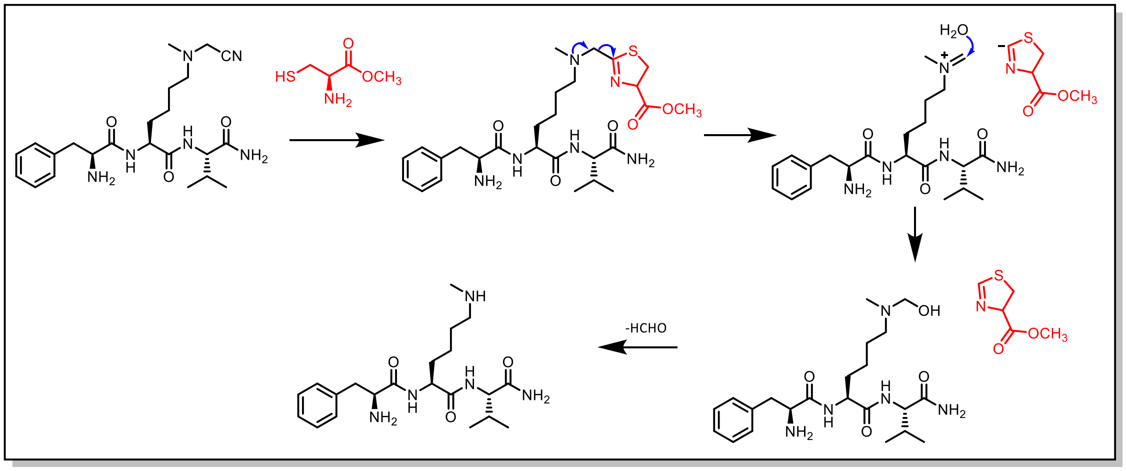

XXI. Supplementary Figure 14. Reaction of nitrile modified cysteine containing thiocyanate peptide with cysteine methylester.

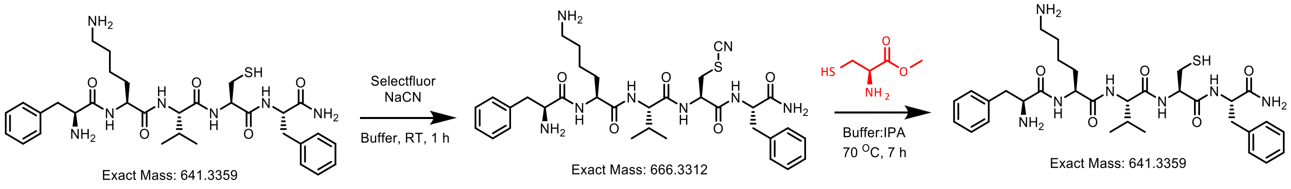

1q 2q 1q

Modification of cysteine containing peptide 1q to nitrile product 2q: To 1.0 mg of cysteine peptide **1q** dissolved in 300 µL of 10 mM sodium phosphate buffer (NaP, pH 7.0), was added sodium cyanide (3 equiv.) and selectfluor (2 equiv.). The reaction mixture was stirred for 1 h. Sample was taken from the reaction mixture, injected into LC-MS to monitor the generation of thiocyanate peptide product **2q**. The reaction mixture was analyzed by HPLC using method A to determine the % conversion. The nitrile product **2q** was observed with a conversion >98%

FKVCF peptide 1q. LCMS: *m/z* 642.33236 (calcd $[M+H]^+$ = 642.3432), *m/z* 664.30374 (calcd $[M+Na]^+$ = 664.3053), *m/z* 321.72346 (calcd $[M+2/2]^+$ = 321.7232) (HPLC analysis at 220 nm). Retention time in HPLC: 8.116

FKVCF peptide thiocyanate products 2q. LCMS: *m/z* 667.17902 (calcd $[M+H]^+$ = 667.1863), *m/z* 689.14639 (calcd $[M+Na]^+$ = 689.1458), *m/z* 705.14915 (calcd $[M+K]^+$ = 705.1489), *m/z* 334.15865 (calcd $[M+2/2]^+$ = 334.1615) (HPLC analysis at 220 nm). Retention time in HPLC: 8.978

HPLC trace of thiocyanate enrichment using cysteine methyl ester

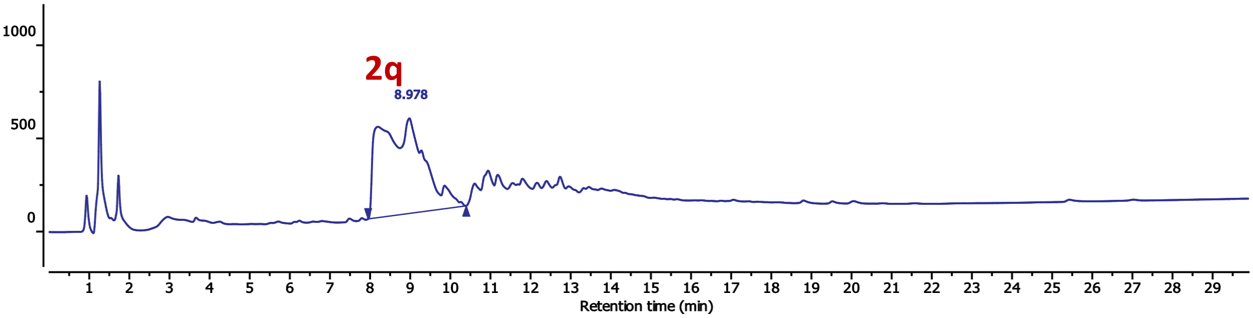

Mass spectra of peak 8.978 (2q)

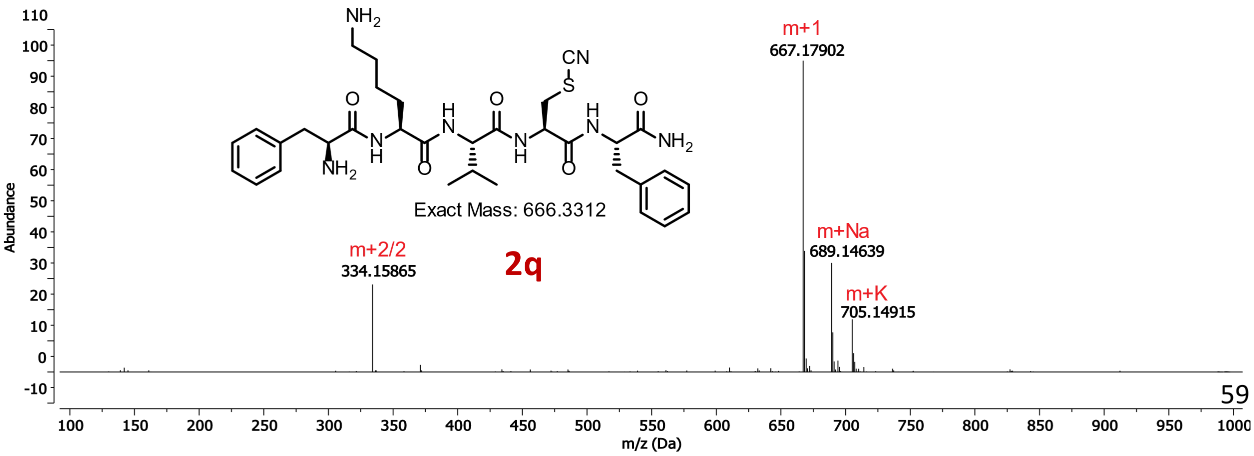

Modification of FKVCF thiocyanate 2q to peptide 1q:

To 1.0 mg of FKVC(CN)F nitrile peptide product **2q** dissolved in 600 µL of 10 mM sodium phosphate buffer (NaP, pH 7.0) and isopropyl alcohol (1:1), was added 3 equiv. of cysteine methylester. The reaction mixture was stirred at 70 ^{O}C for 7 h. Sample was taken from the reaction mixture, injected into LC-MS to monitor the generation of denitrilation peptide product **1q**.

FKVSF peptide 1q. LCMS: *m/z* 642.33236 (calcd $[M+H]^+$ = 642.3432), *m/z* 664.30374 (calcd $[M+Na]^+$ = 664.3053), *m/z* 321.72346 (calcd $[M+2/2]^+$ = 321.7232) (HPLC analysis at 220 nm). Retention time in HPLC: 8.116

FKVSF peptide thiocyanate product 2q. LCMS: *m/z* 667.17902 (calcd $[M+H]^+$ = 667.1863), *m/z* 689.14639 (calcd $[M+Na]^+$ = 689.1458), *m/z* 705.14915 (calcd $[M+K]^+$ = 705.1489), *m/z* 334.15865 (calcd $[M+2/2]^+$ = 334.1615) (HPLC analysis at 220 nm). Retention time in HPLC: 8.978

HPLC Trace of thiocyanate reversal using cysteine methyl ester

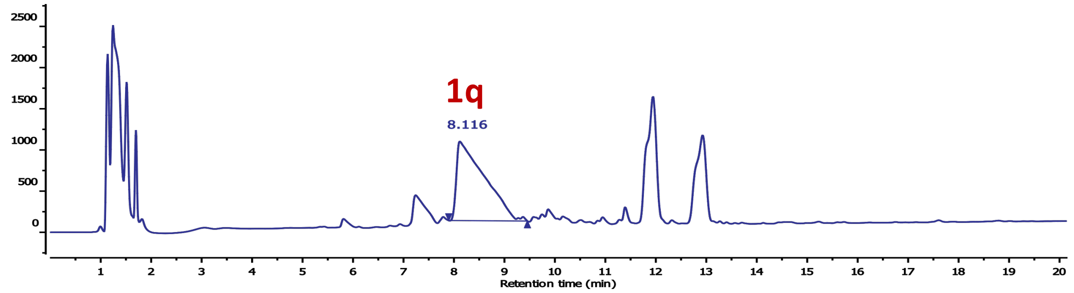

Mass spectra of peak 8.116 (1q)

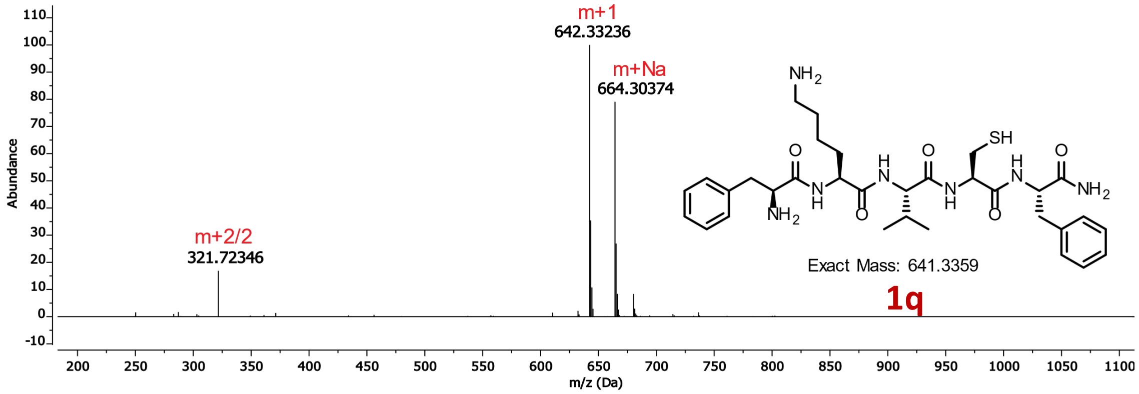

XXII. Supplementary Figure 15. Plausible mechanism for the enrichment of modified cysteine (thiocyanate) residues with cysteine methylester.

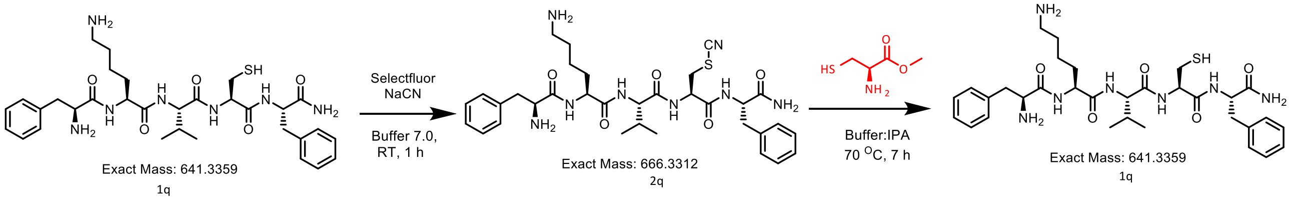

PLAUSIBLE MECHANISM FOR THIOCYANATE REVERSAL

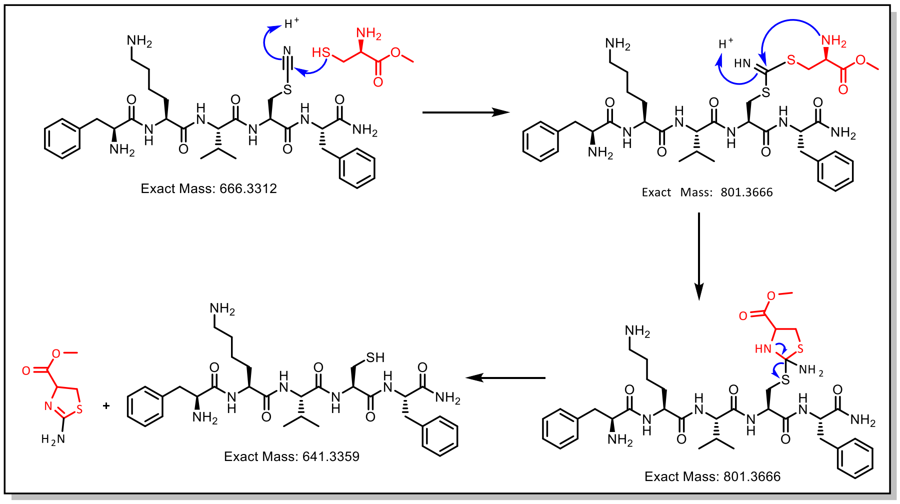

XXIII. Supplementary Figure 16. Differential enrichment of modified N-terminal dimethylation over modified dimethyllysine peptide.

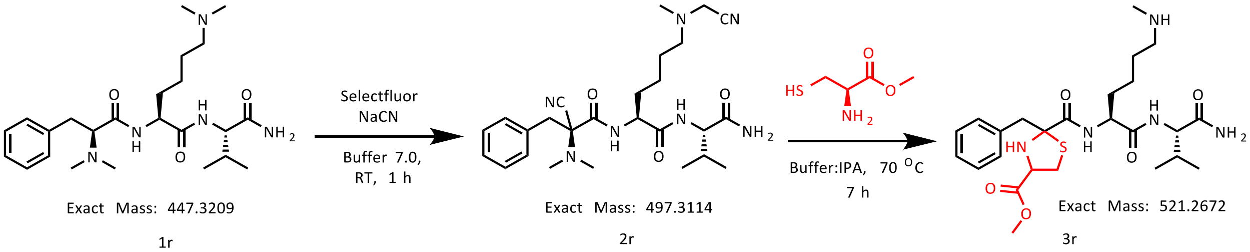

1r 2r 3r

To 1.0 mg of dual dimethylation peptide **1r** dissolved in 300 µL of 10 mM sodium phosphate buffer (NaP, pH 7.0), was added sodium cyanide (3 equiv.) and selectfluor (2 equiv.). The reaction mixture was stirred for 1 h. Sample was taken from the reaction mixture, injected into LC-MS to monitor the generation of di-nitrile peptide product **2r**. The reaction mixture was analyzed by HPLC using method A to determine the % conversion. The di-nitrile product **2r** was observed with a conversion >98%

Di-nitrile modified peptide product 2r. LCMS: *m/z* 498.38303 (calcd $[M+H]^+$ = 498.3187), *m/z* 520.36316 (calcd $[M+Na]^+$ = 520.3114), *m/z* 536.35834 (calcd $[M+K]^+$ = 536.4114), (HPLC analysis at 220 nm). Retention time in HPLC: 13.576

HPLC Trace of di-nitrile modification

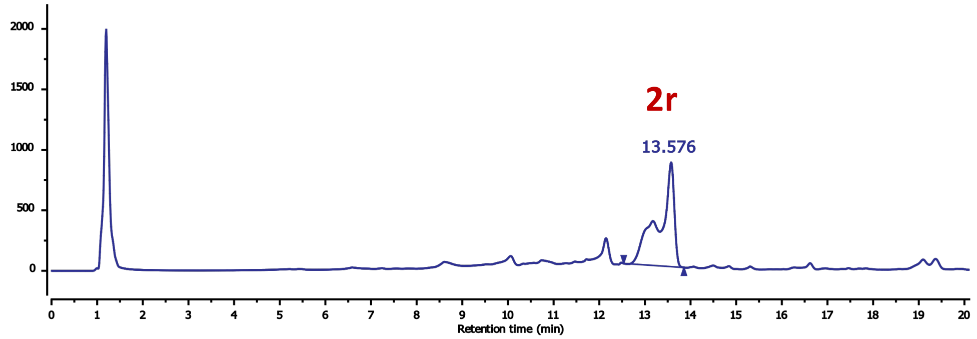

Mass spectra of peak 13.576 (2r)

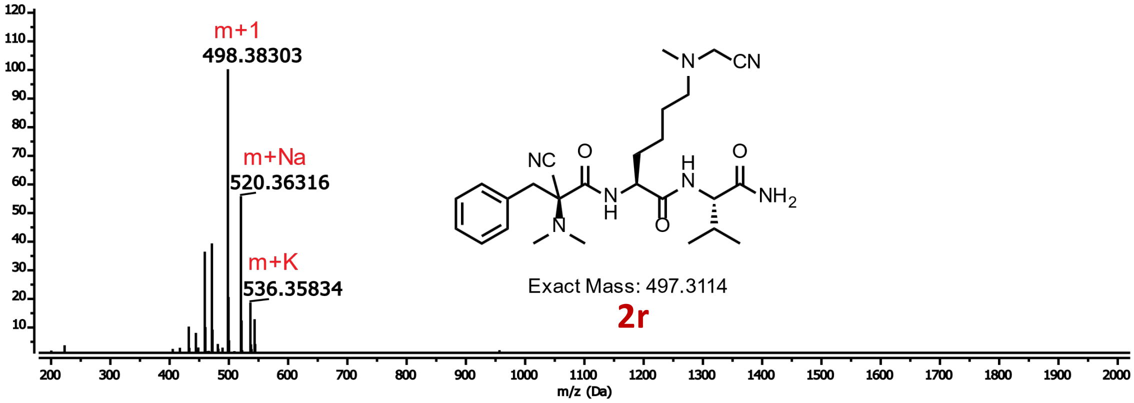

To 1.0 mg of di-nitrile modified peptide **2r** dissolved in 600 μL of 10 mM sodium phosphate buffer (NaP, pH 7.0) and isopropyl alcohol (1:1), was added 3 equiv. of cysteine methylester. The reaction mixture was stirred at 70 ^{O}C for 7 h. Sample was taken from the reaction mixture, injected into LC-MS to monitor the generation of peptide products **3r**. The reaction mixture was analyzed by HPLC using method A to determine the % conversion to the thiazolidine modified peptide **3r**.

Thiazolidine-peptide product 3r. LCMS: *m/z* 522.32766 (calcd $[M+H]^+$ = 522.3349), *m/z* 1043.67951 (calcd $[2M+H]^+$ = 1043.5417), *m/z* 1065.68794 $[2m+Na]^+$ =1065.5344. (HPLC analysis at 220 nm). Retention time in HPLC: 12.175

HPLC Trace of thiazolidination modification using cysteine methyl ester

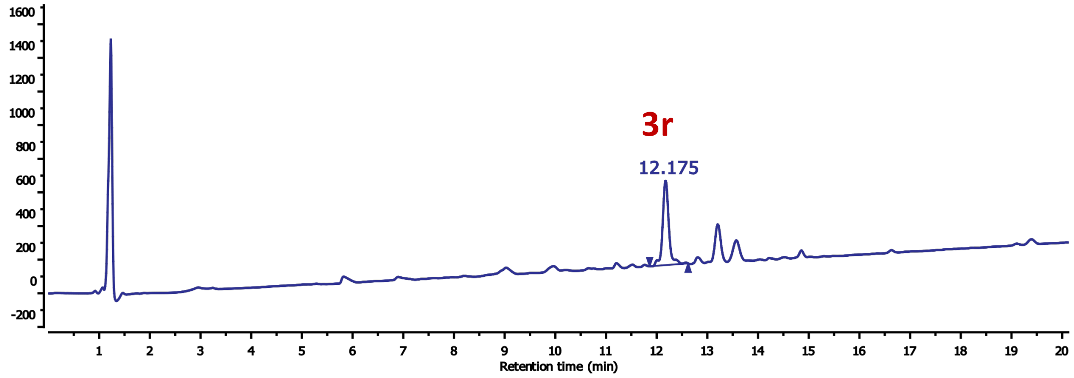

Mass spectra of peak 12.175 (3r)

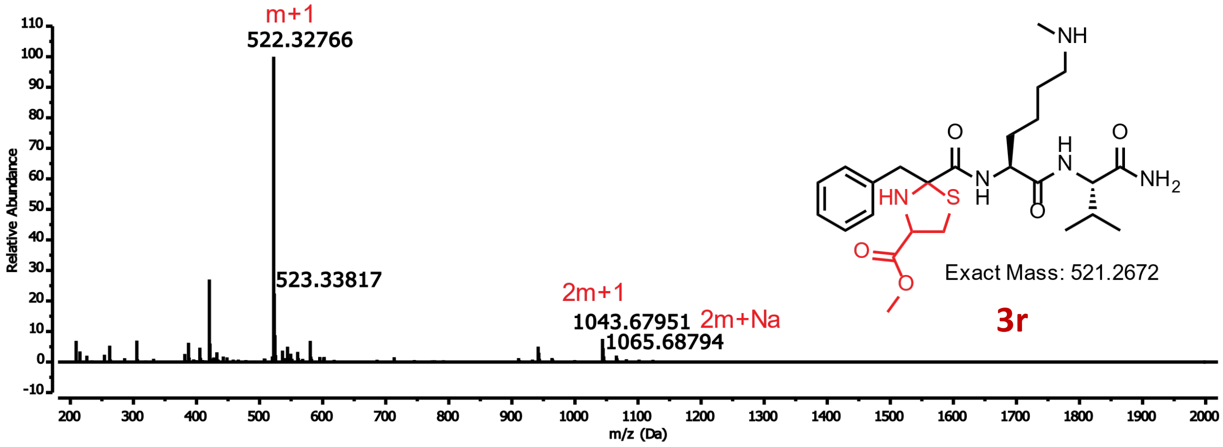

XXIV. Supplementary Figure 17. Synthesis of N,N-dimethyl amino acids.

General procedure 1 for the synthesis of N,N-dimethyl amino acid: To 1 g of amino acid was added 5 equiv. of formaldehyde and 5 equiv. of formic acid. The reaction mixture was refluxed at 100 ^{0}C for 16 h. After being stirred for 16 h, the reaction mixture was basified using 10% NaOH solution. The organic material was extracted with CH_2Cl_2, and the extracts were dried over $MgSO_4$, filtered, and concentrated in vacuo. The residue was purified by column chromatography to give the title compound.

General procedure 2 for the synthesis of N,N-dimethyl amino acid: To a solution of amino acid (1g) and 3 mL formalin (37% in water) in DMF (20 mL) was added 4 g $NaBH(OAc)_3$. After being stirred for 16 h, the reaction mixture was concentrated in vacuo, and the residue was diluted with 30 mL of $NaHCO_3$. The organic material was extracted with EtOAc, and the extracts were dried over $MgSO_4$, filtered, and concentrated in vacuo. The residue was purified by column chromatography to give the title compound.

General procedure for the synthesis of amino acid methyl ester hydrochloride: To 1 g of amino acid, 2 equiv. of freshly distilled chlorotrimethylsilane was added slowly and stirred at room temperature for 5 minutes. Then methanol (100 mL) was added, and the resulting solution or suspension was stirred at room temperature for 16 h. After completion of the reaction, the reaction mixture was concentrated on a rotary evaporator to give the amino acid methyl ester chloride.

Synthesis of N,N-dimethyl phenylalanine methylester.

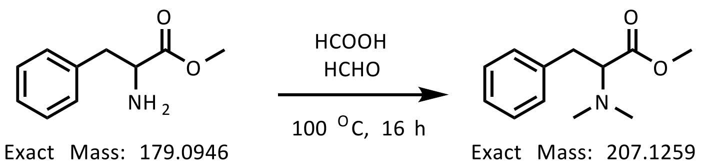

1 g of N,N-dimethyl phenylalanine methylester was synthesized using **general procedure 1 for the synthesis of N,N-dimethyl amino acid.**

N,N-dimethyl phenylalanine methylester: ^{1}**H NMR** (400 MHz, CD_3OD) δ 7.29 – 7.21 (m, 2H), 7.22 – 7.14 (m, 3H), 3.55 (s, 3H), 3.41 (dd, J = 5.1 Hz, 1H), 3.04 – 2.92 (m, 2H), 2.37 (s, 6H). ^{13}**C NMR** (101 MHz, CD_3OD) δ 171.67, 137.61, 128.78, 128.05, 126.22, 69.68, 50.15, 47.40, 47.19, 46.98, 40.85, 35.50.

^{1}H NMR of N'N-dimethyl phenylalanine methylester

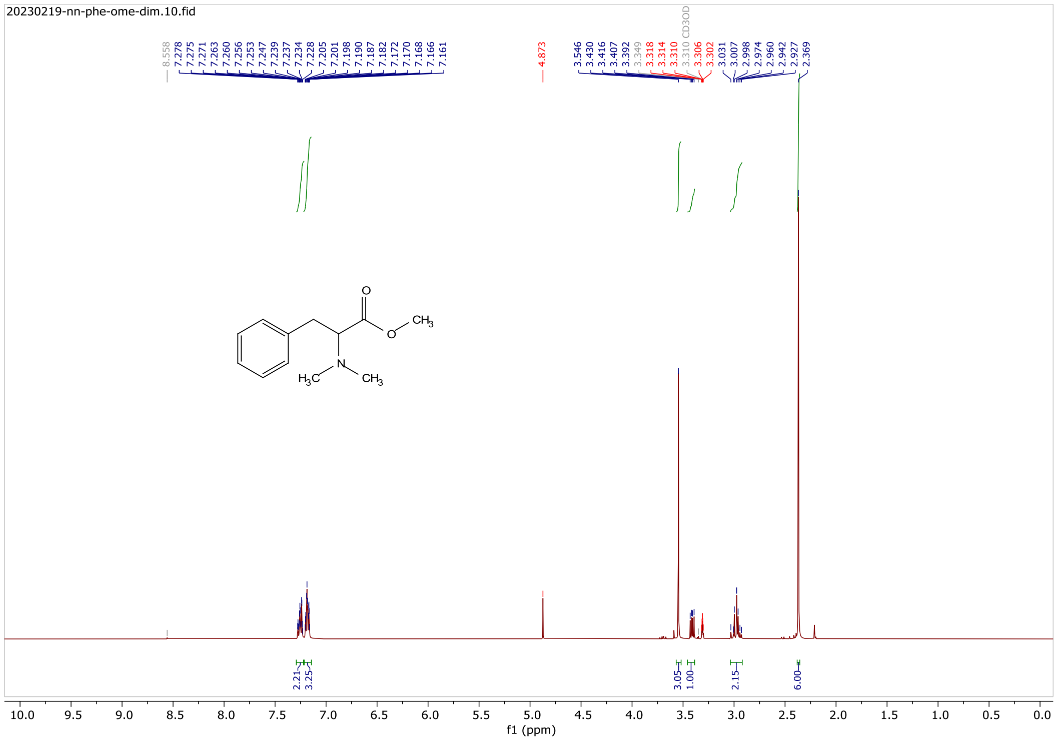

^{13}C NMR of N'N-dimethyl phenylalanine methyl ester

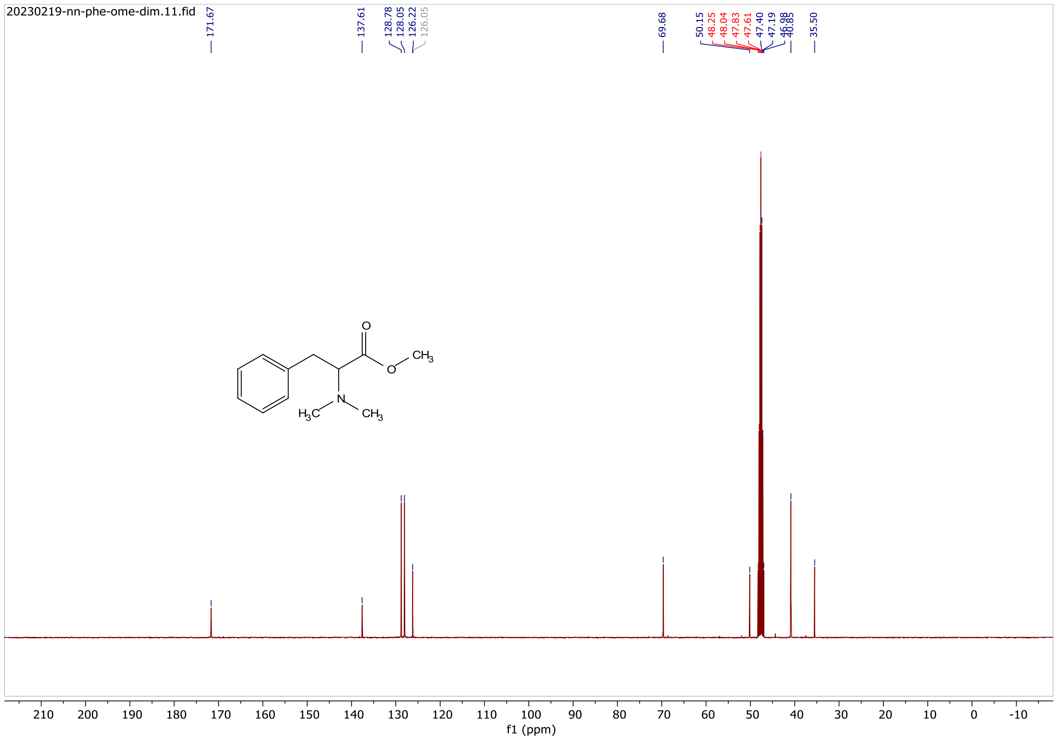

Synthesis of N,N-dimethyl serine methyl ester.

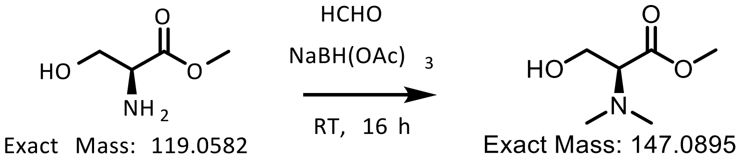

1 g of N,N-dimethyl serine methylester was synthesized from serine methyl ester using **general procedure 1 for the synthesis of N,N-dimethyl amino acid.**

N,N-dimethyl serine methylester: ^{1}**H NMR** (400 MHz, CD_3OD) δ 3.96 – 3.79 (m, 2H), 3.76 (s, 3H), 3.50 (t, *J* = 6.0 Hz, 1H), 2.49 (s, 6H). ^{13}**C NMR** (101 MHz, CD_3OD) δ 172.49, 35.58, 30.28, 26.07, 19.84.

^{1}H NMR of N,N-dimethyl serine methyl ester

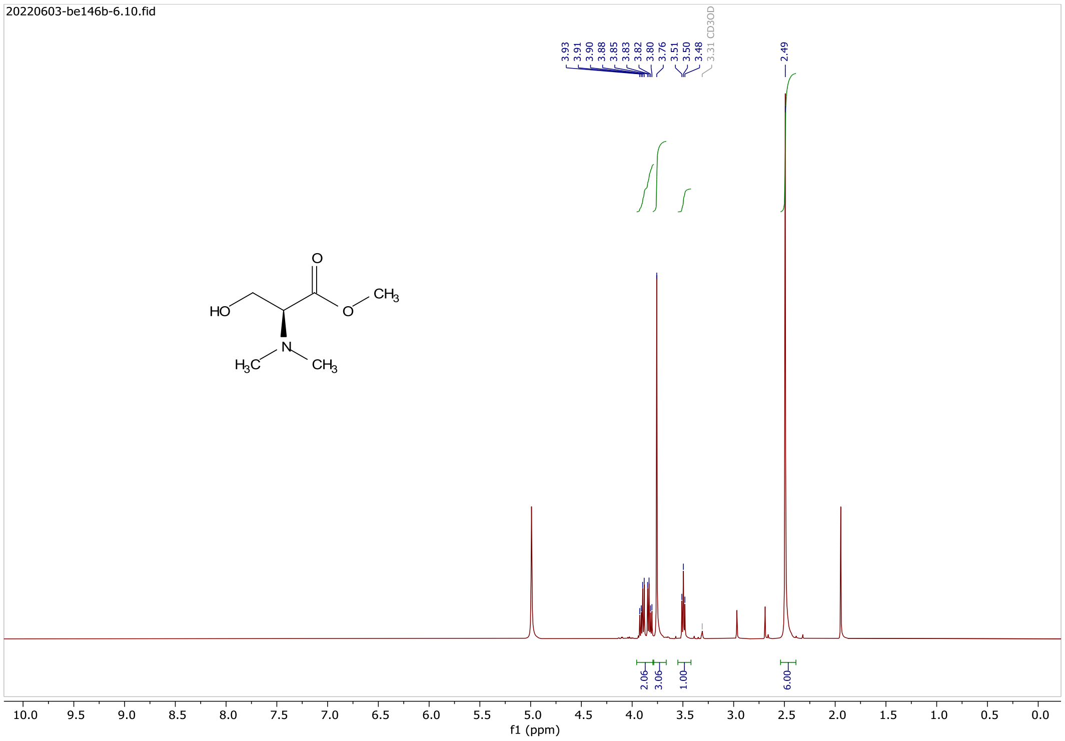

^{13}C NMR of N,N-dimethyl serine methyl ester

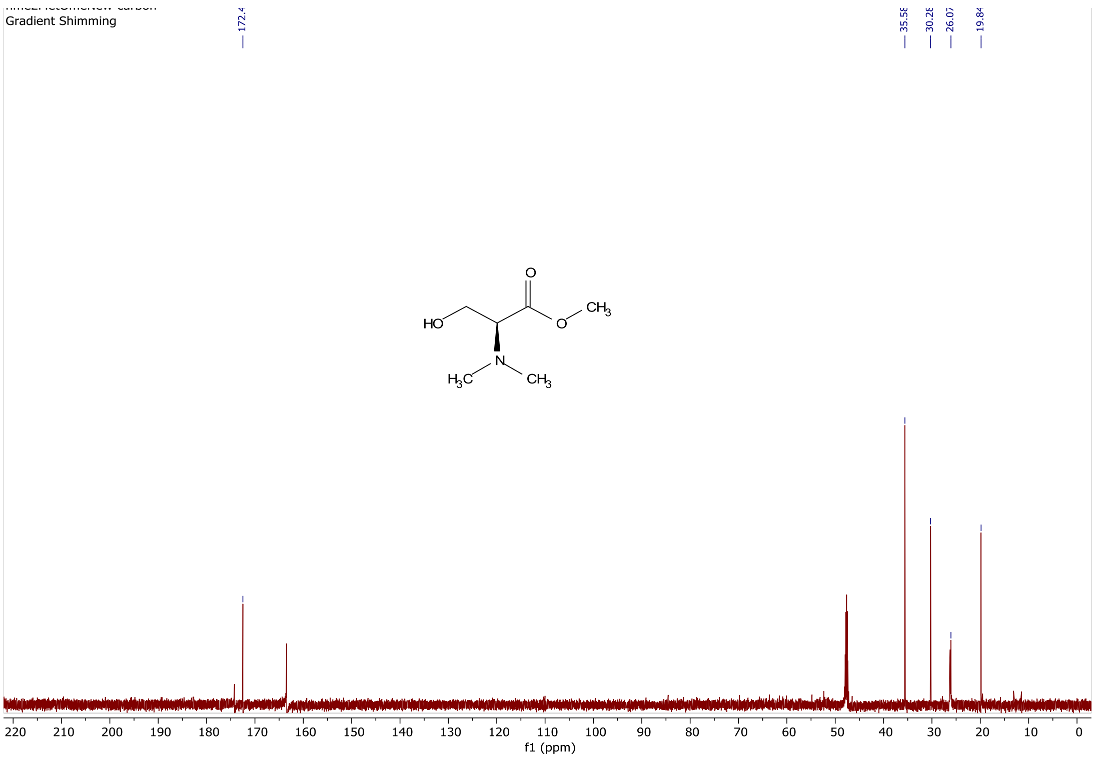

Synthesis of N,N-dimethyl lysine methyl ester.

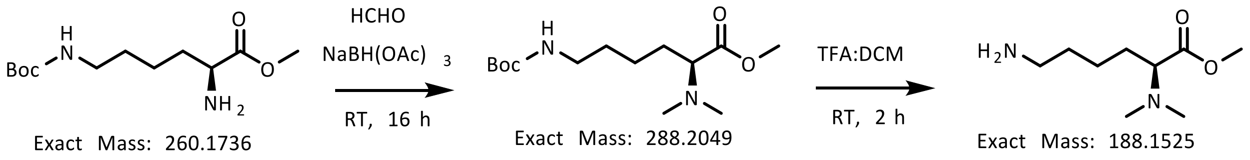

1 g of methyl N_6-(tert-butoxycarbonyl)-N_2,N_2-dimethyl-L-lysinate was synthesized from methyl N_6-(tert-butoxycarbonyl)-L-lysinate using **general procedure 1 for the synthesis of N,N-dimethyl amino acid.**

methyl N_6-(tert-butoxycarbonyl)-N_2,N_2-dimethyl-L-lysinate: ^{1}H NMR (400 MHz, CD_3OD) δ 3.75 (s, 3H), 3.30 (t, *J* = 6.0 Hz, 1H), 3.05 (q, *J* = 6.9 Hz, 2H), 2.42 (s, 6H), 1.77 (qdd, *J* = 13.2, 9.1, 6.7 Hz, 2H), 1.55 – 1.49 (m, 2H), 1.45 (s, 9H), 1.38 – 1.27 (m, 2H). **^{13}C NMR** (101 MHz, CD_3OD) δ 172.49, 163.39, 60.07, 47.59, 47.30, 35.52, 30.22, 27.38, 19.46, 13.07.

^{1}H NMR of methyl N_6-(tert-butoxycarbonyl)-N_2,N_2-dimethyl-L-lysinate

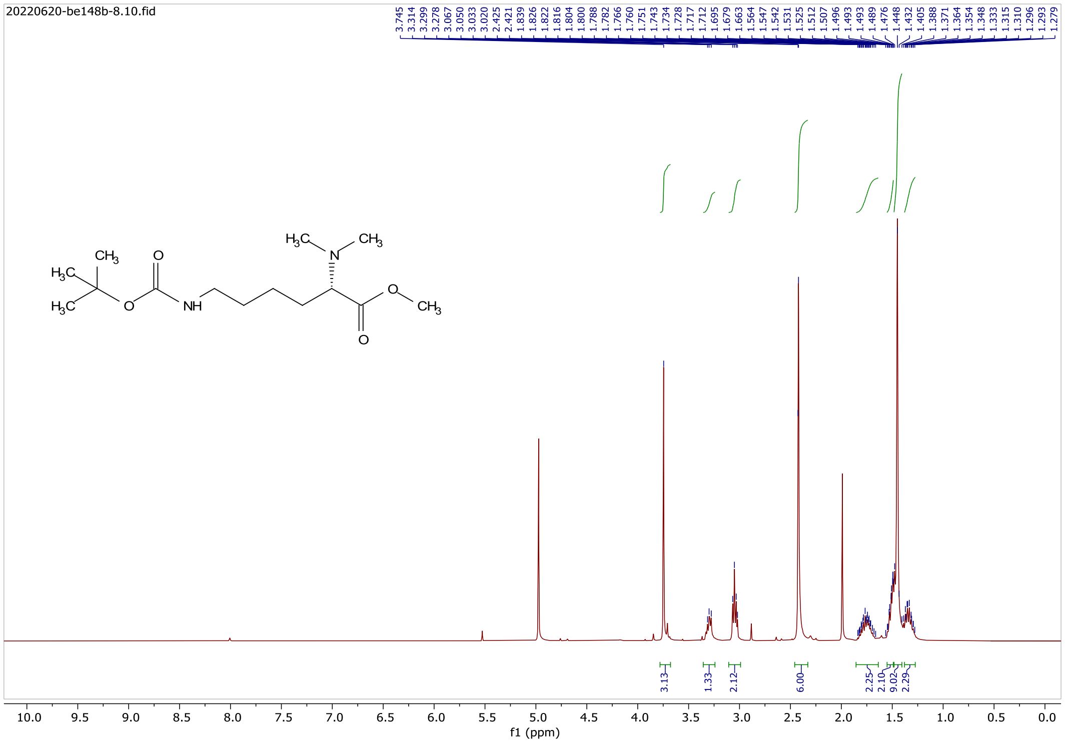

^{13}C NMR of methyl N_6-(tert-butoxycarbonyl)-N_2,N_2-dimethyl-L-lysinate

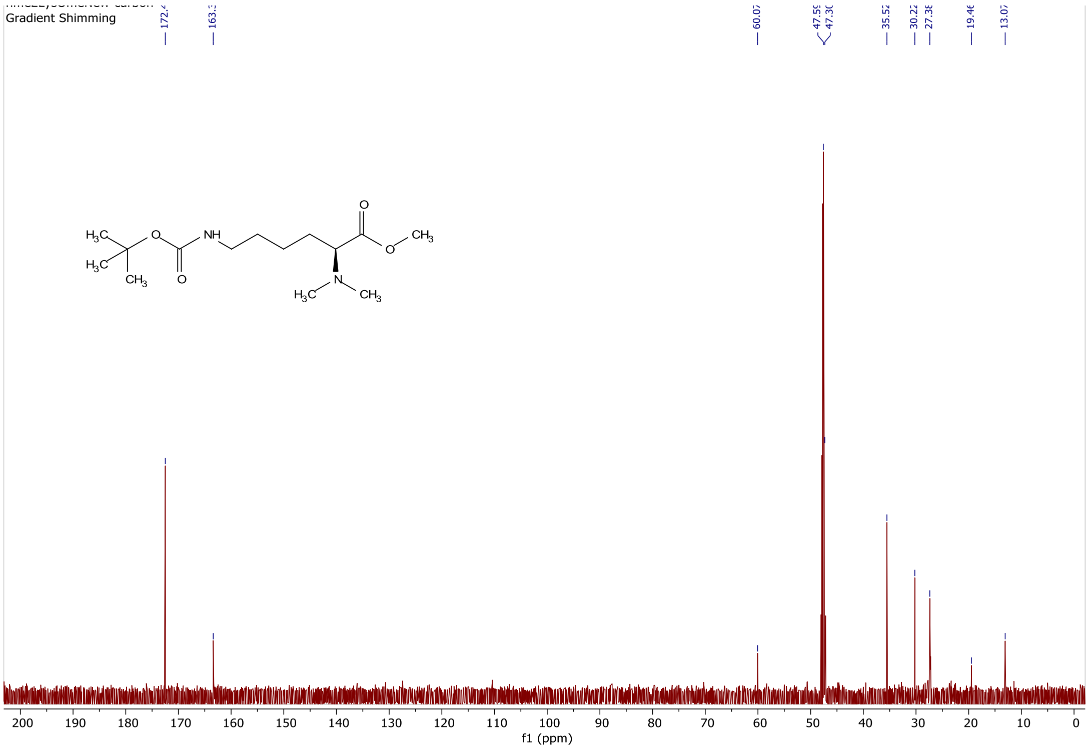

Boc-deprotection of methyl N_6-(tert-butoxycarbonyl)-N_2,N_2-dimethyl-L-lysinate: To 1 g of methyl N_6-(tert-butoxycarbonyl)-N_2,N_2-dimethyl-L-lysinate was added 10 mL of CH_2Cl_2 and 10 mL of trifluoroacetic acid. Reaction was stirred at room temperature for 2 h. After completion of the reaction, the reaction mixture was concentrated on a rotary evaporator to give **N,N-dimethyl lysine methylester.**

N,N-dimethyl lysine methylester: ^{1}**H NMR** (400 MHz, CD_3OD) δ 3.99 (dd, *J* = 27.1, 7.3 Hz, 1H), 3.78 (s, 3H), 3.01 – 2.87 (m, 2H), 2.83 (s, 6H), 2.62 (t, *J* = 6.5 Hz, 2H), 2.04 – 1.75 (m, 2H), 1.73 – 1.51 (m, 2H), 1.50 – 1.29 (m, 2H). ^{13}**C NMR** (101 MHz, CD_3OD) δ 172.49, 60.15, 53.47, 52.31, 25.87, 21.65, 15.53, 13.11.

^{1}H NMR of N,N-dimethyl lysine methyl ester

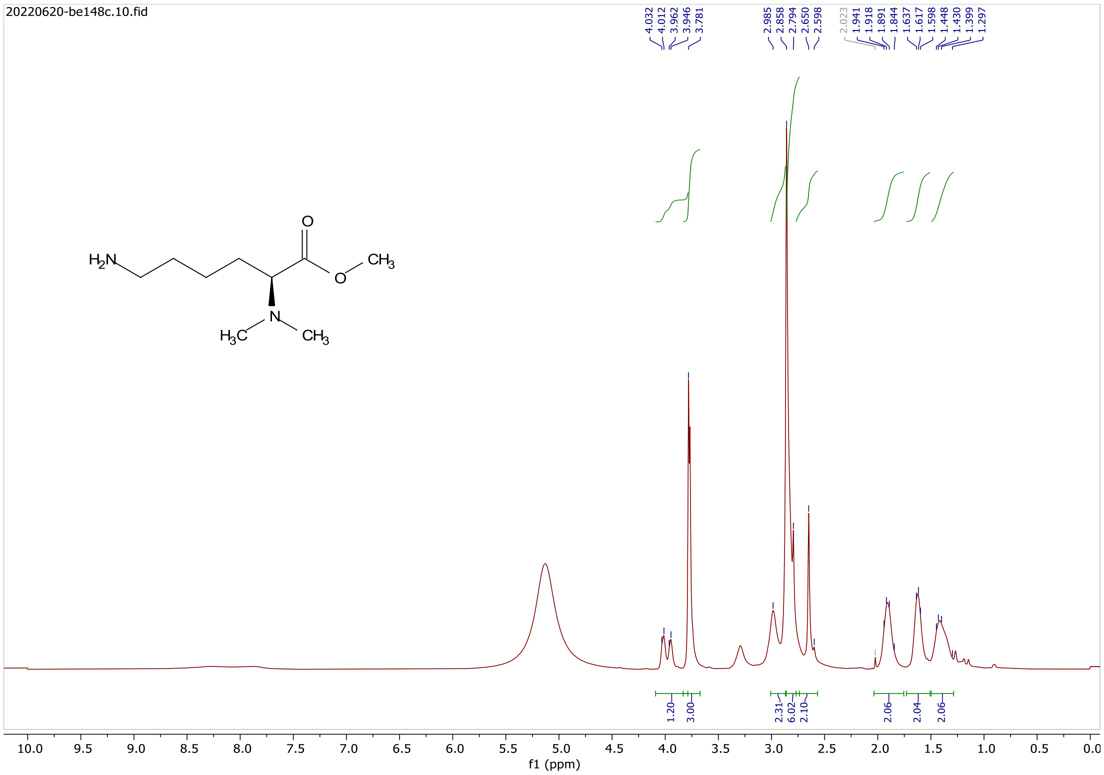

^{13}C NMR of N,N-dimethyl lysine methyl ester

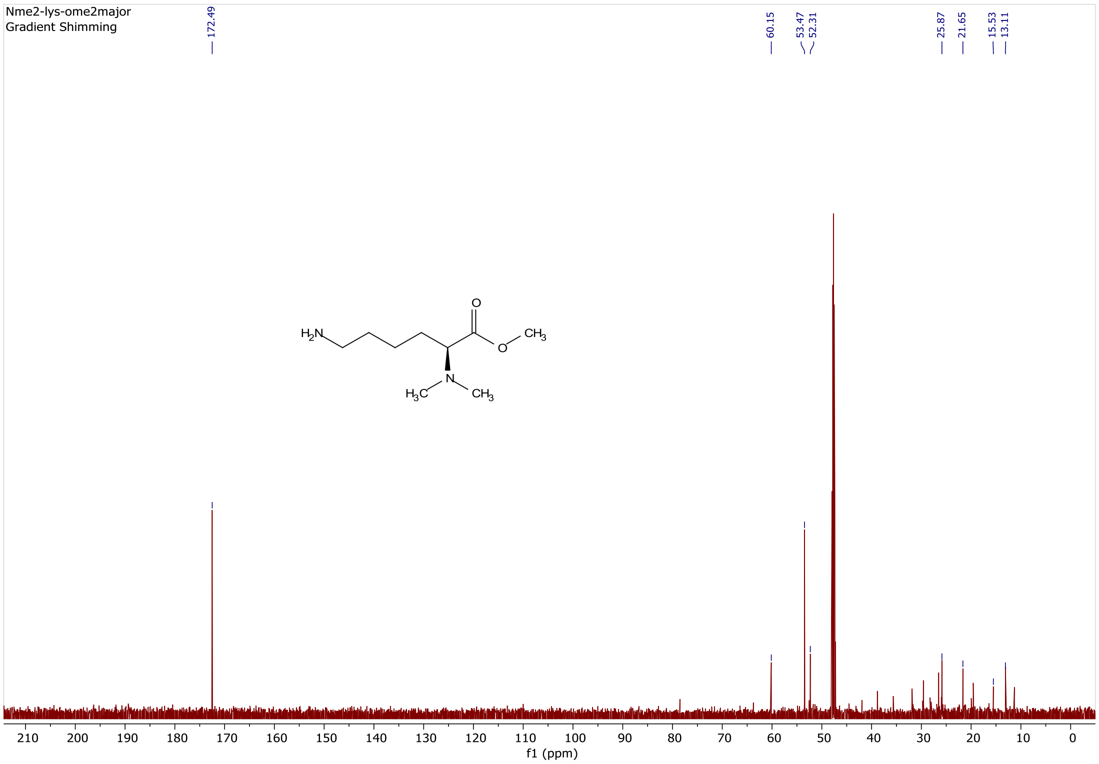

Synthesis of N,N-dimethyl aspartic acid methyl ester.

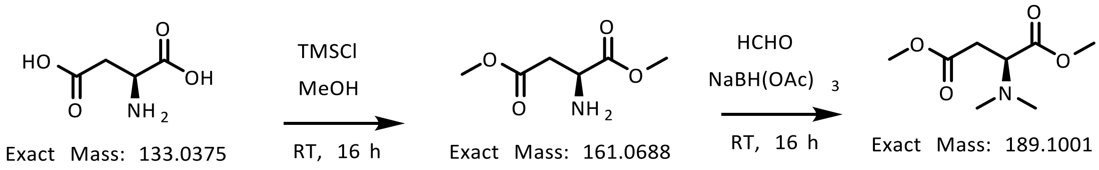

1 g of **dimethyl L-aspartate** was synthesized from aspartic acid using **General procedure for the synthesis of amino acid methyl ester hydrochloride.**

Dimethyl L-aspartate: ^{1}**H NMR** (400 MHz, CD_3OD) δ 4.31 (d, *J* = 31.1 Hz, 1H), 3.80 (s, 3H), 3.71 (s, 3H), 3.03 (s, 2H). ^{13}**C NMR** (101 MHz, CD_3OD) δ 170.18, 168.35, 53.44, 52.38, 49.36, 33.79.

^{1}H NMR of aspartic acid methyl ester

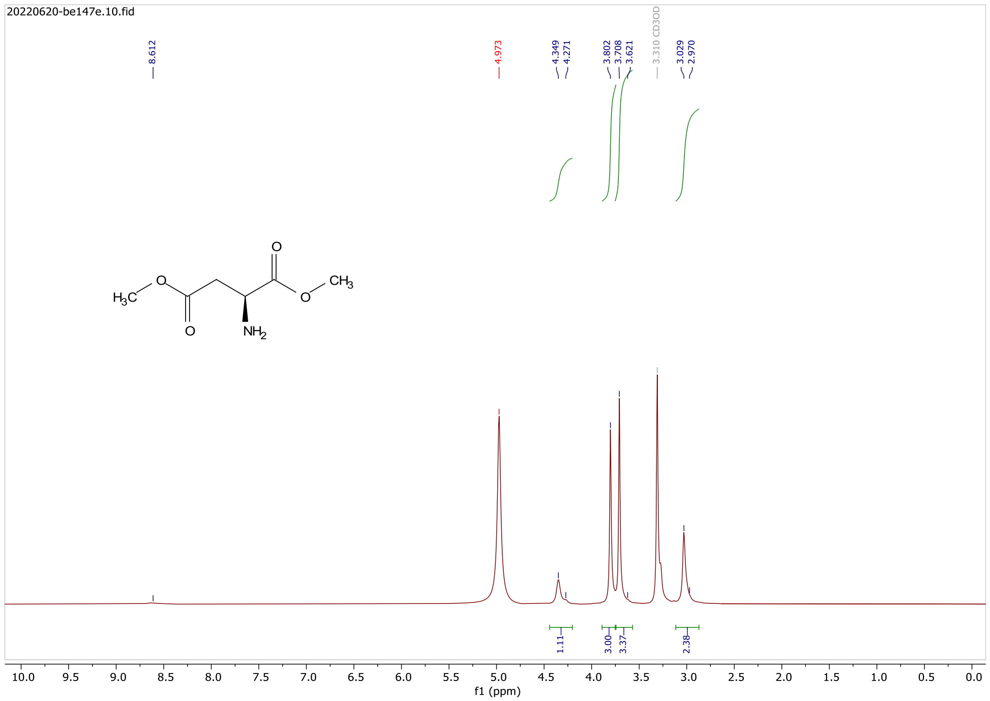

^{13}C NMR of aspartic acid methyl ester

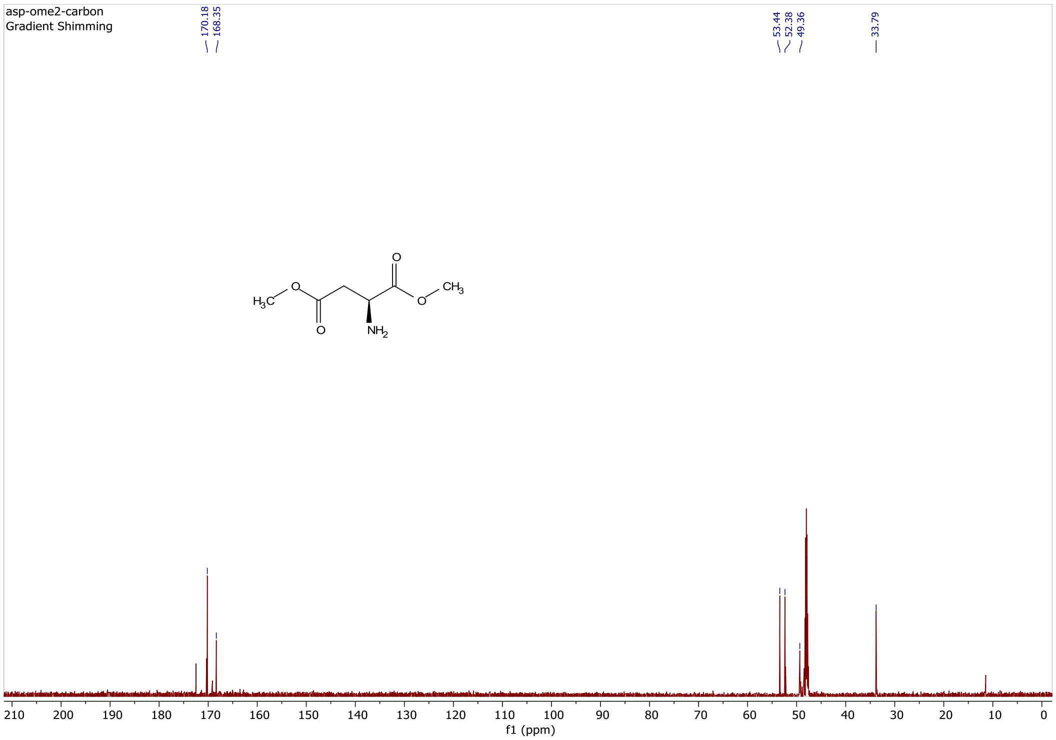

1 g of **dimethyl N,N-dimethylaspartate** was synthesized from **dimethyl L-aspartate** using **general procedure 1 for the synthesis of N,N-dimethyl amino acid.**

Dimethyl N,N-dimethylaspartate: ^{1}**H NMR** (400 MHz, CD_3OD) δ 3.76 (t, *J* = 6.8 Hz, 1H), 3.74 (s, 3H), 3.69 (s, 3H), 2.85 (dd, *J* = 7.5 Hz, 1H), 2.65 (dd, *J* = 7.5 Hz, 1H), 2.34 (s, 6H). ^{13}**C NMR** (101 MHz, CD_3OD) δ 171.95, 171.53, 72.81, 60.15, 53.44, 19.50, 13.07.

^{1}H NMR of dimethyl N,N-dimethylaspartate

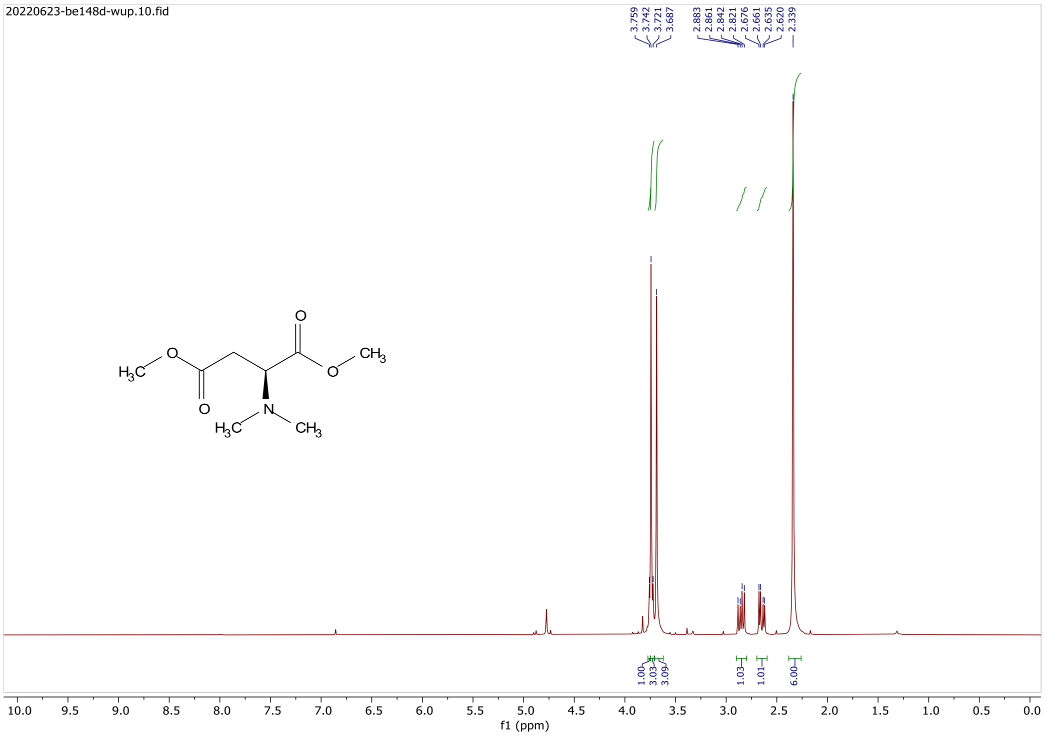

^{13}C NMR of dimethyl N,N-dimethylaspartate

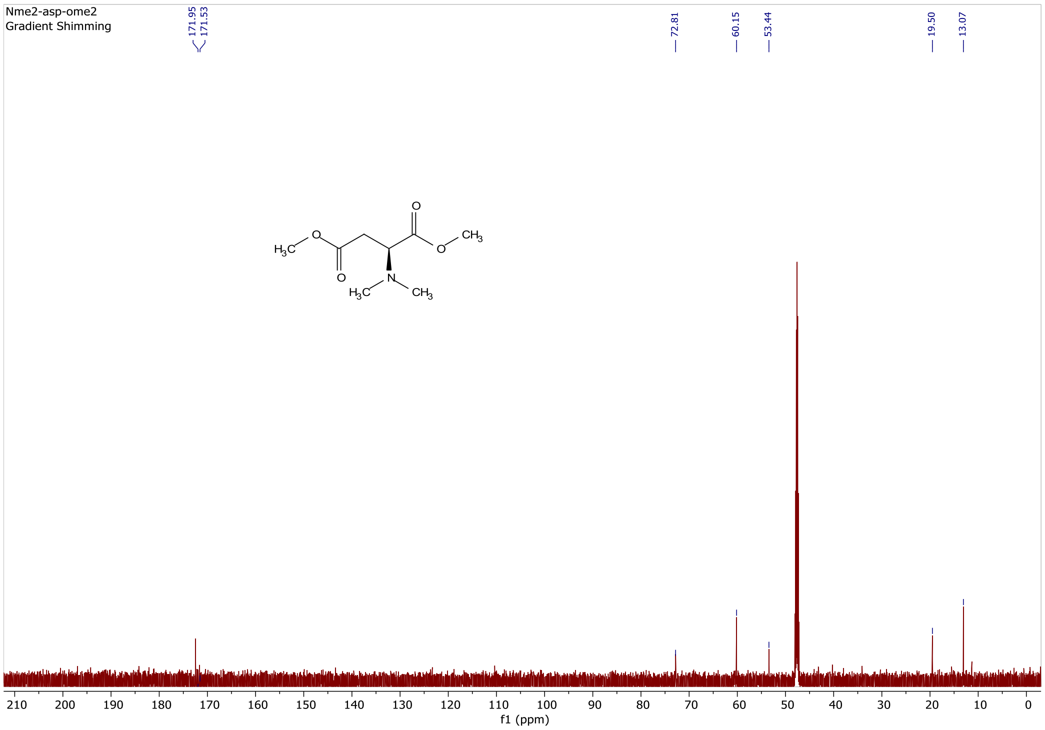

Synthesis of N,N-dimethyl methionine methylester.

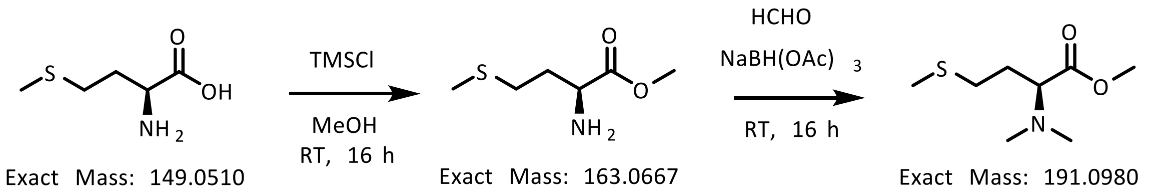

1 g of **methionine methyl ester** was synthesized from **methionine hydrochloride** using **General procedure for the synthesis of amino acid methyl ester hydrochloride.**

Methionine methyl ester: ^{1}H NMR (400 MHz, CD_3OD) δ 4.21 (t, *J* = 6.3 Hz, 1H), 3.85 (s, 3H), 2.65 (t, *J* = 7.2 Hz, 2H), 2.22 (ddt, *J* = 27.0, 13.7, 6.7 Hz, 2H), 2.11 (s, 3H). **^{13}C NMR** (101 MHz, CD_3OD) δ 169.37, 52.38, 51.33, 29.24, 28.63, 13.52.

^{1}H NMR of methionine methylester

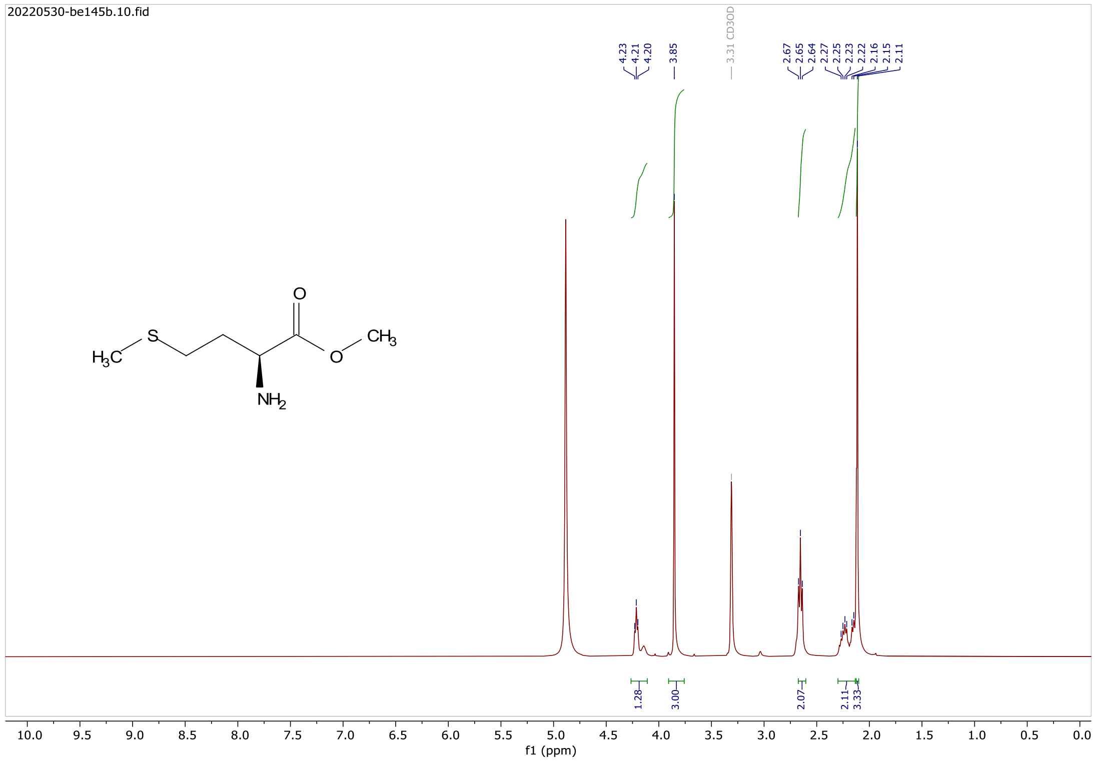

^{13}C NMR of methionine methylester

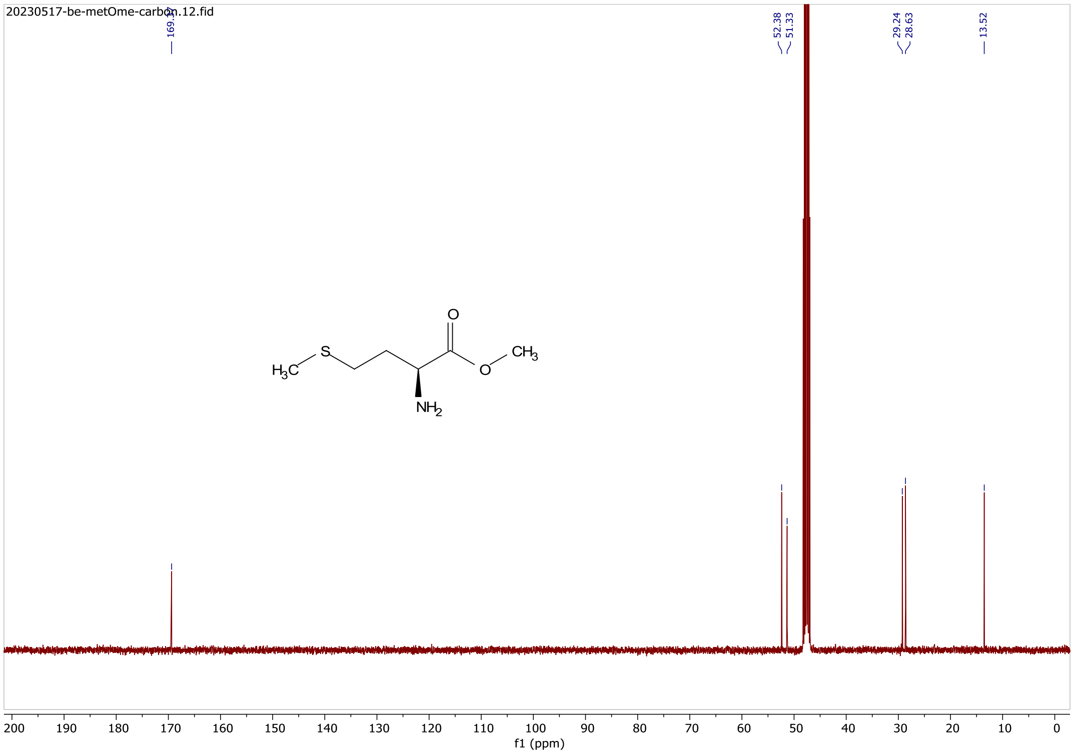

1 g of **N,N-dimethyl methionine methyl ester** was synthesized from **methionine methyl ester** using **general procedure 1 for the synthesis of N,N-dimethyl amino acid.**

N,N-dimethyl methionine methyl ester: ^{1}H NMR (400 MHz, CD_3OD) δ 3.72 (s, 3H), 3.37 (t, *J* = 7.2 Hz, 1H), 2.52 (t, *J* = 7.3 Hz, 2H), 2.33 (s, 6H), 2.08 (s, 3H), 2.00 (dt, *J* = 14.8, 7.2 Hz, 1H), 1.90 (dq, *J* = 14.1, 7.0 Hz, 1H). **^{13}C NMR** (101 MHz, CD_3OD) δ 163.37, 48.51, 47.66, 35.57, 30.28, 19.47, 11.41.

^{1}H NMR of N,N-dimethyl methionine methyl ester

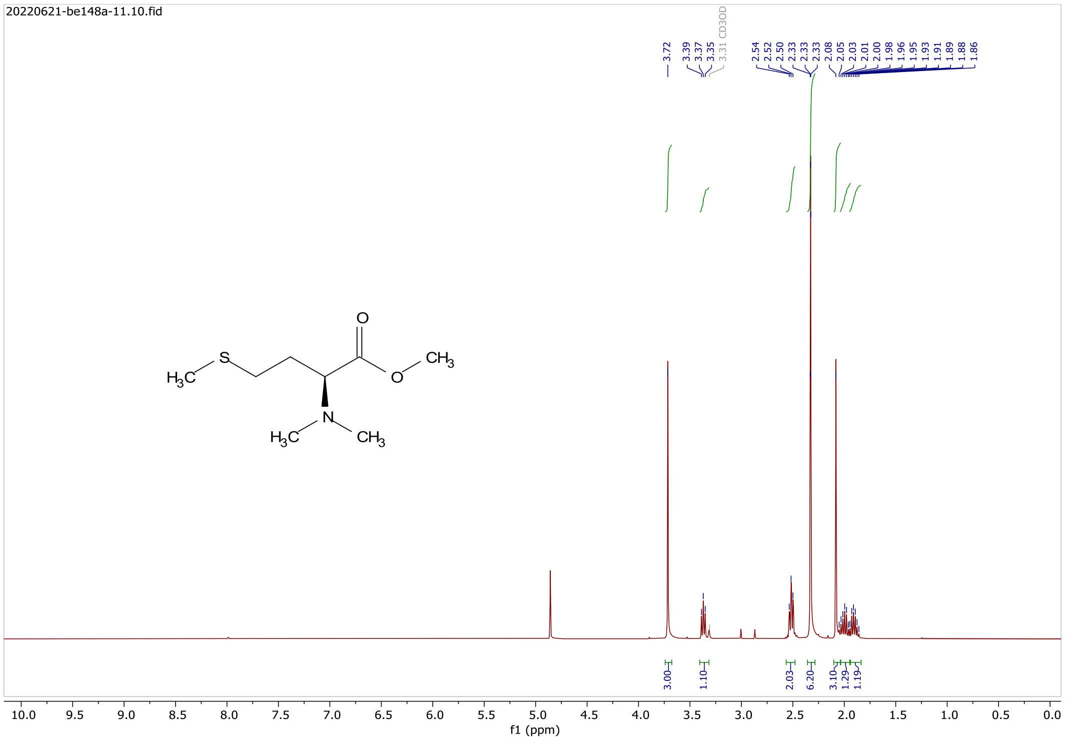

^{13}C NMR of N,N-dimethyl methionine methyl ester

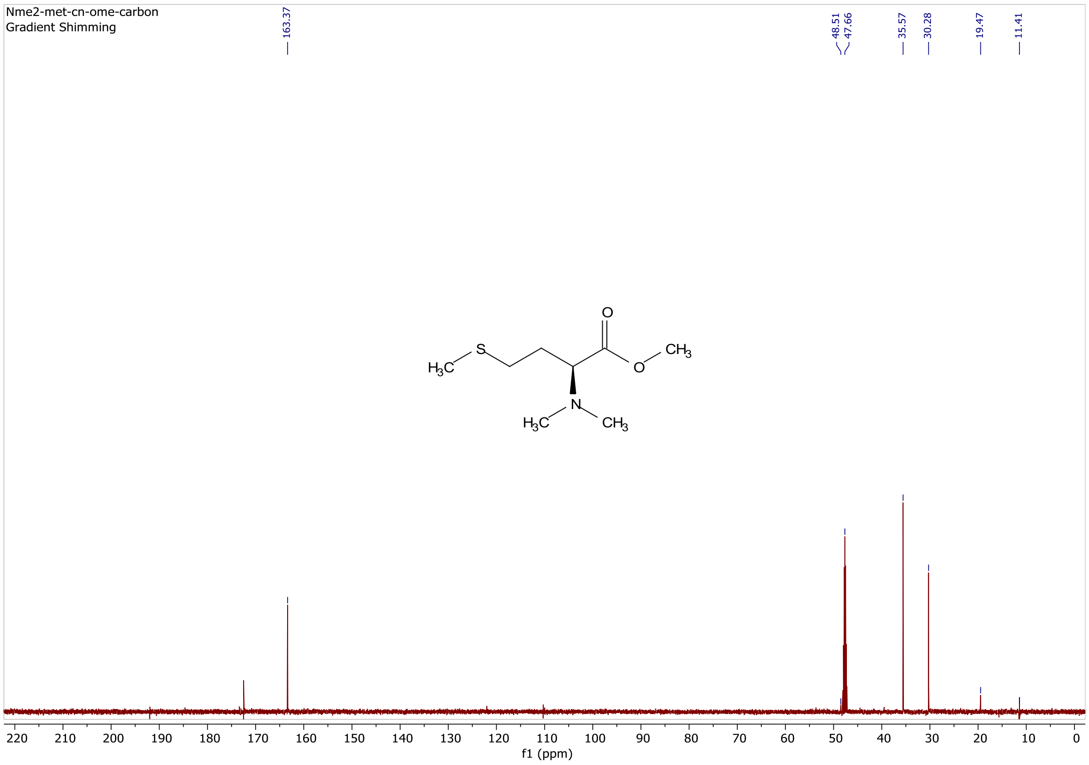

Solid-support mediated synthesis of N,N-dimethyl cysteine phenylalanine amide.

Fmoc Solid-Phase Peptide Synthesis (Fmoc-SPPS).[1] Peptides were synthesized manually on a 0.25 mm scale using Rink amide resin. Resin was swollen with CH_2Cl_2 for 1 h at room temperature. Fmoc was deprotected using 20 % piperidine in DMF for 5 min to obtain a deprotected peptide-resin. First, Fmoc protected phenylalanine (1.25 mm/5 equiv.) was coupled using HOAt (1.25 mm/5 equiv.) and DIC (1.25 mm/5 equiv.) in DMF for 15 min at room temperature. Fmoc-protected cysteine-Trt (0.75 mm/3 equiv.) was subsequently coupled on the resin using HBTU (0.75 mm/3 equiv.) and DIEA (1.5 mm/6 equiv.) in DMF for 5 min at room temperature.

Dimethylation : For N-terminal dimethylation, the last amino acid coupled to the resin (cysteine) was Fmoc-deprotected using 20 % piperidine in DMF. To the deprotected peptide on resin, 2 mL of 37 % formaldehyde in water solution was added to resin-bound peptide in DMF. 1 g of sodium triacetoxyborohydride was added to the mixture and stirred 10 h. Resin-bound peptide was cleaved from the resin using a cocktail of 95:2.5:2.5, trifluoroacetic acid: water: TES for 2 h. The resin was removed by filtration and the resulting solution was concentrated. The residue was diluted with ACN/water mixture. The resulting solution was purified by HPLC to afford ***N,N-dimethyl cysteine phenylalanine amide.***

N,N-dimethyl cysteine phenylalanine amide: **^{1}H NMR** (400, CD_3OD) δ 7.35 – 7.25 (m, 3H), 7.28 – 7.17 (m, 2H), 4.85 – 4.81 (m, 1H), 3.73 (dd, J = 8.1, 4.0 Hz, 1H), 3.30 – 3.24 (m, 1H), 3.06 (dd, J = 14.2, 4.0 Hz, 1H), 2.98 – 2.82 (m, 2H), 2.47 (s, 6H), 2.03 (s, 1H). **^{13}C NMR** (101 MHz, CD_3OD) δ 163.84, 159.88, 129.18, 128.89, 128.15, 126.55, 67.90, 51.20, 47.69, 44.45, 37.56.

^{1}H NMR of N,N-dimethyl cysteine phenylalanine amide

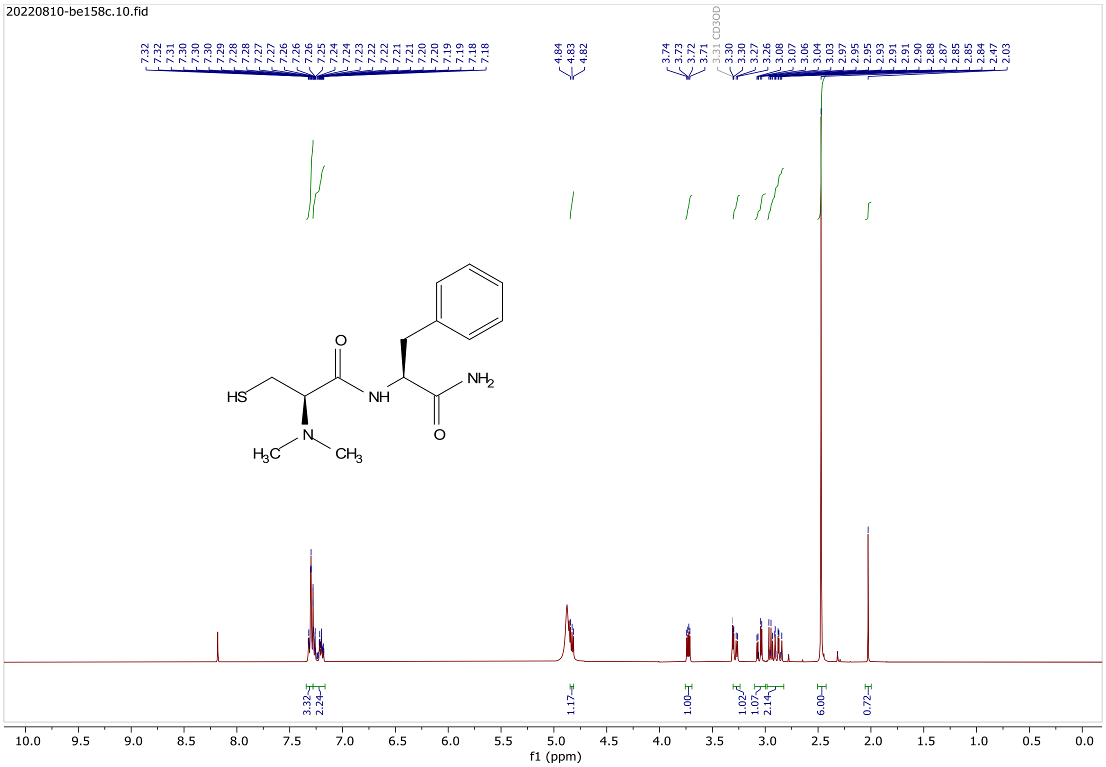

^{13}C NMR of *N,N-dimethyl cysteine phenylalanine amide*

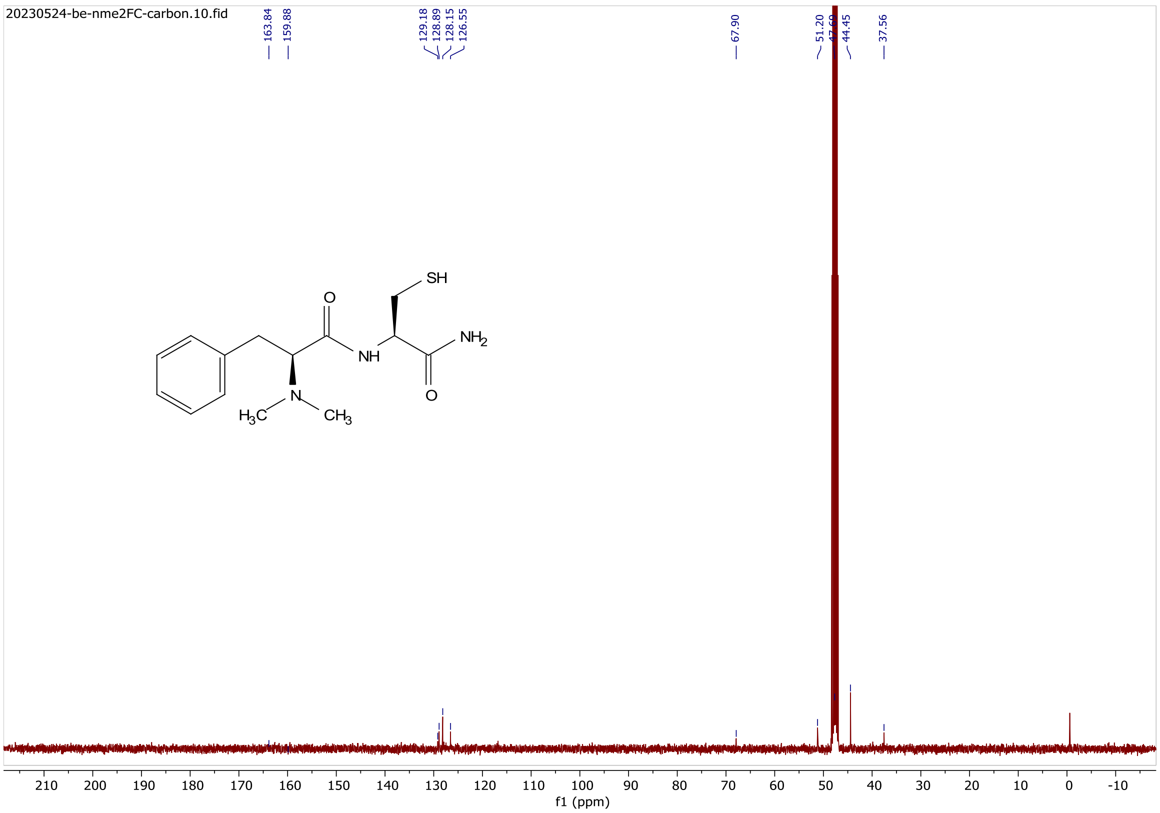

Solid-support mediated synthesis of N,N-dimethyl asparagine phenylalanine amide.

Fmoc Solid-Phase Peptide Synthesis (Fmoc-SPPS).[1] Peptides were synthesized manually on a 0.25 mm scale using Rink amide resin. Resin was swollen with CH_2Cl_2 for 1 h at room temperature. Fmoc was deprotected using 20 % piperidine in DMF for 5 min to obtain a deprotected peptide-resin. First, Fmoc protected phenylalanine (1.25 mm/5 equiv.) was coupled using HOAt (1.25 mm/5 equiv.) and DIC (1.25 mm/5 equiv.) in DMF for 15 min at room temperature. Fmoc-protected asparagine-Trt (0.75 mm/3 equiv.) was subsequently coupled on the resin using HBTU (0.75 mm/3 equiv.) and DIEA (1.5 mm/6 equiv.) in DMF for 5 min at room temperature.

Dimethylation: For N-terminal dimethylation, the last amino acid coupled to the resin (asparagine) was Fmoc-deprotected using 20 % piperidine in DMF. To the deprotected peptide on resin, 2 mL of 37 % formaldehyde in water solution was added to resin-bound peptide in DMF. 1 g of sodium triacetoxyborohydride was added to the mixture and stirred 10 h. Resin-bound peptide was cleaved from the resin using a cocktail of 95:2.5:2.5, trifluoroacetic acid: water: TES for 2 h. The resin was removed by filtration and the resulting solution was concentrated. The residue was diluted with ACN/water mixture. The resulting solution was purified by HPLC to afford ***N,N-dimethyl asparagine phenylalanine amide.***

N,N-dimethyl asparagine *phenylalanine* amide: ^{1}**H NMR** (400 MHz, CD_3OD) δ 7.32 – 7.28 (m, 3H), 7.28 – 7.16 (m, 2H), 4.80 (dd, J = 11.4, 4.3 Hz, 1H), 3.87 (dd, J = 7.9, 5.5 Hz, 1H), 3.40 (dd, J = 14.2, 4.4 Hz, 2H), 2.86 – 2.79 (m, 2H), 2.39 (s, 6H). ^{13}**C NMR** (101 MHz, CD_3OD) δ 174.13, 172.49, 171.22, 137.46, 128.94, 128.22, 126.54, 64.39, 54.31, 40.55, 37.31, 32.92.

^{1}H NMR of N,N-dimethyl asparagine-phenylalanine amide

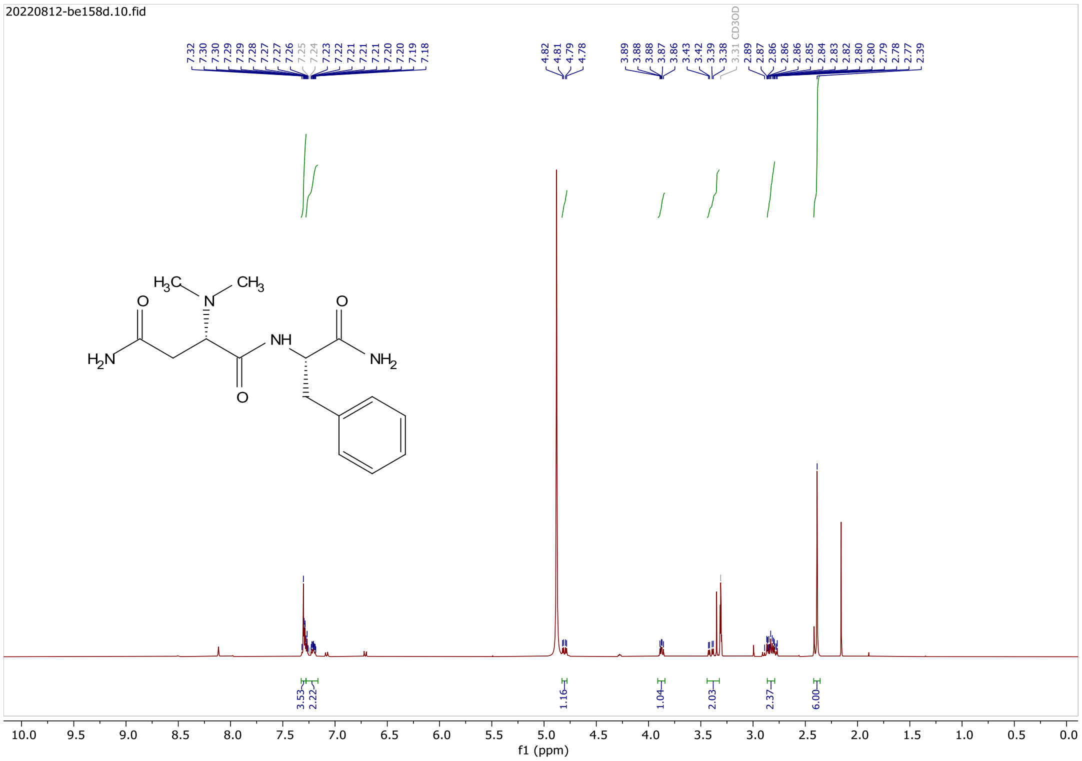

Impurity @2.1 is acetonitrile

^{13}C NMR of *N,N-dimethyl asparagine phenylalanine amide*

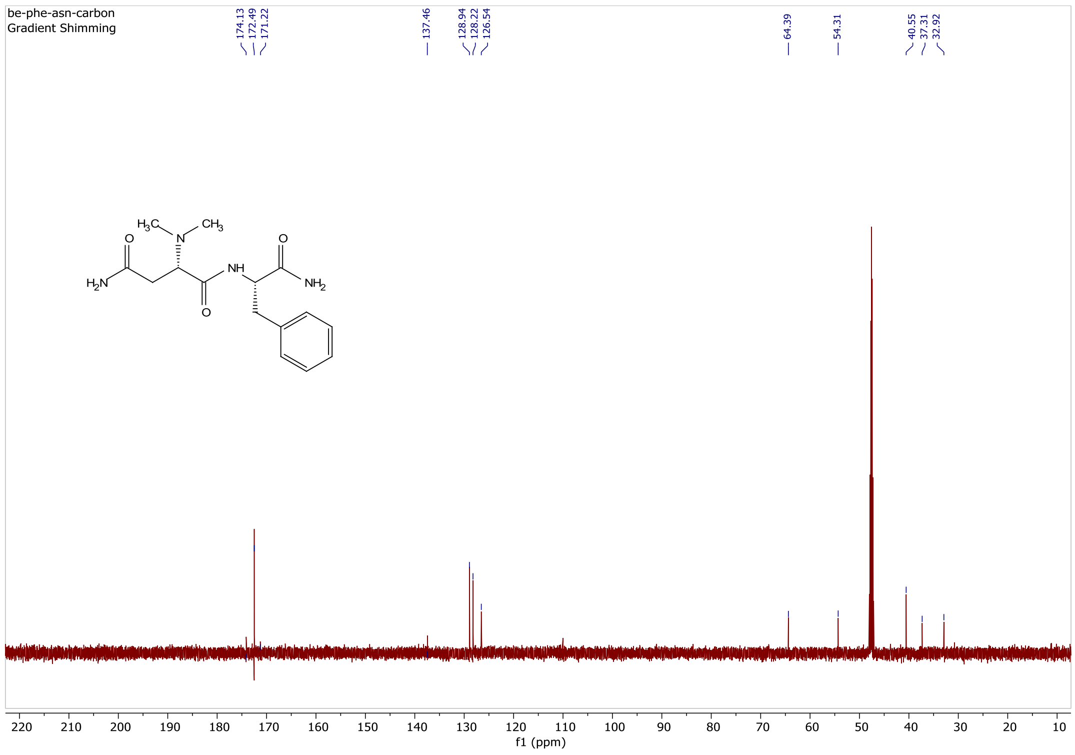

Synthesis of N-methyl proline methyl ester.

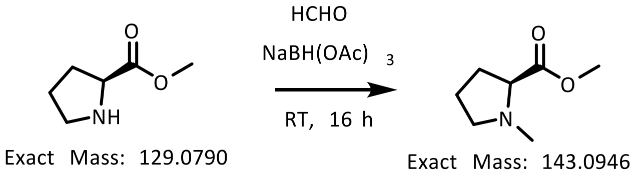

1 g of **N-methyl proline methylester** was synthesized from **proline methyl ester** using **general procedure 1 for the synthesis of N,N-dimethyl amino acid.**

N-methyl proline methylester: ^{1}H NMR (400 MHz, CD_3OD) δ 3.78 (s, 3H), 3.23 (ddd, *J* = 10.3, 6.2, 4.3 Hz, 1H), 2.60 – 2.52 (m, 1H), 2.54 (s, 3H), 2.37 – 2.24 (m, 1H), 2.06 – 1.97 (m, 2H), 1.97 – 1.90 (m, 2H). **^{13}C NMR** (101 MHz, CD_3OD) δ 163.49, 60.14, 53.42, 44.17, 35.57, 19.47, 13.07.

^{1}H NMR of N-methyl proline methylester

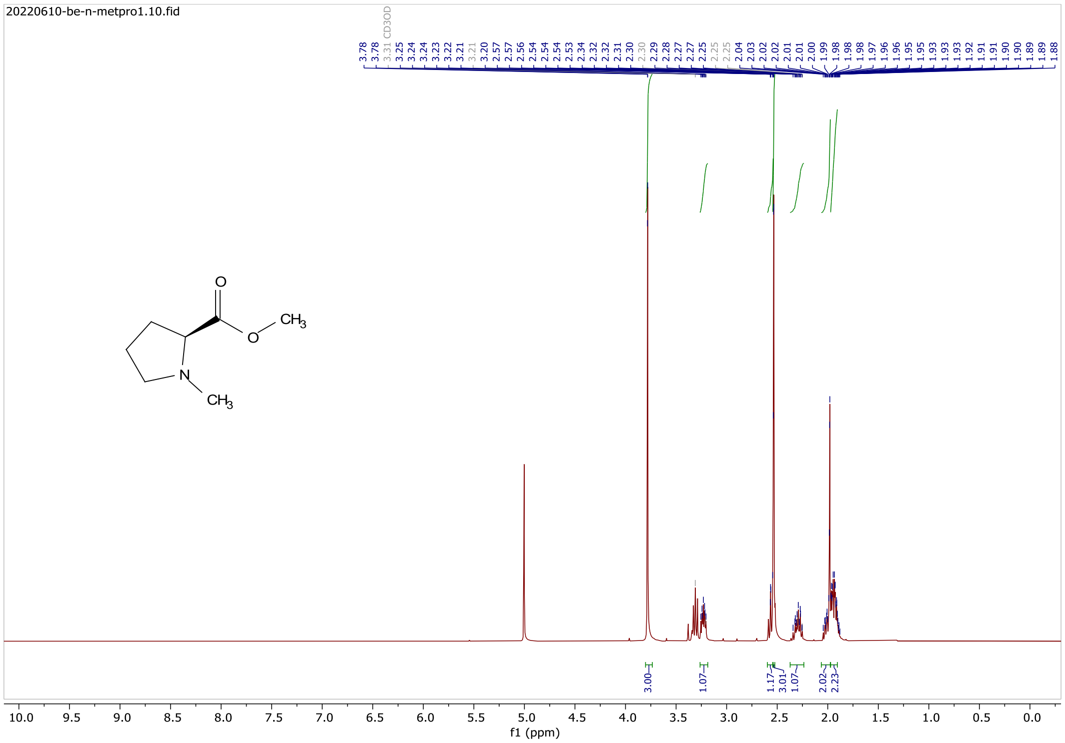

^{13}C NMR of N-methyl proline methylester

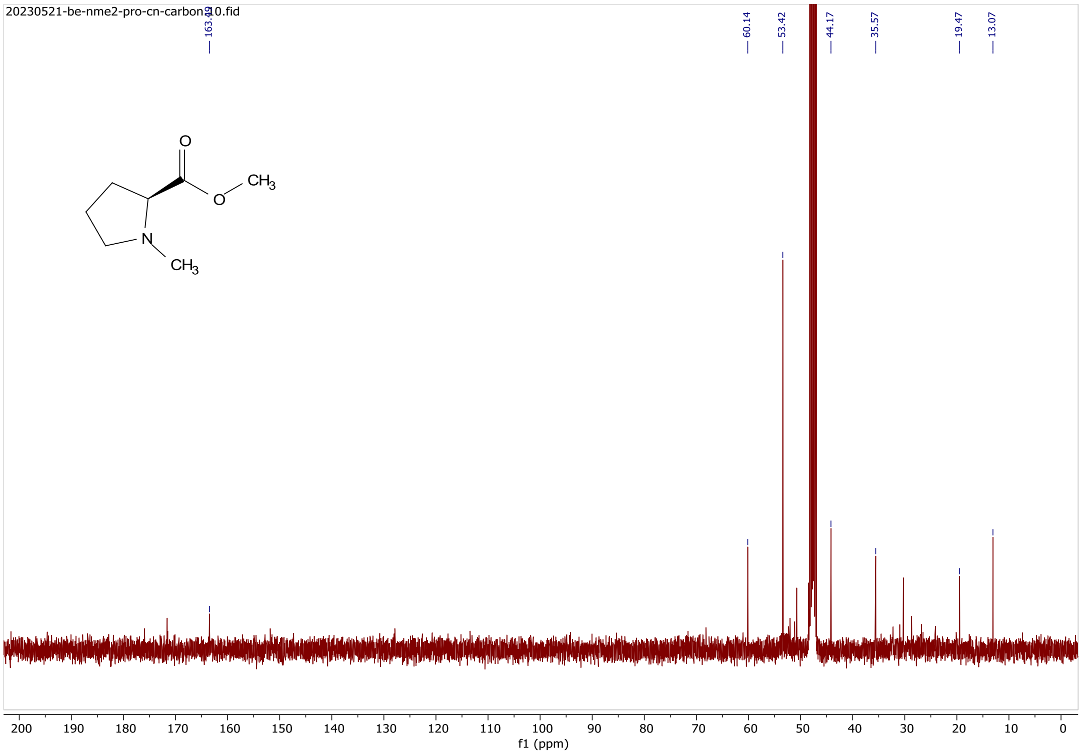

Synthesis of N,N-dimethyl leucine methyl ester.

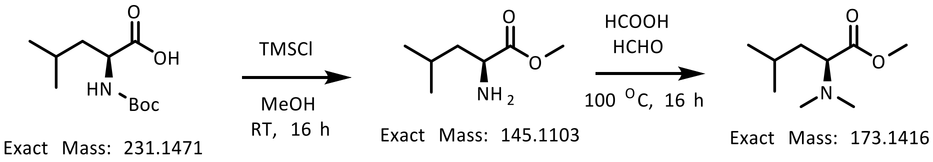

1 g of leucine methyl ester was synthesized from (tert-butoxycarbonyl)-L-leucine using **general procedure for the synthesis of amino acid methyl ester hydrochloride.**

Leucine methyl ester: ^{1}H NMR (600 MHz, DMSO-d_6) δ 3.90 (p, J = 6.5 Hz, 1H), 3.72 (s, 3H), 1.83 – 1.71 (m, 1H), 1.71 – 1.59 (m, 2H), 0.87 (dd, J = 6.5, 1.0 Hz, 6H). **^{13}C NMR** (151 MHz, CD_3OD) δ 170.01, 52.23, 51.08, 39.27, 24.19, 20.92.

^{1}H NMR of leucine methyl ester

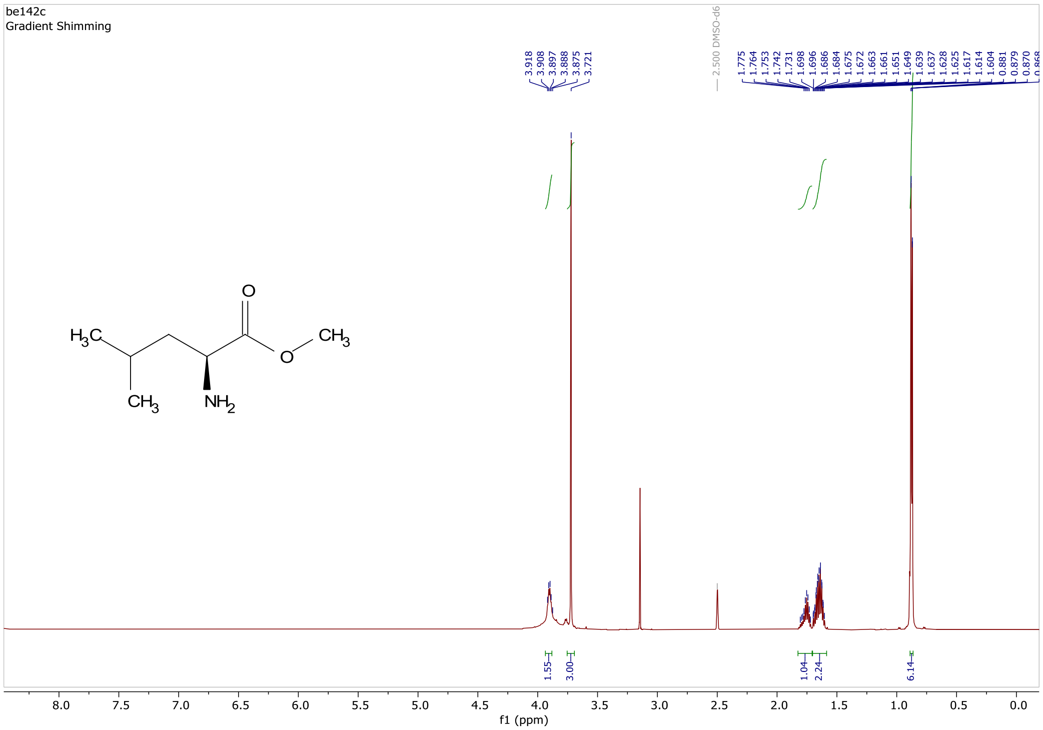

^{13}C NMR of leucine methyl ester

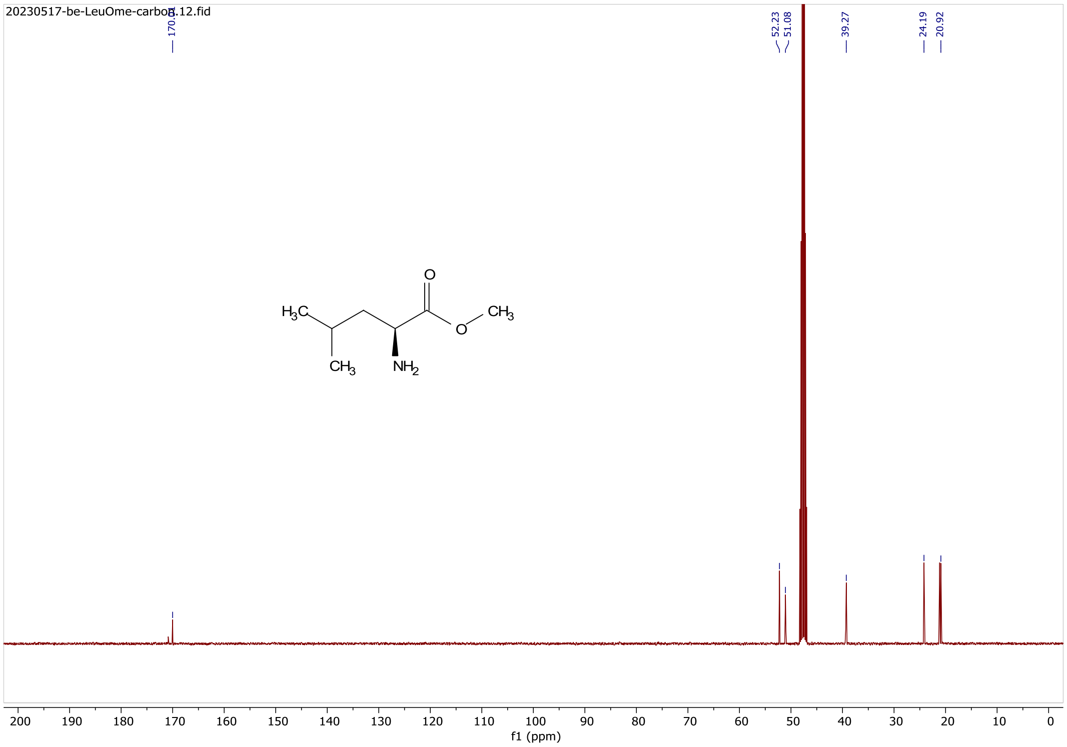

1 g of N,N-dimethyl leucine methyl ester was synthesized from leucine methyl ester using **general procedure 1 for the synthesis of N,N-dimethyl amino acid.**

N,N-dimethyl leucine methylester: ^{1}H NMR (400 MHz, $CDCl_3$) δ 3.67 (s, 3H), 3.19 (dd, J = 8.1, 6.5 Hz, 1H), 2.29 (s, 6H), 1.62 – 1.52 (m, 2H), 1.51 – 1.40 (m, 1H), 0.94 – 0.81 (m, 6H). **^{13}C NMR** (101 MHz, CD_3OD) δ 172.49, 65.42, 50.19, 40.54, 38.41, 24.90, 21.38.

^{1}H NMR of N,N-dimethyl leucine methyl ester

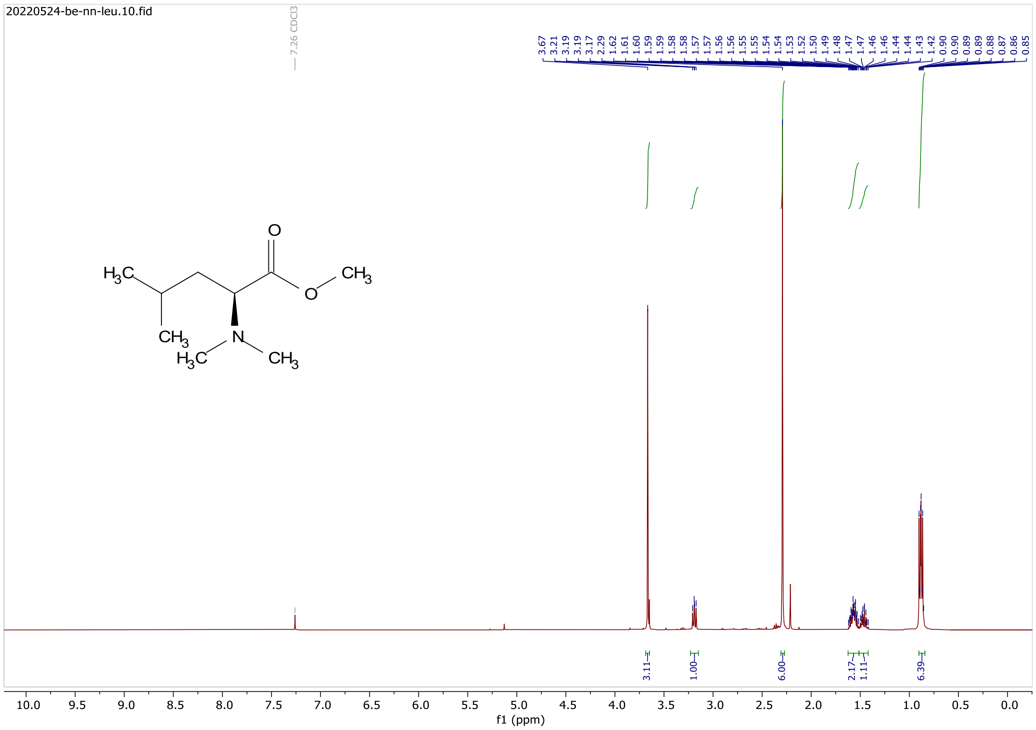

^{13}C NMR of N,N-dimethyl leucine methyl ester

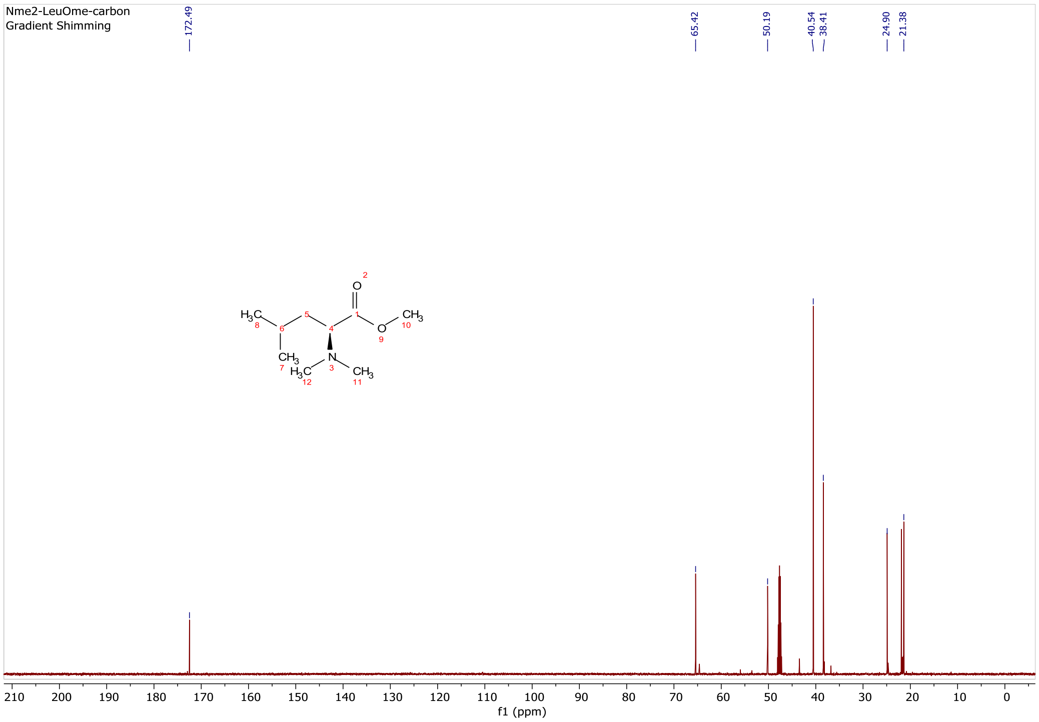

XXV. Supplementary Figure 18. Evaluation of diverse N-terminal amino acids modification.

Nitrilation of N,N-dimethyl phenylalanine methyl ester

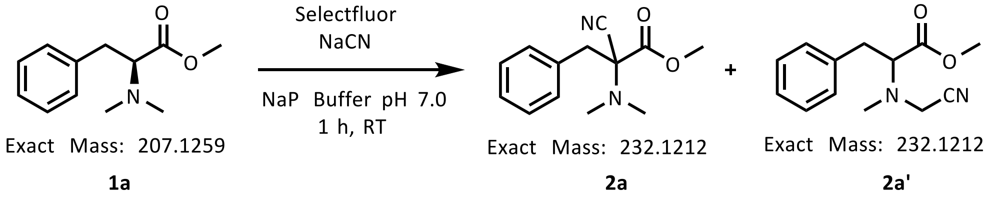

100 mg of N,N-dimethyl phenylalanine methyl ester was modified using **general procedure 1**. Concentration of the mixture gave a residue, which was purified by silica gel column chromatography (eluent: 100 % CH_2Cl_2) to give the nitrile products of N,N-dimethyl phenylalanine methyl ester **2a** and **2a'** which could not be separated by silica gel column chromatography. % Yields were determined by NMR (80 % vs 20 %)

^{1}H NMR of α-nitrile N,N-dimethyl phenylalanine methyl ester 2a (600 MHz, $CDCl_3$) δ 7.35 – 7.30 (m, 3H), 7.30 – 7.27 (m, 2H), 3.56 (s, 3H), 3.37 (d, *J* = 12.8 Hz, 1H), 3.17 (d, *J* = 12.8 Hz, 1H), 2.44 (s, 6H).

^{13}C NMR of α-nitrile N,N-dimethyl phenylalanine methyl ester 2a (151 MHz, $CDCl_3$) δ 167.26, 132.81, 129.96, 128.67, 128.12, 114.51, 74.17, 53.19, 43.01, 40.94.

^{1}H NMR of N-methyl nitrile N,N-dimethyl phenylalanine methyl ester 2a' (600 MHz, $CDCl_3$) δ 7.28 – 7.20 (m, 3H), 7.22 – 7.17 (m, 2H), 3.73 (t, *J* = 4.4 Hz, 1H), 3.68 (d, *J* = 5.9 Hz, 2H), 3.65 (s, 3H), 3.06 (dd, *J* = 13.7, 8.6 Hz, 1H), 2.97 (dd, *J* = 13.6, 6.7 Hz, 1H), 2.53 (s, 3H).

^{13}C NMR of N-methyl nitrile N,N-dimethyl phenylalanine methyl ester 2a' (151 MHz, $CDCl_3$) δ 171.23, 136.95, 129.02, 128.55, 126.83, 115.67, 67.56, 51.55, 42.80, 38.70, 36.02.

^{1}H NMR of α-nitrile and N-methyl nitrile N,N-dimethyl phenylalanine methyl ester products 2a and 2a’

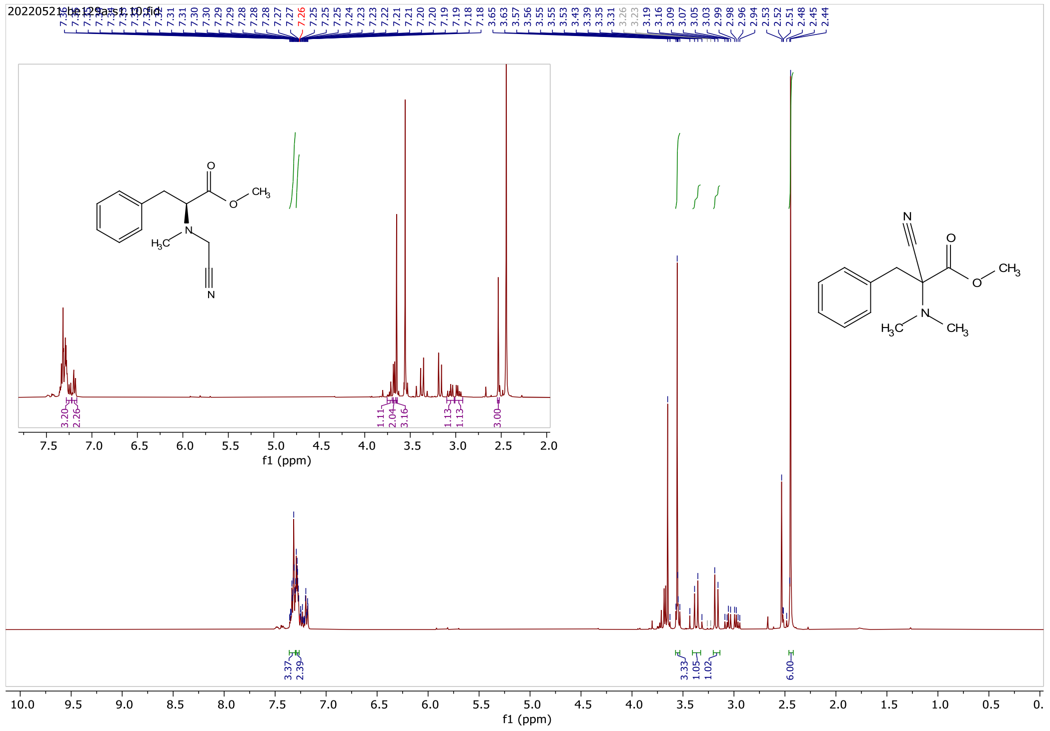

^{13}C NMR of α-nitrile and N-methyl nitrile N,N-dimethyl phenylalanine methyl ester products 2a and 2a’

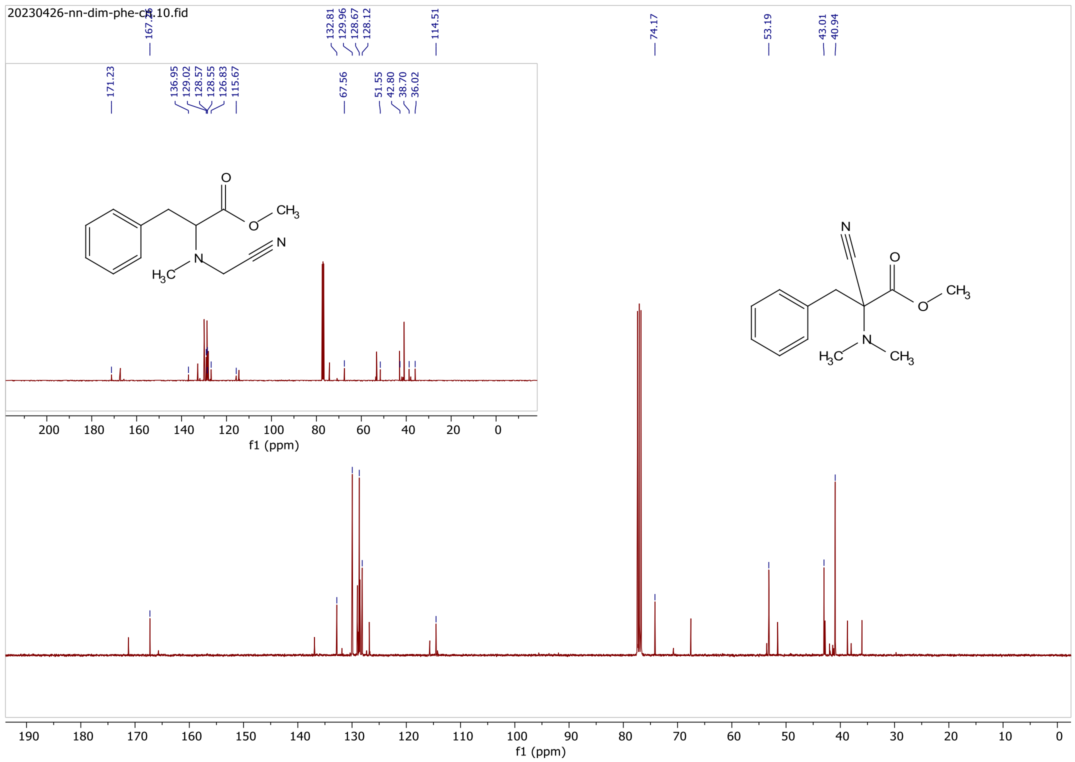

Nitrilation of N,N-dimethyl serine methyl ester.

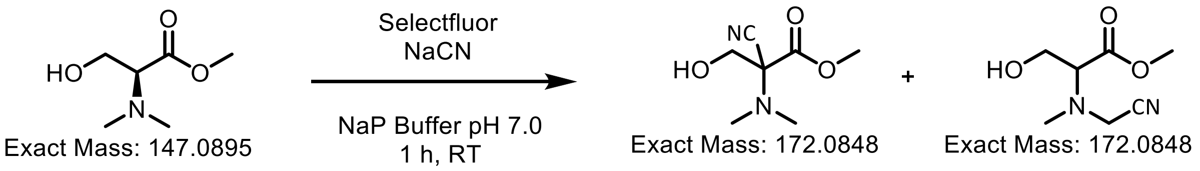

100 mg of N,N-dimethyl serine methyl ester was modified using **general procedure 1**. Concentration of the mixture gave a residue, which was purified by silica gel column chromatography (eluent: 100 % CH_2Cl_2) to give the nitrile products of N,N-dimethyl serine methyl ester which could not be separated by silica gel column chromatography. % Yields were determined by NMR (72 % vs 28 %).

^{1}H NMR of α-nitrile N,N-dimethyl serine methyl ester (400 MHz, CD_3OD) δ 4.01 (d, J = 10.6 Hz, 1H), 3.91 (d, J = 10.6 Hz, 1H), 3.86 (s, 3H), 2.30 (s, 6H). **^{13}C NMR** (101 MHz, CD_3OD) δ 172.49, 116.14, 61.01, 52.42, 35.00, 29.85.

^{1}H NMR of N-methyl nitrile N,N-dimethyl serine methyl ester (400 MHz, CD_3OD) δ 3.84 (d, J = 2.0 Hz, 2H), 3.82 (d, J = 4.6 Hz, 1H), 3.79 (d, J = 5.1 Hz, 2H), 3.74 (s, 3H), 3.45 (t, J = 6.1 Hz, 1H), 2.49 (s, 3H). **^{13}C NMR** (101 MHz, CD_3OD) δ 163.58, 114.24, 89.65, 59.13, 52.21, 47.55, 25.90.

^{1}H NMR of α-nitrile and N-methyl nitrile N,N-dimethyl serine methyl ester products

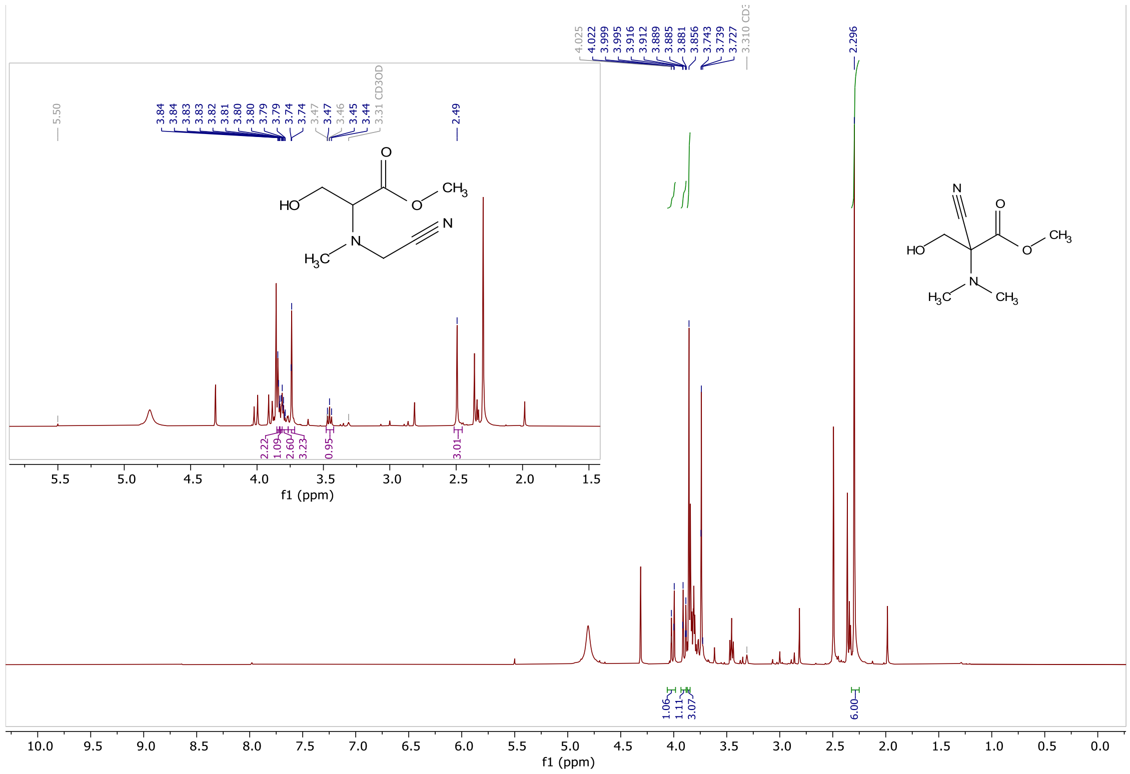

^{13}C NMR of α-nitrile and N-methyl nitrile N,N-dimethyl serine methyl ester products

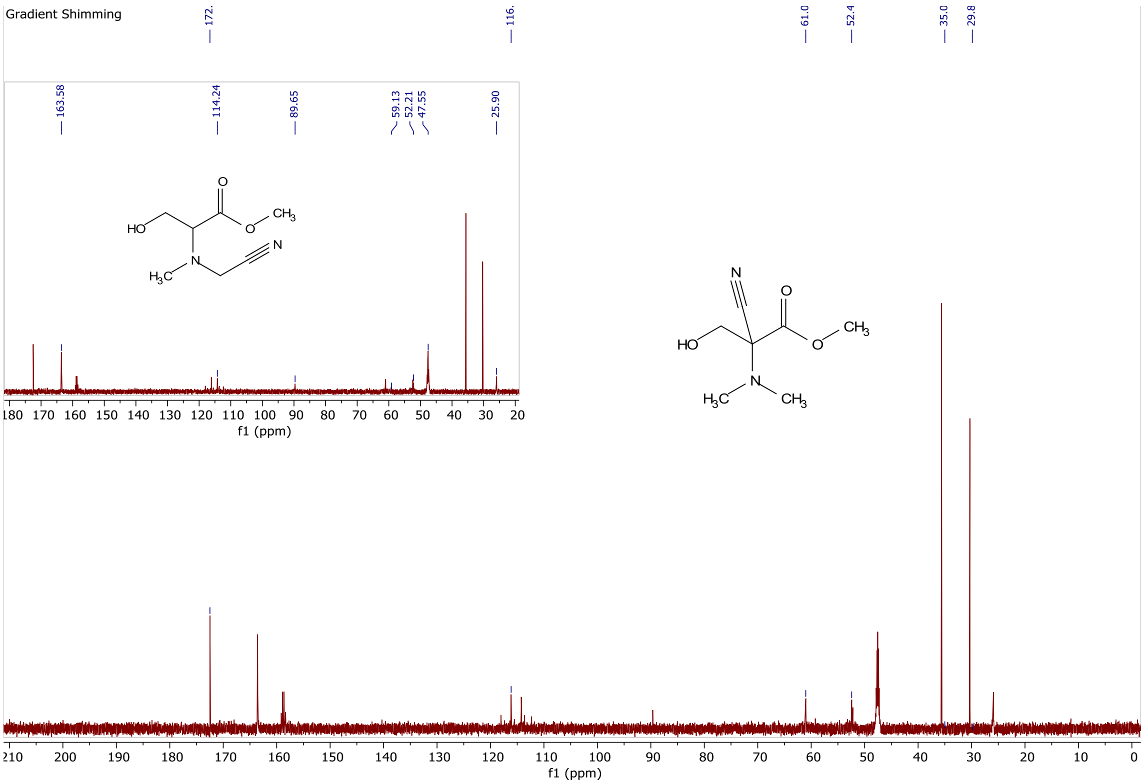

Nitrilation of N,N-dimethyl lysine methyl ester.

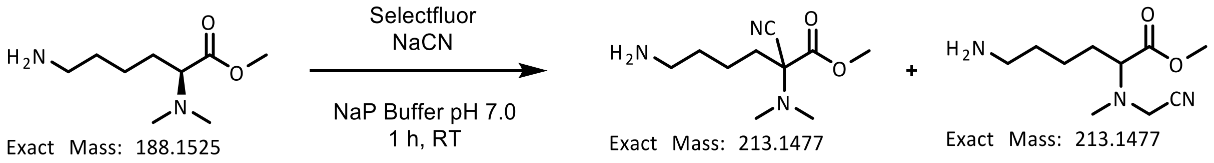

100 mg of N,N-dimethyl lysine methyl ester was modified using **general procedure 1**. Concentration of the mixture gave a residue, which was purified by silica gel column chromatography (eluent: 5 % methanol in CH_2Cl_2) to give the nitrile products of N,N-dimethyl lysine methyl ester which could not be separated by silica gel column chromatography. % Yields were determined by NMR (82 % vs 18 %).

^{1}H NMR of α-nitrile N,N-dimethyl lysine methyl ester (400 MHz, CD_3OD) δ 3.88 – 3.82 (m, 2H), 3.75 (d, *J* = 1.6 Hz, 2H), 3.67 (s, 3H), 3.46 (d, *J* = 4.8 Hz, 2H), 3.46 – 3.38 (m, 2H), 2.16 (s, 6H). **^{13}C NMR** (101 MHz, CD_3OD) δ 172.49, 114.53, 89.64, 52.20, 35.59, 30.27, 21.73, 21.56, 11.42.

^{1}H NMR of N-methyl nitrile N,N-dimethyl lysine methyl ester (400 MHz, CD_3OD) δ 3.88 – 3.85 (m, 2H), 3.85 (s, 3H), 3.82 – 3.74 (m, 2H), 3.75 – 3.71 (m, 2H), 3.40 – 3.35 (m, 2H), 2.86 (t, *J* = 1.2 Hz, 1H), 2.32 (s, 3H). **^{13}C NMR** (101 MHz, CD_3OD) δ 159.19, 116.43, 79.38, 47.11, 43.02, 38.77, 28.07, 25.88, 25.71, 23.91.

^{1}H NMR of α-nitrile and N-methyl nitrile N,N-dimethyl lysine methyl ester products

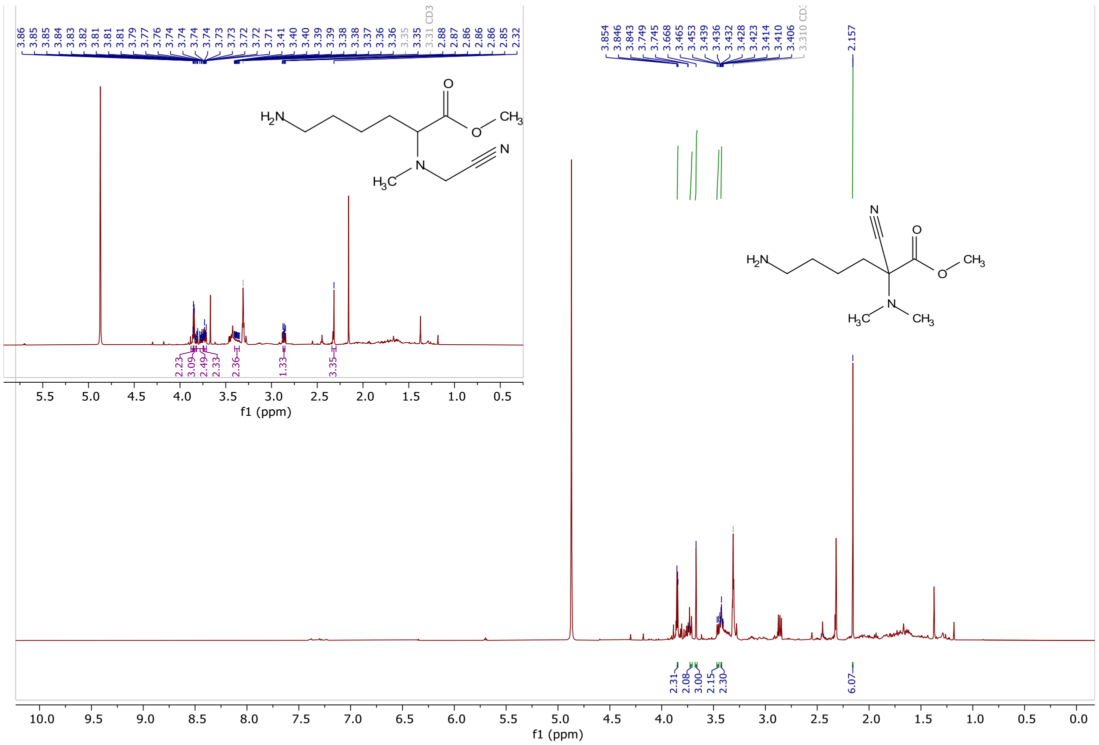

^{13}C NMR of α-nitrile and N-methyl nitrile N,N-dimethyl lysine methyl ester products

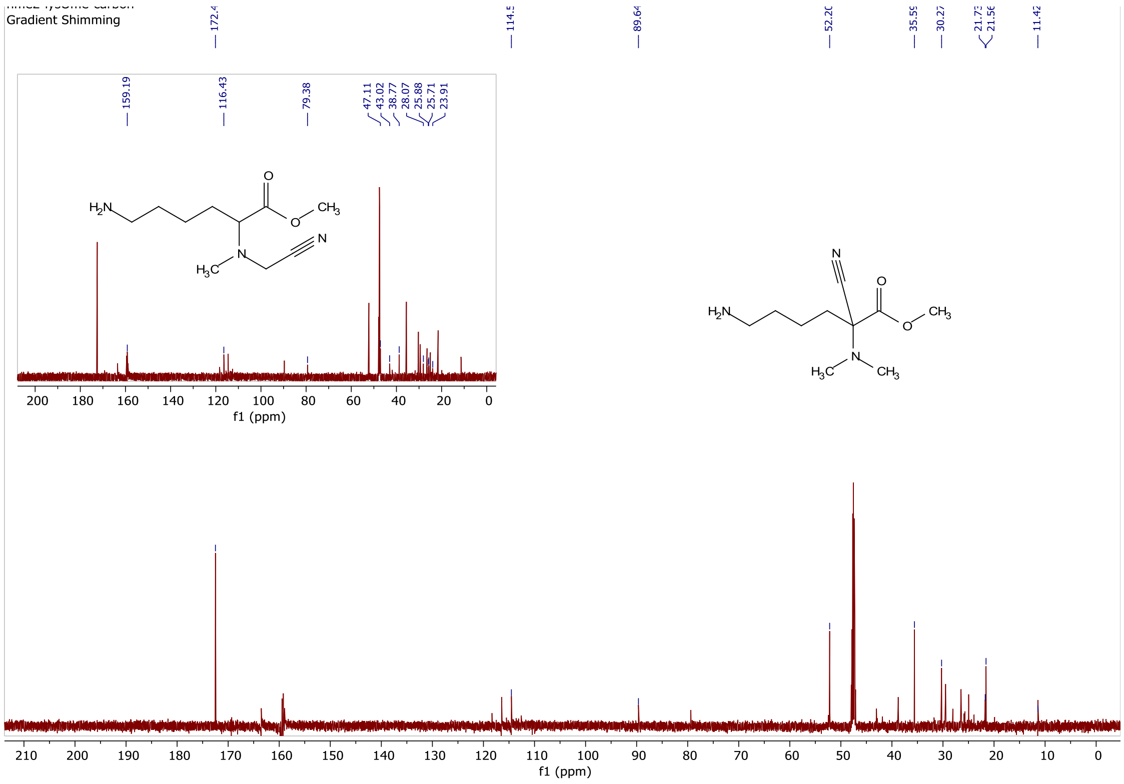

Nitrilation of dimethyl N,N-dimethyl-L-aspartate.

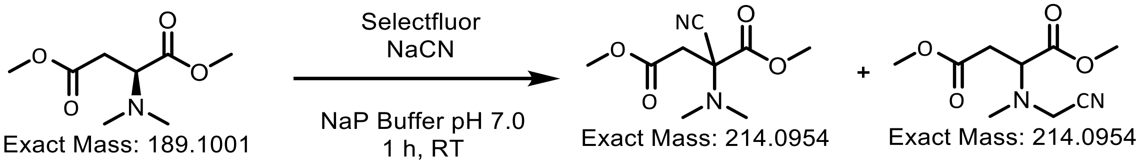

100 mg of dimethyl N,N-dimethyl-aspartate was modified using **general procedure 1**. Concentration of the mixture gave a residue, which was purified by silica gel column chromatography (eluent: 3 % methanol in CH_2Cl_2) to give the nitrile products of dimethyl N,N-dimethyl aspartate nitrile products which could not be separated by silica gel column chromatography. % Yields were determined by NMR (74 % vs 26 %).

^{1}H NMR of α-nitrile dimethyl N,N-dimethyl-L-aspartate (400 MHz, CD_3OD) δ 3.84 (s, 3H), 3.71 (s, 3H), 3.25 (d, *J* = 16.7 Hz, 1H), 3.15 (d, *J* = 16.7 Hz, 1H), 2.33 (s, 6H). **^{13}C NMR** (101 MHz, CD_3OD) δ 172.49, 110.51, 52.55, 41.79, 39.72, 26.71, 11.38.

^{1}H NMR of N-methyl nitrile dimethyl N,N-dimethyl-L-aspartate (400 MHz, CD_3OD) δ 3.87 (s, 2H), 3.78 (d, *J* = 1.2 Hz, 1H), 3.73 (s, 3H), 3.67 (s, 3H), 2.71 (t, *J* = 7.3 Hz, 1H), 2.65 (t, *J* = 7.3 Hz, 1H), 2.45 (s, 3H).

^{1}H NMR of α-nitrile and N-methyl nitrile dimethyl N,N-dimethyl-L-aspartate products

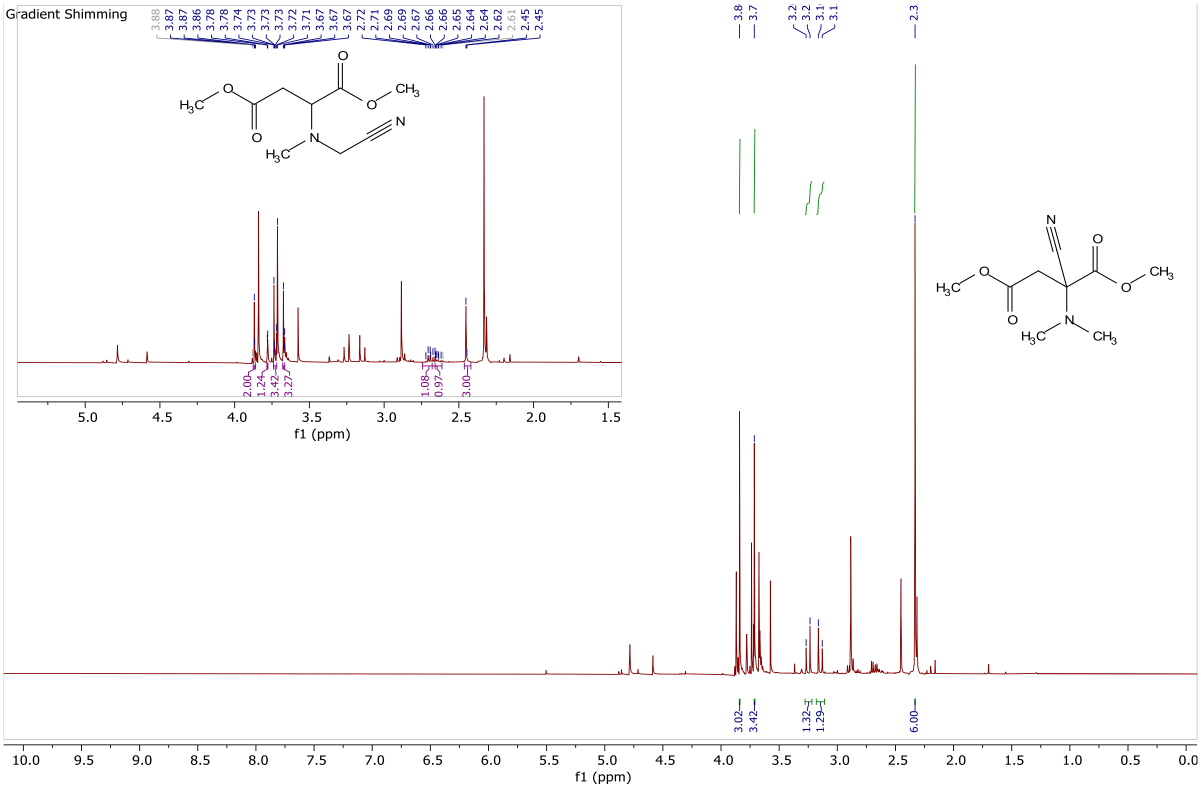

^{13}C NMR of α-nitrile nitrile dimethyl N,N-dimethyl-L-aspartate product

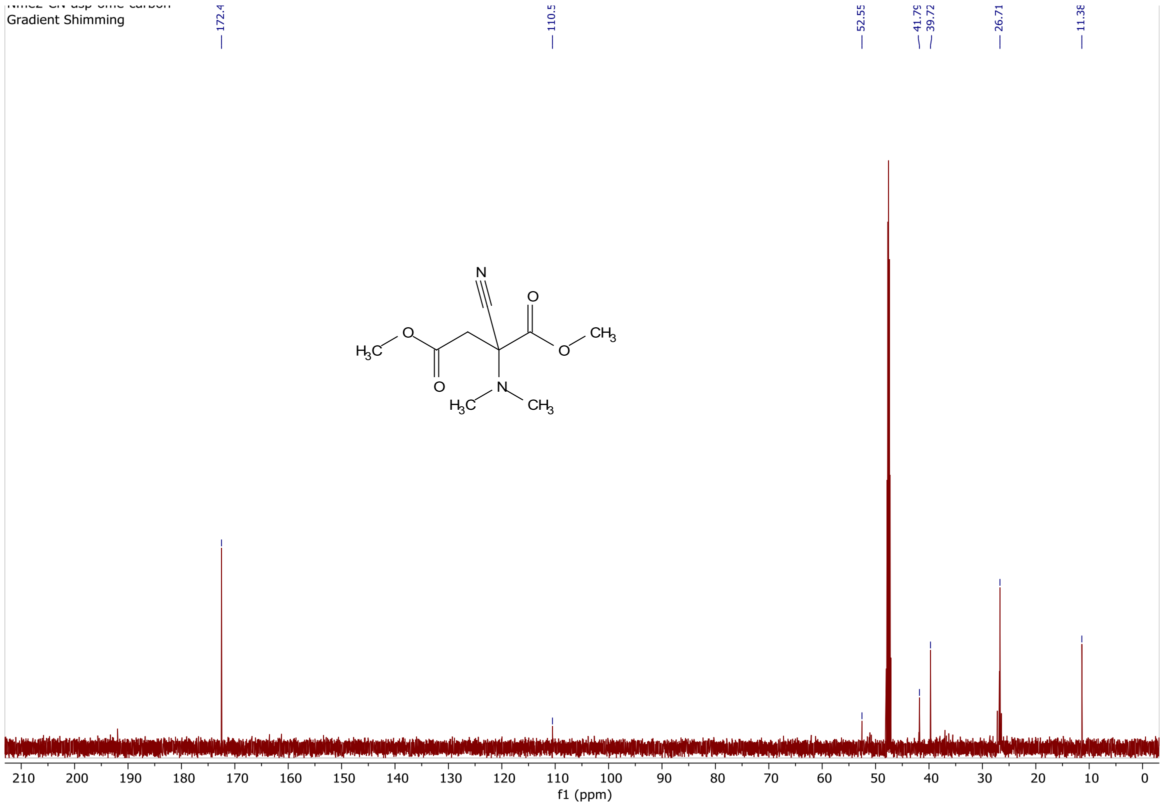

Nitrilation of N,N-dimethyl methionine methyl ester.

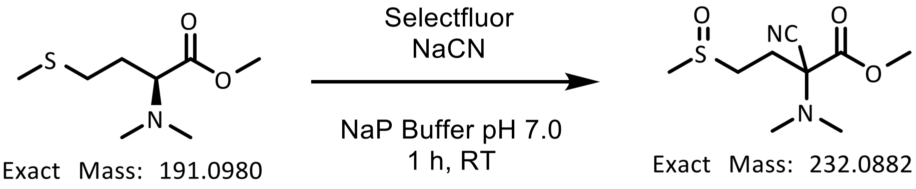

100 mg of N,N-dimethyl methionine methyl ester was modified using **general procedure 1**. Concentration of the mixture gave a residue, which was purified by silica gel column chromatography (eluent: 100 % CH_2Cl_2) to give the nitrile products of N,N-dimethyl methionine methyl ester which could not be separated by silica gel column chromatography. % Yields were determined by NMR (81 % vs 19 %).

^{1}H NMR of α-nitrile N,N-dimethyl methionine methyl ester (600 MHz, CD_3OD) δ 3.86 (s, 3H), 2.67 – 2.58 (m, 2H), 2.33 (s, 6H), 2.33 – 2.30 (m, 2H), 2.11 (s, 3H). **^{13}C NMR** (151 MHz, CD_3OD) δ 163.28, 110.00, 57.30, 35.60, 32.50, 30.32, 20.02, 13.91.

^{1}H NMR of N-methyl nitrile N,N-dimethyl methionine methyl ester (600 MHz, CD_3OD) δ 3.88 (s, 2H), 3.86 (s, 3H), 3.86 – 3.84 (m, 1H), 2.67 – 2.58 (m, 2H), 2.36 (s, 3H), 2.33 – 2.30 (m, 2H), 2.11 (s, 3H). **^{13}C NMR** (151 MHz, CD_3OD) δ 172.49, 115.18, 81.82, 60.12, 53.83, 51.03, 29.80, 28.94, 11.34.

^{1}H NMR of α-nitrile and N-methyl nitrile N,N-dimethyl methionine methyl ester products

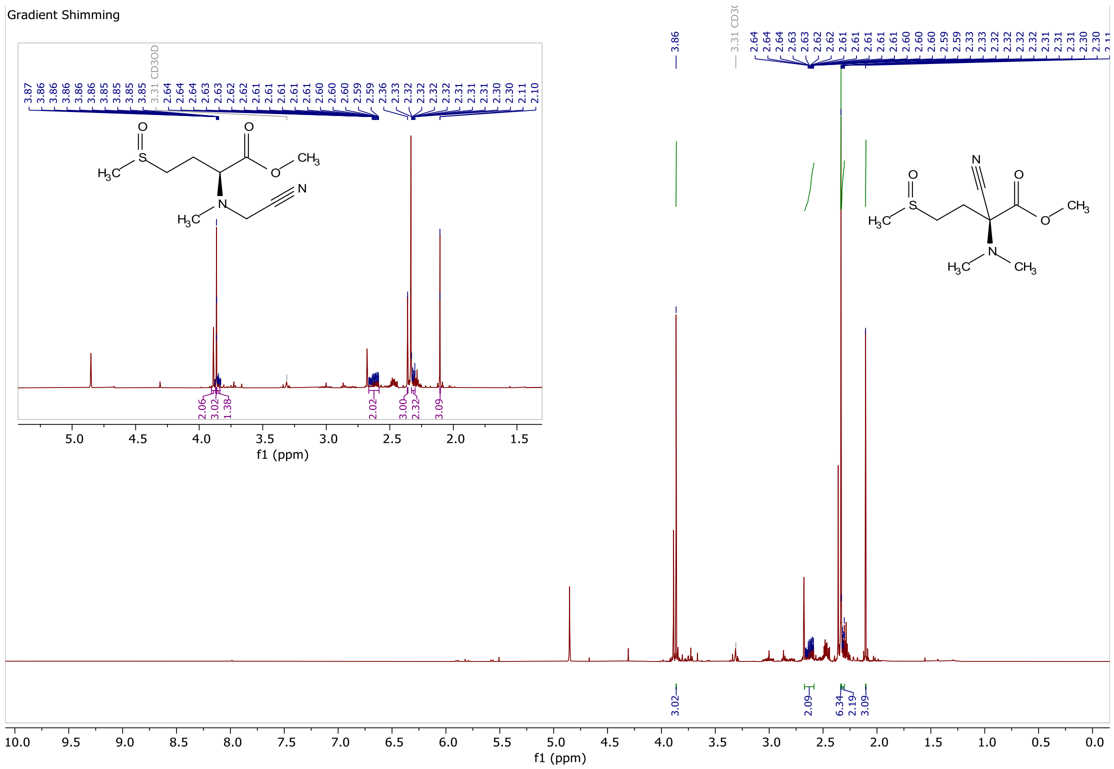

^{13}C NMR of α-nitrile and N-methyl nitrile N,N-dimethyl methionine methyl ester products

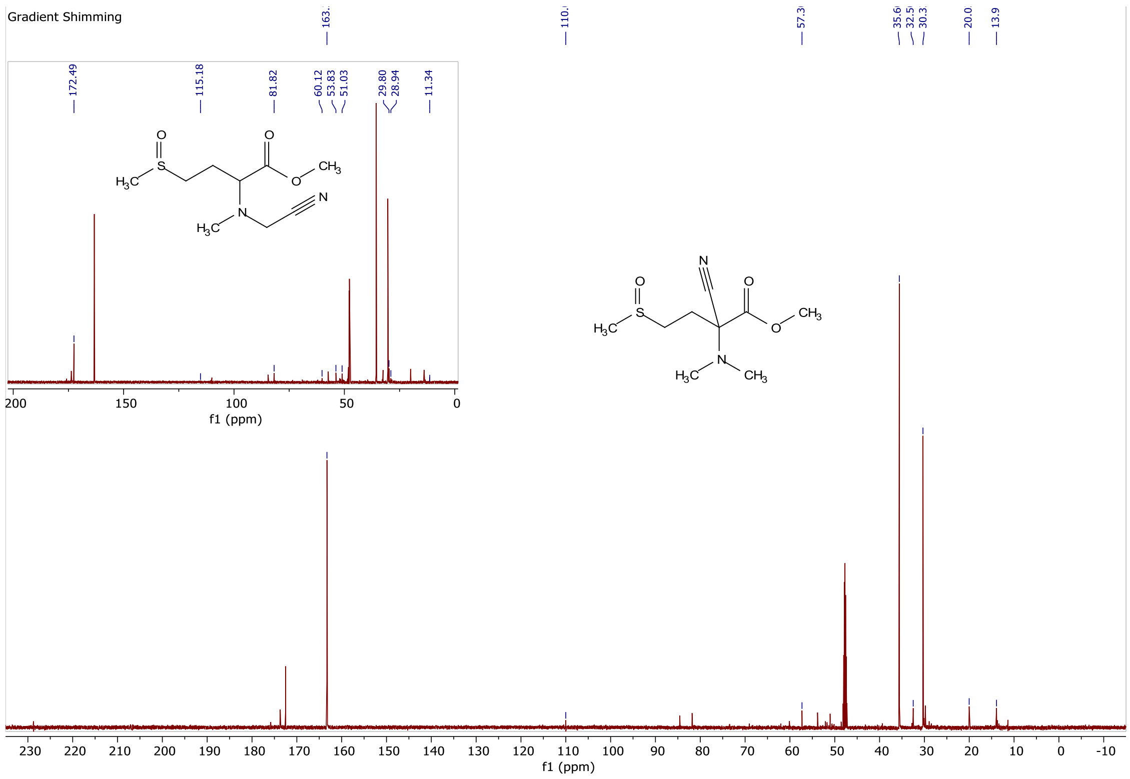

Nitrilation of N,N-dimethyl cysteine phenylalanine amide.

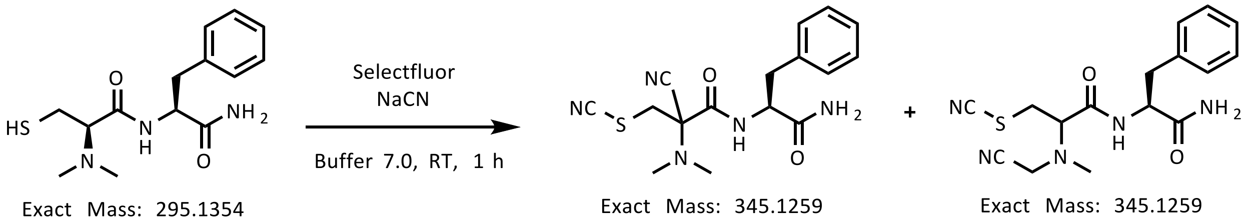

100 mg of N,N-dimethyl cysteine phenylalanine amide was modified using **general procedure 1**. Concentration of the mixture gave a residue, which was purified by silica gel column chromatography (eluent: 2 % CH_2Cl_2) to give the nitrile products of N,N-dimethyl cysteine phenylalanine amide which could not be separated by silica gel column chromatography. % Yields were determined by NMR (70 % vs 30 %).

^{1}H NMR of α-nitrile N,N-dimethyl cysteine phenylalanine amide (400 MHz, CD_3OD) δ 7.31 – 7.28 (m, 3H), 7.28 – 7.26 (m, 2H), 4.76 – 4.71 (m, 1H), 3.28 – 3.21 (m, 2H), 3.05 – 2.91 (m, 2H), 2.33 (s, 2H), 2.03 (s, 6H). **^{13}C NMR** (101 MHz, CD_3OD) δ 172.49, 167.17, 129.77, 128.17, 127.64, 126.25, 115.82, 114.09, 67.37, 52.24, 42.12, 39.73, 35.49.

^{1}H NMR of N-methyl nitrile N,N-dimethyl cysteine phenylalanine amide (400 MHz, CD_3OD) δ 7.28 – 7.26 (m, 3H), 7.23 – 7.18 (m, 2H), 4.71 – 4.63 (m, 1H), 3.82 – 3.58 (m, 2H), 3.45 – 3.40 (m, 1H), 3.26 – 3.20 (m, 2H), 2.33 (s, 2H), 2.12 (s, 3H). **^{13}C NMR** (101 MHz, CD_3OD) δ 171.36, 166.02, 133.17, 129.89, 128.23, 127.82, 114.09, 110.00, 74.33, 50.39, 41.90, 41.49, 40.16, 37.20.

^{1}H NMR of α-nitrile and N-methyl nitrile N,N-dimethyl phenylalanine-cysteine amide

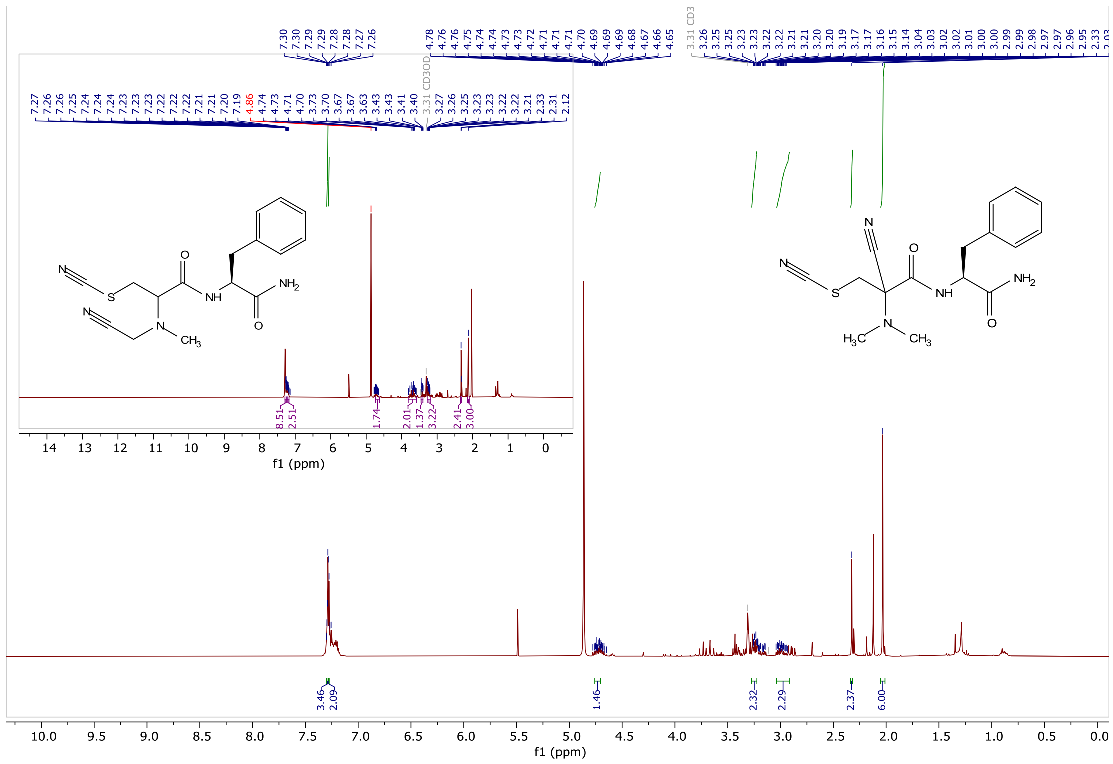

^{13}C NMR of α-nitrile and N-methyl nitrile N,N-dimethyl phenylalanine-cysteine amide

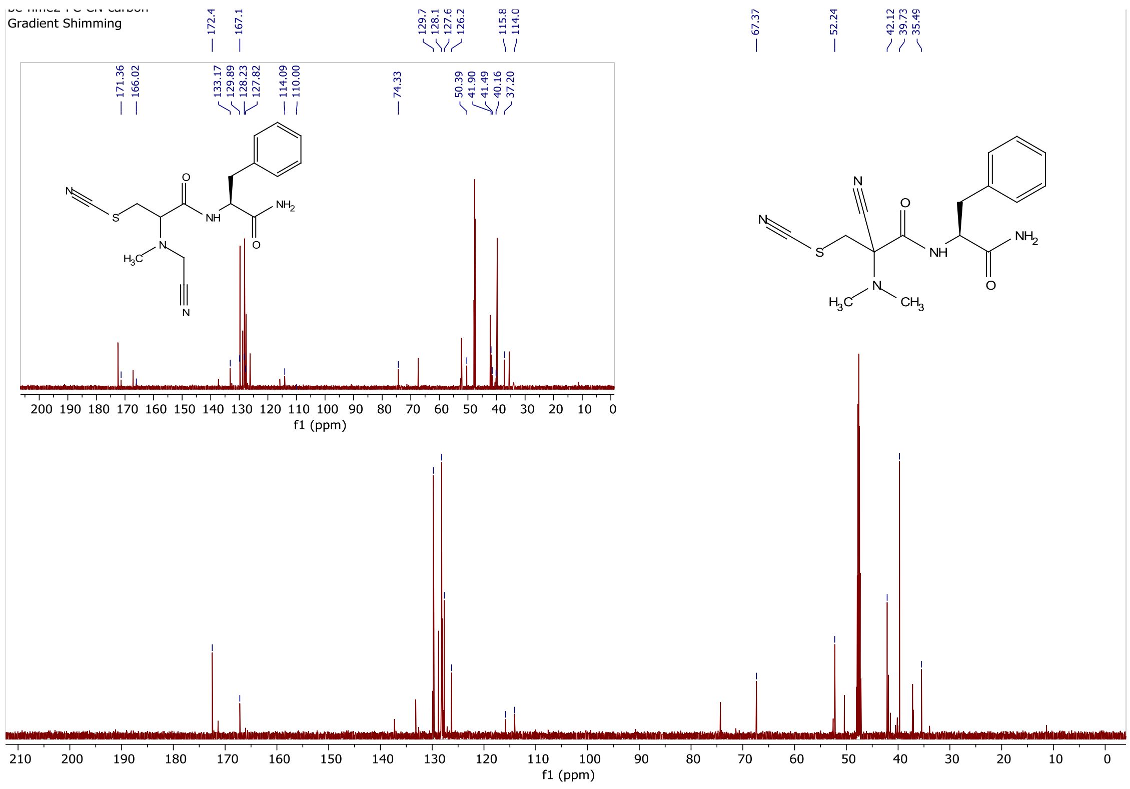

Cyanation of N,N-dimethyl asparagine phenylalanine amide.

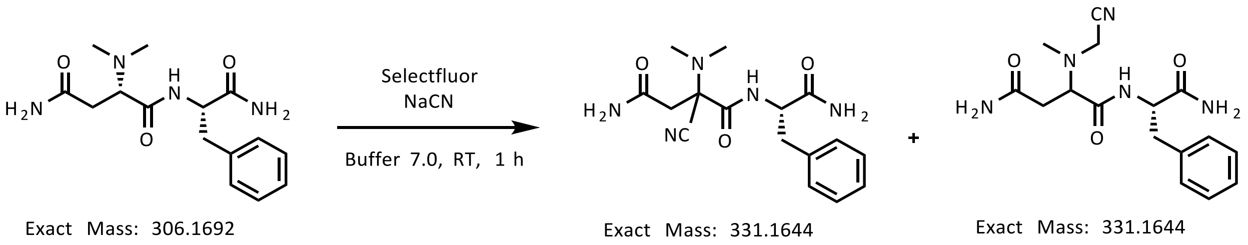

100 mg of N,N-dimethyl asparagine phenylalanine amide was modified using **general procedure 1**. Concentration of the mixture gave a residue, which was purified by silica gel column chromatography (eluent: 2 % CH_2Cl_2) to give the nitrile products of N,N-dimethyl asparagine phenylalanine amide which could not be separated by silica gel column chromatography. % Yields were determined by NMR (76 % vs 24 %).

^{1}H NMR of α-nitrile N,N-dimethyl asparagine phenylalanine amide (400 MHz, CD_3OD) δ 7.30 (d, *J* = 1.9 Hz, 5H), 4.71 (d, *J* = 4.6 Hz, 1H), 2.88 – 2.82 (m, 2H), 2.73 – 2.65 (m, 2H), 2.06 (s, 6H). **^{13}C NMR** (101 MHz, CD_3OD) δ 174.99, 174.22, 171.00, 137.57, 128.91, 128.24, 126.46, 115.97, 62.96, 54.16, 41.42, 37.54, 33.14

^{1}H NMR of N-methyl nitrile N,N-dimethyl asparagine phenylalanine amide ^{1}H NMR (400 MHz, CD_3OD) δ 7.28 – 7.19 (m, 5H), 4.69 (d, *J* = 4.6 Hz, 1H), 3.61 – 3.57 (m, 1H), 3.40 – 3.34 (m, 2H), 3.23 (d, *J* = 18.8 Hz, 2H), 2.54 – 2.46 (m, 2H), 1.94 (s, 3H).

^{1}H NMR of α-nitrile and N-methyl nitrile N,N-dimethyl asparagine phenylalanine amide

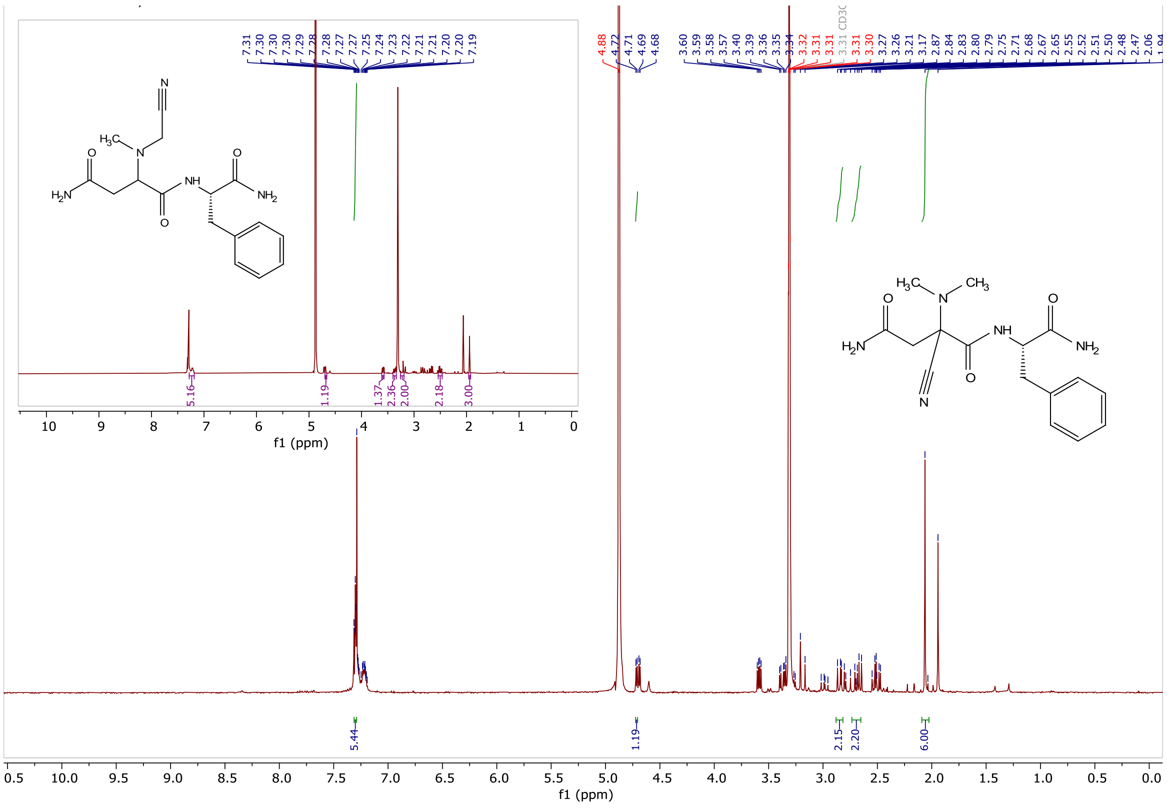

^{13}C NMR of α-nitrile N,N-dimethyl asparagine phenylalanine amide product

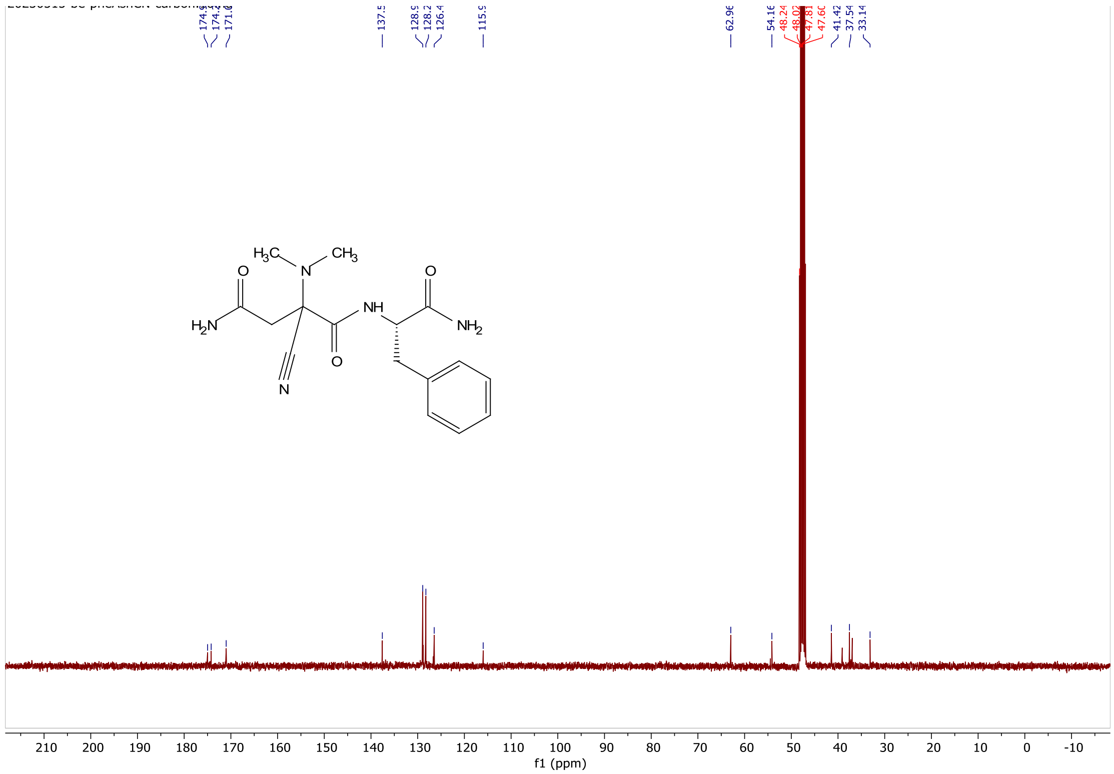

Nitrilation of N-methyl proline methyl ester.

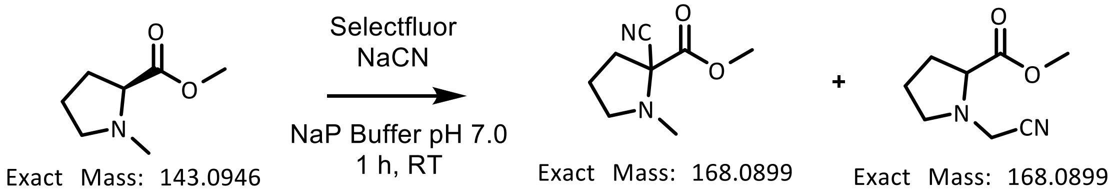

100 mg of N-methyl proline methyl ester was modified using **general procedure 1**. Concentration of the mixture gave a residue, which was purified by silica gel column chromatography (eluent: 2 % methanol in CH_2Cl_2) to give the nitrile products of N,N-dimethyl methionine methyl ester which could not be separated by silica gel column chromatography. % Yields were determined by NMR (82 % vs 18 %).

^{1}H NMR of α-nitrile N-methyl proline methyl ester (600 MHz, CD_3OD) δ 3.86 – 3.84 (m, 3H), 2.71 – 2.49 (m, 2H), 2.48 – 2.41 (m, 2H), 2.40 (d, J = 0.7 Hz, 3H), 2.01 – 1.91 (m, 2H). **^{13}C NMR** (151 MHz, CD_3OD) δ 163.41, 110.07, 87.87, 73.83, 55.44, 51.50, 35.85, 25.50.

^{1}H NMR of N-methyl nitrile N-methyl proline methyl ester (600 MHz, CD_3OD) δ 4.30 (d, J = 0.8 Hz, 2H), 3.87 – 3.85 (m, 3H), 3.21 – 3.15 (m, 1H), 2.63 – 2.50 (m, 2H), 2.49 – 2.42 (m, 2H), 2.07 – 1.98 (m, 2H).

^{1}H NMR of α-nitrile and N-methyl nitrile N-methyl proline methyl ester products

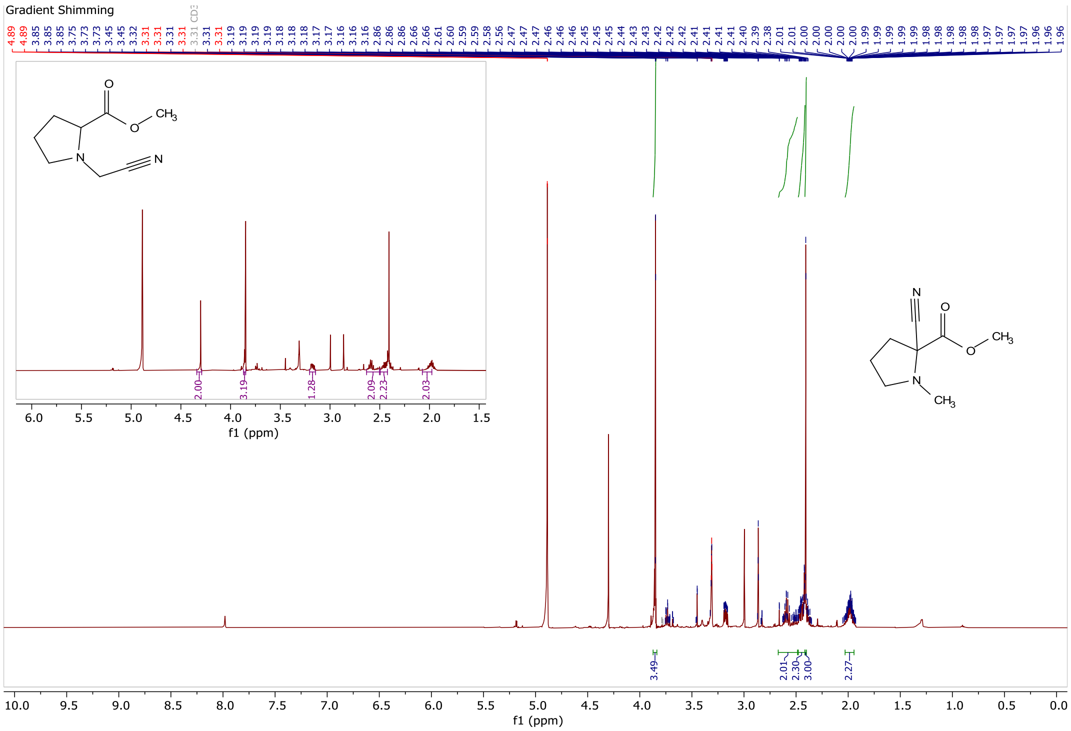

^{13}C NMR of α-nitrile N-methyl proline methyl ester products

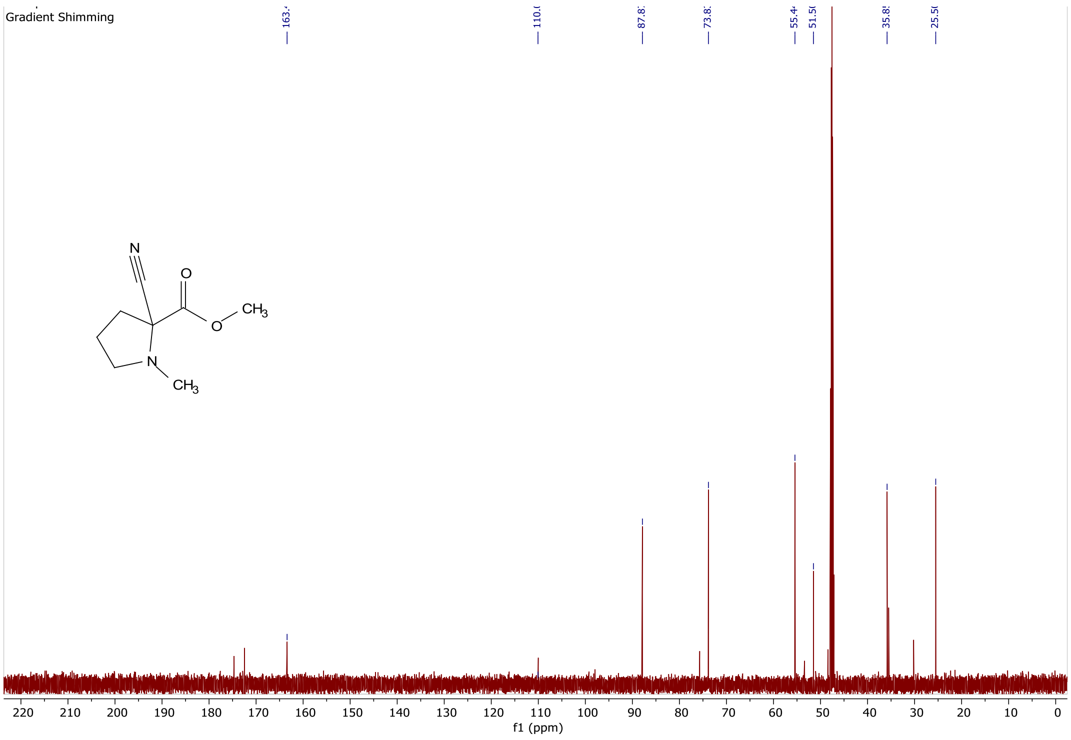

Nitrilation of N,N-dimethyl leucine methyl ester.

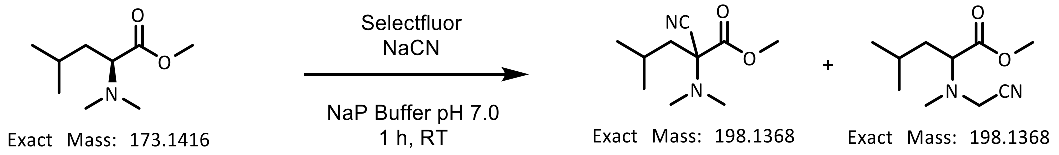

100 mg of N,N-dimethyl leucine methyl ester was modified using **general procedure 1**. Concentration of the mixture gave a residue, which was purified by silica gel column chromatography (eluent: 100 % CH_2Cl_2) to give the nitrile products of N,N-dimethyl leucine methyl ester which could not be separated by silica gel column chromatography. % Yields were determined by NMR (81 % vs 19 %).

^{1}H NMR of α-nitrile N,N-dimethyl leucine methyl ester (600 MHz, $CDCl_3$) δ 3.85 (s, 3H), 2.32 (s, 6H), 1.90 (dd, J = 6.4, 3.6 Hz, 2H), 1.65 – 1.58 (m, 1H), 1.06 (d, J = 6.7 Hz, 3H), 0.92 (d, J = 6.6 Hz, 3H). **^{13}C NMR of α-nitrile N,N-dimethyl leucine methyl ester** (151 MHz, $CDCl_3$) δ 168.09, 115.14, 71.21, 53.39, 45.11, 40.63, 25.37, 22.99.

^{1}H NMR of N-methyl nitrile N,N-dimethyl leucine methyl ester (600 MHz, $CDCl_3$) δ 3.71 (s, 3H), 3.63 (q, J = 17.6 Hz, 2H), 3.33 (t, J = 7.6 Hz, 1H), 2.46 (s, 3H), 1.65 – 1.58 (m, 1H), 1.63 – 1.50 (m, 2H), 0.94 (d, J = 6.5 Hz, 3H), 0.89 (d, J = 6.5 Hz, 3H). **^{13}C NMR of N-methyl nitrile N,N-dimethyl leucine methyl ester** (151 MHz, $CDCl_3$) δ 172.42, 116.15, 63.76, 51.47, 42.25, 38.63, 38.54, 24.75, 22.17.

^{1}H NMR of α-nitrile and N-methyl nitrile N,N-dimethyl leucine methyl ester products

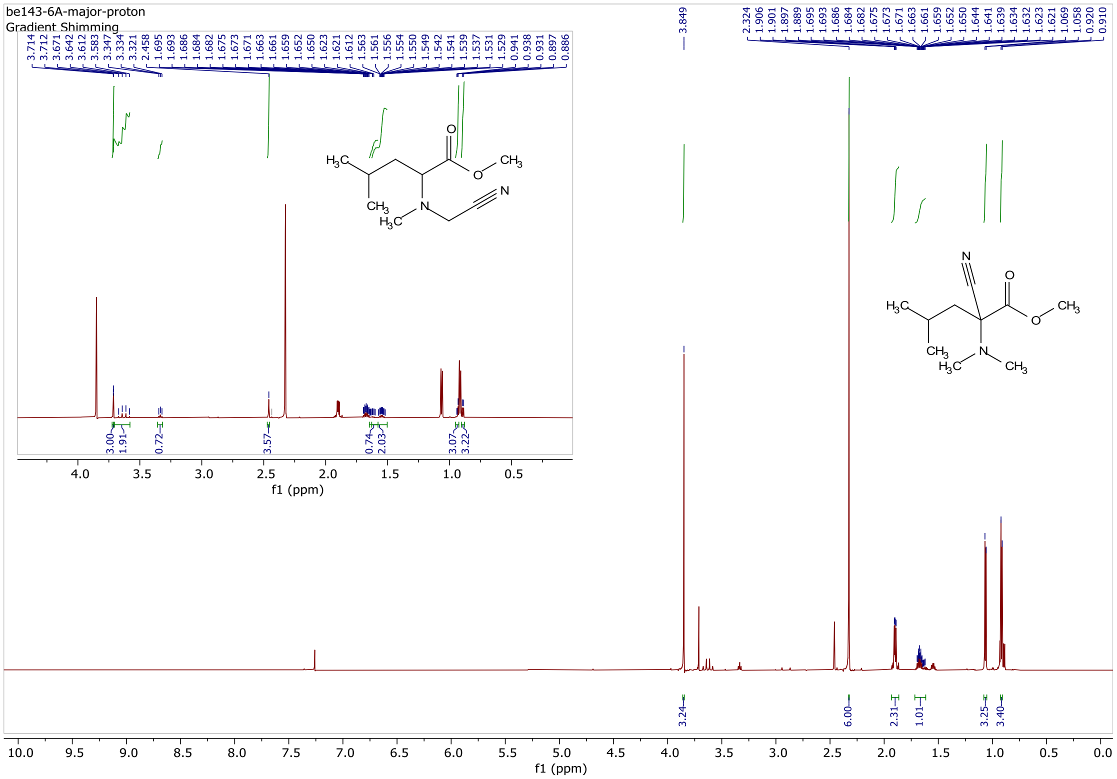

^{13}C NMR of α-nitrile and N-methyl nitrile N,N-dimethyl leucine methyl ester products

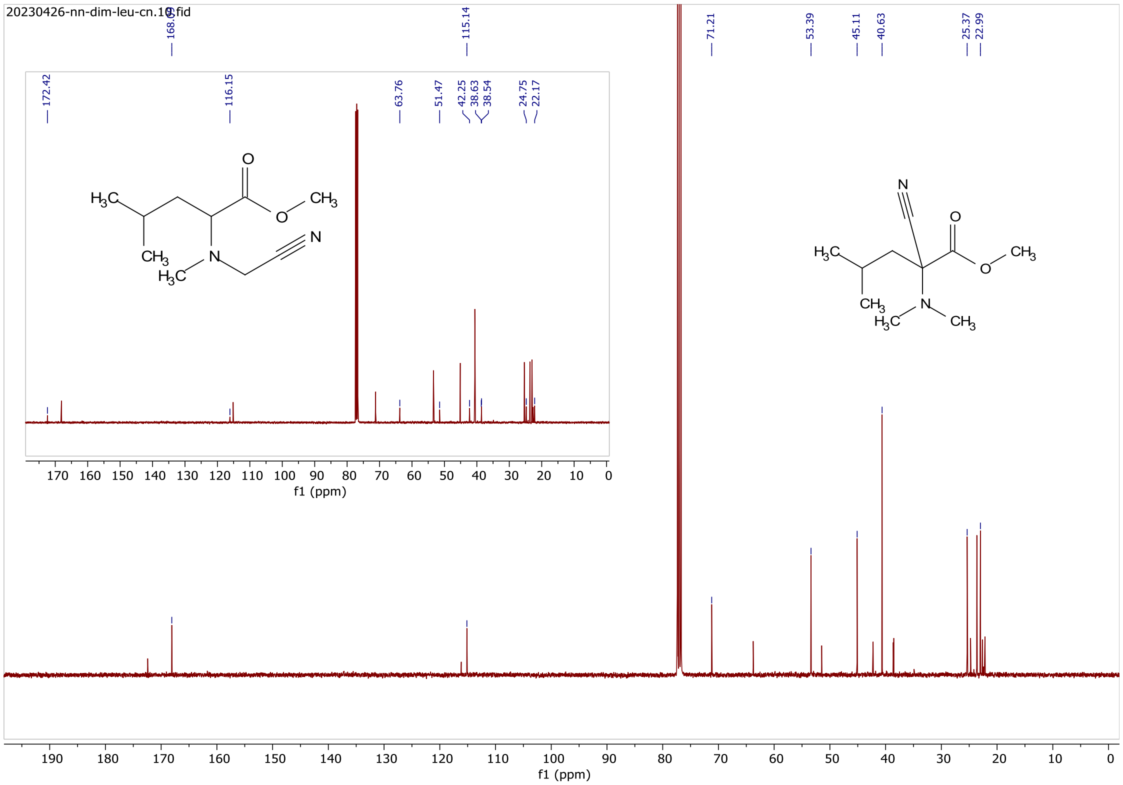

XXVI. Supplementary Figure 19. General procedures for the solid-support synthesis of N-terminal dimethyl peptides.

Fmoc Solid-Phase Peptide Synthesis (Fmoc-SPPS).[1] Peptides were synthesized manually on a 0.25 mm scale using Rink amide resin. Resin was swollen with CH_2Cl_2 for 1 h at room temperature. Fmoc was deprotected using 20 % piperidine in DMF for 5 min to obtain a deprotected peptide-resin. First, Fmoc protected amino acid (1.25 mm/5 equiv.) was coupled using HOAt (1.25 mm/5 equiv.) and DIC (1.25 mm/5 equiv.) in DMF for 15 min at room temperature. Fmoc-protected amino acids (0.75 mm/3 equiv.) were sequentially coupled on the resin using HBTU (0.75 mm/3 equiv.) and DIEA (1.5 mm/6 equiv.) in DMF for 5 min at room temperature.

Dimethylation:

For N-terminal dimethylation, the last amino acid coupled to the resin was deprotected using 20% piperidine in DMF. To the deprocted peptides on resin, 2 mL of 37 % formaldehyde in water solution was added to resin-bound peptides in DMF. 1 g of sodium triacetoxyborohydride was added to the mixture and stirred 10 h. Resin-bound peptides were cleaved from the resin using a cocktail of 95:2.5:2.5, trifluoroacetic acid: water: TES for 2 h. The resin was removed by filtration and the resulting solution was concentrated. The residue was diluted with ACN/water mixture. The resulting solution was purified by HPLC.

XXVII. Supplementary Figure 20. Pan-specific modification of N-terminal dimethyl containing peptides.

Modification of NTMT peptide inhibitor sequence 1s: To 1.0 mg of N,N-dimethyl NTMT peptide **1s** dissolved in 300 μL of 10 mM sodium phosphate buffer (NaP, pH 7.0), was added sodium cyanide (3 equiv.) and selectfluor (2 equiv.). The reaction mixture was stirred for 1 h. Sample was taken from the reaction mixture, injected into LC-MS to monitor the generation of nitrile peptide products (**2s** and **2s'**). The reaction mixture was analyzed by HPLC using method A to determine the % conversion. The nitrile products were not separable by HPLC. The nitrile products were observed with 89 % conversion.

N,N-dimethyl NTMT peptide 1s. LCMS: *m/z* 815.5116 (calcd [M+H]+ = 815.5189), *m/z* 408.2595 (calcd [M+2/2]+ = 408.2558), Purity: >95 % (HPLC analysis at 220 nm). Retention time in HPLC: 5.055

N,N-dimethyl NTMT nitrile peptide products 2s and 2s'. LCMS: *m/z* 840.5077 (calcd [M+H]+ = 840.5203), *m/z* 420.7576 (calcd [M+2/2]+ = 420.7565), Purity: >95 % (HPLC analysis at 220 nm). Retention time in HPLC: 6.454

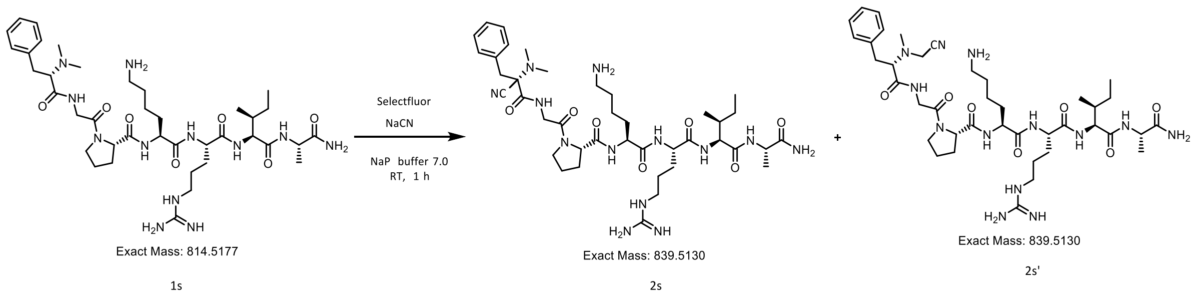

HPLC trace of starting peptide 1s

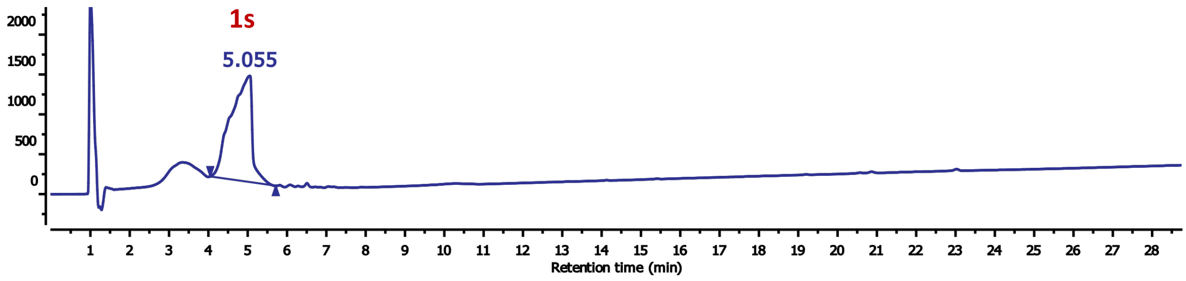

HPLC trace of reaction to 2s and 2s'

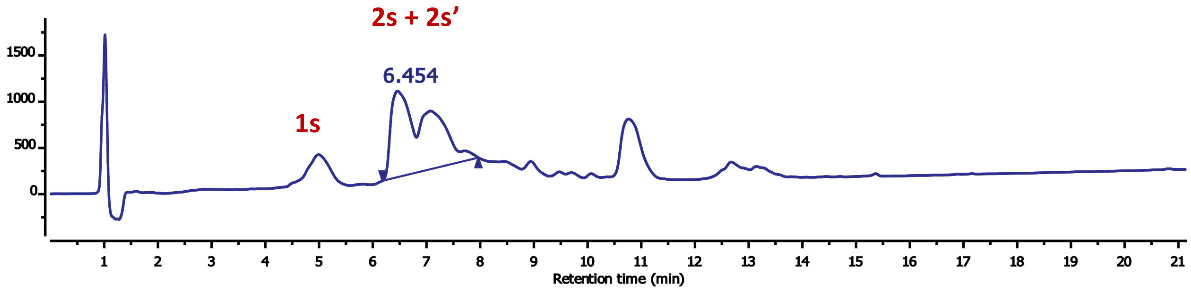

Mass spectrum of 1s

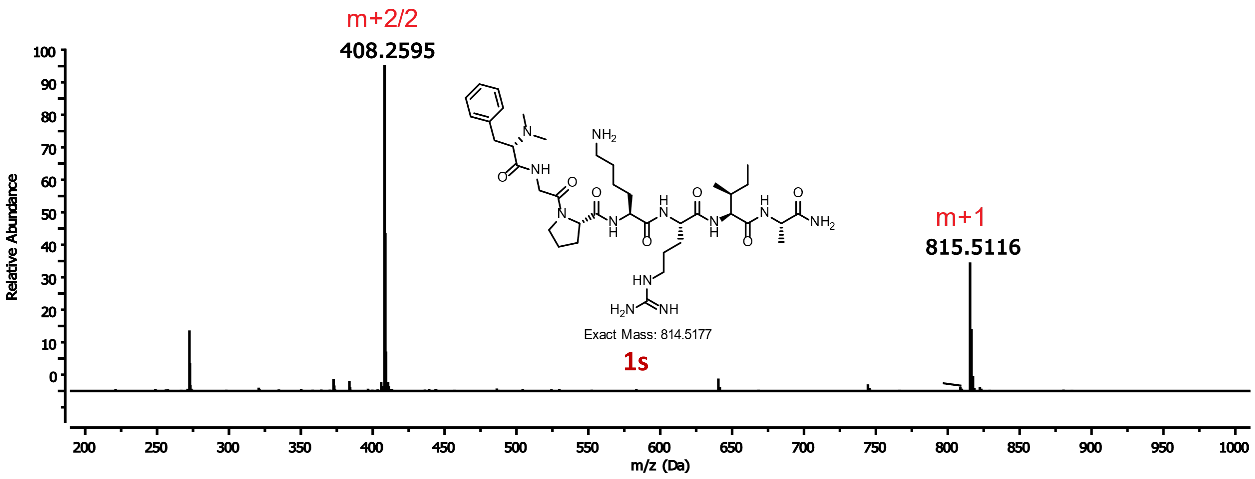

Mass spectrum of 2s and 2s'

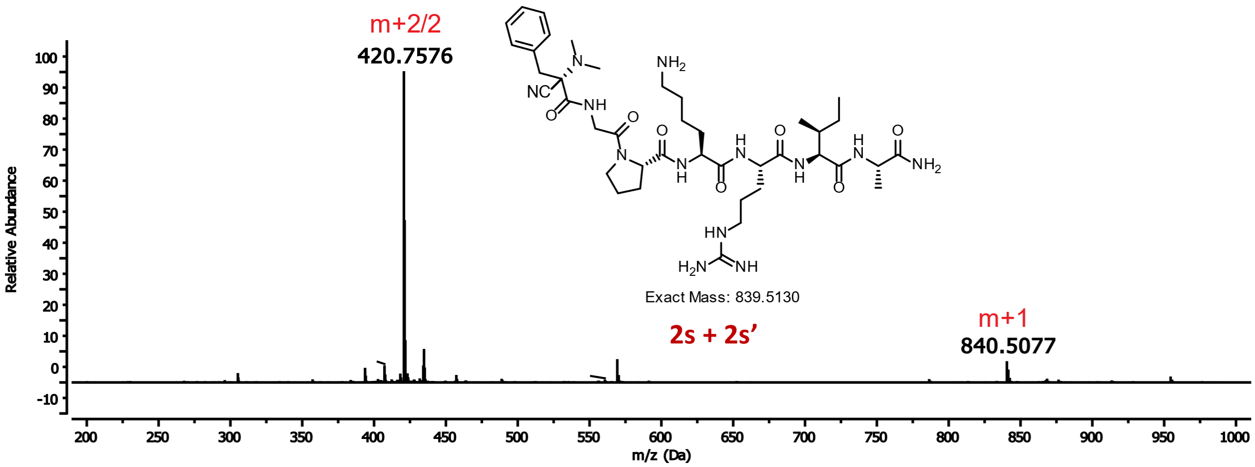

Modification of cytochrome c-557 peptide sequence 1t: To 1.0 mg of N,N-dimethyl cytochrome c-557 peptide **1t** dissolved in 300 µL of 10 mM sodium phosphate buffer (NaP, pH 7.0), was added sodium cyanide (3 equiv.) and selectfluor (2 equiv.). The reaction mixture was stirred for 1 h. Sample was taken from the reaction mixture, injected into LC-MS to monitor the generation of nitrile peptide products (**2t** and **2t'**). The reaction mixture was analyzed by HPLC using method A to determine the % conversion. The nitrile products were not separable by HPLC. The nitrile products were observed with a conversion of >98 %.

N,N-dimethyl cytochrome c-557 nitrile peptide products 2t and 2t'. LCMS: *m/z* 1060.58353 (calcd [M+H]+ = 1060.5899), *m/z* 530.79517 (calcd [M+2/2]+ = 530.7913), Purity: >95 % (HPLC analysis at 220 nm). Retention time in HPLC: 5.259

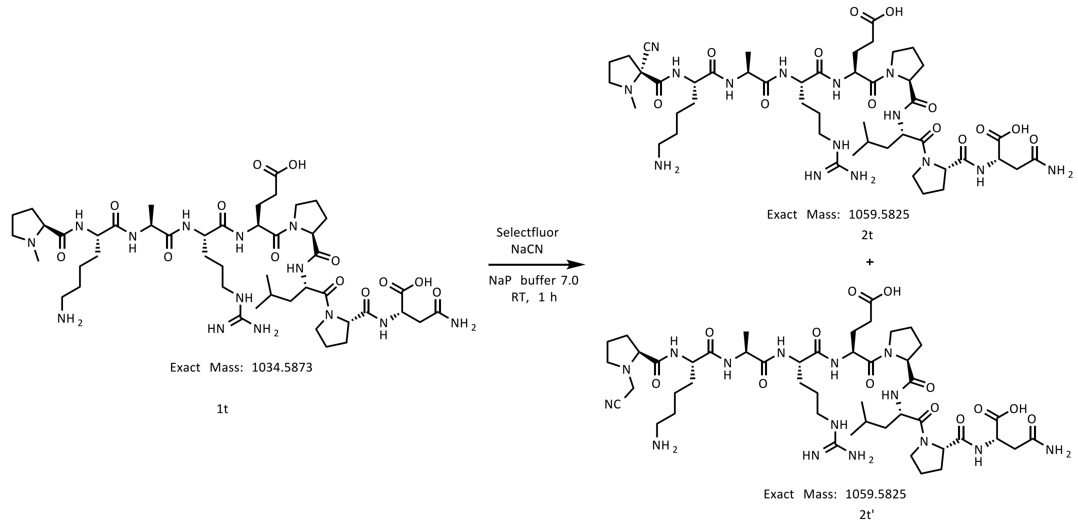

HPLC trace of reaction

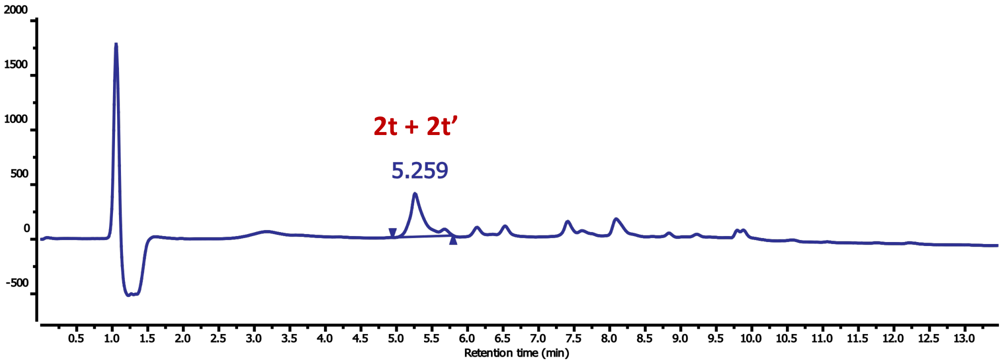

Mass spectrum of peak 5.259

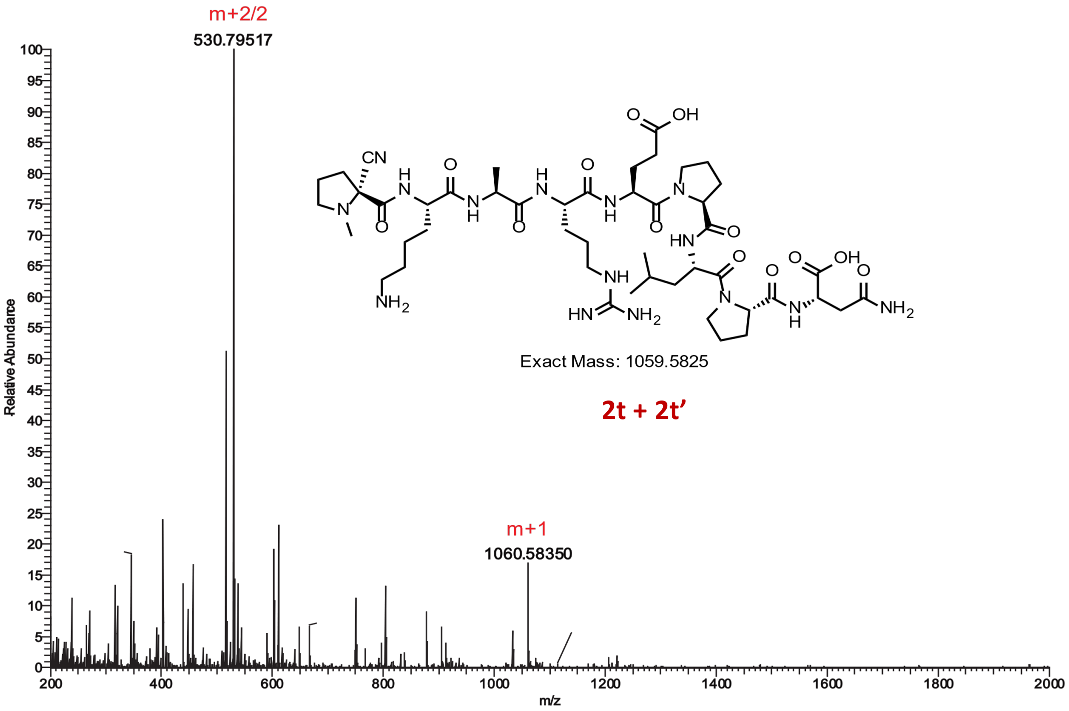

Modification of histone RCC1 peptide sequence 1u: To 1.0 mg of N,N-dimethyl RCC1 peptide **1u** dissolved in 300 µL of 10 mM sodium phosphate buffer (NaP, pH 7.0), was added sodium cyanide (3 equiv.) and selectfluor (2 equiv.). The reaction mixture was stirred for 1 h. Sample was taken from the reaction mixture, injected into LC-MS to monitor the generation of nitrile peptide products (**2u** and **2u**'). The reaction mixture was analyzed by HPLC using method A to determine the % conversion. The nitrile products were not separable by HPLC. The nitrile products were observed with >98 % conversion.

N,N-dimethyl RCC1 nitrile peptide products 2u and 2u'. LCMS: *m/z* 1056.58832 (calcd [M+H]+ = 1056.5797), *m/z* 528.78623 (calcd [M+2/2]+ = 528.7898), *m/z* 352.33743 (calcd [M+3/3]+ = 352.8574), Purity: >95 % (HPLC analysis at 220 nm). Retention time in HPLC: 3.542

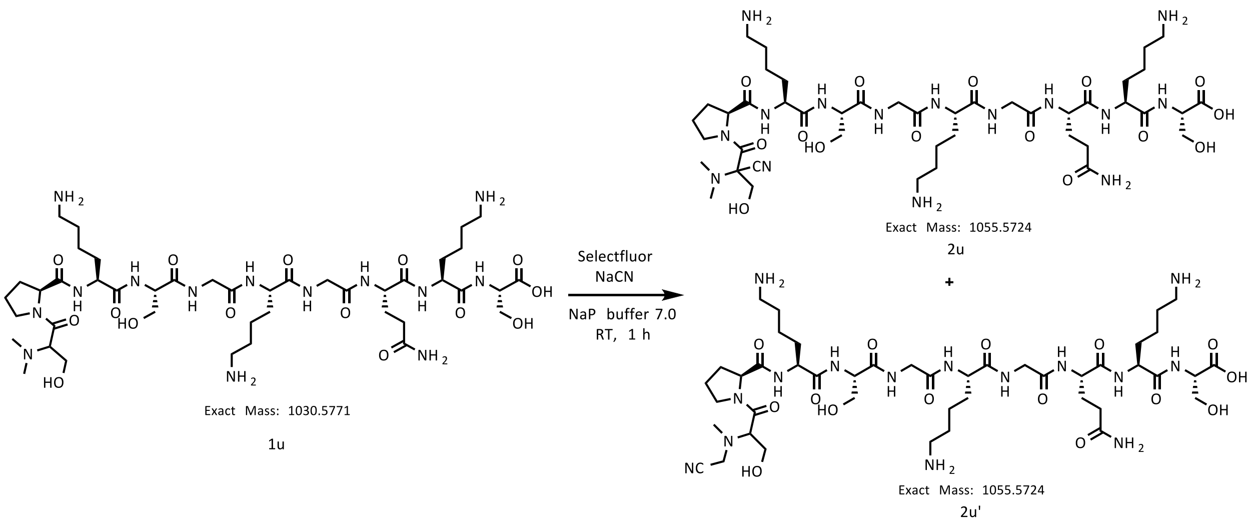

HPLC trace of reaction

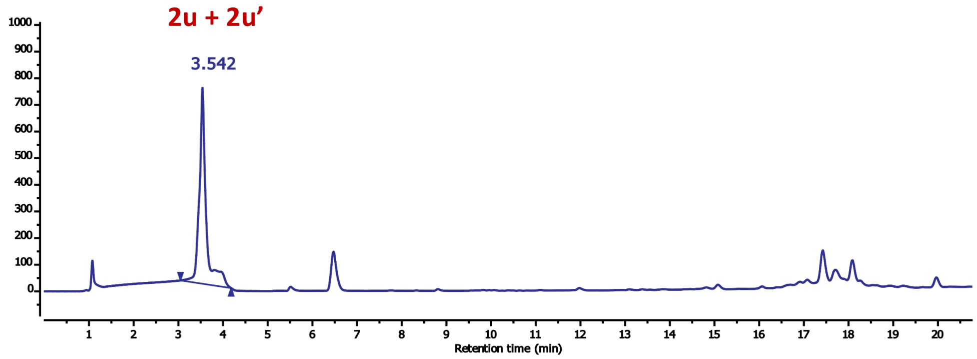

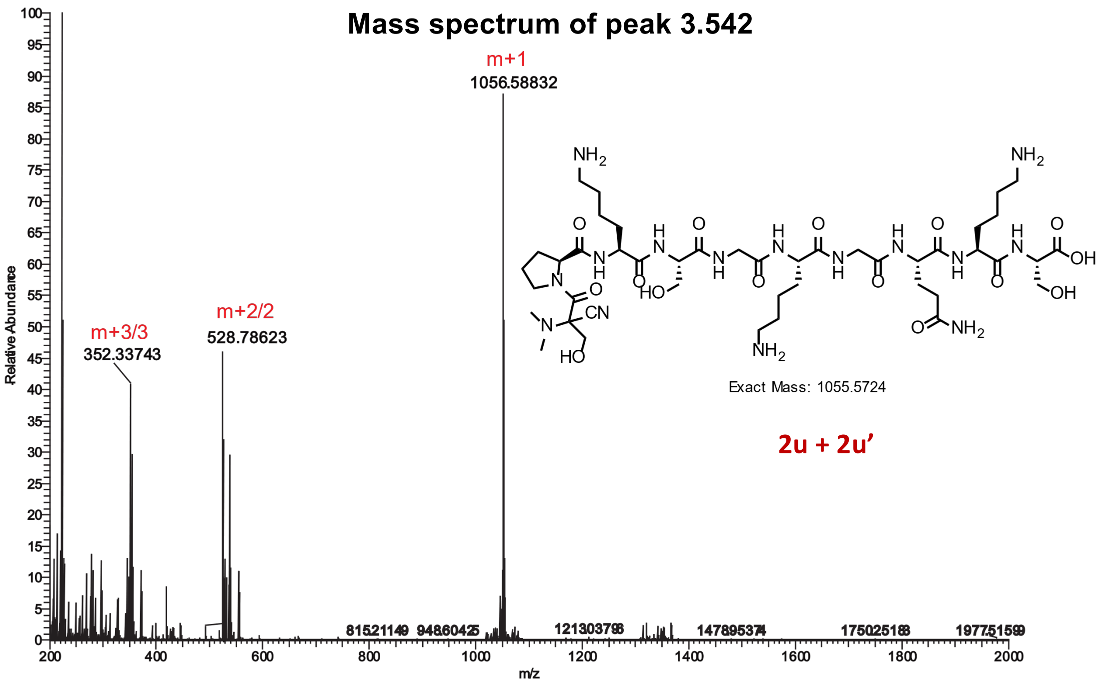

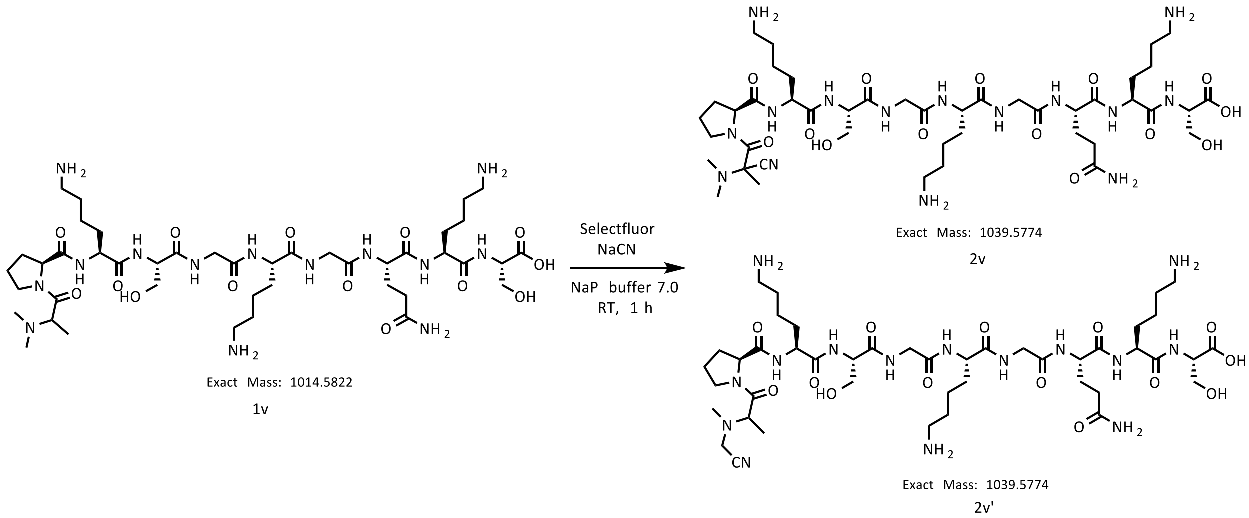

Modification of myosin light chain 1 peptide sequence 1v: To 1.0 mg of N,N-dimethyl histone myosin light chain-1 peptide **1v** dissolved in 300 µL of 10 mM sodium phosphate buffer (NaP, pH 7.0), was added sodium cyanide (3 equiv.) and selectfluor (2 equiv.). The reaction mixture was stirred for 1 h. Sample was taken from the reaction mixture, injected into LC-MS to monitor the generation of nitrile peptide products (**2v** and **2v’**). The reaction mixture was analyzed by HPLC using method A to determine the % conversion. The nitrile products were not separable by HPLC. The nitrile products were observed with conversion of >98 %.

N,N-dimethyl myosin light chain nitrile peptide products 2v and 2v’. LCMS: *m/z* 1040.05042 (calcd [M+H]+ = 1040.5847), *m/z* 347.06802 (calcd [M+3/3]+ = 347.5282), Purity: >95 % (HPLC analysis at 220 nm). Retention time in HPLC: 4.634

HPLC trace of reaction

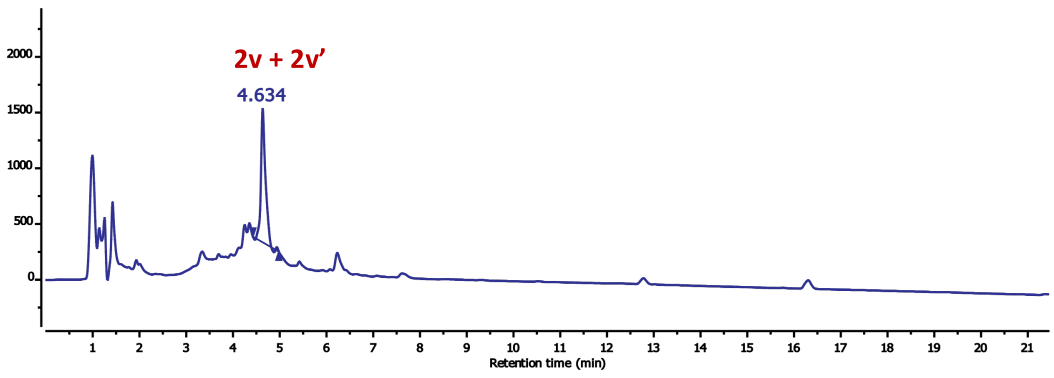

Mass spectrum of peak 4.634

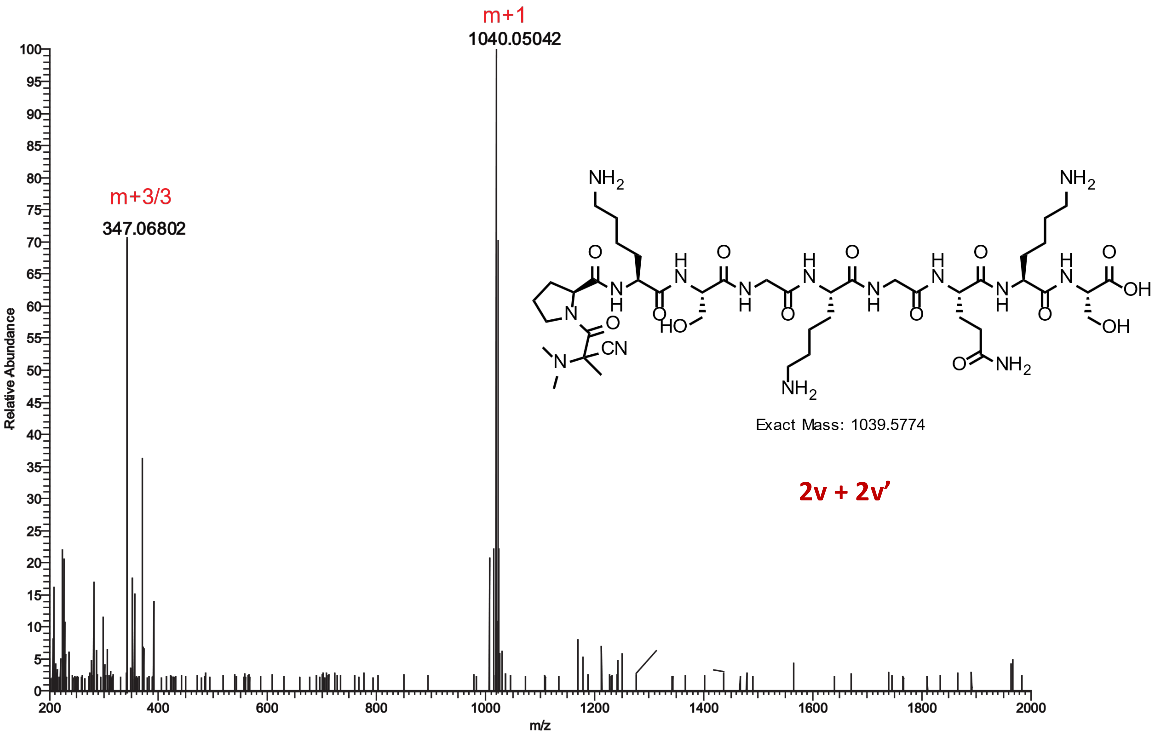

Modification of histone H2B peptide sequence 1w: To 1.0 mg of N,N-dimethyl histone H2B peptide **1w** dissolved in 300 μL of 10 mM sodium phosphate buffer (NaP, pH 7.0), was added sodium cyanide (3 equiv.) and selectfluor (2 equiv.). The reaction mixture was stirred for 1 h. Sample was taken from the reaction mixture, injected into LC-MS to monitor the generation of nitrile peptide products (**2w** and **2w'**). The reaction mixture was analyzed by HPLC using method A to determine the % conversion. The nitrile products were not separable by HPLC. The nitrile products were observed with conversion of >98 %.

N,N-dimethyl histone H2B nitrile peptide products 2w and 2w'. LCMS: *m/z* 1221.63452 (calcd [M+H]+ = 1221.6409), *m/z* 611.32074 (calcd [M+2/2]+ = 611.3168), Purity: >95 % (HPLC analysis at 220 nm). Retention time in HPLC: 6.14

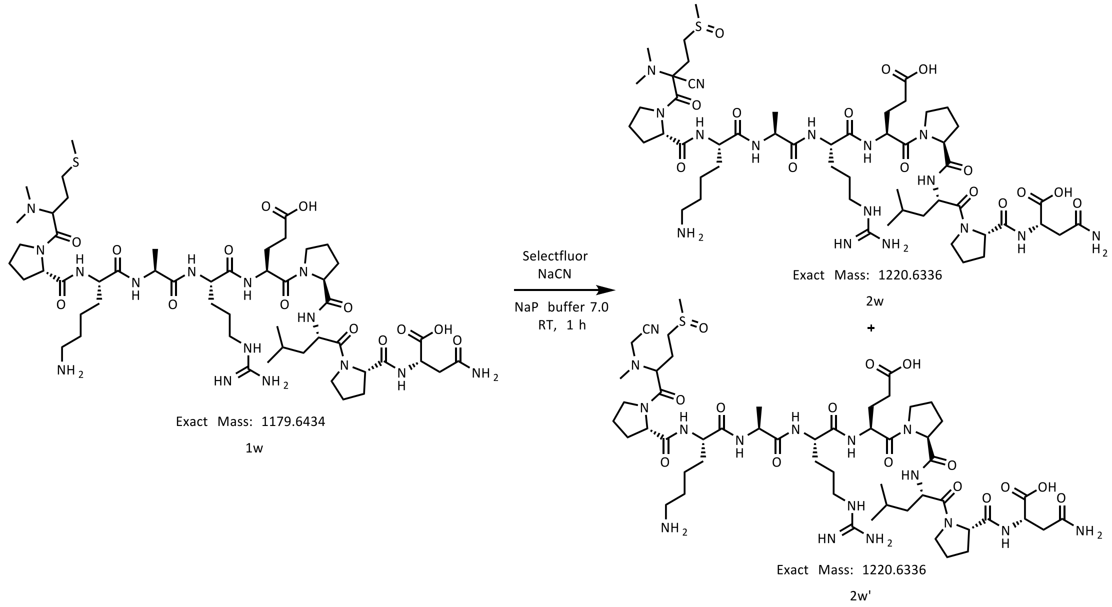

HPLC trace of reaction

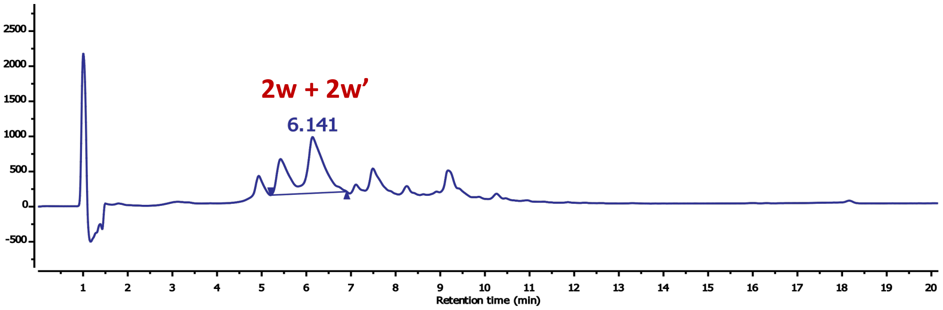

Mass spectrum of peak 6.141

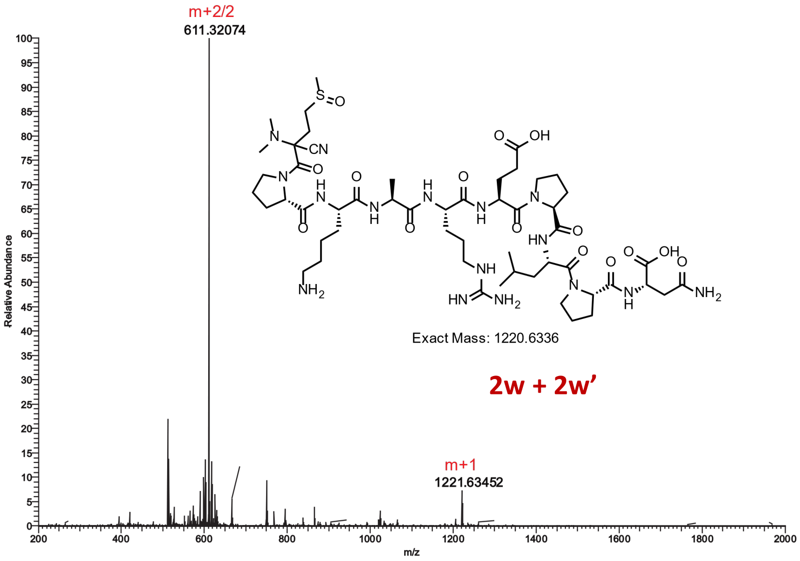

Modification of NTMT peptide inhibitor sequence without N-terminal dimethylation 1x: To 1.0 mg of N,N-dimethyl NTMT negative control peptide **1x** dissolved in 300 µL of 10 mM sodium phosphate buffer (NaP, pH 7.0), was added sodium cyanide (3 equiv.) and selectfluor (2 equiv.). The reaction mixture was stirred for 1 h. The reaction mixture was analyzed by HPLC using method A to determine the % conversion. No modification of the peptide was observed.

N,N-dimethyl NTMT negative control peptide 1x. LCMS: *m/z* 640.4148 (calcd [M+H]+ = 640.4253), *m/z* 320.7111 (calcd [M+2/2]+ = 320.7090), Purity: >95 % (HPLC analysis at 220 nm). Retention time in HPLC: 4.850

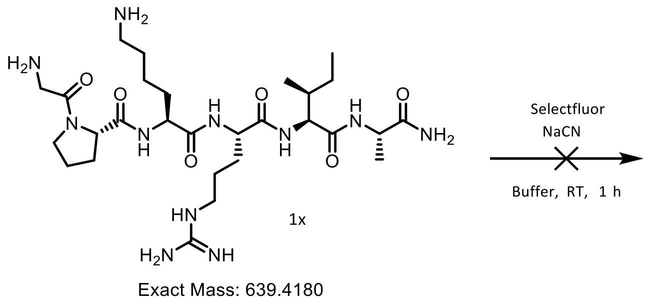

HPLC trace of reaction

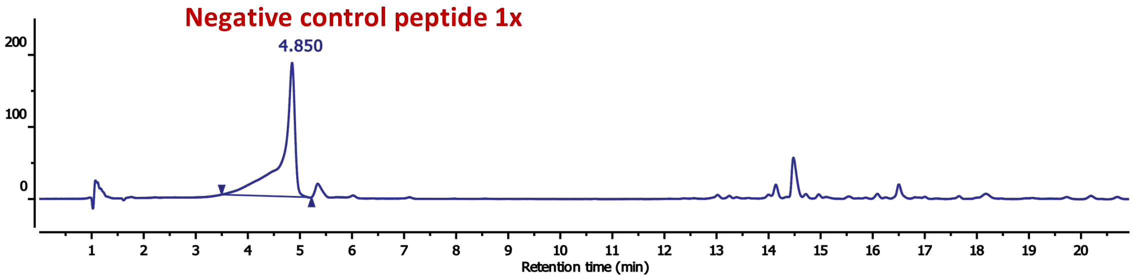

Mass spectrum of peak 4.850

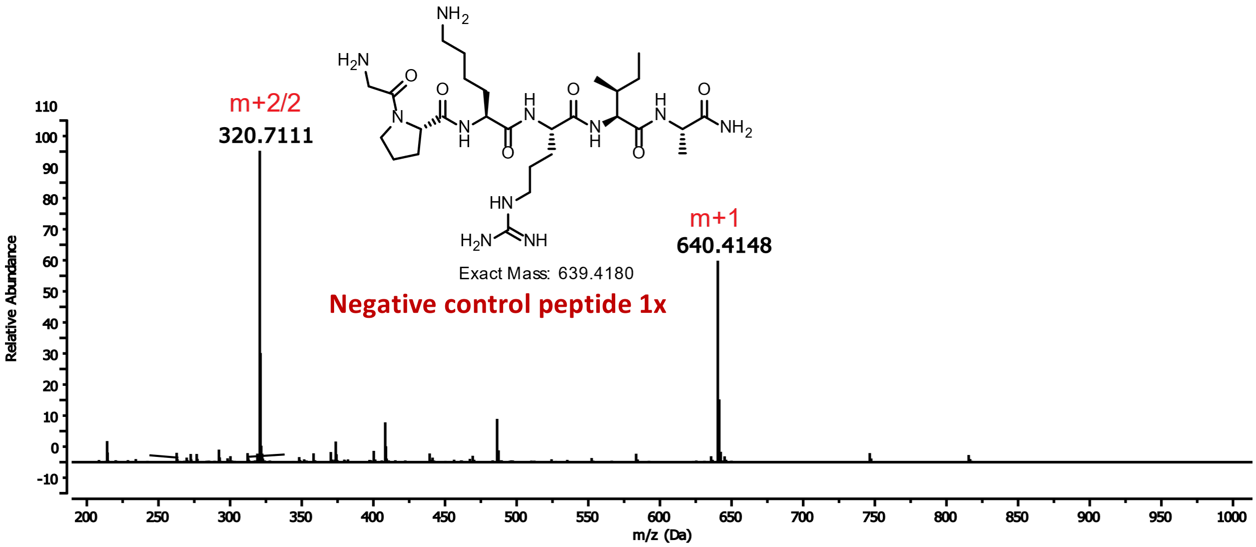

XXVIII. Supplementary Figure 21. Synthesis of cysteine-based affinity tags.

Synthesis of cysteine alkyne analog.

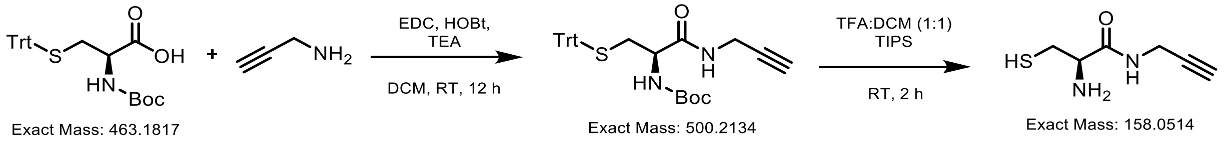

Synthesis of tert-butyl (R)-(1-oxo-1-(prop-2-yn-1-ylamino)-3-(tritylthio)propan-2-yl)carbamate: To a solution of Boc-(Trt)Cys-OH (500 mg, 1.08 mmol) and propargylamine (1.08 mmol) in CH_2Cl_2 (10 mL) was added EDC (1.08 mmol) HOBt (1.08 mmol) and triethylamine (1.08 mmol). Reaction was stirred for 12 h at room temperature and quenched with saturated aqueous $NaHCO_3$. Reaction was extracted with CH_2Cl_2 (3 x 20 mL) and organic fractions evaporated to obtain a white residue. The crude product was purified by column chromatography to give 76 % yield of the title compound.

tert-butyl (R)-(1-oxo-1-(prop-2-yn-1-ylamino)-3-(tritylthio)propan-2-yl)carbamate: ^1H **NMR** (400 MHz, CD_3OD) δ 7.44 – 7.17 (m, 15H), 3.97 (t, *J* = 6.6 Hz, 1H), 3.91 (dd, *J* = 4.5, 2.6 Hz, 2H), 2.56 – 2.40 (m, 2H), 1.43 (s, 9H).

^1H NMR of tert-butyl (R)-(1-oxo-1-(prop-2-yn-1-ylamino)-3-(tritylthio)propan-2-yl)carbamate

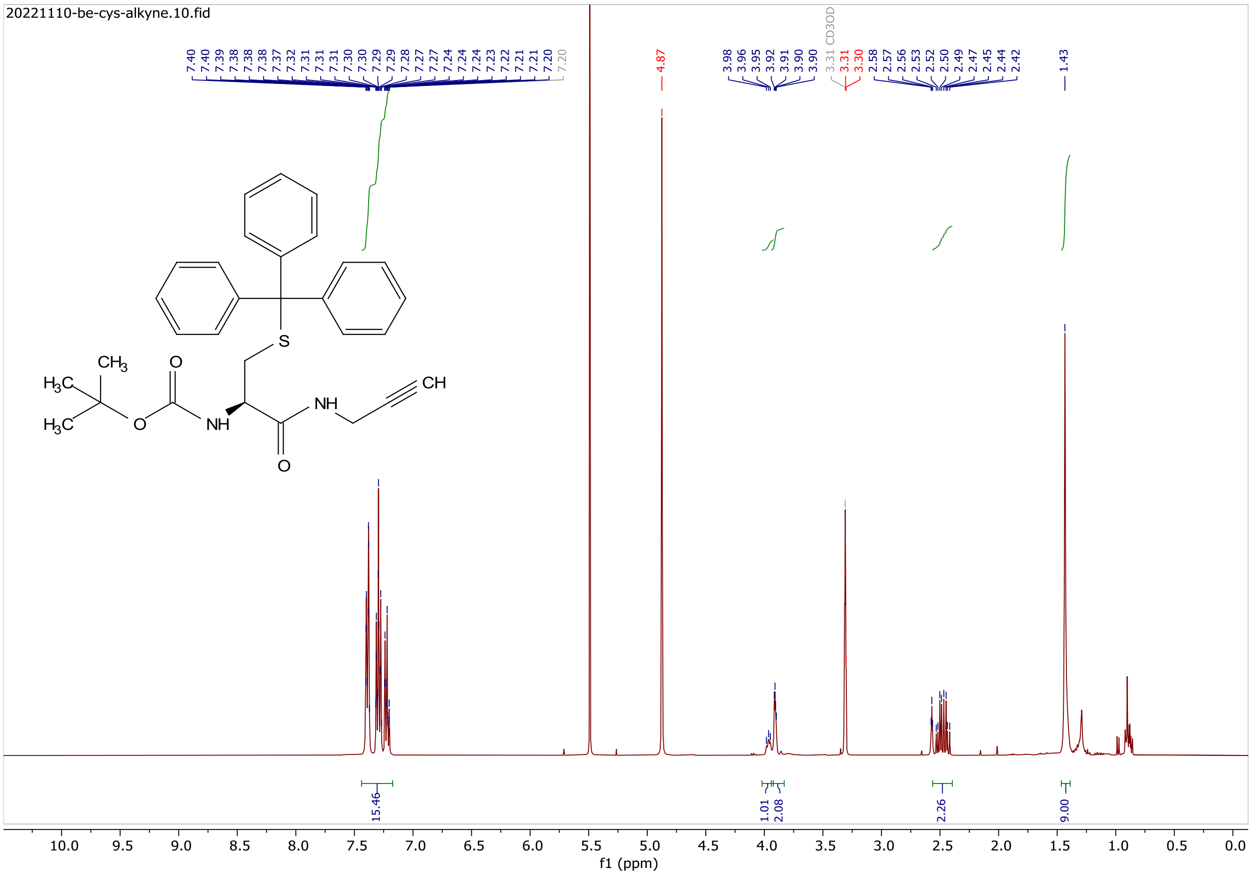

Synthesis of 2-amino-3-mercapto-N-(prop-2-yn-1-yl)propanamide: To 500 mg of tert-butyl (R)-(1-oxo-1-(prop-2-yn-1-ylamino)-3-(tritylthio)propan-2-yl)carbamate, was added 10 mL of CH_2Cl_2, 10 mL of trifluoroacetic acid, and 200 µL of triisopropylsilane (TIPS). The reaction mixture was stirred at room temperature for 2 h. The reaction was concentrated in vacuo and washed with hexane (3x) to remove trityl-based impurities. Concentration of the residue gave the title compound.

2-amino-3-mercapto-N-(prop-2-yn-1-yl)propanamide: ^{1}H NMR (400 MHz, CD_3OD) δ 4.09 (d, J = 6.1 Hz, 1H), 4.04 (d, J = 2.6 Hz, 2H), 3.10 – 2.97 (m, 2H), 2.67 (t, J = 2.6 Hz, 1H).

^{1}H NMR of 2-amino-3-mercapto-N-(prop-2-yn-1-yl)propanamide

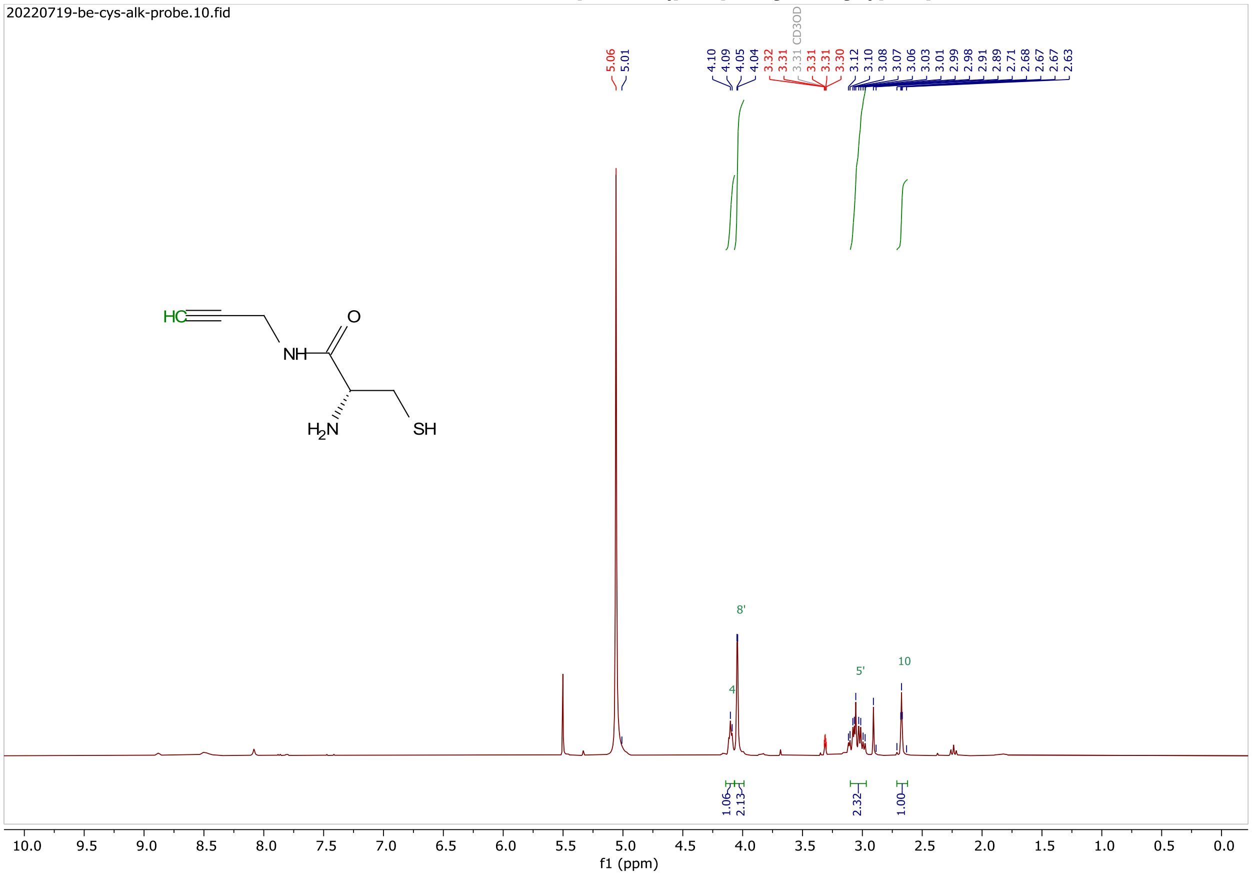

Synthesis of cysteine azide analog.

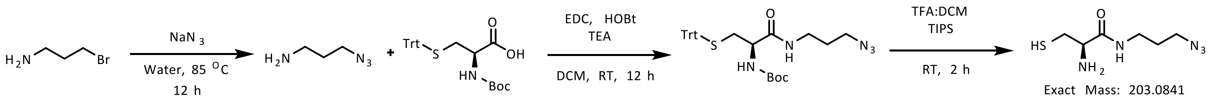

Synthesis of azido propylamine: To (1 g, 4.6 mmol) 2-bromopropylamine hydrobromide in 7 mL of water, was added (1 g, 15 mmol) of sodium azide. The reaction was stirred at 85 ^{O}C for 12 h. After 12 h, the reaction mixture was cooled and 200 mg NaOH was added to the reaction mixture followed by addition of diethyl ether and water. The mixture was stirred for 10 min, and organic layer was collected, dried in vacuo, and purified by column chromatography on silica gel with 8 % MeOH-CH_2Cl_2 mixture as the eluent.

Azido propylamine: **^{1}H NMR** (400 MHz, CD_3OD) δ 3.37 (t, *J* = 6.8 Hz, 2H), 2.71 (t, *J* = 7.0 Hz, 2H), 1.73 (p, *J* = 6.8 Hz, 2H).

^{1}H NMR of azido propylamine

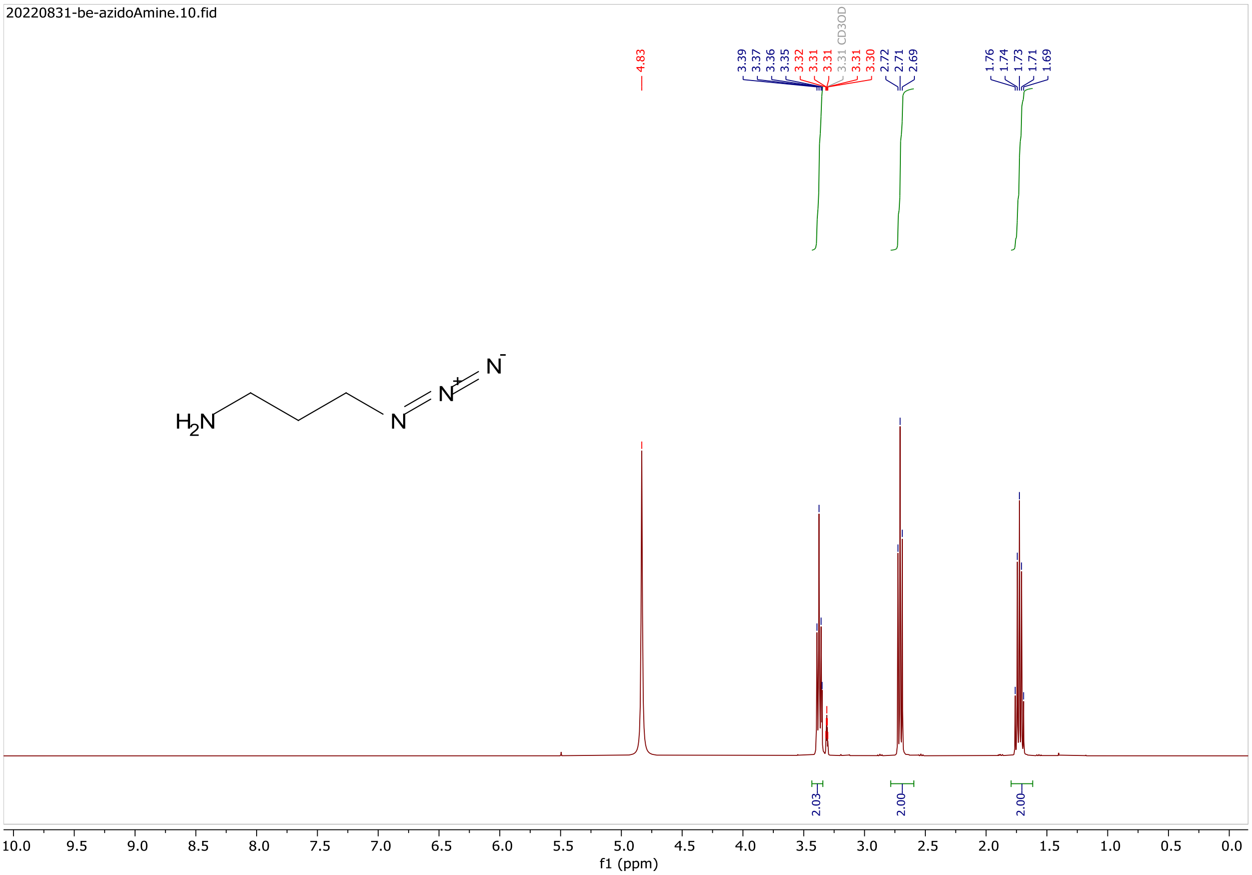

Synthesis of tert-butyl (R)-(1-((3-azidopropyl)amino)-1-oxo-3-(tritylthio)propan-2-yl)carbamate: To a solution of Boc-(Trt)Cys-OH (500 mg, 1.08 mmol) and azidopropylamine (1.08 mmol) in CH_2Cl_2 (10 mL) was added EDC (1.08 mmol) HOBt (1.08 mmol) and triethylamine (1.08 mmol). Reaction was stirred for 12 h at room temperature and quenched with saturated aqueous $NaHCO_3$. Reaction was extracted with CH_2Cl_2 (3 x 20 mL) and organic fractions evaporated to obtain a white residue. The cured product was purified by column chromatography to give 85 % yield of the title compound.

tert-butyl (R)-(1-((3-azidopropyl)amino)-1-oxo-3-(tritylthio)propan-2-yl)carbamate: ^{1}H NMR (400 MHz, CD_3OD) δ 7.47 – 7.15 (m, 15H), 3.94 (t, *J* = 6.8 Hz, 1H), 3.34 (d, *J* = 7.9 Hz, 2H), 3.20 (ddt, *J* = 20.2, 13.5, 6.6 Hz, 2H), 2.48 (q, *J* = 7.5, 6.8 Hz, 2H), 1.71 (h, *J* = 6.4 Hz, 2H), 1.43 (s, 9H).

^{1}H NMR of tert-butyl (R)-(1-((3-azidopropyl)amino)-1-oxo-3-(tritylthio)propan-2-yl)carbamate

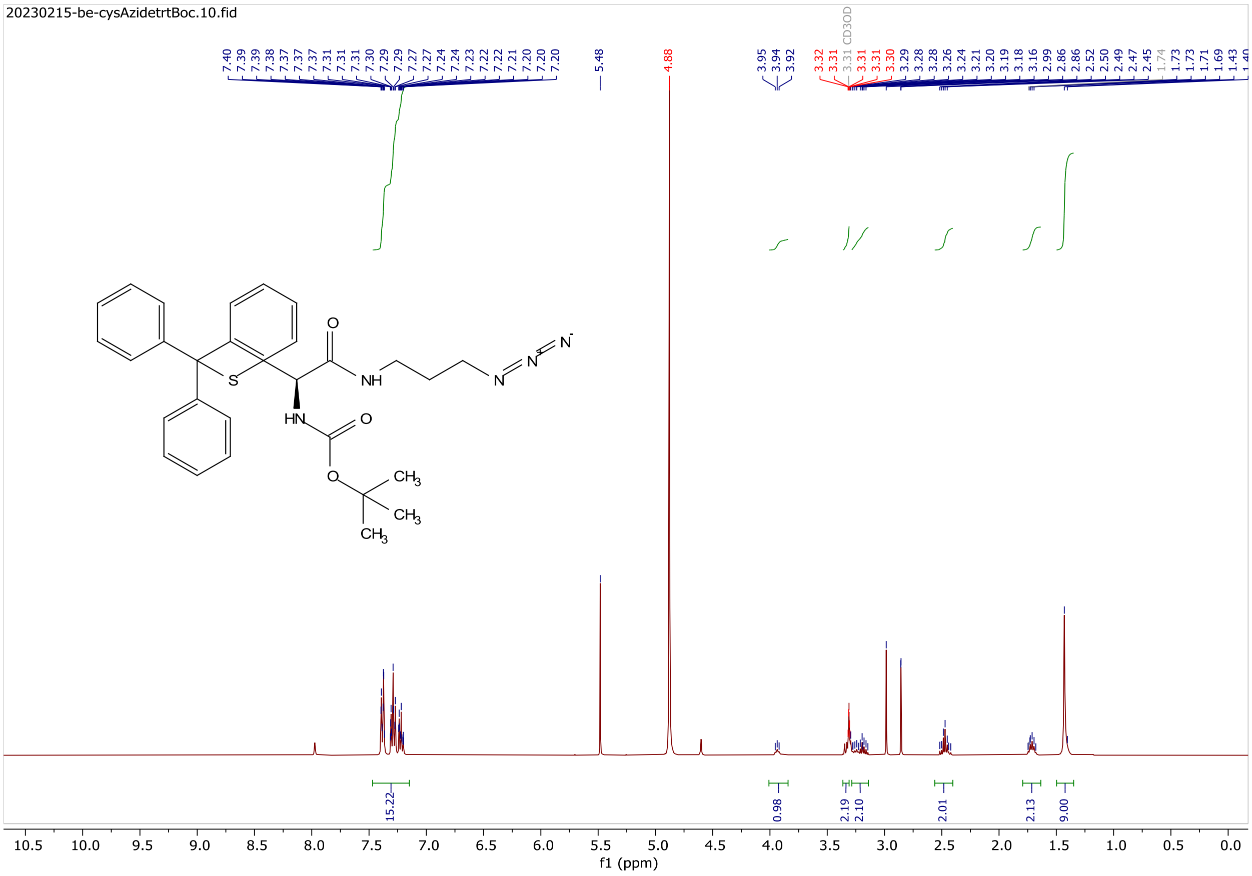

Synthesis of -2-amino-N-(3-azidopropyl)-3-mercaptopropanamide: To 500 mg of tert-butyl (R)-(1-((3-azidopropyl)amino)-1-oxo-3-(tritylthio)propan-2-yl)carbamate, was added 10 mL of CH_2Cl_2, 10 mL of trifluoroacetic acid, and 200 µL of triisopropylsilane (TIPS). The reaction mixture was stirred at room temperature for 2 h. The reaction was concentrated in vacuo and washed with hexane (3x) to remove trityl-based impurities. Concentration of the residue gave the title compound.

2-amino-N-(3-azidopropyl)-3-mercaptopropanamide: ^{1}H NMR (400 MHz, CD_3OD) δ 4.00 (ddd, J = 22.0, 6.7, 5.4 Hz, 1H), 3.39 (td, J = 6.2, 5.6, 3.1 Hz, 2H), 3.36 – 3.31 (m, 2H), 3.11 – 3.00 (m, 1H), 2.95 (dd, J = 14.6, 6.8 Hz, 1H), 1.80 (p, J = 6.4 Hz, 2H).

^{1}H NMR of 2-amino-N-(3-azidopropyl)-3-mercaptopropanamide

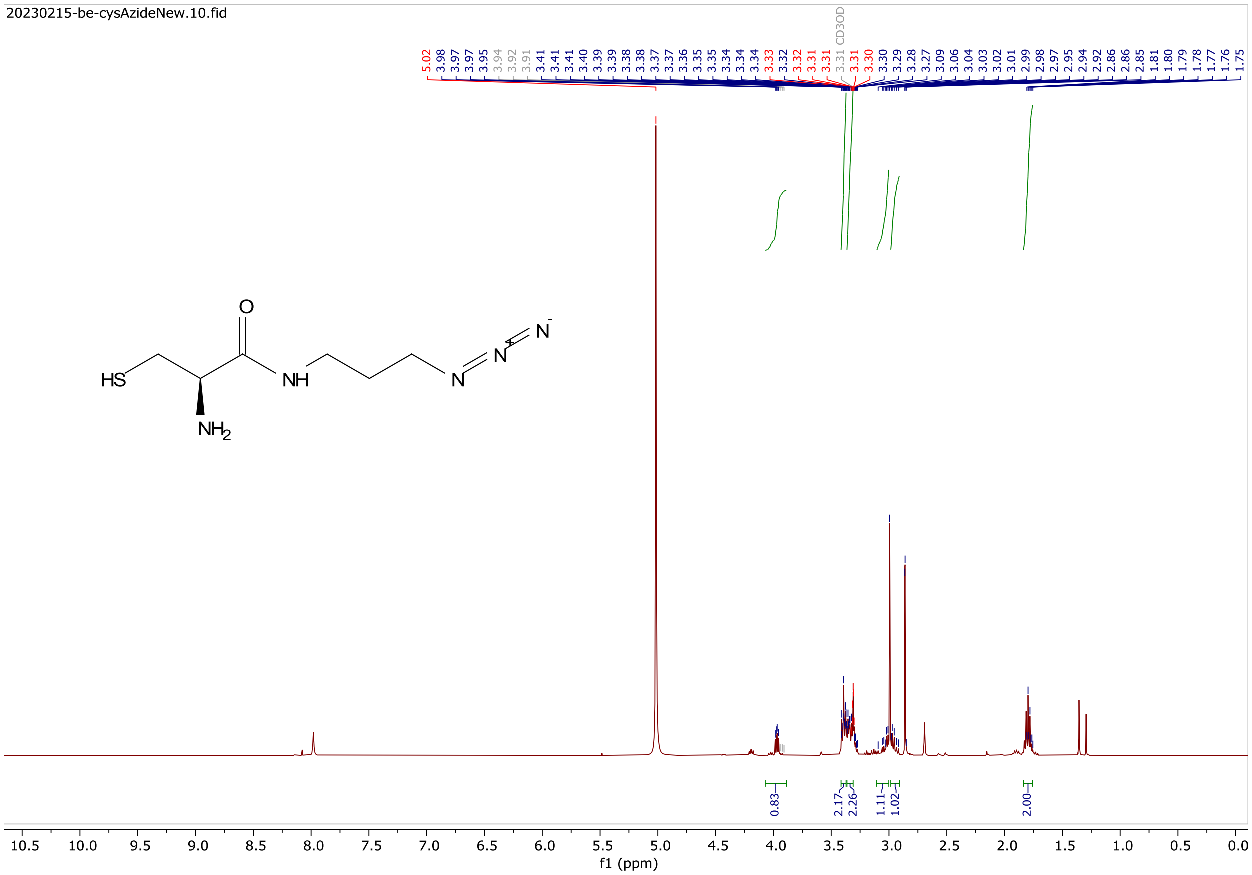

Synthesis of cysteine biotin analog.

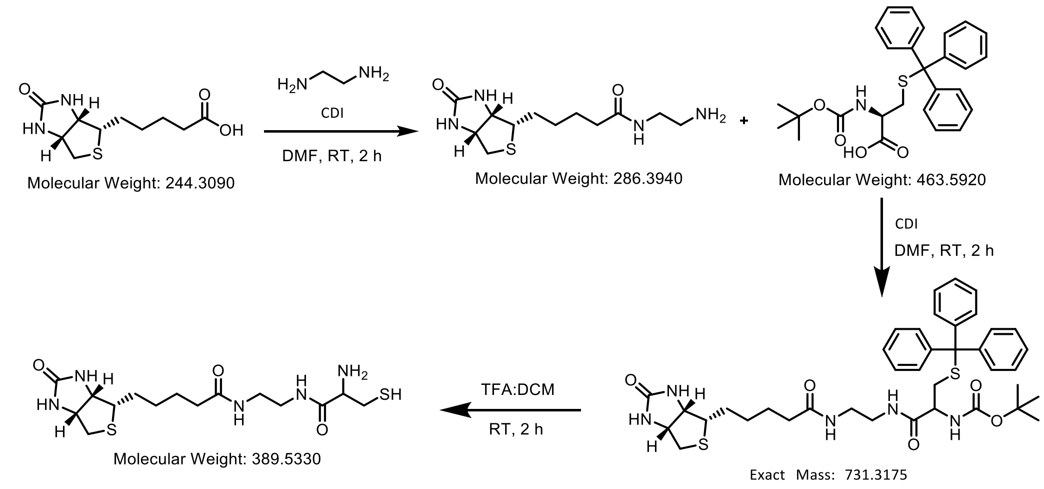

Synthesis of N-(2-aminoethyl)-5-((3aS,4S,6aR)-2-oxohexahydro-1H-thieno[3,4-d]imidazol-4-yl)pentanamide: To 1 g of biotin dissolved in 10 mL of DMF was added carbonyl diimidazole (864 mg, 5.3 mmol). The reaction was stirred for 30 minutes. After 30 minutes, ethylenediamine (1.4 mL, 22.6 mmol) was added in single portion to the reaction mixture. The reaction was

stirred for a further 2 h and DMF removed in vacuo. Residue was dissolved in 50 mL of CH_2Cl_2 and washed with water (3 x 50 mL) and saturated brine (50 mL). The organic fraction was dried with anhydrous magnesium sulfate. Solvent was reduced to 10 mL in vacuo and products collected by filtration and dried in vacuo.

N-(2-aminoethyl)-5-((3aS,4S,6aR)-2-oxohexahydro-1H-thieno[3,4-d]imidazol-4-yl)pentanamide: ^{1}**H NMR** (400 MHz, DMSO-*d*$_6$) δ 7.81 (t, *J* = 22.6 Hz, 1H), 6.43 (s, 1H), 6.36 (s, 1H), 4.30 (dd, *J* = 7.7, 5.0 Hz, 1H), 4.12 (t, *J* = 6.2 Hz, 1H), 3.13 – 3.05 (m, 2H), 3.02 (t, *J* = 6.1 Hz, 2H), 2.91 – 2.71 (m, 1H), 2.58 (d, *J* = 5.4 Hz, 2H), 2.57 – 2.51 (m, 2H), 2.05 (td, *J* = 7.4, 4.6 Hz, 2H), 1.76 – 1.50 (m, 2H), 1.45 (dt, *J* = 22.2, 8.1 Hz, 2H), 1.37 – 1.17 (m, 2H).

^{1}H NMR of N-(2-aminoethyl)-5-((3aS,4S,6aR)-2-oxohexahydro-1H-thieno[3,4-d]imidazol-4-yl)pentanamide

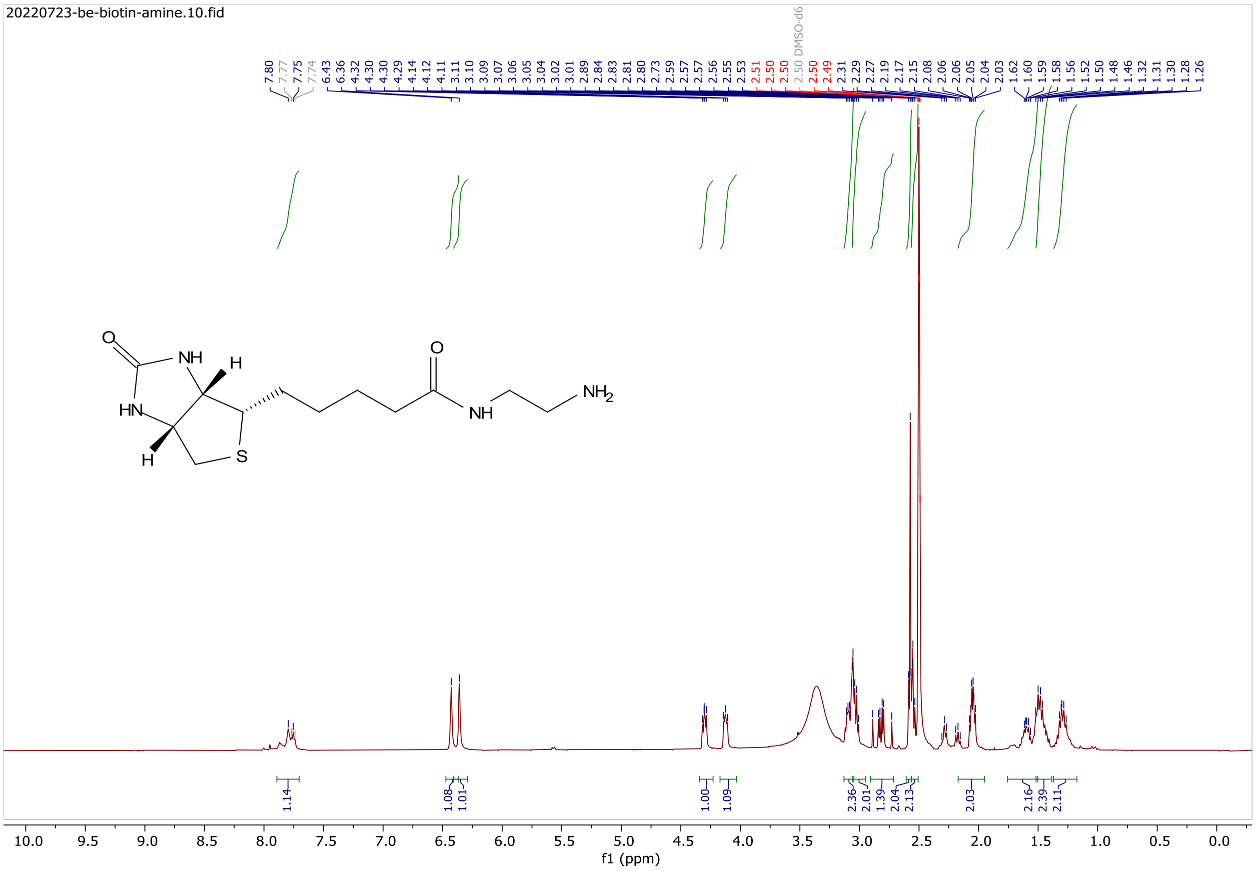

Synthesis of tert-butyl (1-oxo-1-((2-(5-((3aS,4S,6aR)-2-oxohexahydro-1H-thieno[3,4-d]imidazol-4-yl)pentanamido)ethyl)amino)-3-(tritylthio)propan-2-yl)carbamate: To 1 g of N-Boc-S-trityl-L-cysteine dissolved in 10 mL of DMF was added carbonyl diimidazole (420 mg, 2.6 mmol). The reaction was stirred for 30 minutes. After 30 minutes, N-(2-aminoethyl)-5-((3aS,4S,6aR)-2-oxohexahydro-1H-thieno[3,4-d]imidazol-4-yl)pentanamide (743 g, 2.6 mmol) was added in single portion to the reaction mixture. The reaction was stirred for a further 2 h and DMF removed in vacuo. Residue was dissolved in 50 mL of CH_2Cl_2 and washed with water (3 x 50 mL) and saturated brine (50 mL). The organic fraction was dried with anhydrous magnesium

sulfate. Solvent was reduced to 10 mL in vacuo and products collected by filtration and dried in vacuo.

tert-butyl (1-oxo-1-((2-(5-((3aS,4S,6aR)-2-oxohexahydro-1H-thieno[3,4-d]imidazol-4-yl)pentanamido)ethyl)amino)-3-(tritylthio)propan-2-yl)carbamate: ^{1}H NMR (400 MHz, DMSO-d_6) δ 7.34 – 7.27 (m, 15H), 7.06 – 6.79 (m, 1H), 6.53 – 6.30 (m, 1H), 4.41 – 3.99 (m, 1H), 3.90 (d, *J* = 7.7 Hz, 1H), 3.06 (d, *J* = 7.0 Hz, 2H), 3.04 (s, 2H), 2.56 (dd, *J* = 12.6, 5.4 Hz, 1H), 2.39 – 2.30 (m, 2H), 2.26 (t, *J* = 9.2 Hz, 1H), 2.08 (s, 1H), 1.54 (d, *J* = 49.8 Hz, 2H), 1.37 (d, *J* = 2.6 Hz, 9H), 1.32 (d, *J* = 20.5 Hz, 2H), 1.21 (s, 2H).

^{1}H NMR of tert-butyl (1-oxo-1-((2-(5-((3aS,4S,6aR)-2-oxohexahydro-1H-thieno[3,4-d]imidazol-4-yl)pentanamido)ethyl)amino)-3-(tritylthio)propan-2-yl)carbamate

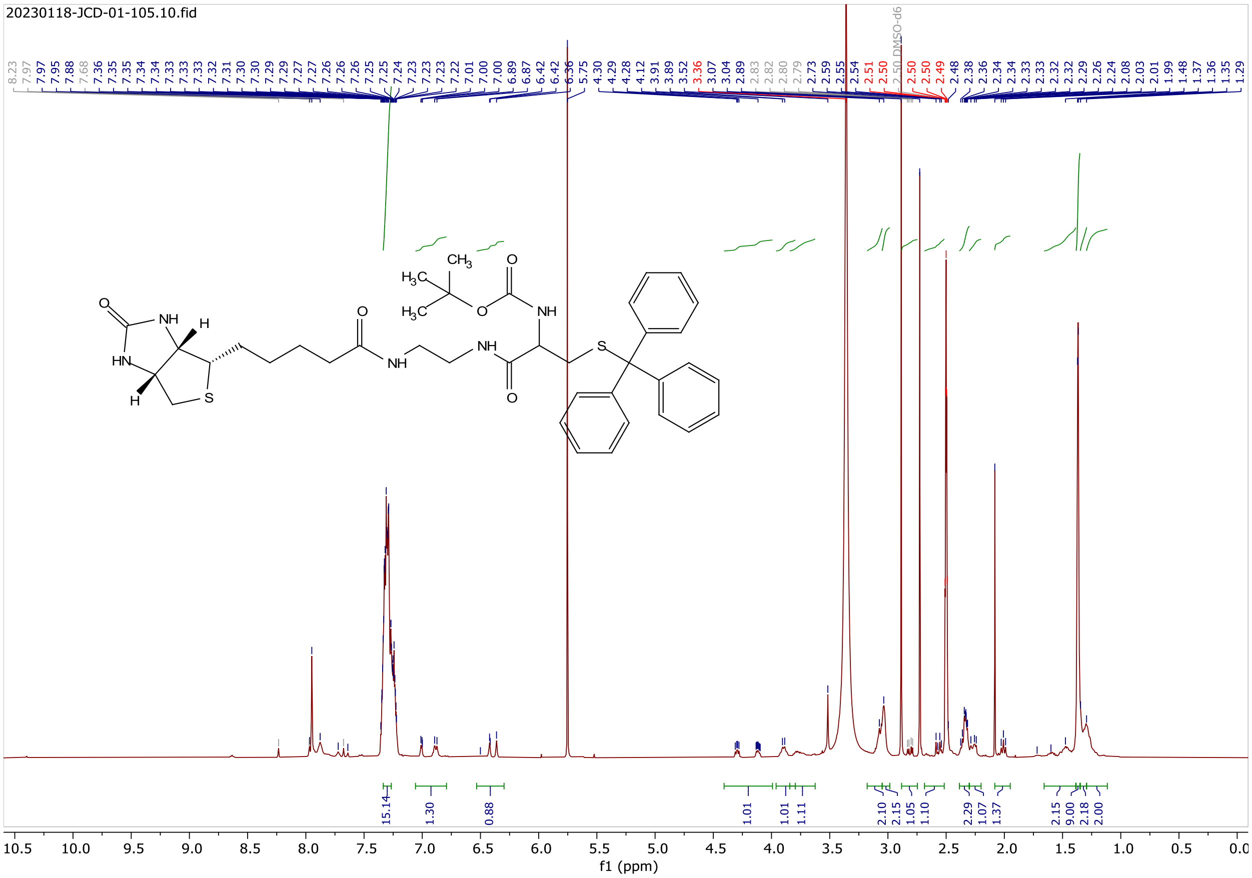

Synthesis of N-(2-(2-amino-3-mercaptopropanamido)ethyl)-5-((3aS,4S,6aR)-2-oxohexahydro-1H-thieno[3,4-d]imidazol-4-yl)pentanamide: To 500 mg of tert-butyl (1-oxo-1-((2-(5-((3aS,4S,6aR)-2-oxohexahydro-1H-thieno[3,4-d]imidazol-4-yl)pentanamido)ethyl)amino)-3-(tritylthio)propan-2-yl)carbamate, was added 10 mL of CH_2Cl_2, 10 mL of trifluoroacetic acid, and 200 µL of triisopropylsilane (TIPS). The reaction mixture was stirred at room temperature for 2 h. The reaction was concentrated in vacuo and washed with hexane (3x) to remove trityl-based impurities. Concentration of the residue gave the title compound.

N-(2-(2-amino-3-mercaptopropanamido)ethyl)-5-((3aS,4S,6aR)-2-oxohexahydro-1H-thieno[3,4-d]imidazol-4-yl)pentanamide: ^{1}H NMR (400 MHz, CD_3OD) δ 8.94 (s, 1H), 8.05 (s,

1H), 4.69 (dd, J = 8.1, 4.7 Hz, 1H), 4.56 – 4.45 (m, 1H), 4.31 – 4.27 (m, 1H), 4.15 – 3.95 (m, 2H), 3.63 (dd, J = 6.6, 4.7 Hz, 1H), 3.41 (dt, J = 8.7, 2.9 Hz, 2H), 3.38 – 3.36 (m, 2H), 3.15 – 3.12 (m, 2H), 3.10 (s, 2H), 2.38 – 2.20 (m, 2H), 1.73 (dtd, J = 30.4, 7.8, 7.3, 5.0 Hz, 2H), 1.65 – 1.46 (m, 2H), 1.43 – 1.31 (m, 2H).

^{1}H NMR of N-(2-(2-amino-3-mercaptopropanamido)ethyl)-5-((3aS,4S,6aR)-2-oxohexahydro-1H-thieno[3,4-d]imidazol-4-yl)pentanamide

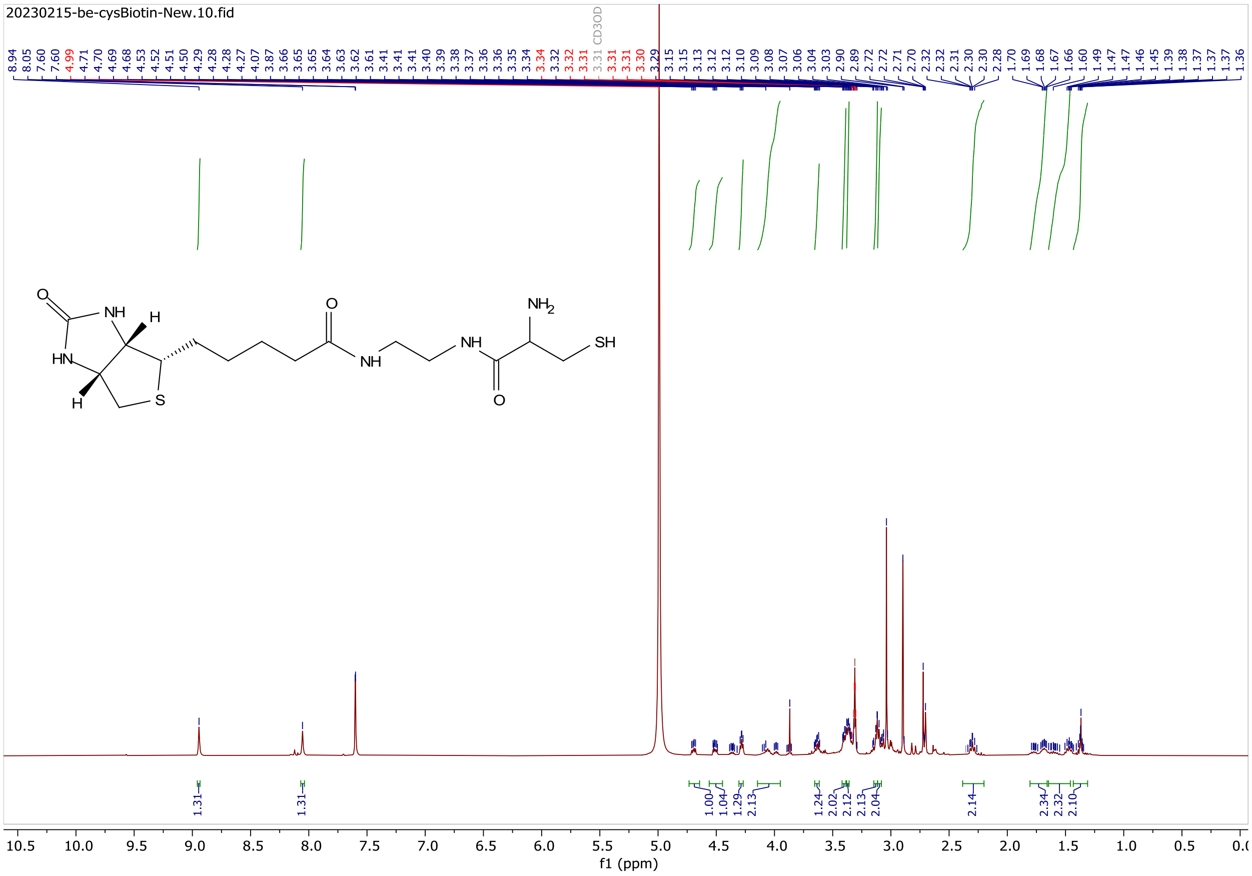

XXIX. Supplementary Figure 22. Diversification of N-terminal dimethyl containing peptides with various affinity tags.

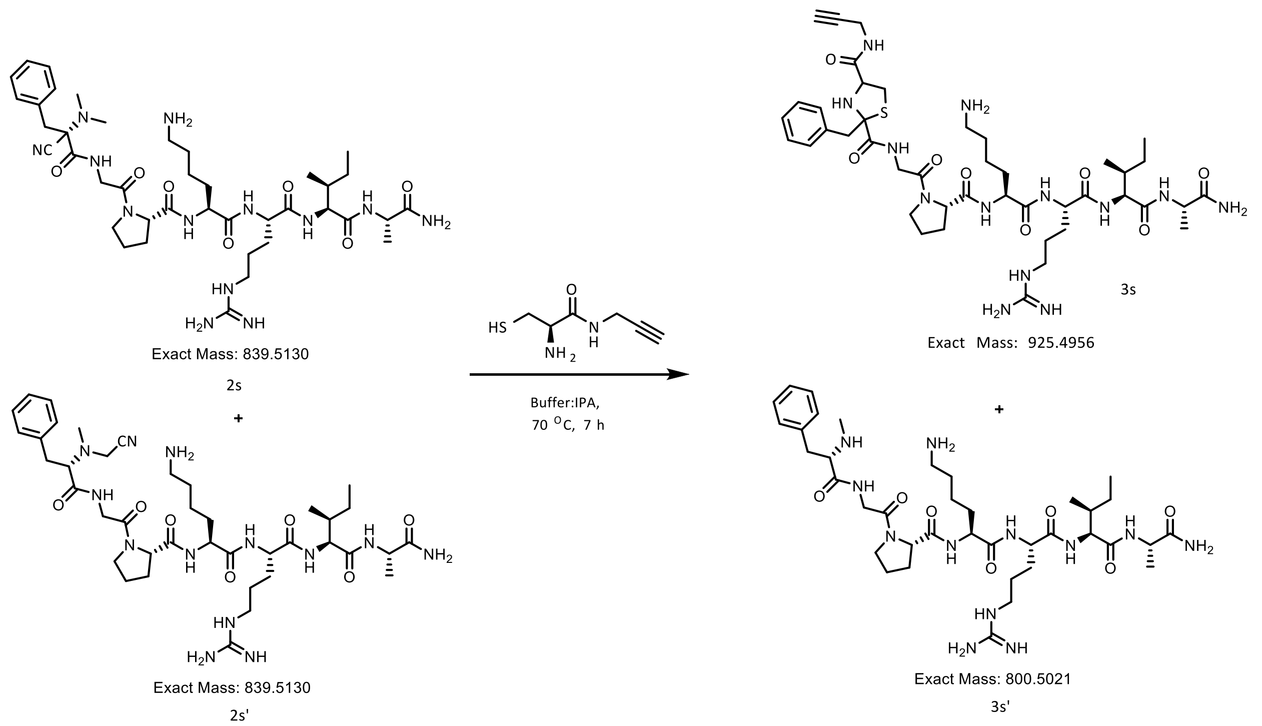

To 1.0 mg of N,N-dimethyl nitrile NTMT peptide **2s + 2s'** dissolved in 600 µL of 10 mM sodium phosphate buffer (NaP, pH 7.0) and isopropyl alcohol (1:1), was added 3 equiv. of cysteine-alkyne analog. The reaction mixture was stirred at 70 ^{O}C for 7 h. Sample was taken from the reaction mixture, injected into LC-MS to monitor the generation of thiazolidine and demethylated peptide products **3s** and **3s'**. The reaction mixture was analyzed by HPLC using method A to determine the % conversion.

N,N-dimethyl-NTMT alkyne thiazolidine peptide products 3s. LCMS: *m/z* 926.4810 (calcd [M+H]+ = 926.5029), *m/z* 463.7447 (calcd [M+2/2]+ = 463.7514), Purity: >95 % (HPLC analysis at 220 nm). Retention time in HPLC: 9.983

Demethylated N-methyl-FRV peptide products 3s'. LCMS: *m/z* 801.50933 (calcd [M+H]+ = 801.5093), *m/z* 401.25465 (calcd [M+2/2]+ = 401.2565), Purity: >95 % (HPLC analysis at 220 nm). Retention time in HPLC: 3.592

HPLC trace of reaction

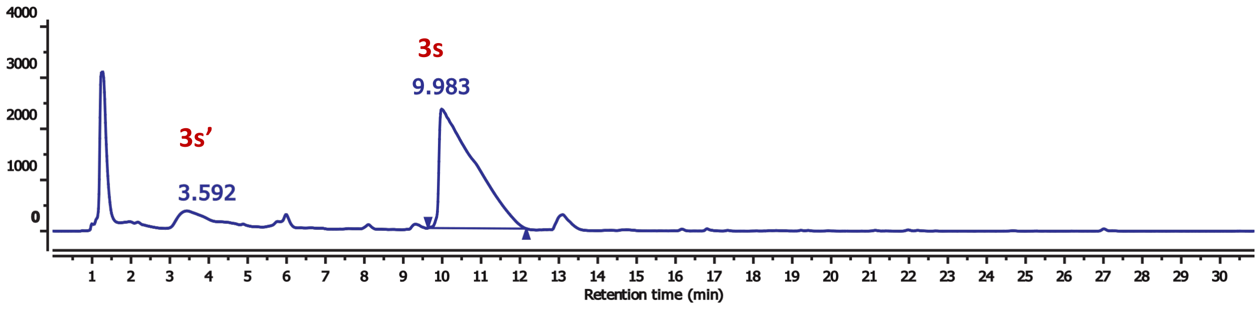

Mass spectra of peak 9.983

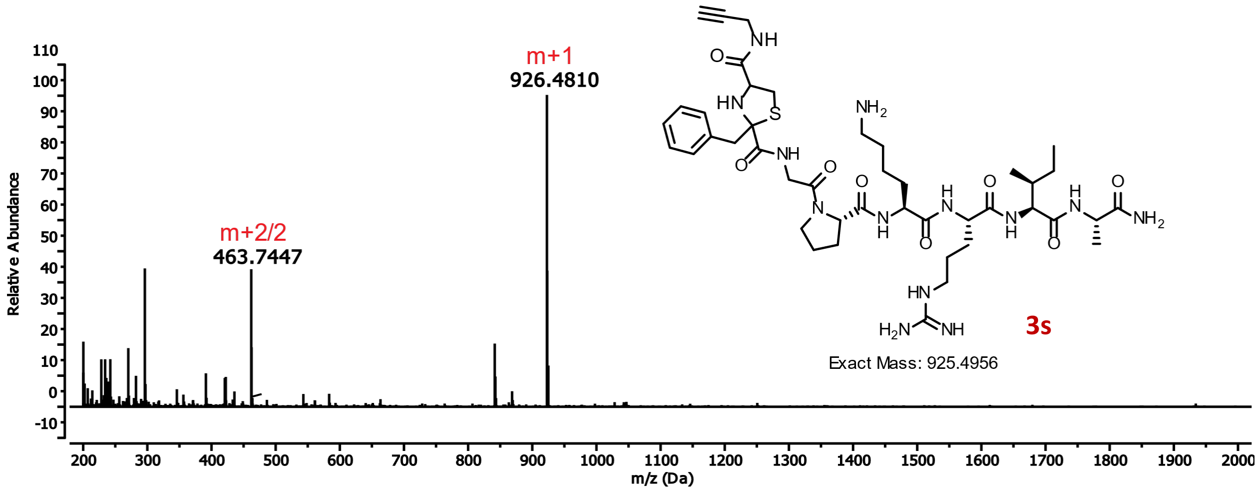

Mass spectra of peak 3.592

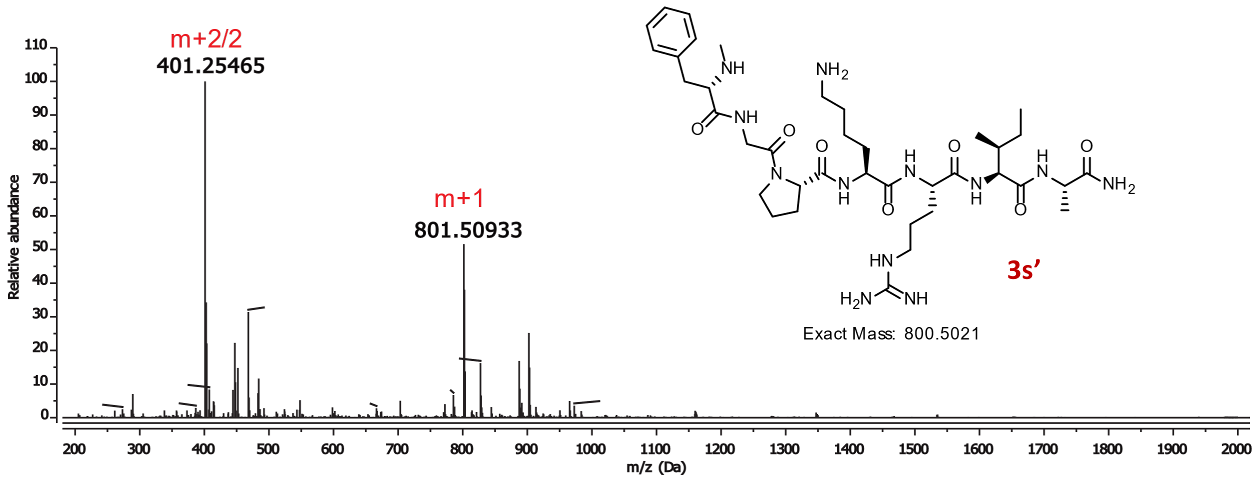

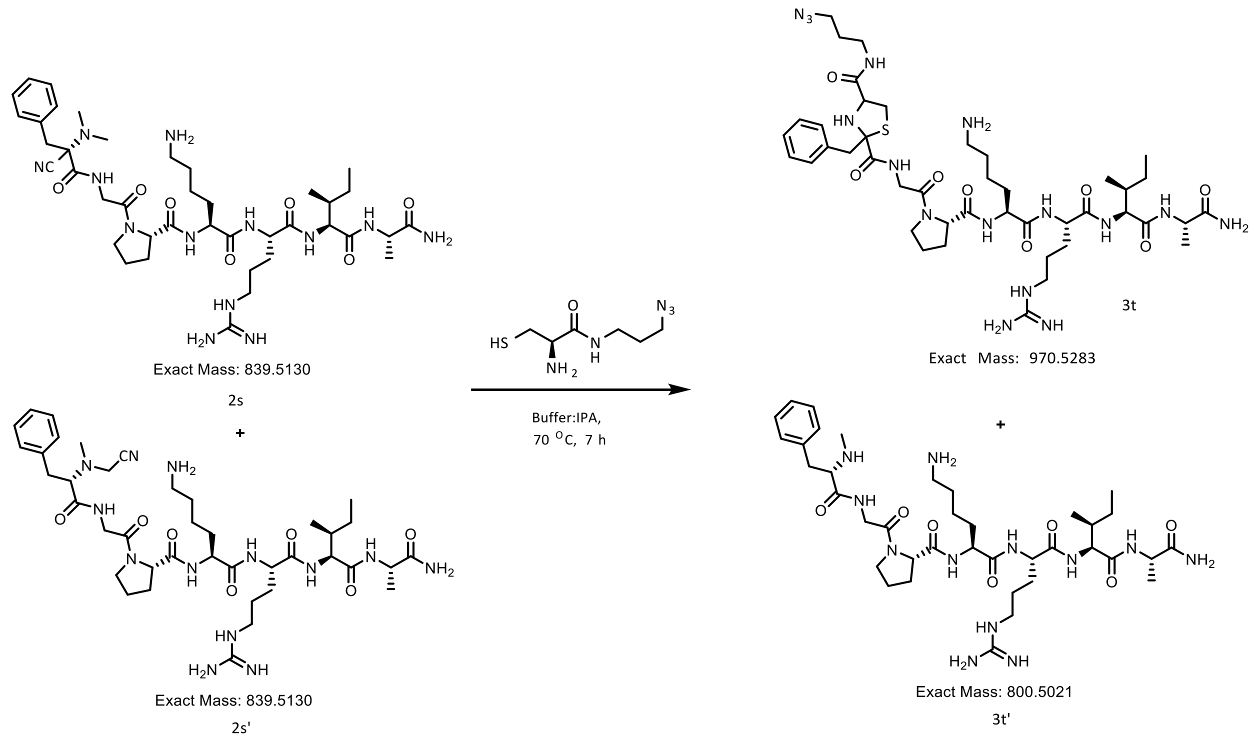

To 1.0 mg of N,N-dimethyl nitrile NTMT peptide **2s + 2s'** dissolved in 600 µL of 10 mM sodium phosphate buffer (NaP, pH 7.0) and isopropyl alcohol (1:1), was added 3 equiv. of cysteine-azide analog. The reaction mixture was stirred at 70 ^{O}C for 7 h. Sample was taken from the reaction mixture, injected into LC-MS to monitor the generation of thiazolidine and demethylated peptide products **3t** and **3t'**. The reaction mixture was analyzed by HPLC using method A to determine the % conversion. We did not observe any **3t'** product.

N,N-dimethyl-NTMT azido thiazolidine peptide products 3t. LCMS: *m/z* 971.53140 (calcd [M+H]+ = 971.5356), *m/z* 486.50450 (calcd [M+2/2]+ = 486.2678), Purity: >95 % (HPLC analysis at 220 nm). Retention time in HPLC: 6.491

HPLC trace of reaction

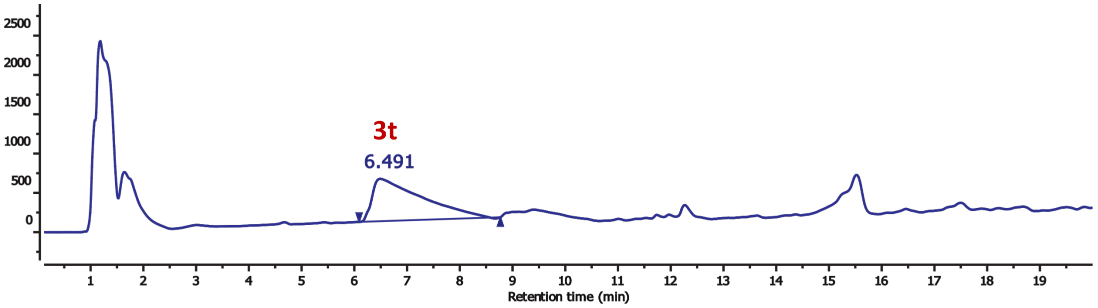

Mass spectra of azido thiazolidine peptide products (peak 6.491)

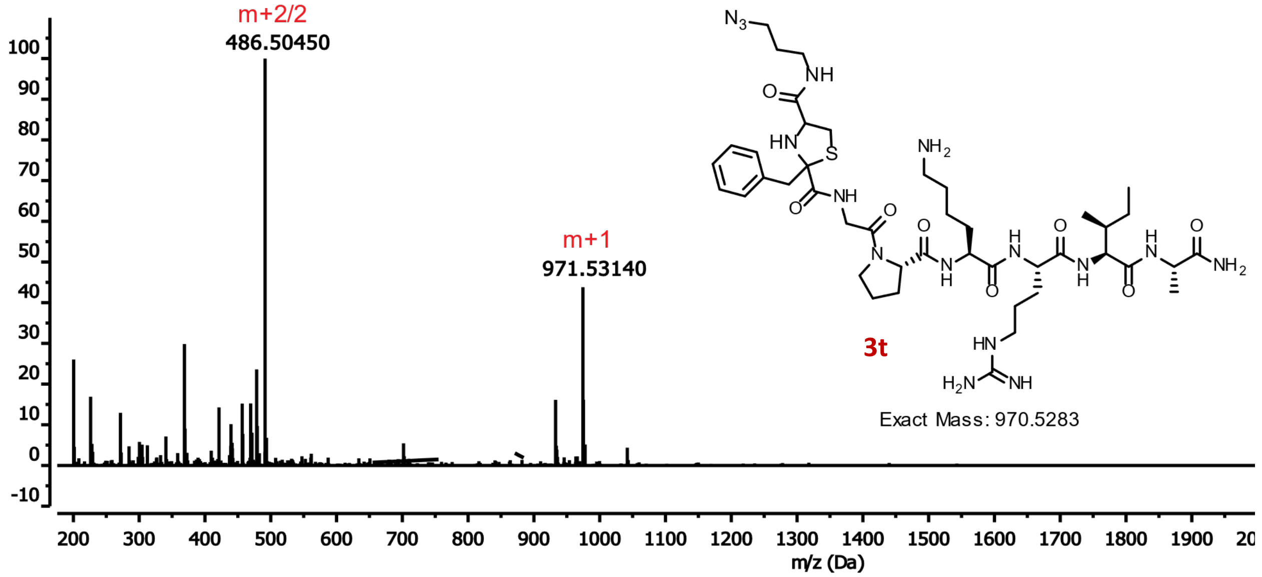

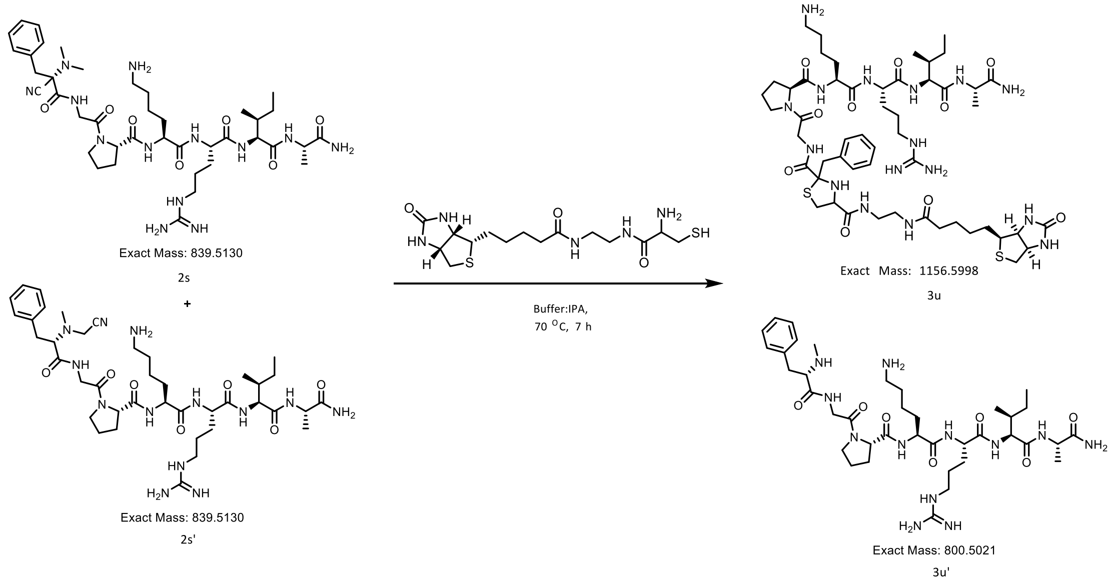

To 1.0 mg of N,N-dimethyl nitrile NTMT peptide **2s + 2s'** dissolved in 600 µL of 10 mM sodium phosphate buffer (NaP, pH 7.0) and isopropyl alcohol (1:1), was added 3 equiv. of cysteine-biotin analog. The reaction mixture was stirred at 70 ^{O}C for 7 h. Sample was taken from the reaction mixture, injected into LC-MS to monitor the generation of thiazolidine and demethylated peptide products **3u** and **3u'**. The reaction mixture was analyzed by HPLC using method A to determine the % conversion. We did not observe **3u'**.

N,N-dimethyl-NTMT biotin thiazolidine peptide products 3u. LCMS: *m/z* 1157.60685 (calcd [M+H]+ = 1157.6071), *m/z* 579.30346 (calcd [M+2/2]+ = 579.3035), Purity: >95 % (HPLC analysis at 220 nm). Retention time in HPLC: 6.953

HPLC trace of reaction

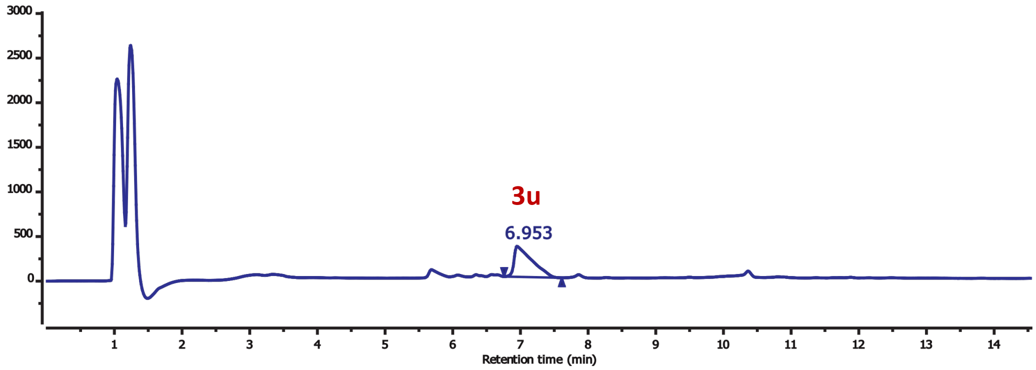

Mass spectra of biotin thiazolidine peptide products (3u)

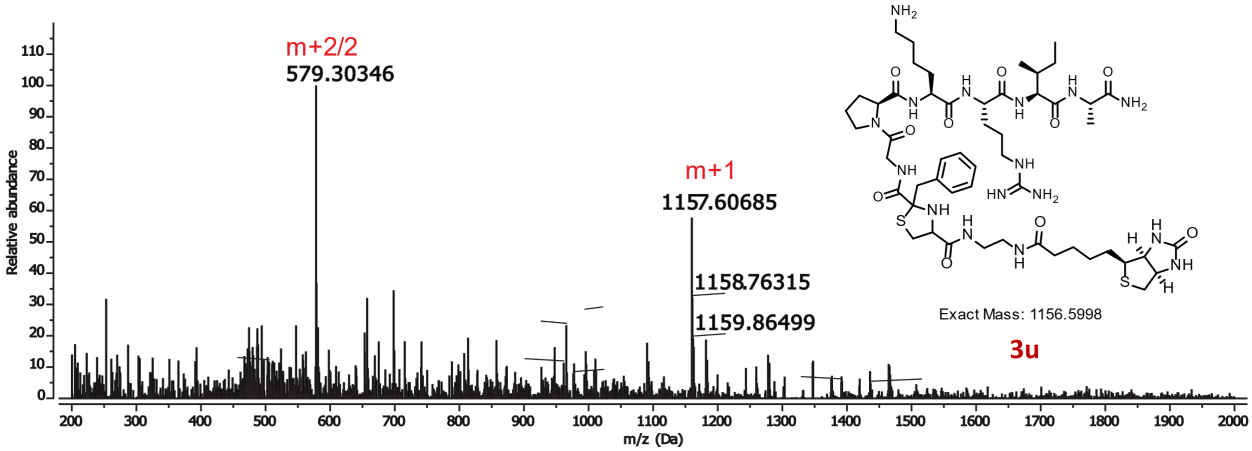

XXX. Supplementary Figure 23. Nitrilation of cell lysate spiked N,N-dimethyl containing peptides.

To 100 µg of LnCap whole cell lysate in 200 µL of NaP buffer pH 7.0 was added 0.1 mg of cytochrome c-557 peptide **1t,** 0.1 mg of myosin light chain 1 peptide **1v**, and 0.1 mg of histone H2B peptide **1w**. To this mixture of cell lysate and histone peptides was added pyridine and selectfluor (10 equiv. each with respect to peptides) and sodium cyanide (3 equiv. with respect to peptides). The reaction mixture was stirred for 1 h. Samples were taken from the reaction mixture, injected into LC-MS to monitor the modification of N,N-dimethyl containing peptides to nitrile products. The reaction mixture was analyzed by HPLC using method A.

MS-Trace of the Cell lysate after the reaction

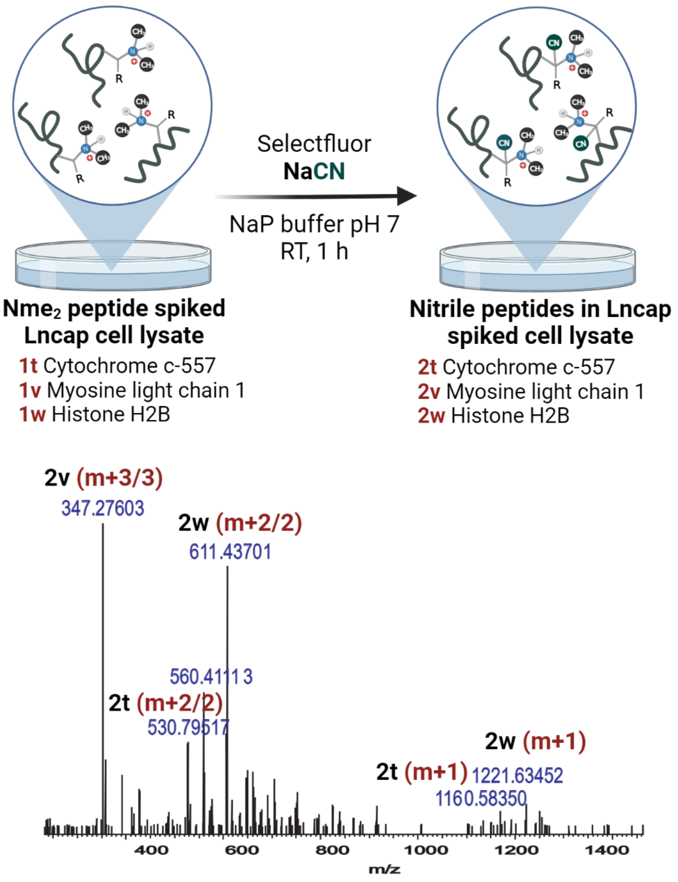

XXXI. Supplementary Figure 24. Incorporation of N-terminal dimethyl on proteins.

General procedure for selective N-terminal dimethylation of proteins: To 0.5 mg of protein in 1 mL of 10 mM sodium acetate buffer (pH 5), 500 µL of stock formaldehyde solution (1 µL of 37% formaldehyde in 1 mL water) was added. The reaction was vortexed for 2 min, followed by the addition of 500 µL of 600 mM $NaBH_3CN$ solution in water, and vortexed for additional 2 min. The reaction was incubated at room temperature for 6 min, filtered through a molecular weight cut off (3 kDa) to remove small molecules and the resulting purified protein was analyzed by MS.

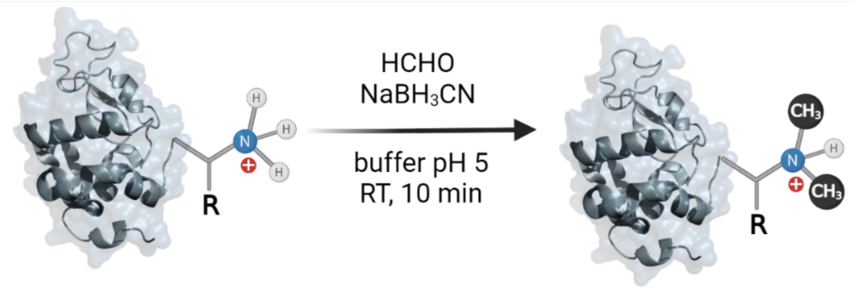

Selective N-terminal dimethylation of aprotinin:

MS trace of unmodified aprotinin (molecular weight = 6511)

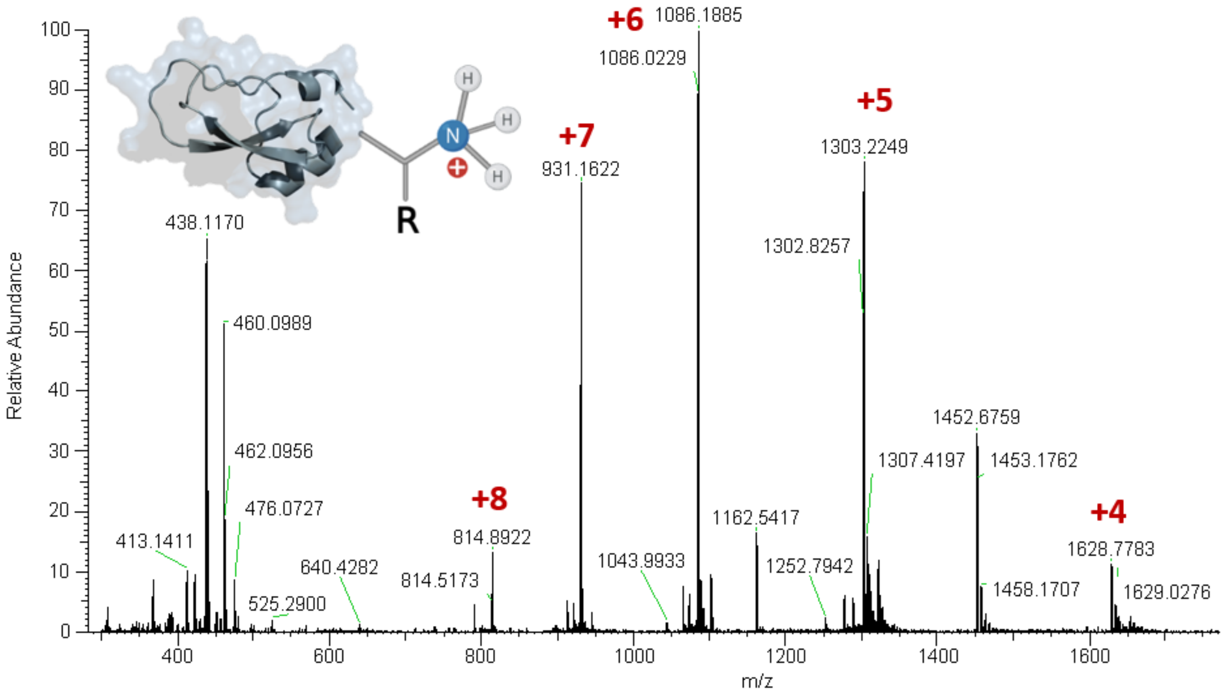

MS trace of N,N-terminal dimethyl aprotinin (molecular weight = 6539)

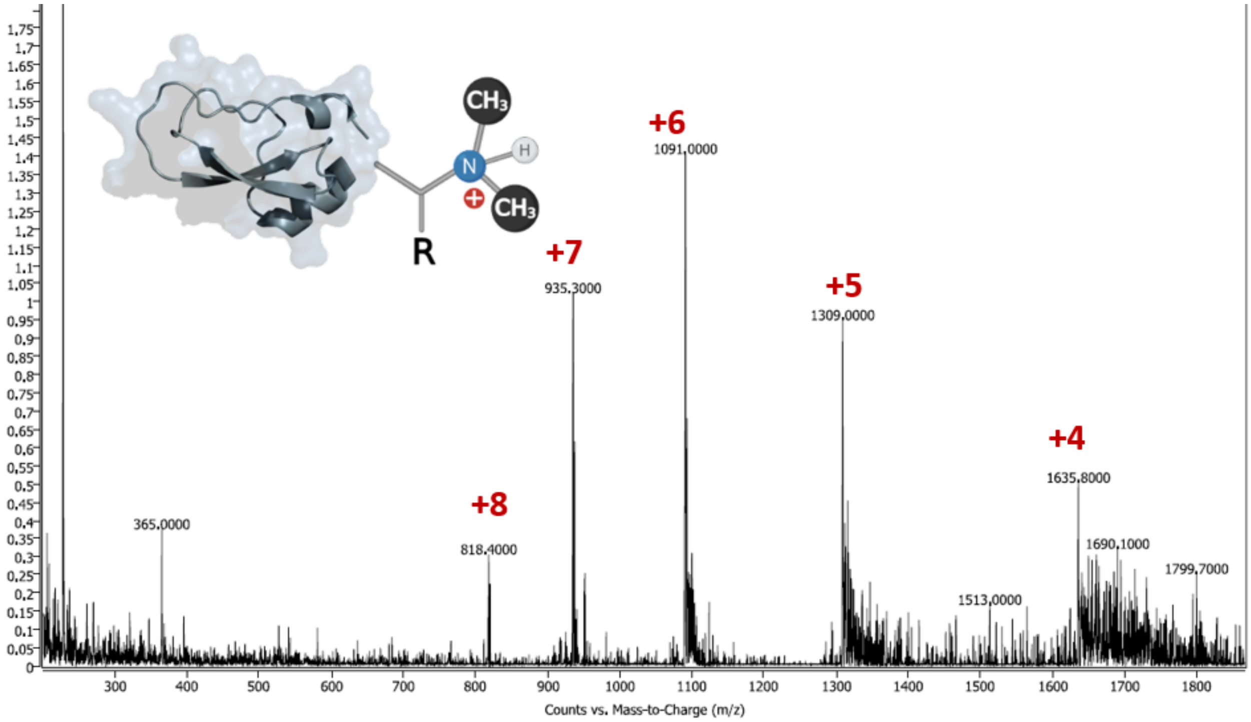

Selective N-terminal dimethylation of ubiquitin:

MS trace of unmodified ubiquitin (molecular weight = 8564)

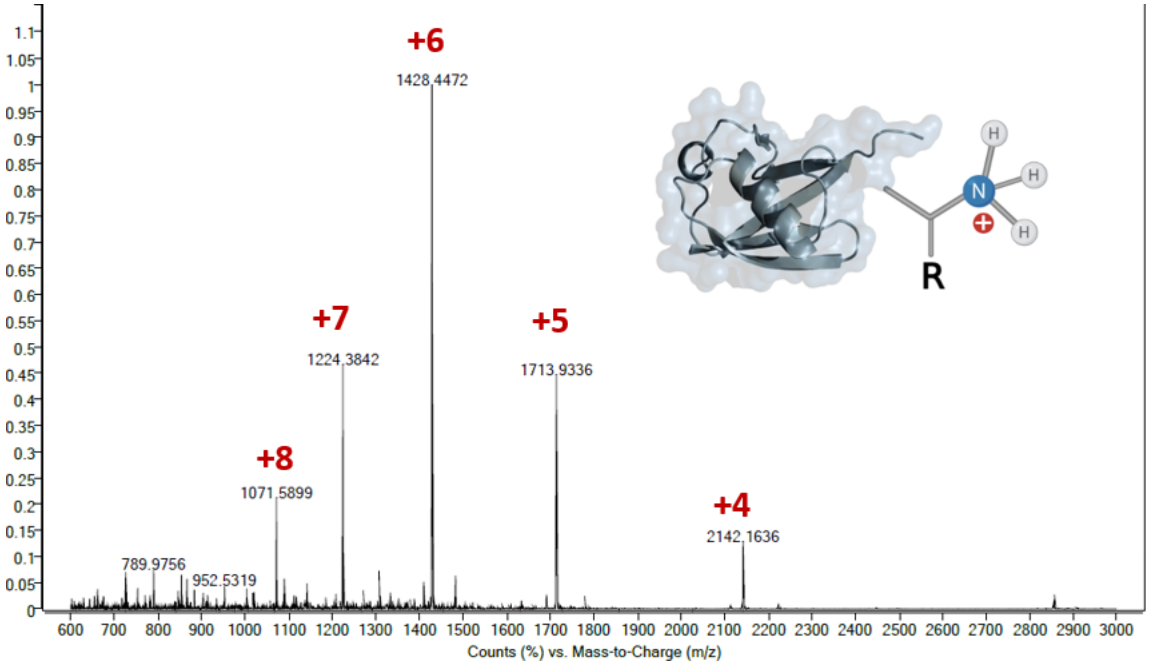

Deconvoluted mass spectra of unmodified ubiquitin (8565)

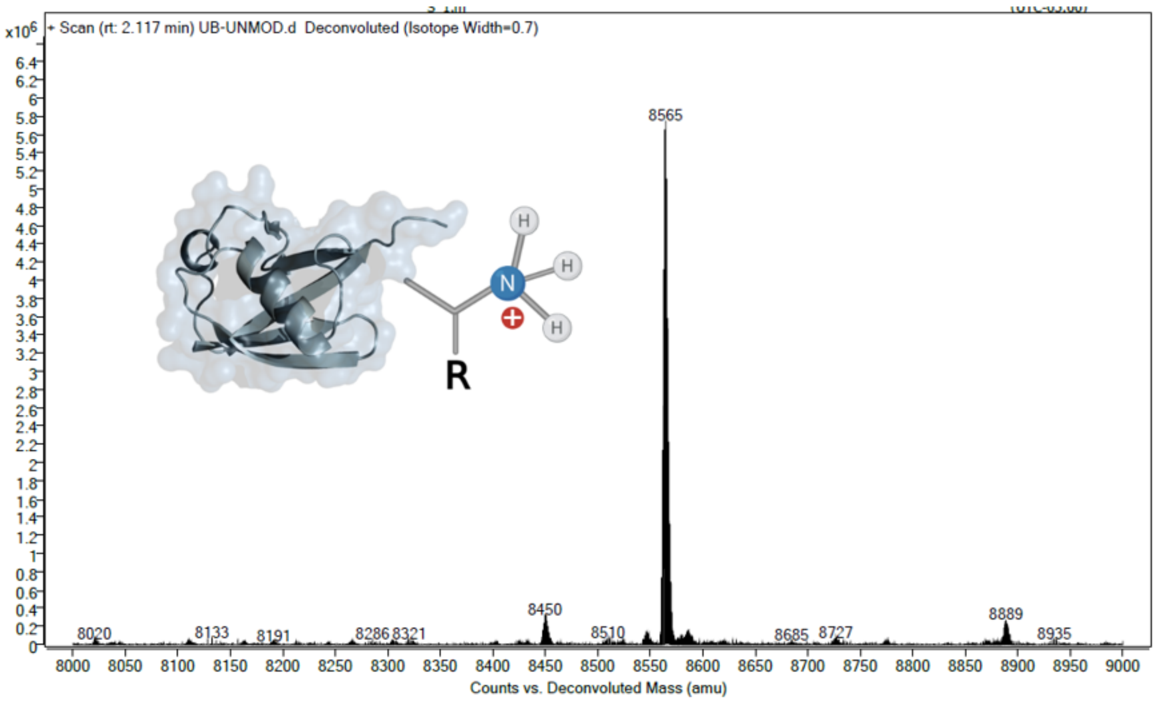

MS trace of N,N-terminal dimethyl ubiquitin (molecular weight = 8593)

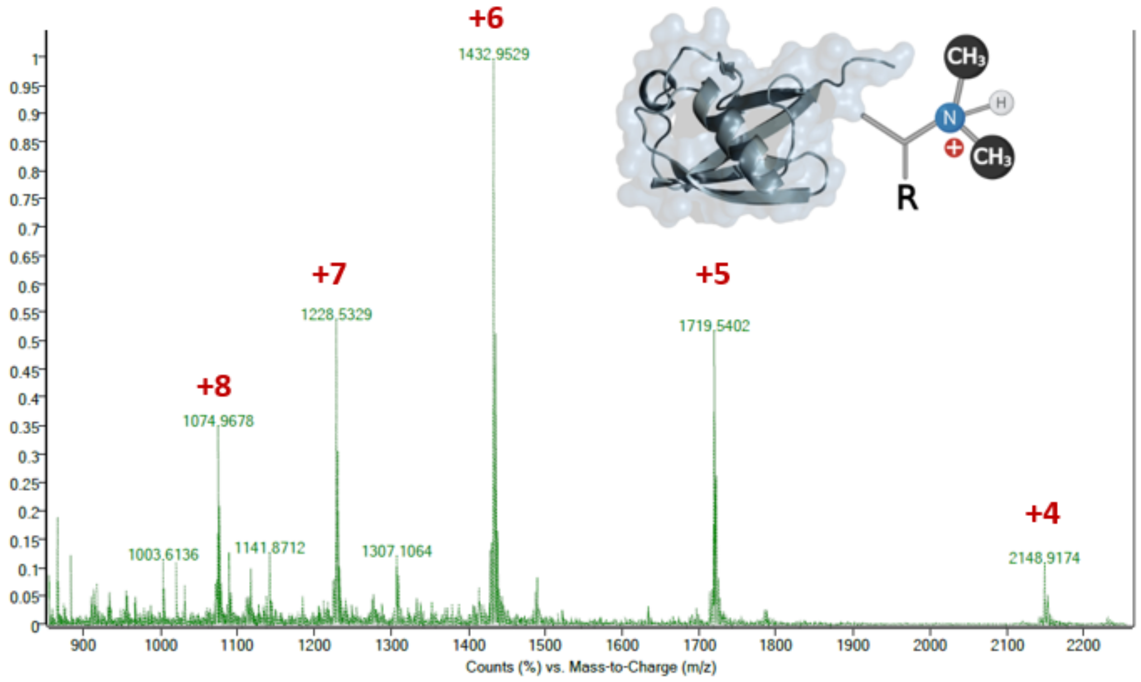

Deconvoluted mass spectra of N,N-terminal dimethyl ubiquitin (8593)

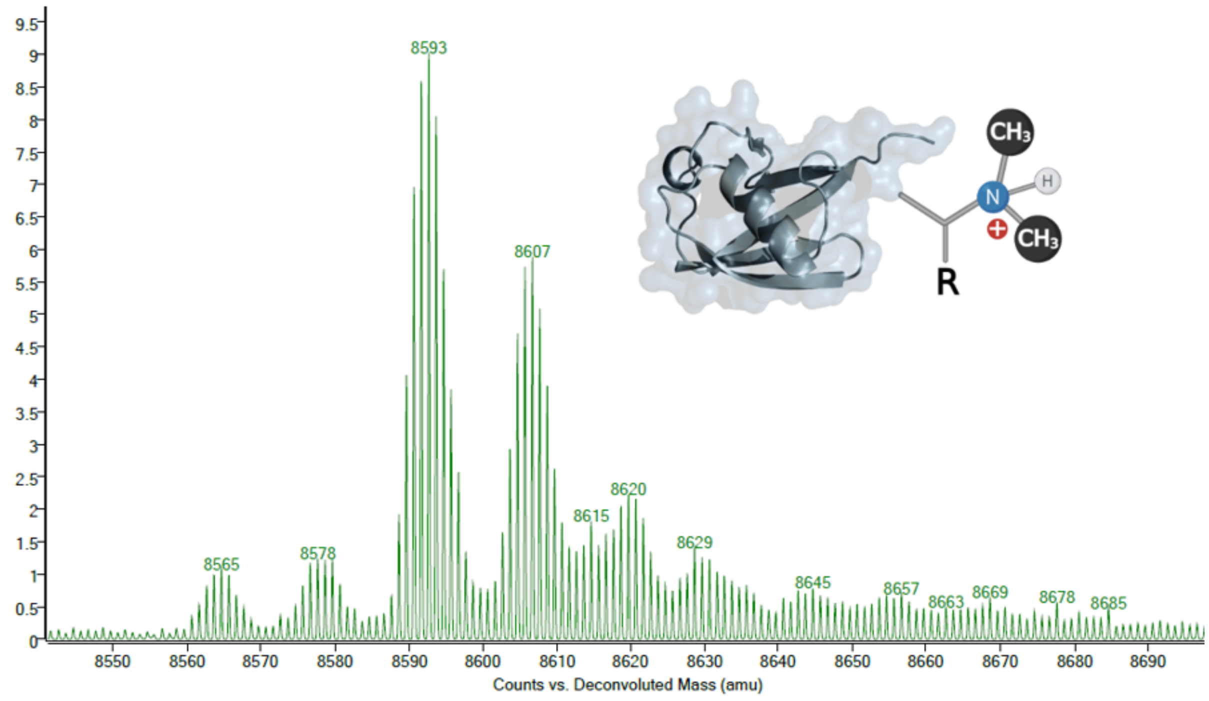

Selective N-terminal dimethylation of insulin:

MS trace of unmodified insulin (molecular weight = 5807)

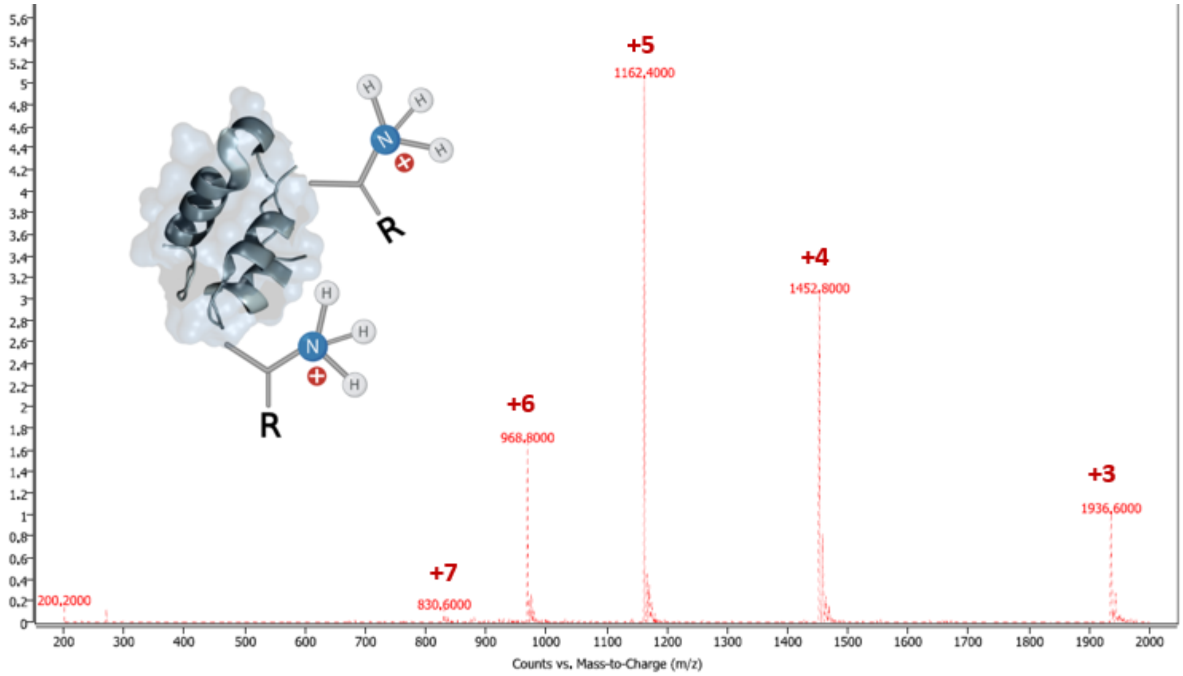

MS trace of N,N-terminal dimethyl insulin (molecular weight = 5863)

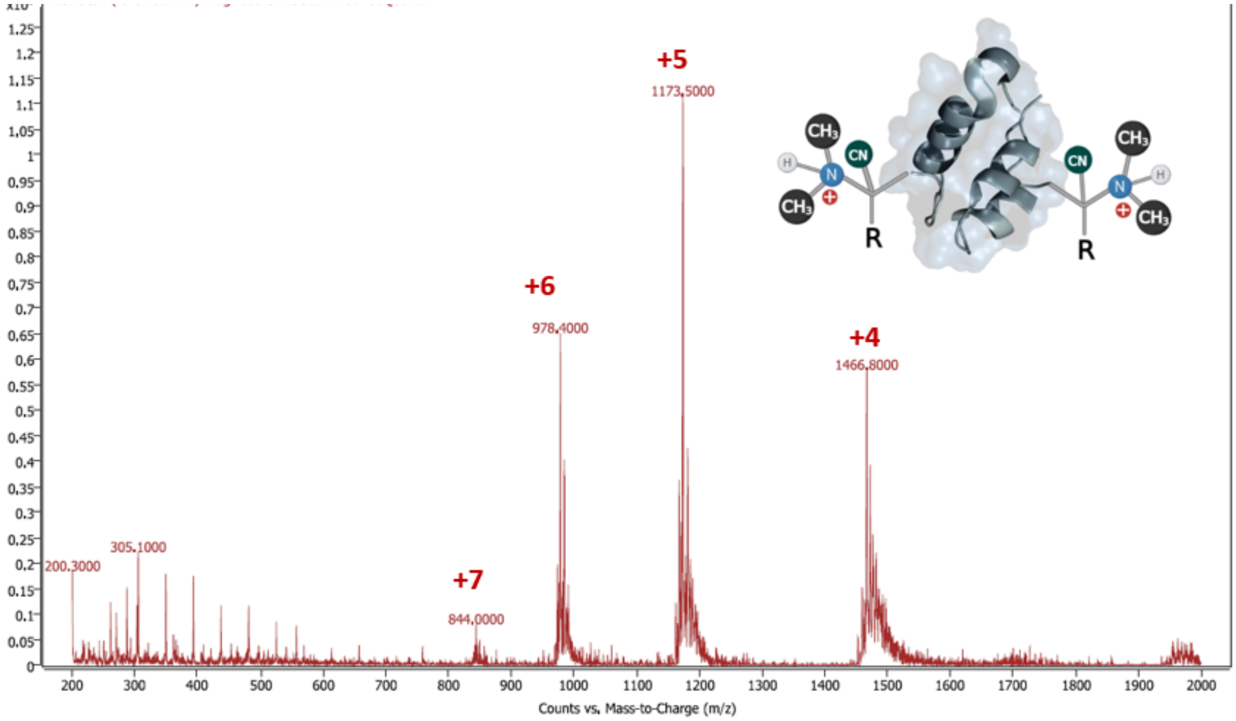

XXXII. Supplementary Figure 25. Nitrile modification of N-terminal dimethyl residues on proteins.

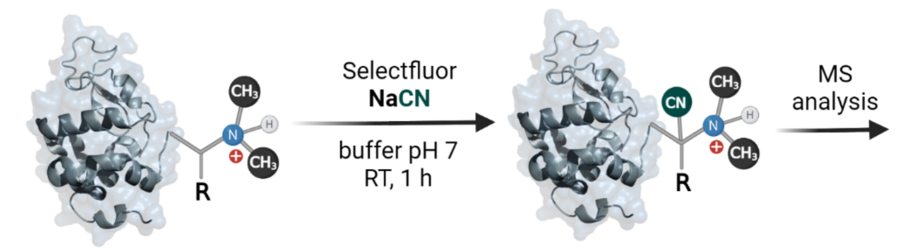

Nitrile modification of Nme$_2$ aprotinin: To 0.5 mg Nme$_2$ containing aprotinin in 300 µL of NaP buffer pH 7 (10 mM), was added 1 mg of selectfluor and 1 mg of NaCN. The reaction was put on a shaker at room temperature for 1 h. Reaction was filtered with a 3 kDa molecular weight cutoff filter, and protein resuspended in 300 µL of NaP buffer. Samples were taken from the reaction mixture, injected into LC-MS to monitor the modification of N,N-dimethyl aprotinin to nitrile product.
<u>In aprotinin</u>, the only residue that is modified in addition to the N,N-dimethyl terminus (Nme$_2$) is methionine which forms a stable sulfoxide product. The mass for this is clearly accounted for in the mass spectrum as we see a mass difference of 41 from the unmodified (sulfoxide =16, nitrile addition = 25, the loss of 1 from oxidation of the alpha proton). The sulfoxide product does not interfere in the enrichment and proteomic analysis of the nitrile product from Nme$_2$.

Nme$_2$ aprotinin molecular weight: 6539
Unmodified Nme$_2$ aprotinin sequence:
(me$_2$)RPDFCLEPPYTGPCKARIIRYFYNAKAGLCQTFVYGGCRAKRNNFKSAEDCMRTCGGAI GPWENL
Nitrile modified aprotinin molecular weight + (sulfoxide on methionine): 6580

MS trace of nitrile modified aprotinin (molecular weight = 6580)

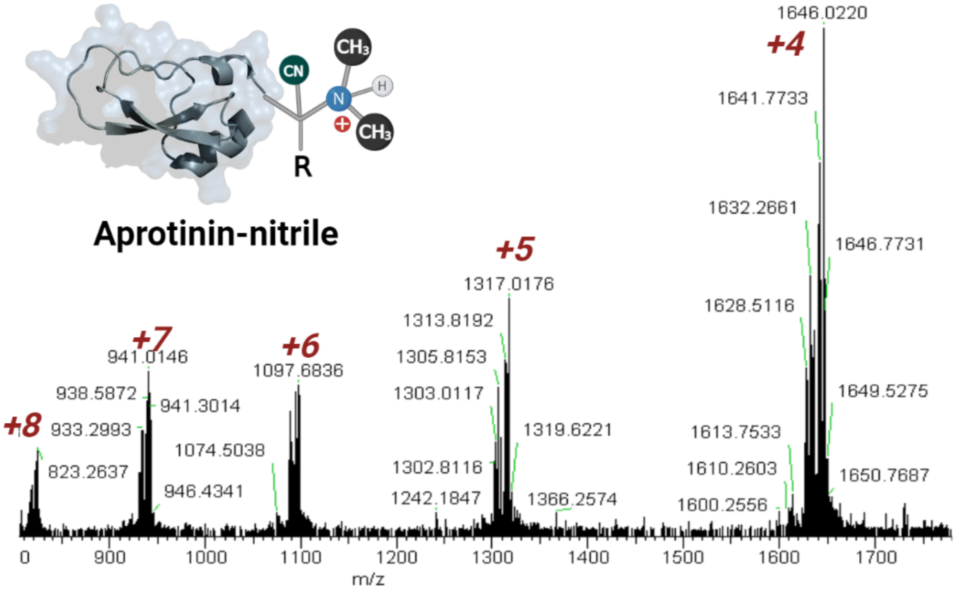

Nitrile modification of Nme_2 Ubiquitin: To 0.5 mg Nme_2 containing ubiquitin in 300 µL of NaP buffer pH 7 (10 mM) was added 1 mg of selectfluor and 1 mg of NaCN. The reaction was stirred at room temperature for 1 h. Reaction was filtered with a 3 kDa molecular weight cutoff filter, and protein resuspended in 300 µL of NaP buffer. Samples were taken from the reaction mixture, injected into LC-MS to monitor the modification of N,N-dimethyl ubiquitin to nitrile products.

In Ubiquitin, the only residue that is modified in addition to the N,N-dimethyl terminus (Nme_2) is methionine which forms a stable sulfoxide product. The mass for this is clearly accounted for the mass spectrum as we see a mass difference of 41 from the unmodified (sulfoxide =16, nitrile addition = 25, the loss of 1 from oxidation of the alpha proton). The sulfoxide product does not interfere in the enrichment and proteomic analysis of the nitrile product from Nme_2.

Nme_2 Ubiquitin molecular weight: 8592
Nitrile modified Ubiquitin molecular weight + (sulfoxide on methionine): 8633

MS trace of nitrile modified Ubiquitin (8633)

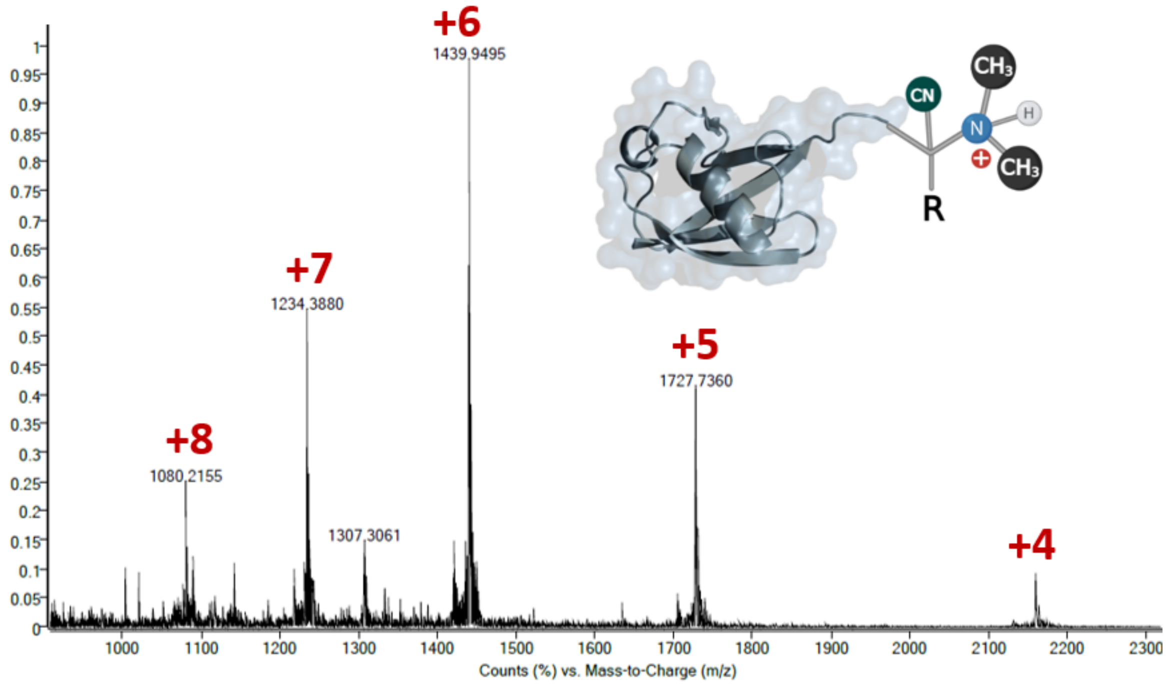

Deconvoluted mass spectra of nitrile modified Ubiquitin (8634)

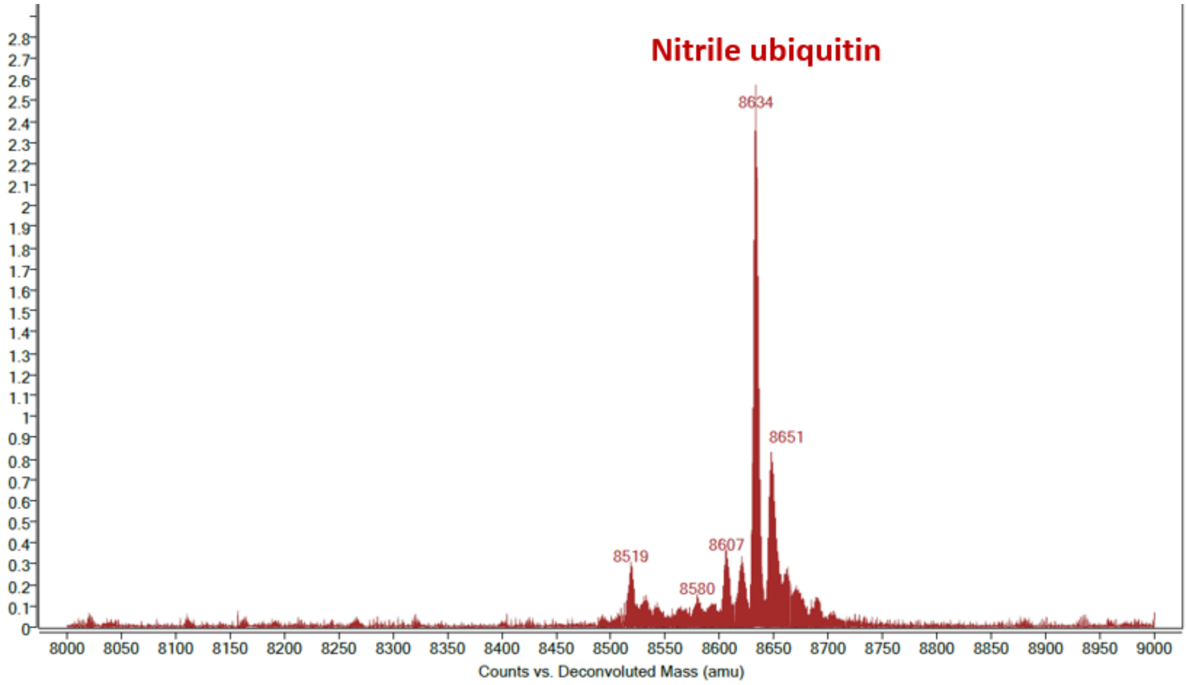

Nitrile modification of Nme_2 Insulin: To 0.5 mg Nme_2 containing insulin in 300 µL of NaP buffer pH 7 (10 mM) was added 1 mg of selectfluor and 1 mg of NaCN. The reaction was stirred at room temperature for 1 h. Reaction was filtered with a 3 kDa molecular weight cutoff filter, and protein resuspended in 300 µL of NaP buffer. Samples were taken from the reaction mixture, injected into LC-MS to monitor the modification of N,N-dimethyl insulin to nitrile products.

In Insulin, the only residue that is modified in addition to the to the N,N-dimethyl terminus (Nme_2) is histidine which forms a stable fluorination side-product as observed from peptide studies reported in the chemoselectivity section of the paper. Insulin has 2 histidine residues and the mass for the observed fluorination is clearly accounted for in the mass spectrum as we see a mass difference of 90 from the unmodified (2 histidine fluorination =40, 2 nitrile addition = 50, the loss of 2 from oxidation of the alpha proton from chain A and B of insulin). Also, the mass spectrum also shows the nitrile product without histidine fluorination with a mass difference of 50 (2 nitrile addition = 50). The histidine fluorination product does not interfere in the enrichment and proteomic analysis of the nitrile product from Nme_2.

Nme_2 Insulin molecular weight: 5863
Unmodified Nme_2 Insulin sequence:

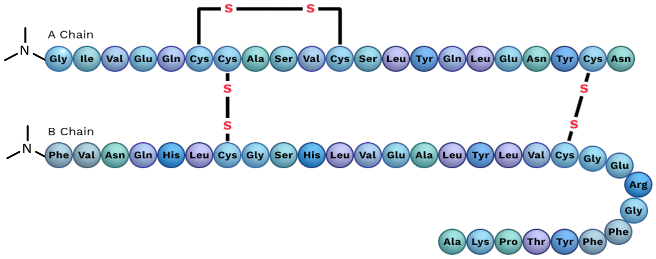

Nitrile modified Insulin molecular weight: 5913
Nitrile modified Insulin molecular weight + (2-histidine fluorination): 5953.

MS trace of nitrile modified Insulin

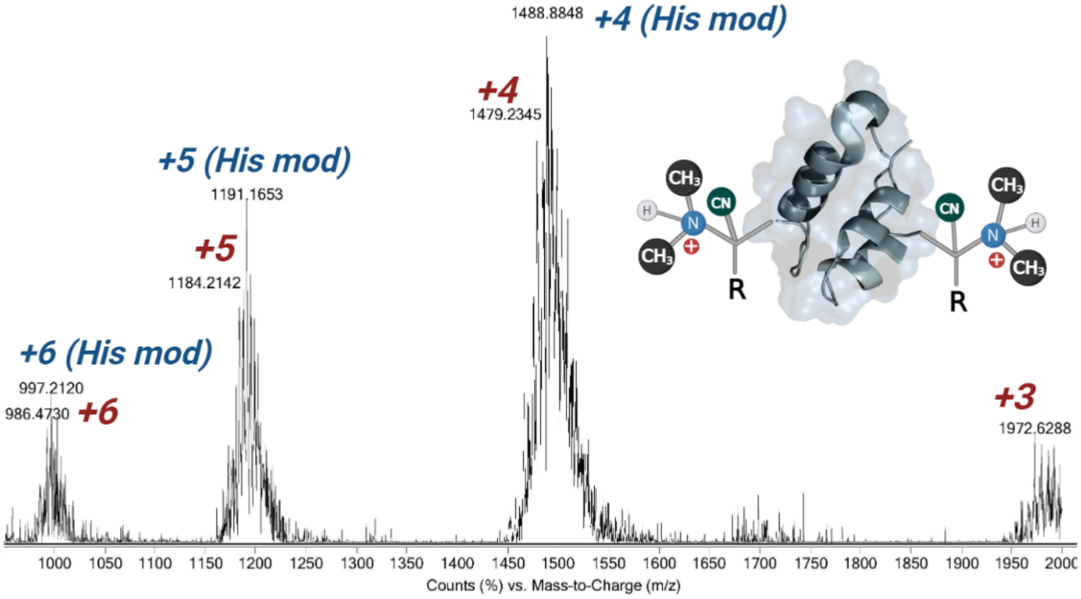

XXXIII. Supplementary Figure 26. Thiazolidination of N-terminal dimethyl containing proteins with cysteine-alkyne tags.

Thiazolidine modification of nitrile-Aprotinin

To 1 mg nitrile modified Aprotinin in 500 µL of NaP buffer pH 7 (10 mM), and 500 µL isopropyl alcohol, was added 100 µL of 200 mM solution of cysteine-alkyne affinity tag in water. The reaction was stirred at 70 ^{O}C for 10 h. Reaction was diluted with 1 mL of water and filtered with a 3 kDa molecular weight cutoff, and proteins resuspended in 300 µL of water and injected into LC-MS to monitor the modification of thiazolidine modified N,N-dimethyl aprotinin.

MS trace of ketone intermediate of Aprotinin (6528)

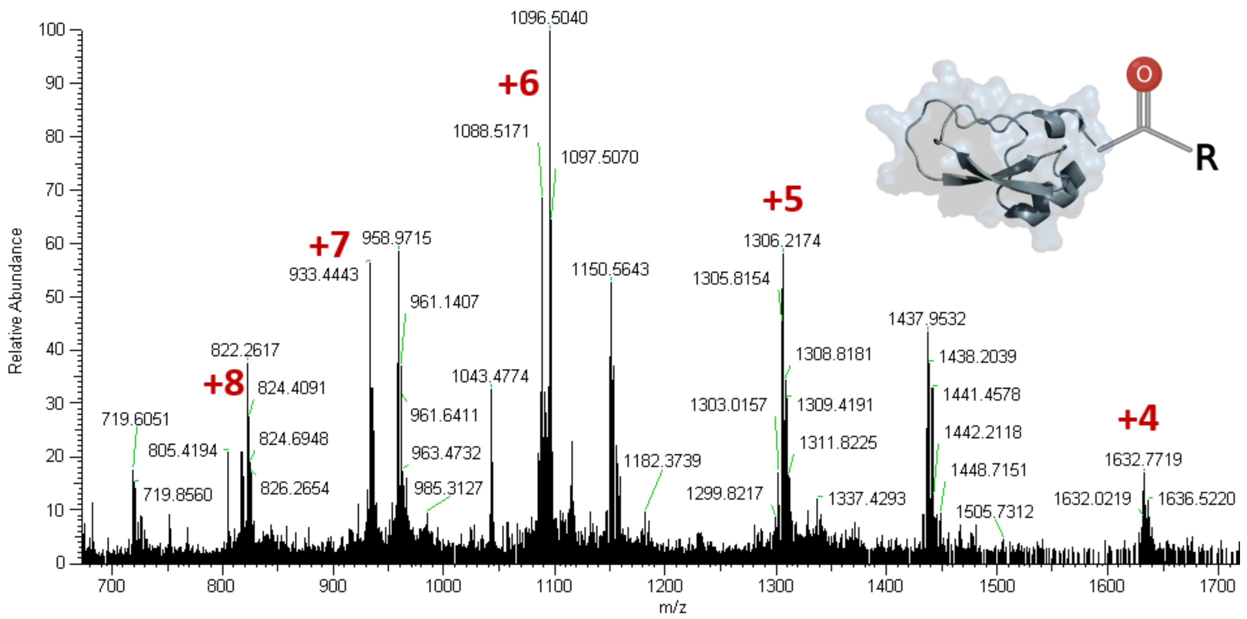

MS trace of thiazolidine modified aprotinin (6668)

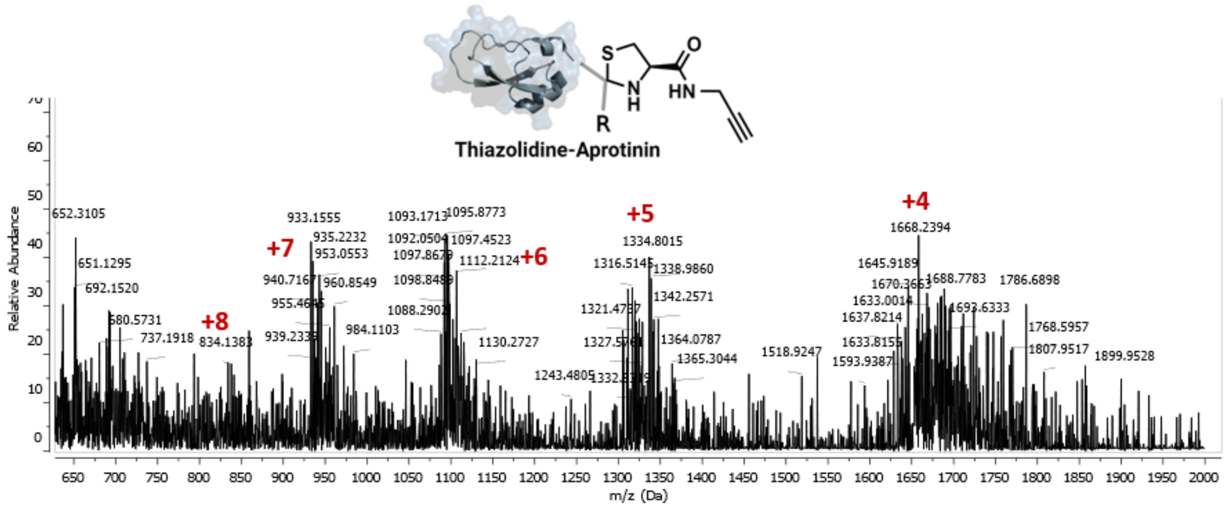

Thiazolidine modification of nitrile-ubiquitin

To 1 mg nitrile modified ubiquitin in 500 µL of NaP buffer pH 7 (10 mM) and 500 µL isopropyl alcohol, was added 100 µL of 200 mM solution of cysteine-alkyne affinity tag in water. The reaction was stirred at 70 ^{O}C for 10 h. Reaction was diluted with 1 mL of water and filtered with a 3 kDa molecular weight cutoff, and proteins resuspended in 300 µL of water and injected into LC-MS to monitor the modification of thiazolidine modified N,N-dimethyl aprotinin.

MS trace of ketone and thiazolidine modified ubiquitin (8722)

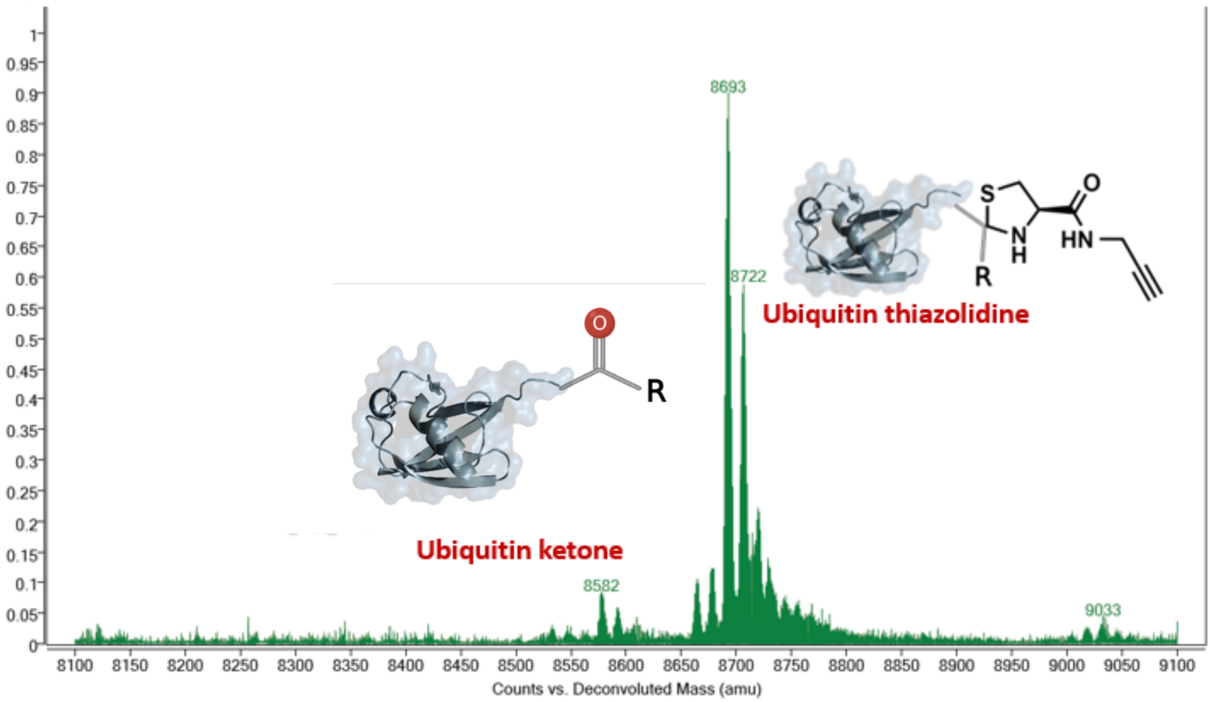

XXXIV Supplementary Figure 27. Thiazolidination of N-terminal dimethyl containing lysate with cysteine-alkyne tags and labeling with fluorophore.

Cell Culture and Drugs. Cells were maintained at 37 °C and 5% CO2. T47D cells were cultured in RPMI supplemented with 10% (V/V) fetal bovine serum (FBS) and 1% (V/V) penicillin/streptomycin (100 µg/mL).

Cell Lysis. Whole cell lysate was generated by lysing cells on ice in RIPA buffer (50 mM TrisHCl [pH 8], 150 mM NaCl, 1% NP-40, 0.5% sodium deoxycholate, 0.1% SDS) supplemented with protease and phosphatase inhibitors. Lysates were centrifuged 6,500 x g, 10 m at 4°C, and soluble lysate was collected. Whole cell lysate proteins were separated using 16% SDS-PAGE. SDS-PAGE gels were stained with Coomassie brilliant blue dye.

Alkylation of cysteines in cell lysates. To 1mg of T47D cell lysate in 500 µL of PBS buffer pH 7.4 (10 mM), 500 µL of 15mM of iodoacetamide in water was added and reaction stirred in the dark for 30 min. Proteins in cell lysate were acetone precipitated to remove small molecules.

Generation of Nme_2 on cell lysate. To 1 mg of alkylated T47D proteins in 500 µL of 10 mM sodium acetate buffer (pH 5), 500 µL of 10% formaldehyde solution in water was added. The reaction was vortexed for 2 min, followed by the addition of 500 µL of 600 mM $NaBH_3CN$ solution in water, and vortexed for additional 2 min. The reaction was incubated at room temperature for 16 min, and acetone precipitated to obtain Nme_2 containing proteins. Proteins were resuspended in 500 µL NaP buffer pH 7 (10 mM).

OxNiTha modification of Nme_2 containing lysates and conjugation with Cy5 azide fluorophore. To 500 μg of Nme_2 modified lysate in 500 μL of 10 mM NaP buffer pH 7 was added 4 mg of selectfluor and 10 mg of sodium cyanide. The reaction was stirred at room temperature for 1 h. Samples were subjected to acetone precipitation to remove small molecules and obtain pure proteins. Proteins were resuspended in 300 μL of 10 mM NaP buffer pH 7 and 300 μL of isopropyl alcohol was added 100 μL of 200 mM cysteine-alkyne probe in water and was incubated at 70 ^{O}C for 10 h. Upon completion of reaction, another round of acetone precipitation was performed, followed by Cy5 azide fluorophore labeling using Click chemistry. Proteins were dissolved in 200 μL of water, followed by the addition of 50 μL of 100 mM TBTA in water, 50 μL of freshly prepared 100 mM ascorbic acid in water, 50 μL of 50 mM of CuSO4 in water, and 2 μL of 10 mM Cy5 azide in DMSO. The reaction was stirred for 1 h and acetone precipitated, followed by analysis of proteins through in gel fluorescence imaging and Coomassie blue staining.

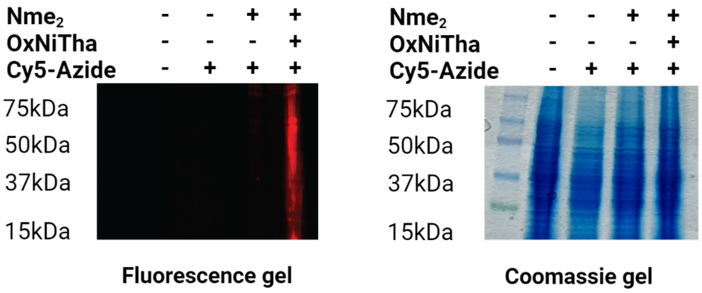

VII. Supplementary Figure 28. Thiazolidination of N-terminal dimethyl containing lysate and enrichment with azide-functionalized resin.

Solid-support mediated synthesis of azide functionalized resin. Azide-resin was synthesized manually on a 0.25 mm scale using Rink amide resin. Resin was swollen with CH_2Cl_2 for 1 h at room temperature. Fmoc was deprotected using 20 % piperidine–DMF for 5 min to obtain a deprotected peptide-resin. First, 2-azidoacetic acid (1.25 mm/5 equiv.) was coupled on resin using HOAt (1.25 mm/5 equiv.) and DIC (1.25 mm/5 equiv.) in DMF for 30 min at room temperature. Resin was subsequently washed 3 times with MeOH, DMF, and CH_2Cl_2. Resin was stored under a vacuum.

OxNiTha modification of Nme_2 containing lysates and enrichment using an azide functionalized resin. To 1 mg of alkylated Nme_2-proteins in 500 μL of 10 mM NaP buffer pH 7 was added 8 mg of selectfluor and 20 mg of sodium cyanide. The reaction was stirred at room temperature for 1 h. Samples were subjected to acetone precipitation to remove small molecules and obtain pure proteins. Proteins were resuspended in 600 μL 10 mM NaP buffer pH 7 and 600 μL of isopropyl alcohol. 100 μL of 200 mM solution of cysteine-alkyne probe in water was added and reaction incubated at 70 ^{O}C for 10 h. Upon completion of reaction, another round of acetone precipitation was performed. Proteins were dissolved in 400 μL of water, followed by the addition of 200 μL of 100 mM TBTA in water, 200 μL of 100 mM freshly prepared ascorbic acid in water, 200 μL of 50 mM of $CuSO_4$ in water. Reaction mixture was introduced into a 5 mL reaction vial containing 200 mg of azide-resin in 1 mL of THF. The

reaction was stirred for 5 h. Upon completion of reaction, the mixture was vortexed for 1 min and the supernatant was carefully removed. Resin was further washed with 4% SDS solution and with PBS buffer pH 7 followed by the careful removal of supernatant. The bound proteins on resin were detached by treatment with 95% TFA in water for 2 h. Eluate was air-dried and neutralized with 1N NaOH solution. Samples were loaded on a Novex WedgeWell 4-20% Tris-Glycine gel. Gel was run in Tris-glycine running buffer at 180V. The gel was then stained with Coomassie brilliant blue for 1 h and destained overnight.

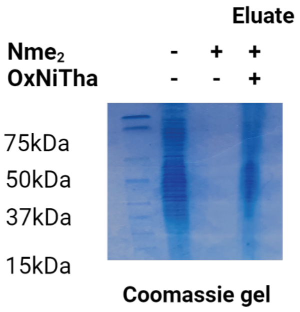

Proteomics analysis of enriched proteins. Enriched proteins obtained from azide-resin were digested using Thermo scientific EasyPep Mini MS sample Prep Kit. Digested peptides were analyzed by LC/MS. Database search of peptides was performed with Proteome Discoverer 3.0. Analysis of enriched peptides and database mapping to proteins identified 1394 protein. A similar search on the negative control sample identified 15 proteins.

XXXVI. Supplementary Figure 29. On-bead digestion of enriched Nme_2 proteins.

On-bead digestion. For on-bead digestion, a published protocol was followed. To the bead, digestion buffer (50 mM NH_4HCO_3) was added, and the mixture was then treated with 1 mM dithiothreitol (DTT) at RT for 30 min, followed by 5 mM iodoacetamide (IAA) at RT for 30 min in the dark. Proteins were digested with 2 µg of lysyl endopeptidase (Wako) at RT for overnight and were further digested overnight with 2 µg trypsin (Promega) at RT. Resulting peptides were desalted with HLB column (Waters) and were dried under vacuum.

LC-MS/MS. LC-MS/MS was performed at MS Bioworks. Half of each digested sample was analyzed by nano LC-MS/MS with a Waters M-Class HPLC system interfaced to a ThermoFisher Fusion Lumos mass spectrometer. Peptides were loaded on a trapping column and eluted over a 75µm analytical column at 350nL/min; both columns were packed with Luna C18 resin (Phenomenex). A 1 h gradient was employed. The mass spectrometer was operated in data-dependent mode, with the Orbitrap operating at 60,000 FWHM and 15,000 FWHM for MS and MS/MS respectively. APD was enabled and the instrument was run with a 3s cycle for MS and MS/MS.

Database search (Proteome Discoverer). Mass spectrometry data was analyzed according to a published protocol.[3] Spectra were searched using Proteome Discoverer 2.1 against 2020 human UniProtKB/Swiss-Prot database (20,379 target sequences). Searching parameters included fully tryptic restriction, precursor mass tolerance (± 20 ppm), and fragment mass tolerance (± 0.05 Da). Methionine oxidation (+15.99492 Da), asaparagine and glutamine deamidation (+0.98402 Da) and protein N-terminal acetylation (+42.03670) were variable modifications (up to 3 allowed per peptide); cysteine was assigned a fixed carbamidomethyl

modification (+57.021465 Da). Percolator was used to filter the peptide spectrum matches (PSMs) to a false discovery rate of 1%.

Statistical Analysis of peptide spectrum matches (PSMs) and unique peptides. Analysis of PSMs and unique peptides for positive and negative control samples were done using Perseus 2.0. Numerical data were $\log_2$ transformed and valid values filtered for. Imputation was done on missing values and two sample t-test performed with FDR = 0.01. Volcano plot of the results was done with GraphPad Prism 8.0.

XXXVII. References

1. Chan, W. C.; White, P. D. Fmoc solid phase peptide synthesis: A practical approach (Oxford Univ. Press, New York, 2000).
2. Soucek, S.; Zeng, Y.; Bellur, D. L.; Bergkessel, M.; Morris, K. J.; Deng, Q.; Duong, D.; Seyfried, N. T.; Guthrie, C.; Staley, J. P.; Fasken, M. B.; Corbett, A. H. Evolutionarily conserved polyadenosine RNA binding protein nab2 cooperates with splicing machinery to regulate the fate of pre-mrna. *Mol. Cell. Biol.* **2016**, 36, 2697–2714.
3. Wingo, T. S.; Duong, D. M.; Zhou, M.; Dammer, E. B.; Wu, H.; Cutler, D. J.; Lah, J. J.; Levey, A. I.; Seyfried, N. T. Integrating next-generation genomic sequencing and mass spectrometry to estimate allele-specific protein abundance in human brain. *J. Proteome Res.* **2017**, 16, 3336–3347.
